# Supplementary material for: Thermal conditions and age structure determine the spawning regularities and condition of Baltic herring (Clupea harengus membras) in the NE of the Baltic Sea
Source: PeerJ. 2019 Jul 22;7:e7345. doi: 10.7717/peerj.7345 (PMC6657675; doi:10.7717/peerj.7345)
Supplement: Appendix 1 — The length, weight, condition factor (K), age, sex, calendar week of sampling and year of analysed Baltic herring. [file peerj-07-7345-s001.pdf]

The length, weight, condition factor (K), age, sex, calendar week of sampling and year of analysed Baltic herring.

LENGTH, cm	WEIGHT, g	K	AGE, yrs	SEX	week	year
16	23,2	0,6	4	2	23	2003
15	18,5	0,5	3	2	20	1999
14,2	18,3	0,6	2	2	25	2003
12,2	12,9	0,7	2	2	25	2007
30,5	16,3	0,1	2	2	19	2012
15,5	9,8	0,3	1	2	26	2001
18,4	18,4	0,3	2	2	22	2000
13,9	8,2	0,3	2	2	22	2007
17,1	18,5	0,4	4	2	19	2012
14,4	11,2	0,4	3	2	24	2001
21,5	37,4	0,4	8	2	17	2012
14,7	12,5	0,4	3	2	18	2002
15,9	15,9	0,4	5	2	21	2014
14,3	11,6	0,4	4	2	21	2003
15	13,6	0,4	3	2	21	2001
14	11,2	0,4	4	2	23	1999
14,9	13,6	0,4	3	2	20	2008
13,8	10,9	0,4	2	2	26	2001
14	11,4	0,4	3	2	25	2004
15,3	14,9	0,4	5	2	19	2006
13,2	9,7	0,4	2	2	20	2007
14,8	13,8	0,4	5	2	23	2010
14,8	13,8	0,4	5	2	23	2010
15,7	16,5	0,4	8	2	23	2010
15,7	16,5	0,4	8	2	23	2010
16,9	20,7	0,4	4	2	20	2001

16,9	20,9	0,4	4	2	18	2004
19,3	31,2	0,4	5	2	18	2012
13,2	10	0,4	2	2	20	2013
14,2	12,5	0,4	4	2	23	2011
18,7	28,7	0,4	10+	2	17	2014
16,2	19	0,4	5	2	19	2004
17	22,2	0,5	8	2	21	2010
17	22,2	0,5	8	2	21	2010
14	12,4	0,5	3	2	18	2007
16,9	21,9	0,5	5	2	21	2000
13,5	11,2	0,5	3	2	19	2015
14,7	14,5	0,5	3	2	20	2004
14,1	12,8	0,5	4	2	20	2009
18,3	28	0,5	10	2	15	2015
13,7	11,8	0,5	3	2	21	2009
14	12,6	0,5	5	2	20	2012
14,7	14,6	0,5	3	2	20	2008
15,4	16,8	0,5	6	2	20	2004
17,9	26,5	0,5	4	2	19	2013
14	12,7	0,5	3	2	19	2009
13,3	10,9	0,5	3	2	25	2004
16,2	19,7	0,5	10	2	19	2015
14,1	13	0,5	2	2	19	2004
16,2	19,8	0,5	5	2	23	2001
15,3	16,7	0,5	3	2	18	2015
21,4	45,7	0,5	10	2	19	2015
15,9	18,8	0,5	3	2	23	2001
14,3	13,7	0,5	4	2	26	2006

12,1	8,3	0,5	1	2	20	2013
16,1	19,6	0,5	6	2	20	2013
14	12,9	0,5	2	2	20	2013
11,3	6,8	0,5	2	2	23	2004
14,5	14,4	0,5	4	2	25	2004
16,8	22,4	0,5	7	2	19	2012
15,2	16,6	0,5	6	2	25	2003
14	13	0,5	3	2	25	2004
15,8	18,7	0,5	4	2	25	2003
14,1	13,3	0,5	8	2	26	2006
16,2	20,2	0,5	6	2	23	2010
16,2	20,2	0,5	6	2	23	2010
16,7	22,2	0,5	4	2	19	2014
15,6	18,1	0,5	4	2	25	2004
13,4	11,5	0,5	3	2	22	2011
13,4	11,5	0,5	2	2	20	2013
15,5	17,8	0,5	5	2	18	2007
13,2	11	0,5	2	2	20	2002
16,1	20,1	0,5	7	2	19	2009
15,8	19	0,5	5	2	21	2001
15,1	16,6	0,5	4	2	20	2009
17	23,7	0,5	6	2	22	2004
18,4	30,1	0,5	6	2	22	2011
16,1	20,2	0,5	6	2	22	2011
15,2	17	0,5	5	2	22	2009
13,3	11,4	0,5	2	2	23	2009
13,1	10,9	0,5	2	2	20	2013
13,6	12,2	0,5	3	2	23	2010

13,6	12,2	0,5	3	2	23	2010
14,4	14,5	0,5	5	2	20	2010
14,4	14,5	0,5	5	2	20	2010
15,7	18,8	0,5	5	2	20	2010
15,7	18,8	0,5	5	2	20	2010
15,3	17,4	0,5	5	2	23	2010
15,3	17,4	0,5	5	2	23	2010
15	16,4	0,5	3	2	18	2015
14,9	16,1	0,5	3	2	20	2012
13,5	12	0,5	3	2	23	2010
13,5	12	0,5	3	2	23	2010
14,7	15,5	0,5	3	2	21	2010
14,7	15,5	0,5	5	2	21	2010
14,7	15,5	0,5	3	2	21	2010
14,7	15,5	0,5	5	2	21	2010
17,1	24,4	0,5	8	2	19	2012
14,2	14	0,5	4	2	21	2009
14,4	14,6	0,5	3	2	17	2014
13,6	12,3	0,5	2	2	20	2013
14,3	14,3	0,5	4	2	22	2011
18,1	29	0,5	6	2	20	2013
16,4	21,6	0,5	3	2	20	2003
15,6	18,6	0,5	3	2	20	2012
14,7	15,6	0,5	4	2	15	2015
13,2	11,3	0,5	3	2	23	2010
13,2	11,3	0,5	3	2	23	2010
16,8	23,3	0,5	5	2	24	2006
16,7	22,9	0,5	6	2	16	2015

15,8	19,4	0,5	5	2	17	2009
14	13,5	0,5	3	2	22	2011
14,5	15	0,5	4	2	26	2006
14,4	14,7	0,5	3	2	20	2010
14,4	14,7	0,5	3	2	20	2010
15,4	18	0,5	5	2	23	1999
16,2	21	0,5	4	2	25	2003
14,7	15,7	0,5	2	2	18	2000
12,3	9,2	0,5	2	2	23	2010
12,3	9,2	0,5	2	2	23	2010
15	16,7	0,5	3	2	22	2001
14,6	15,4	0,5	5	2	19	2010
14,6	15,4	0,5	5	2	19	2010
14,9	16,4	0,5	3	2	20	2012
14,3	14,5	0,5	2	2	19	2001
14,2	14,2	0,5	3	2	26	2006
15,6	18,9	0,5	3	2	18	2015
14,6	15,5	0,5	3	2	20	2010
14,6	15,5	0,5	3	2	20	2010
13,1	11,2	0,5	6	2	20	2009
15,2	17,5	0,5	4	2	22	2011
13,8	13,1	0,5	2	2	20	2012
14,4	14,9	0,5	4	2	21	2009
14,3	14,6	0,5	4	2	21	2011
14,3	14,6	0,5	3	2	19	2015
14,1	14	0,5	4	2	19	2015
11,7	8	0,5	2	2	25	2004
15,9	20,1	0,5	3	2	17	2014

17,3	25,9	0,5	6	2	21	2010
17,3	25,9	0,5	6	2	21	2010
16,7	23,3	0,5	5	2	25	2003
16	20,5	0,5	4	2	22	2003
15	16,9	0,5	8	2	23	2001
15	16,9	0,5	6	2	22	2007
14,9	16,6	0,5	3	2	21	2006
14,4	15	0,5	3	2	20	2003
13,1	11,3	0,5	1	2	15	2015
13,9	13,5	0,5	2	2	19	2012
14,3	14,7	0,5	3	2	18	2015
14	13,8	0,5	3	2	23	2015
16	20,6	0,5	4	2	21	2001
11	6,7	0,5	2	2	23	2004
12	8,7	0,5	2	2	26	2006
15	17	0,5	3	2	20	2003
15	17	0,5	4	2	25	2003
15	17	0,5	5	2	23	2010
15	17	0,5	5	2	23	2010
13,2	11,6	0,5	3	2	19	2015
15,5	18,8	0,5	5	2	23	2010
15,5	18,8	0,5	5	2	23	2010
15,5	18,8	0,5	4	2	20	2013
16,6	23,1	0,5	4	2	20	2013
15,9	20,3	0,5	4	2	17	2008
15,1	17,4	0,5	7	2	23	2015
16,4	22,3	0,5	4	2	15	2015
14,4	15,1	0,5	4	2	23	2001

16,2	21,5	0,5	3	2	18	2002
16,2	21,5	0,5	4	2	16	2015
11,7	8,1	0,5	2	2	19	2006
14,8	16,4	0,5	4	2	21	2010
14,8	16,4	0,5	4	2	21	2010
14	13,9	0,5	2	2	19	2001
14,1	14,2	0,5	3	2	18	2015
15	17,1	0,5	5	2	26	2006
15	17,1	0,5	4	2	15	2015
17,4	26,7	0,5	5	2	20	2012
15,2	17,8	0,5	4	2	21	2011
16,8	24,1	0,5	4	2	16	2015
15,6	19,3	0,5	5	2	22	2009
14,5	15,5	0,5	3	2	21	2010
14,5	15,5	0,5	3	2	21	2010
13,6	12,8	0,5	3	2	18	2008
13,6	12,8	0,5	2	2	19	2010
13,6	12,8	0,5	2	2	19	2010
17,2	25,9	0,5	8	2	20	2013
13,7	13,1	0,5	2	2	22	2009
15	17,2	0,5	4	2	21	2011
15	17,2	0,5	4	2	20	2013
16,9	24,6	0,5	5	2	20	2012
13	11,2	0,5	3	2	20	2007
14,2	14,6	0,5	2	2	26	2003
14,7	16,2	0,5	3	2	20	2010
14,7	16,2	0,5	3	2	20	2010
13,3	12	0,5	4	2	21	2009

14,1	14,3	0,5	2	2	19	2013
14	14	0,5	2	2	20	2012
16,3	22,1	0,5	6	2	20	2013
16,2	21,7	0,5	4	2	23	2001
14,9	16,9	0,5	8	2	26	2005
14,6	15,9	0,5	4	2	19	1999
14,6	15,9	0,5	5	2	20	2007
15,3	18,3	0,5	4	2	23	2011
14,5	15,6	0,5	4	2	21	2009
12,5	10	0,5	2	2	18	2002
15,4	18,7	0,5	6	2	20	2001
15,8	20,2	0,5	4	2	20	2013
16,8	24,3	0,5	5	2	23	2001
16	21	0,5	5	2	20	2012
16,2	21,8	0,5	8	2	23	2008
13,6	12,9	0,5	3	2	21	2010
13,6	12,9	0,5	3	2	21	2010
17	25,2	0,5	8	2	20	2013
14,3	15	0,5	4	2	19	2009
14,3	15	0,5	3	2	19	2015
11,4	7,6	0,5	2	2	22	2011
13,7	13,2	0,5	2	2	19	2013
13,7	13,2	0,5	2	2	20	2013
14,2	14,7	0,5	3	2	21	2010
14,2	14,7	0,5	3	2	21	2010
14,2	14,7	0,5	3	2	16	2015
14,1	14,4	0,5	3	2	21	2014
14,1	14,4	0,5	3	2	23	2014

14	14,1	0,5	3	2	17	2008
14,6	16	0,5	3	2	20	2012
15,7	19,9	0,5	5	2	20	2006
13	11,3	0,5	2	2	17	2014
15,4	18,8	0,5	6	2	20	2013
18,5	32,6	0,5	7	2	19	2015
14,8	16,7	0,5	5	2	19	2009
14,8	16,7	0,5	3	2	23	2010
14,8	16,7	0,5	3	2	23	2010
13,4	12,4	0,5	2	2	20	2010
13,4	12,4	0,5	2	2	20	2010
15,2	18,1	0,5	4	2	22	2011
15	17,4	0,5	3	2	21	2001
15	17,4	0,5	3	2	23	2008
15	17,4	0,5	3	2	21	2010
15	17,4	0,5	3	2	21	2010
12,3	9,6	0,5	2	2	21	2009
15,6	19,6	0,5	3	2	20	2010
15,6	19,6	0,5	3	2	20	2010
14,7	16,4	0,5	3	2	21	2010
14,7	16,4	0,5	3	2	21	2010
14,7	16,4	0,5	4	2	23	2011
15,3	18,5	0,5	5	2	21	2010
15,3	18,5	0,5	5	2	21	2010
13,6	13	0,5	4	2	18	2015
15,1	17,8	0,5	6	2	20	2013
12,9	11,1	0,5	2	2	22	2007
12,5	10,1	0,5	2	2	18	2007

15,8	20,4	0,5	4	2	17	2009
13,7	13,3	0,5	3	2	23	2010
13,7	13,3	0,5	3	2	23	2010
14,1	14,5	0,5	3	2	18	2008
14,1	14,5	0,5	3	2	20	2010
14,1	14,5	0,5	3	2	20	2010
16,7	24,1	0,5	4	2	19	2015
15,4	18,9	0,5	6	2	20	2013
13,8	13,6	0,5	2	2	23	2001
14	14,2	0,5	3	2	18	2015
14,8	16,8	0,5	4	2	17	2009
14,8	16,8	0,5	4	2	23	2010
14,8	16,8	0,5	4	2	23	2010
14,8	16,8	0,5	6	2	23	2011
15,2	18,2	0,5	3	2	23	2001
14,5	15,8	0,5	5	2	21	2010
14,5	15,8	0,5	5	2	21	2010
14,5	15,8	0,5	4	2	22	2011
15	17,5	0,5	4	2	23	2004
13,3	12,2	0,5	4	2	26	2006
13	11,4	0,5	2	2	23	2006
17	25,5	0,5	8	2	15	2015
15,7	20,1	0,5	4	2	20	2013
14,7	16,5	0,5	5	2	19	1999
14,7	16,5	0,5	3	2	18	2015
13,4	12,5	0,5	2	2	20	2013
14,9	17,2	0,5	4	2	18	2008
14,2	14,9	0,5	2	2	16	2008

13,1	11,7	0,5	2	2	21	2001
13,1	11,7	0,5	3	2	22	2011
14,6	16,2	0,5	3	2	21	2005
14,6	16,2	0,5	3	2	16	2015
13,6	13,1	0,5	2	2	26	2006
13,6	13,1	0,5	4	2	21	2009
14,1	14,6	0,5	6	2	20	2011
16,5	23,4	0,5	5	2	20	2013
16,8	24,7	0,5	6	2	20	2013
14	14,3	0,5	2	2	19	2001
13,8	13,7	0,5	2	2	19	2013
13,9	14	0,5	2	2	20	2012
14,8	16,9	0,5	5	2	21	2010
14,8	16,9	0,5	5	2	21	2010
15	17,6	0,5	4	2	19	2009
15	17,6	0,5	4	2	21	2014
15,6	19,8	0,5	5	2	21	2010
15,6	19,8	0,5	5	2	21	2010
14,5	15,9	0,5	7	2	20	2009
16,7	24,3	0,5	5	2	20	2012
11,9	8,8	0,5	2	2	20	2007
15,8	20,6	0,5	6	2	23	2010
15,8	20,6	0,5	6	2	23	2010
12,7	10,7	0,5	2	2	21	2009
14,7	16,6	0,5	4	2	21	2009
15,4	19,1	0,5	3	2	25	2003
15,4	19,1	0,5	4	2	19	2010
15,4	19,1	0,5	4	2	19	2010

17	25,7	0,5	10	2	22	2007
17,3	27,1	0,5	10	2	23	1999
13	11,5	0,5	2	2	19	2014
13	11,5	0,5	2	2	19	2015
14,2	15	0,5	4	2	15	2015
15,2	18,4	0,5	6	2	23	2001
15,2	18,4	0,5	5	2	24	2001
18,2	31,6	0,5	8	2	20	2010
18,2	31,6	0,5	8	2	20	2010
13,5	12,9	0,5	2	2	20	2013
14,8	17	0,5	4	2	21	2005
14,8	17	0,5	4	2	19	2009
15	17,7	0,5	3	2	23	2001
12,4	10	0,5	2	2	23	2004
14,5	16	0,5	4	2	20	2000
17	25,8	0,5	4	2	20	2013
16,5	23,6	0,5	3	2	22	2003
15,4	19,2	0,5	4	2	23	2001
15,4	19,2	0,5	4	2	19	2004
14,7	16,7	0,5	3	2	19	2008
14,7	16,7	0,5	3	2	19	2010
14,7	16,7	0,5	3	2	19	2010
18,9	35,5	0,5	6	2	20	2013
16,4	23,2	0,5	7	2	22	2009
13,2	12,1	0,5	2	2	20	2013
15,5	19,6	0,5	6	2	20	2008
14,3	15,4	0,5	2	2	24	2001
14,3	15,4	0,5	4	2	25	2004

15,2	18,5	0,5	6	2	20	2013
15,2	18,5	0,5	4	2	16	2015
16,2	22,4	0,5	4	2	17	2014
16,1	22	0,5	6	2	26	2006
12,7	10,8	0,5	2	2	25	2004
15	17,8	0,5	3	2	18	2015
15,3	18,9	0,5	5	2	19	2010
15,3	18,9	0,5	5	2	19	2010
15,3	18,9	0,5	4	2	15	2015
13,4	12,7	0,5	2	2	20	2010
13,4	12,7	0,5	2	2	20	2010
14,1	14,8	0,5	3	2	23	2001
14,1	14,8	0,5	3	2	17	2008
13	11,6	0,5	3	2	21	2008
11,9	8,9	0,5	2	2	18	2007
16,4	23,3	0,5	4	2	20	2001
13,5	13	0,5	3	2	26	2006
13,5	13	0,5	2	2	21	2008
14	14,5	0,5	3	2	20	2012
13,6	13,3	0,5	2	2	19	2013
16,3	22,9	0,5	6	2	25	2004
13,8	13,9	0,5	3	2	26	2006
14,4	15,8	0,5	3	2	15	2015
16,2	22,5	0,5	4	2	18	2001
13,1	11,9	0,5	3	2	21	2005
17,4	27,9	0,5	6	2	23	2001
15,7	20,5	0,5	4	2	19	2001
16,5	23,8	0,5	4	2	19	2002

15,8	20,9	0,5	5	2	20	2012
14,3	15,5	0,5	2	2	21	2003
14,3	15,5	0,5	2	2	19	2008
14,6	16,5	0,5	4	2	21	2009
18,3	32,5	0,5	6	2	19	2015
15	17,9	0,5	7	2	22	2009
15,3	19	0,5	8	2	19	2010
15,3	19	0,5	6	2	20	2010
15,3	19	0,5	8	2	19	2010
15,3	19	0,5	6	2	20	2010
16,4	23,4	0,5	5	2	23	2010
16,4	23,4	0,5	5	2	23	2010
14,8	17,2	0,5	4	2	25	2004
12,1	9,4	0,5	2	2	26	2006
11,7	8,5	0,5	2	2	21	2006
18,2	32	0,5	5	2	20	2012
14,2	15,2	0,5	4	2	26	2006
14,2	15,2	0,5	3	2	19	2007
14,2	15,2	0,5	5	2	23	2010
14,2	15,2	0,5	5	2	23	2010
15,4	19,4	0,5	4	2	19	2012
13,3	12,5	0,5	3	2	19	2015
14,5	16,2	0,5	4	2	20	2004
14,5	16,2	0,5	3	2	21	2006
14,5	16,2	0,5	3	2	20	2010
14,5	16,2	0,5	3	2	20	2010
17,4	28	0,5	4	2	19	2012
14,1	14,9	0,5	3	2	20	2001

14,1	14,9	0,5	3	2	19	2015
16,2	22,6	0,5	8	2	21	2003
14,7	16,9	0,5	3	2	19	2010
14,7	16,9	0,5	3	2	19	2010
14,9	17,6	0,5	3	2	20	2008
14	14,6	0,5	3	2	19	2015
15,7	20,6	0,5	3	2	20	2010
15,7	20,6	0,5	3	2	20	2010
12	9,2	0,5	2	2	22	2011
15,8	21	0,5	3	2	20	2001
15,8	21	0,5	8	2	26	2003
13,5	13,1	0,5	2	2	23	2001
12,5	10,4	0,5	3	2	21	2005
14,4	15,9	0,5	4	2	26	2009
14,4	15,9	0,5	3	2	23	2010
14,4	15,9	0,5	3	2	23	2010
15,2	18,7	0,5	5	2	18	2014
13	11,7	0,5	2	2	20	2012
13,6	13,4	0,5	3	2	23	2010
13,6	13,4	0,5	3	2	23	2010
13,8	14	0,5	5	2	25	2004
13,8	14	0,5	2	2	18	2008
13,8	14	0,5	3	2	22	2011
13,8	14	0,5	2	2	19	2012
15,3	19,1	0,5	5	2	21	2000
15	18	0,5	5	2	18	2015
16,6	24,4	0,5	7	2	20	2012
14,3	15,6	0,5	5	2	25	2005

14,3	15,6	0,5	4	2	26	2009
16,8	25,3	0,5	6	2	21	2010
16,8	25,3	0,5	6	2	21	2010
14,8	17,3	0,5	4	2	19	2004
13,1	12	0,5	2	2	17	2014
11,9	9	0,5	2	2	18	2007
16,5	24	0,5	6	2	17	2008
16,5	24	0,5	4	2	20	2013
14,2	15,3	0,5	5	2	22	2007
15,1	18,4	0,5	3	2	21	2015
14,5	16,3	0,5	2	2	23	2001
14,5	16,3	0,5	2	2	23	2010
14,5	16,3	0,5	2	2	23	2010
16	21,9	0,5	4	2	20	2013
15,6	20,3	0,5	10	2	18	2007
13,2	12,3	0,5	6	2	21	2009
13,2	12,3	0,5	2	2	19	2012
12,4	10,2	0,5	3	2	26	2006
12,4	10,2	0,5	2	2	26	2006
12,4	10,2	0,5	2	2	20	2013
16,4	23,6	0,5	6	2	20	2013
14,1	15	0,5	3	2	21	2009
14,7	17	0,5	3	2	19	2015
17	26,3	0,5	4	2	19	2010
17	26,3	0,5	4	2	19	2010
15,2	18,8	0,5	5	2	25	2004
13,3	12,6	0,5	2	2	23	2010
13,3	12,6	0,5	3	2	23	2010

13,3	12,6	0,5	2	2	23	2010
13,3	12,6	0,5	3	2	23	2010
13,3	12,6	0,5	2	2	20	2013
14	14,7	0,5	3	2	23	2011
14	14,7	0,5	3	2	21	2015
15,3	19,2	0,5	4	2	20	1999
13,4	12,9	0,5	3	2	19	2015
13,9	14,4	0,5	2	2	19	2013
18,1	31,8	0,5	5	2	17	2014
15	18,1	0,5	5	2	19	2000
15	18,1	0,5	7	2	22	2009
15	18,1	0,5	6	2	20	2011
15	18,1	0,5	9	2	21	2014
15	18,1	0,5	9	2	23	2014
13,5	13,2	0,5	2	2	23	2001
14,6	16,7	0,5	3	2	20	2012
14,6	16,7	0,5	3	2	18	2015
15,4	19,6	0,5	3	2	25	2003
13,6	13,5	0,5	2	2	21	2008
13,7	13,8	0,5	2	2	20	2001
13,7	13,8	0,5	3	2	23	2003
14,8	17,4	0,5	4	2	20	2012
12,7	11	0,5	2	2	23	2009
15,5	20	0,5	3	2	22	2003
13	11,8	0,5	2	2	21	2005
13	11,8	0,5	2	2	20	2007
13	11,8	0,5	2	2	21	2010
13	11,8	0,5	2	2	21	2010

13	11,8	0,5	2	2	20	2012
16,4	23,7	0,5	5	2	20	2009
15,1	18,5	0,5	4	2	21	2009
15,1	18,5	0,5	3	2	20	2012
15,6	20,4	0,5	3	2	23	2003
15,6	20,4	0,5	5	2	19	2010
15,6	20,4	0,5	5	2	19	2010
15,9	21,6	0,5	4	2	20	2001
21	49,8	0,5	6	2	16	2015
16,6	24,6	0,5	6	2	20	2013
16,8	25,5	0,5	6	2	19	2004
14,2	15,4	0,5	3	2	23	2009
14,5	16,4	0,5	3	2	19	1999
14,5	16,4	0,5	2	2	21	2001
14,5	16,4	0,5	2	2	18	2002
14,9	17,8	0,5	3	2	18	2008
15,2	18,9	0,5	4	2	19	2000
12	9,3	0,5	2	2	18	2007
13,1	12,1	0,5	2	2	21	2006
14,7	17,1	0,5	2	2	19	2001
18,3	33	0,5	6	2	20	2013
16,2	22,9	0,5	3	2	21	2010
16,2	22,9	0,5	3	2	21	2010
16,9	26	0,5	4	2	17	2009
14,1	15,1	0,5	6	2	26	2006
14,1	15,1	0,5	4	2	23	2009
16,5	24,2	0,5	3	2	18	2001
10,7	6,6	0,5	2	2	23	2004

12,8	11,3	0,5	2	2	19	2009
15,3	19,3	0,5	3	2	19	2015
13,2	12,4	0,5	2	2	21	2009
13,2	12,4	0,5	2	2	20	2013
17,5	28,9	0,5	6	2	18	2015
15	18,2	0,5	3	2	20	2003
15	18,2	0,5	3	2	19	2010
15	18,2	0,5	9	2	19	2010
15	18,2	0,5	3	2	19	2010
15	18,2	0,5	9	2	19	2010
15	18,2	0,5	3	2	19	2015
14	14,8	0,5	3	2	17	2008
14	14,8	0,5	4	2	26	2009
17	26,5	0,5	4	2	15	2015
17	26,5	0,5	4	2	18	2015
15,4	19,7	0,5	5	2	20	2000
15,4	19,7	0,5	3	2	21	2001
15,4	19,7	0,5	4	2	18	2015
15,5	20,1	0,5	3	2	20	2001
15,5	20,1	0,5	8	2	15	2015
12,6	10,8	0,5	3	2	22	2005
13,9	14,5	0,5	2	2	19	2008
11,4	8	0,5	2	2	20	2004
11,4	8	0,5	2	2	18	2007
15,8	21,3	0,5	4	2	15	2015
13,4	13	0,5	2	2	19	2009
14,3	15,8	0,5	3	2	19	2010
14,3	15,8	0,5	3	2	19	2010

13,8	14,2	0,5	3	2	20	2004
16,3	23,4	0,5	6	2	23	2001
12,9	11,6	0,5	2	2	20	2012
13,5	13,3	0,5	2	2	20	2013
13,7	13,9	0,5	3	2	20	2007
13,7	13,9	0,5	3	2	22	2007
13,7	13,9	0,5	3	2	23	2010
13,7	13,9	0,5	3	2	23	2010
13,6	13,6	0,5	2	2	20	2007
13,6	13,6	0,5	2	2	19	2013
16,2	23	0,5	4	2	19	2012
15,2	19	0,5	4	2	25	2004
15,2	19	0,5	4	2	22	2011
17,6	29,5	0,5	7	2	19	2011
14,9	17,9	0,5	3	2	19	2009
14,5	16,5	0,5	8	2	20	2004
14,5	16,5	0,5	6	2	26	2006
14,2	15,5	0,5	2	2	18	2002
17	26,6	0,5	4	2	16	2015
13	11,9	0,5	2	2	18	2002
13	11,9	0,5	3	2	22	2005
13	11,9	0,5	2	2	20	2007
13	11,9	0,5	3	2	17	2014
15,3	19,4	0,5	5	2	21	2010
15,3	19,4	0,5	5	2	21	2010
15,3	19,4	0,5	6	2	21	2011
15,3	19,4	0,5	4	2	23	2011
11,8	8,9	0,5	2	2	21	2005

12,7	11,1	0,5	2	2	21	2006
16	22,2	0,5	6	2	21	2014
16,6	24,8	0,5	3	2	16	2015
15	18,3	0,5	5	2	20	2007
15	18,3	0,5	3	2	19	2008
15	18,3	0,5	3	2	18	2012
15	18,3	0,5	5	2	19	2013
15,5	20,2	0,5	7	2	19	2009
14,4	16,2	0,5	3	2	15	2015
15,8	21,4	0,5	3	2	17	2002
15,6	20,6	0,5	3	2	21	2010
15,6	20,6	0,5	3	2	21	2010
15,7	21	0,5	9	2	25	2004
15,7	21	0,5	3	2	22	2005
13,1	12,2	0,5	2	2	20	2013
14,8	17,6	0,5	4	2	22	2009
17,5	29,1	0,5	5	2	19	2010
17,5	29,1	0,5	5	2	19	2010
14	14,9	0,5	2	2	19	2013
14,6	16,9	0,5	3	2	26	2003
16,7	25,3	0,5	4	2	19	2001
16,7	25,3	0,5	4	2	20	2004
13,2	12,5	0,5	3	2	20	2007
13,9	14,6	0,5	5	2	25	2007
15,2	19,1	0,5	5	2	20	2007
15,2	19,1	0,5	3	2	18	2014
16,1	22,7	0,5	3	2	18	2002
16,1	22,7	0,5	4	2	17	2014

13,8	14,3	0,5	3	2	22	2007
13,8	14,3	0,5	3	2	23	2010
13,8	14,3	0,5	3	2	23	2010
13,4	13,1	0,5	5	2	23	2010
13,4	13,1	0,5	5	2	23	2010
15,3	19,5	0,5	6	2	19	2010
15,3	19,5	0,5	6	2	19	2010
13,7	14	0,5	3	2	20	2009
14,5	16,6	0,5	2	2	23	2001
14,5	16,6	0,5	4	2	21	2009
14,5	16,6	0,5	3	2	20	2010
14,5	16,6	0,5	3	2	20	2010
14,5	16,6	0,5	3	2	17	2014
13,5	13,4	0,5	3	2	26	2006
17,6	29,7	0,5	6	2	19	2000
14,2	15,6	0,5	3	2	23	2010
14,2	15,6	0,5	3	2	23	2010
17,5	29,2	0,5	8	2	16	2015
16,3	23,6	0,5	3	2	19	2001
16,3	23,6	0,5	4	2	19	2015
12,9	11,7	0,5	2	2	22	2009
12,9	11,7	0,5	7	2	23	2010
12,9	11,7	0,5	7	2	23	2010
15	18,4	0,5	4	2	25	2004
15	18,4	0,5	5	2	18	2007
15	18,4	0,5	3	2	20	2010
15	18,4	0,5	3	2	20	2010
12,2	9,9	0,5	2	2	20	2010

12,2	9,9	0,5	2	2	20	2010
12,2	9,9	0,5	2	2	17	2014
15,6	20,7	0,5	4	2	19	2013
17	26,8	0,5	6	2	20	2004
16,2	23,2	0,5	3	2	19	2010
16,2	23,2	0,5	3	2	19	2010
14,1	15,3	0,5	4	2	19	2009
14,4	16,3	0,5	3	2	20	2010
14,4	16,3	0,5	3	2	20	2010
14,4	16,3	0,5	3	2	18	2015
14,8	17,7	0,5	9	2	25	2005
15,1	18,8	0,5	4	2	19	2008
15,1	18,8	0,5	4	2	22	2011
13	12	0,5	3	2	22	2007
13	12	0,5	4	2	23	2009
13	12	0,5	2	2	20	2013
16,8	25,9	0,5	5	2	19	1999
14,6	17	0,5	3	2	19	2010
14,6	17	0,5	3	2	19	2010
16,1	22,8	0,5	4	2	19	2004
16,4	24,1	0,5	6	2	24	2001
16,4	24,1	0,5	6	2	23	2005
14	15	0,5	2	2	20	2003
14	15	0,5	3	2	21	2003
14	15	0,5	3	2	19	2014
15,2	19,2	0,5	3	2	19	2002
16	22,4	0,5	7	2	26	2009
13,1	12,3	0,5	2	2	26	2006

13,1	12,3	0,5	2	2	19	2013
14,9	18,1	0,5	4	2	26	2005
14,9	18,1	0,5	4	2	26	2006
15,3	19,6	0,5	5	2	25	2004
15,3	19,6	0,5	3	2	21	2010
15,3	19,6	0,5	3	2	21	2010
16,3	23,7	0,5	6	2	25	2003
15,9	22	0,5	5	2	20	2007
13,9	14,7	0,5	2	2	19	2013
13,9	14,7	0,5	3	2	19	2013
11,3	7,9	0,5	2	2	18	2014
14,7	17,4	0,5	4	2	22	2009
14,7	17,4	0,5	4	2	20	2011
14,7	17,4	0,5	4	2	18	2015
14,5	16,7	0,5	3	2	19	2001
14,5	16,7	0,5	3	2	21	2010
14,5	16,7	0,5	3	2	21	2010
15,7	21,2	0,5	3	2	22	2001
15,7	21,2	0,5	5	2	21	2009
13,2	12,6	0,5	2	2	19	2009
16,2	23,3	0,5	5	2	19	2014
17,7	30,4	0,5	5	2	19	2000
17,2	27,9	0,5	10	2	25	2007
14,2	15,7	0,5	3	2	19	2002
14,2	15,7	0,5	3	2	22	2005
14,2	15,7	0,5	3	2	18	2007
13,3	12,9	0,5	2	2	20	2002
13,3	12,9	0,5	2	2	19	2013

13,3	12,9	0,5	2	2	20	2013
13,7	14,1	0,5	2	2	21	2003
11	7,3	0,5	1	2	18	2007
13,4	13,2	0,5	2	2	20	2010
13,4	13,2	0,5	2	2	20	2010
13,6	13,8	0,5	2	2	20	2001
16,4	24,2	0,5	3	2	23	2010
16,4	24,2	0,5	3	2	23	2010
13,5	13,5	0,5	3	2	19	2000
16,1	22,9	0,5	4	2	21	2014
14,8	17,8	0,5	3	2	21	2001
14,8	17,8	0,5	6	2	16	2008
14,8	17,8	0,5	3	2	22	2011
14,1	15,4	0,5	3	2	20	2010
14,1	15,4	0,5	3	2	20	2010
14,1	15,4	0,5	3	2	18	2014
11,7	8,8	0,5	2	2	23	2004
14,6	17,1	0,5	5	2	20	2004
15,2	19,3	0,5	4	2	22	2011
12,9	11,8	0,5	2	2	18	2007
12	9,5	0,5	2	2	21	2005
16,5	24,7	0,5	4	2	19	2004
12,6	11	0,5	2	2	19	2013
17,6	30	0,6	6	2	20	2013
14	15,1	0,6	2	2	20	2000
14	15,1	0,6	3	2	22	2005
14	15,1	0,6	3	2	20	2007
14	15,1	0,6	3	2	20	2008

14	15,1	0,6	3	2	22	2011
14	15,1	0,6	3	2	19	2015
15,4	20,1	0,6	5	2	20	2010
15,4	20,1	0,6	5	2	20	2010
15,6	20,9	0,6	3	2	21	2003
12,2	10	0,6	2	2	19	2007
12,4	10,5	0,6	2	2	17	2014
13	12,1	0,6	2	2	20	2000
13	12,1	0,6	3	2	21	2005
13	12,1	0,6	2	2	23	2006
13	12,1	0,6	3	2	23	2010
13	12,1	0,6	3	2	23	2010
16,4	24,3	0,6	4	2	18	2001
14,7	17,5	0,6	2	2	21	2001
14,7	17,5	0,6	3	2	25	2004
14,7	17,5	0,6	2	2	23	2010
14,7	17,5	0,6	2	2	23	2010
17,9	31,6	0,6	4	2	18	2015
13,9	14,8	0,6	2	2	21	2006
13,9	14,8	0,6	2	2	19	2013
15	18,6	0,6	3	2	23	2001
15	18,6	0,6	4	2	19	2009
16,1	23	0,6	8	2	23	2015
13,1	12,4	0,6	3	2	21	2008
17	27,1	0,6	6	2	19	2004
12,7	11,3	0,6	3	2	16	2015
13,8	14,5	0,6	2	2	20	2001
13,8	14,5	0,6	2	2	23	2001

14,2	15,8	0,6	4	2	20	2001
14,2	15,8	0,6	5	2	22	2007
14,2	15,8	0,6	3	2	19	2010
14,2	15,8	0,6	3	2	19	2010
14,2	15,8	0,6	7	2	21	2014
14,2	15,8	0,6	7	2	23	2014
15,1	19	0,6	3	2	20	2010
15,1	19	0,6	3	2	20	2010
15,1	19	0,6	4	2	15	2015
14,8	17,9	0,6	2	2	23	2001
14,8	17,9	0,6	3	2	22	2007
14,8	17,9	0,6	3	2	19	2010
14,8	17,9	0,6	3	2	19	2010
14,8	17,9	0,6	3	2	18	2015
13,2	12,7	0,6	2	2	22	2009
13,2	12,7	0,6	4	2	23	2009
13,7	14,2	0,6	3	2	23	2010
13,7	14,2	0,6	2	2	23	2010
13,7	14,2	0,6	3	2	23	2010
13,7	14,2	0,6	2	2	23	2010
15,9	22,2	0,6	4	2	20	2011
19	37,9	0,6	5	2	19	2013
13,3	13	0,6	2	2	23	2003
13,6	13,9	0,6	3	2	22	2005
13,6	13,9	0,6	2	2	20	2010
13,6	13,9	0,6	2	2	20	2010
14,6	17,2	0,6	3	2	19	1999
16,2	23,5	0,6	5	2	17	2009

13,4	13,3	0,6	3	2	25	2004
13,4	13,3	0,6	3	2	26	2009
13,5	13,6	0,6	2	2	20	2000
14,1	15,5	0,6	4	2	20	2008
14,1	15,5	0,6	3	2	20	2012
16,6	25,3	0,6	4	2	19	2015
16,4	24,4	0,6	5	2	20	1999
12,1	9,8	0,6	2	2	20	2000
12,1	9,8	0,6	3	2	21	2005
12,1	9,8	0,6	2	2	26	2006
15,5	20,6	0,6	4	2	19	2015
14,9	18,3	0,6	4	2	20	2008
12,3	10,3	0,6	2	2	26	2006
12,3	10,3	0,6	2	2	26	2006
16,1	23,1	0,6	6	2	19	1999
14	15,2	0,6	3	2	23	2010
14	15,2	0,6	3	2	23	2010
14	15,2	0,6	3	2	23	2011
14	15,2	0,6	4	2	19	2013
14,3	16,2	0,6	4	2	23	2010
14,3	16,2	0,6	4	2	23	2010
14,3	16,2	0,6	3	2	18	2015
15	18,7	0,6	4	2	19	2004
12,9	11,9	0,6	2	2	17	2002
14,5	16,9	0,6	4	2	20	1999
14,5	16,9	0,6	2	2	18	2002
15,1	19,1	0,6	3	2	17	2014
13,9	14,9	0,6	3	2	17	2014

12,6	11,1	0,6	2	2	20	2013
15,8	21,9	0,6	5	2	26	2006
15,8	21,9	0,6	5	2	18	2007
14,8	18	0,6	4	2	23	1999
14,8	18	0,6	2	2	17	2009
15,2	19,5	0,6	6	2	19	2012
13	12,2	0,6	3	2	22	2005
13	12,2	0,6	3	2	19	2007
13	12,2	0,6	2	2	19	2013
13	12,2	0,6	2	2	19	2013
16,4	24,5	0,6	3	2	25	2003
13,8	14,6	0,6	4	2	19	2009
13,8	14,6	0,6	2	2	23	2010
13,8	14,6	0,6	2	2	23	2010
13,8	14,6	0,6	3	2	19	2013
12	9,6	0,6	2	2	26	2006
12	9,6	0,6	2	2	18	2007
12	9,6	0,6	2	2	20	2007
15,3	19,9	0,6	3	2	22	2003
15,3	19,9	0,6	4	2	26	2003
15,4	20,3	0,6	3	2	23	2003
15,5	20,7	0,6	4	2	19	2008
14,6	17,3	0,6	5	2	18	2007
14,4	16,6	0,6	2	2	18	2002
14,4	16,6	0,6	4	2	25	2004
13,1	12,5	0,6	2	2	17	2008
13,1	12,5	0,6	2	2	19	2013
16,7	25,9	0,6	5	2	20	2009

13,7	14,3	0,6	3	2	25	2004
17,4	29,3	0,6	9	2	20	2004
12,2	10,1	0,6	2	2	19	2002
16,3	24,1	0,6	7	2	21	2014
14,1	15,6	0,6	2	2	17	2014
13,2	12,8	0,6	2	2	21	2009
12,7	11,4	0,6	2	2	20	2013
13,6	14	0,6	3	2	19	2013
13,3	13,1	0,6	3	2	25	2004
13,3	13,1	0,6	3	2	22	2005
13,5	13,7	0,6	2	2	20	2002
13,5	13,7	0,6	4	2	22	2011
13,4	13,4	0,6	2	2	19	2006
13,4	13,4	0,6	3	2	19	2015
15	18,8	0,6	4	2	18	2015
14,7	17,7	0,6	5	2	19	1999
14,7	17,7	0,6	3	2	21	2001
14,7	17,7	0,6	3	2	20	2003
14,7	17,7	0,6	4	2	18	2014
16,9	26,9	0,6	4	2	16	2008
14,3	16,3	0,6	2	2	21	2003
16,2	23,7	0,6	6	2	20	2011
16,2	23,7	0,6	7	2	21	2014
16,6	25,5	0,6	5	2	19	1999
14	15,3	0,6	2	2	21	2001
14	15,3	0,6	4	2	23	2009
14	15,3	0,6	7	2	26	2009
14	15,3	0,6	3	2	20	2010

14	15,3	0,6	3	2	20	2010
14	15,3	0,6	2	2	19	2012
14	15,3	0,6	3	2	23	2015
14,5	17	0,6	3	2	19	1999
14,5	17	0,6	4	2	21	2009
14,5	17	0,6	4	2	18	2015
15,1	19,2	0,6	4	2	21	2011
15,1	19,2	0,6	4	2	19	2012
15,1	19,2	0,6	3	2	17	2014
15,8	22	0,6	3	2	18	2000
15,8	22	0,6	3	2	19	2011
12,8	11,7	0,6	2	2	23	2007
15,2	19,6	0,6	3	2	18	2001
15,2	19,6	0,6	3	2	23	2001
15,2	19,6	0,6	10	2	26	2006
15,2	19,6	0,6	3	2	21	2010
15,2	19,6	0,6	3	2	21	2010
10,3	6,1	0,6	2	2	23	2004
13,9	15	0,6	3	2	20	2008
13,9	15	0,6	2	2	19	2014
17,8	31,5	0,6	3	2	21	2015
15,5	20,8	0,6	3	2	17	2014
18,4	34,8	0,6	4	2	16	2015
14,2	16	0,6	3	2	23	2010
14,2	16	0,6	3	2	23	2010
11,5	8,5	0,6	2	2	20	2004
12,9	12	0,6	2	2	18	2014
18,2	33,7	0,6	10	2	23	2010

18,2	33,7	0,6	10	2	23	2010
16	22,9	0,6	6	2	20	2004
14,6	17,4	0,6	2	2	19	2014
14,9	18,5	0,6	5	2	19	2012
14,9	18,5	0,6	4	2	17	2014
14,4	16,7	0,6	4	2	22	2011
13,8	14,7	0,6	2	2	19	2013
13,8	14,7	0,6	3	2	18	2014
17	27,5	0,6	8	2	23	2008
15,9	22,5	0,6	3	2	17	2012
16,2	23,8	0,6	3	2	21	2001
13	12,3	0,6	2	2	21	2000
13	12,3	0,6	3	2	18	2008
13	12,3	0,6	2	2	22	2009
15	18,9	0,6	4	2	22	2004
15	18,9	0,6	3	2	20	2007
13,7	14,4	0,6	3	2	18	2014
14,1	15,7	0,6	3	2	19	2002
15,8	22,1	0,6	3	2	21	2001
15,8	22,1	0,6	7	2	26	2005
15,8	22,1	0,6	5	2	19	2010
15,8	22,1	0,6	5	2	19	2010
15,8	22,1	0,6	4	2	17	2014
14,7	17,8	0,6	6	2	22	2003
14,7	17,8	0,6	4	2	19	2015
16,7	26,1	0,6	5	2	18	2000
16,7	26,1	0,6	5	2	21	2010
16,7	26,1	0,6	5	2	21	2010

16,7	26,1	0,6	8	2	22	2015
13,1	12,6	0,6	2	2	18	2008
13,1	12,6	0,6	2	2	23	2009
13,1	12,6	0,6	2	2	20	2013
13,1	12,6	0,6	2	2	20	2013
15,1	19,3	0,6	4	2	21	2000
15,7	21,7	0,6	7	2	22	2009
15,7	21,7	0,6	4	2	22	2011
14,3	16,4	0,6	2	2	23	2001
14,3	16,4	0,6	4	2	21	2009
14,3	16,4	0,6	3	2	19	2015
13,2	12,9	0,6	2	2	20	2013
14,5	17,1	0,6	5	2	25	2004
14,5	17,1	0,6	6	2	18	2008
15,2	19,7	0,6	6	2	20	2008
13,4	13,5	0,6	4	2	21	2000
13,4	13,5	0,6	2	2	19	2013
16,3	24,3	0,6	5	2	18	1999
16,3	24,3	0,6	5	2	21	2010
16,3	24,3	0,6	5	2	21	2010
12,4	10,7	0,6	2	2	23	2006
15,3	20,1	0,6	4	2	19	1999
15,3	20,1	0,6	7	2	19	2012
14	15,4	0,6	4	2	19	2009
14	15,4	0,6	2	2	18	2014
14	15,4	0,6	3	2	19	2014
15,5	20,9	0,6	6	2	20	2013
15,4	20,5	0,6	5	2	19	2001

15,4	20,5	0,6	3	2	15	2015
11,3	8,1	0,6	2	2	20	2004
14,8	18,2	0,6	3	2	23	2001
14,8	18,2	0,6	2	2	26	2003
14,8	18,2	0,6	3	2	25	2004
12,7	11,5	0,6	3	2	23	2010
12,7	11,5	0,6	3	2	23	2010
12,7	11,5	0,6	2	2	21	2014
12,2	10,2	0,6	2	2	21	2005
12,2	10,2	0,6	2	2	20	2008
11,7	9	0,6	2	2	20	2002
16,2	23,9	0,6	4	2	23	2001
13,9	15,1	0,6	2	2	23	2010
13,9	15,1	0,6	2	2	23	2010
14,6	17,5	0,6	3	2	19	2012
14,4	16,8	0,6	3	2	22	2001
15	19	0,6	3	2	20	2008
15	19	0,6	5	2	21	2011
15	19	0,6	4	2	23	2011
15	19	0,6	3	2	19	2013
15	19	0,6	3	2	19	2013
13,8	14,8	0,6	2	2	18	2000
13,8	14,8	0,6	5	2	18	2007
13,8	14,8	0,6	3	2	20	2010
13,8	14,8	0,6	2	2	23	2010
13,8	14,8	0,6	3	2	20	2010
13,8	14,8	0,6	2	2	23	2010
13,8	14,8	0,6	2	2	19	2013

12,5	11	0,6	2	2	20	2004
12,5	11	0,6	2	2	19	2013
16,5	25,3	0,6	5	2	16	2015
15,7	21,8	0,6	5	2	19	1999
15,7	21,8	0,6	3	2	18	2001
16,3	24,4	0,6	3	2	20	2003
15,1	19,4	0,6	3	2	17	2008
15,1	19,4	0,6	6	2	19	2008
15,1	19,4	0,6	9	2	22	2009
14,7	17,9	0,6	3	2	21	2003
14,7	17,9	0,6	4	2	21	2009
14,7	17,9	0,6	5	2	21	2010
14,7	17,9	0,6	5	2	21	2010
14,1	15,8	0,6	3	2	20	2008
14,1	15,8	0,6	3	2	19	2015
12,9	12,1	0,6	2	2	21	2006
12,9	12,1	0,6	2	2	21	2008
15,6	21,4	0,6	4	2	23	2009
11,9	9,5	0,6	2	2	20	2004
11,9	9,5	0,6	2	2	18	2007
11,6	8,8	0,6	2	2	20	2004
15,2	19,8	0,6	3	2	21	2001
15,2	19,8	0,6	3	2	25	2003
15,2	19,8	0,6	4	2	17	2009
13,7	14,5	0,6	3	2	15	2015
16	23,1	0,6	6	2	22	2007
17,1	28,2	0,6	6	2	17	2002
17,1	28,2	0,6	5	2	19	2010

17,1	28,2	0,6	5	2	19	2010
17,1	28,2	0,6	5	2	20	2012
15,3	20,2	0,6	9	2	22	2007
15,3	20,2	0,6	4	2	20	2011
15,3	20,2	0,6	5	2	19	2012
15,4	20,6	0,6	4	2	18	2015
14,5	17,2	0,6	3	2	26	2001
14,5	17,2	0,6	4	2	22	2011
12,3	10,5	0,6	2	2	22	2005
14,3	16,5	0,6	3	2	18	2014
13	12,4	0,6	3	2	20	2011
13	12,4	0,6	2	2	19	2013
12,1	10	0,6	2	2	25	2004
12,1	10	0,6	2	2	18	2007
12,1	10	0,6	2	2	20	2007
16,4	24,9	0,6	4	2	18	2001
14	15,5	0,6	3	2	18	2000
14	15,5	0,6	3	2	19	2010
14	15,5	0,6	3	2	19	2010
14	15,5	0,6	3	2	20	2013
12,6	11,3	0,6	2	2	20	2010
12,6	11,3	0,6	2	2	20	2010
13,1	12,7	0,6	3	2	18	2014
16,8	26,8	0,6	7	2	25	2004
13,4	13,6	0,6	3	2	18	2007
14,9	18,7	0,6	4	2	21	2005
14,9	18,7	0,6	5	2	18	2007
13,3	13,3	0,6	2	2	18	2002

13,3	13,3	0,6	2	2	19	2010
13,3	13,3	0,6	2	2	19	2010
15,8	22,3	0,6	3	2	19	2004
16,5	25,4	0,6	5	2	19	2010
16,5	25,4	0,6	5	2	19	2010
16,1	23,6	0,6	7	2	20	2012
14,6	17,6	0,6	4	2	23	2003
16,3	24,5	0,6	5	2	18	2001
16,3	24,5	0,6	6	2	21	2001
14,2	16,2	0,6	4	2	23	2010
14,2	16,2	0,6	4	2	23	2010
15,7	21,9	0,6	4	2	20	2001
15	19,1	0,6	3	2	17	2002
15	19,1	0,6	6	2	25	2004
15	19,1	0,6	3	2	25	2004
15	19,1	0,6	3	2	20	2007
15	19,1	0,6	5	2	22	2007
15	19,1	0,6	2	2	22	2013
15	19,1	0,6	3	2	18	2014
13,9	15,2	0,6	3	2	22	2015
16,6	25,9	0,6	3	2	20	2003
15,6	21,5	0,6	3	2	18	2001
15,1	19,5	0,6	3	2	20	2003
15,1	19,5	0,6	4	2	21	2006
15,1	19,5	0,6	3	2	23	2010
15,1	19,5	0,6	3	2	23	2010
16	23,2	0,6	3	2	18	2001
16	23,2	0,6	6	2	20	2011

12,4	10,8	0,6	2	2	24	2009
14,7	18	0,6	4	2	19	1999
14,7	18	0,6	3	2	18	2000
14,7	18	0,6	4	2	26	2006
14,7	18	0,6	3	2	18	2014
14,7	18	0,6	5	2	15	2015
15,2	19,9	0,6	4	2	18	2008
15,2	19,9	0,6	3	2	19	2014
17,9	32,5	0,6	3	2	19	2013
15,4	20,7	0,6	3	2	18	2015
16,4	25	0,6	5	2	18	2012
15,3	20,3	0,6	5	2	15	2015
15,3	20,3	0,6	3	2	18	2015
13,8	14,9	0,6	2	2	23	2008
12	9,8	0,6	2	2	20	2004
12	9,8	0,6	2	2	22	2011
15,9	22,8	0,6	4	2	17	2009
12,8	11,9	0,6	2	2	24	2006
14,5	17,3	0,6	7	2	26	2009
14,3	16,6	0,6	2	2	19	2000
14,3	16,6	0,6	5	2	23	2006
14,3	16,6	0,6	3	2	16	2015
15,8	22,4	0,6	5	2	20	2001
15,8	22,4	0,6	3	2	17	2014
16,3	24,6	0,6	3	2	20	2003
12,9	12,2	0,6	2	2	22	2007
12,5	11,1	0,6	2	2	26	2006
12,5	11,1	0,6	2	2	20	2009

18,1	33,7	0,6	4	2	18	2014
14,9	18,8	0,6	4	2	26	2006
14,9	18,8	0,6	3	2	19	2014
13,6	14,3	0,6	2	2	18	2014
13,6	14,3	0,6	3	2	23	2015
14	15,6	0,6	2	2	18	2002
14	15,6	0,6	4	2	20	2006
14	15,6	0,6	3	2	20	2008
14	15,6	0,6	4	2	22	2009
14	15,6	0,6	3	2	19	2013
14	15,6	0,6	3	2	19	2014
18,5	36	0,6	6	2	20	2010
18,5	36	0,6	6	2	20	2010
18,5	36	0,6	5	2	18	2014
14,6	17,7	0,6	3	2	20	2010
14,6	17,7	0,6	3	2	20	2010
14,6	17,7	0,6	4	2	19	2014
16	23,3	0,6	5	2	25	2002
16	23,3	0,6	8	2	20	2010
16	23,3	0,6	8	2	20	2010
18,7	37,2	0,6	4	2	15	2015
15	19,2	0,6	3	2	20	2007
13	12,5	0,6	2	2	20	2008
13	12,5	0,6	2	2	23	2010
13	12,5	0,6	2	2	23	2010
13	12,5	0,6	2	2	19	2013
13	12,5	0,6	2	2	19	2013
13	12,5	0,6	2	2	19	2014

13,5	14	0,6	2	2	21	2000
13,5	14	0,6	3	2	23	2008
16,2	24,2	0,6	3	2	19	2010
16,2	24,2	0,6	3	2	19	2010
14,2	16,3	0,6	2	2	20	2001
14,2	16,3	0,6	2	2	23	2006
14,2	16,3	0,6	3	2	23	2008
14,2	16,3	0,6	4	2	20	2011
14,4	17	0,6	3	2	21	1999
14,4	17	0,6	3	2	19	2001
14,4	17	0,6	4	2	22	2003
14,4	17	0,6	4	2	20	2011
13,1	12,8	0,6	3	2	22	2005
13,1	12,8	0,6	2	2	19	2013
13,4	13,7	0,6	2	2	19	2002
13,4	13,7	0,6	2	2	21	2003
13,4	13,7	0,6	2	2	18	2014
11,2	8	0,6	2	2	19	2004
15,2	20	0,6	5	2	21	2010
15,2	20	0,6	5	2	21	2010
15,4	20,8	0,6	3	2	21	2003
13,2	13,1	0,6	2	2	20	2001
13,2	13,1	0,6	2	2	17	2002
15,3	20,4	0,6	3	2	23	2003
15,3	20,4	0,6	4	2	25	2003
15,3	20,4	0,6	3	2	26	2003
15,3	20,4	0,6	4	2	23	2006
12,3	10,6	0,6	2	2	18	2007

13,9	15,3	0,6	4	2	19	2009
13,9	15,3	0,6	2	2	20	2012
14,7	18,1	0,6	3	2	19	2002
14,7	18,1	0,6	3	2	21	2015
17	28	0,6	5	2	17	2014
12,1	10,1	0,6	2	2	19	2006
12,1	10,1	0,6	2	2	18	2007
16,1	23,8	0,6	5	2	20	2007
16,1	23,8	0,6	5	2	20	2012
15,8	22,5	0,6	3	2	22	2003
14,5	17,4	0,6	3	2	25	2004
14,5	17,4	0,6	4	2	26	2009
13,8	15	0,6	4	2	23	1999
13,8	15	0,6	3	2	18	2008
13,8	15	0,6	3	2	22	2011
13,8	15	0,6	2	2	21	2013
14,1	16	0,6	4	2	23	2009
15,7	22,1	0,6	5	2	21	2010
15,7	22,1	0,6	5	2	21	2010
14,3	16,7	0,6	3	2	19	2000
14,3	16,7	0,6	3	2	23	2010
14,3	16,7	0,6	3	2	23	2010
14,3	16,7	0,6	4	2	20	2011
14,3	16,7	0,6	4	2	17	2012
14,3	16,7	0,6	4	2	16	2015
12,7	11,7	0,6	2	2	18	2002
12,7	11,7	0,6	2	2	22	2005
16	23,4	0,6	4	2	25	2004

16	23,4	0,6	5	2	19	2006
16	23,4	0,6	9	2	21	2011
16,4	25,2	0,6	5	2	19	2002
14,9	18,9	0,6	3	2	20	2006
14,9	18,9	0,6	4	2	26	2006
16,8	27,1	0,6	6	2	19	2004
13,7	14,7	0,6	4	2	25	2004
13,7	14,7	0,6	2	2	19	2010
13,7	14,7	0,6	2	2	19	2010
13,7	14,7	0,6	3	2	19	2014
13,7	14,7	0,6	2	2	19	2015
12,4	10,9	0,6	2	2	22	2005
15	19,3	0,6	4	2	23	2011
15	19,3	0,6	3	2	19	2014
15	19,3	0,6	5	2	21	2014
15	19,3	0,6	3	2	15	2015
14,6	17,8	0,6	3	2	19	2001
14,6	17,8	0,6	2	2	21	2001
14,6	17,8	0,6	4	2	21	2005
14	15,7	0,6	2	2	23	2006
14	15,7	0,6	3	2	16	2008
14	15,7	0,6	8	2	23	2010
14	15,7	0,6	8	2	23	2010
14	15,7	0,6	3	2	17	2014
15,1	19,7	0,6	3	2	21	2001
15,9	23	0,6	3	2	21	2001
15,9	23	0,6	3	2	19	2004
15,9	23	0,6	9	2	19	2014

12,8	12	0,6	4	2	26	2006
15,4	20,9	0,6	3	2	19	2004
15,4	20,9	0,6	6	2	22	2011
13,6	14,4	0,6	2	2	26	2006
13,6	14,4	0,6	2	2	26	2006
13,6	14,4	0,6	2	2	22	2011
13,6	14,4	0,6	2	2	19	2013
13,6	14,4	0,6	3	2	19	2015
14,4	17,1	0,6	2	2	21	2003
14,4	17,1	0,6	4	2	22	2011
12,2	10,4	0,6	2	2	22	2013
14,2	16,4	0,6	3	2	19	2001
14,2	16,4	0,6	2	2	19	2014
12,9	12,3	0,6	2	2	19	2002
13,5	14,1	0,6	2	2	19	2014
13,9	15,4	0,6	5	2	23	2007
13	12,6	0,6	2	2	19	2002
13	12,6	0,6	3	2	23	2010
13	12,6	0,6	3	2	23	2010
13,4	13,8	0,6	2	2	18	2014
16,8	27,2	0,6	6	2	22	2005
15,7	22,2	0,6	4	2	19	1999
15,7	22,2	0,6	3	2	19	2010
15,7	22,2	0,6	3	2	19	2010
16	23,5	0,6	3	2	19	2012
14,8	18,6	0,6	3	2	21	2000
14,8	18,6	0,6	4	2	17	2009
14,8	18,6	0,6	5	2	23	2010

14,8	18,6	0,6	5	2	23	2010
14,8	18,6	0,6	3	2	15	2015
13,1	12,9	0,6	2	2	19	2002
13,1	12,9	0,6	2	2	19	2013
13,1	12,9	0,6	2	2	21	2014
13,1	12,9	0,6	2	2	23	2014
16,2	24,4	0,6	4	2	17	2009
13,2	13,2	0,6	2	2	18	2002
13,2	13,2	0,6	2	2	23	2010
13,2	13,2	0,6	2	2	23	2010
17,1	28,7	0,6	6	2	20	2013
14,5	17,5	0,6	3	2	19	2010
14,5	17,5	0,6	3	2	19	2010
21,2	54,7	0,6	10	2	16	2015
16,5	25,8	0,6	5	2	21	2000
14,9	19	0,6	4	2	20	2004
14,9	19	0,6	4	2	17	2009
11,7	9,2	0,6	2	2	22	2007
14,3	16,8	0,6	3	2	18	2000
14,3	16,8	0,6	3	2	17	2014
13,8	15,1	0,6	2	2	19	2000
13,8	15,1	0,6	2	2	18	2001
13,8	15,1	0,6	2	2	19	2010
13,8	15,1	0,6	2	2	19	2010
15,5	21,4	0,6	5	2	19	2010
15,5	21,4	0,6	5	2	19	2010
18,4	35,8	0,6	4	2	21	2012
15	19,4	0,6	8	2	20	2010

15	19,4	0,6	5	2	21	2010
15	19,4	0,6	8	2	20	2010
15	19,4	0,6	5	2	21	2010
15	19,4	0,6	6	2	23	2011
18,5	36,4	0,6	6	2	19	2013
16,3	24,9	0,6	5	2	19	2010
16,3	24,9	0,6	5	2	19	2010
15,4	21	0,6	6	2	17	2009
15,1	19,8	0,6	4	2	21	2009
15,1	19,8	0,6	6	2	22	2011
15,1	19,8	0,6	5	2	21	2014
15,3	20,6	0,6	3	2	26	2003
15,3	20,6	0,6	6	2	20	2010
15,3	20,6	0,6	6	2	20	2010
15,3	20,6	0,6	3	2	16	2015
14,6	17,9	0,6	4	2	19	1999
14,6	17,9	0,6	2	2	21	2003
15,2	20,2	0,6	5	2	21	2000
15,2	20,2	0,6	4	2	23	2009
15,2	20,2	0,6	3	2	19	2015
11,3	8,3	0,6	2	2	20	2004
15,8	22,7	0,6	5	2	20	2006
15,8	22,7	0,6	3	2	19	2013
13,7	14,8	0,6	4	2	25	2004
16,8	27,3	0,6	7	2	23	2001
14	15,8	0,6	3	2	18	2000
14	15,8	0,6	2	2	18	2015
17,2	29,3	0,6	6	2	18	2014

16,4	25,4	0,6	4	2	15	2015
19,8	44,7	0,6	6	2	17	2014
16,9	27,8	0,6	6	2	18	2001
17,6	31,4	0,6	6	2	23	2015
17	28,3	0,6	7	2	23	2001
14,4	17,2	0,6	5	2	22	2009
12,7	11,8	0,6	2	2	20	2003
12,7	11,8	0,6	2	2	22	2009
14,7	18,3	0,6	3	2	25	2003
14,7	18,3	0,6	3	2	22	2009
14,7	18,3	0,6	7	2	26	2009
14,7	18,3	0,6	5	2	23	2010
14,7	18,3	0,6	5	2	23	2010
14,7	18,3	0,6	3	2	18	2015
16	23,6	0,6	4	2	18	2015
15,7	22,3	0,6	6	2	19	2000
14,2	16,5	0,6	3	2	25	2004
14,2	16,5	0,6	3	2	18	2007
14,2	16,5	0,6	5	2	23	2010
14,2	16,5	0,6	5	2	23	2010
16,2	24,5	0,6	3	2	19	2010
16,2	24,5	0,6	3	2	19	2010
18,4	35,9	0,6	4	2	15	2015
13,6	14,5	0,6	2	2	23	2009
11,6	9	0,6	2	2	18	2007
21	53,4	0,6	9	2	17	2014
14,8	18,7	0,6	3	2	22	2000
14,8	18,7	0,6	4	2	25	2004

14,8	18,7	0,6	4	2	23	2008
14,8	18,7	0,6	4	2	20	2009
14,8	18,7	0,6	3	2	20	2013
14,8	18,7	0,6	3	2	17	2014
15,6	21,9	0,6	5	2	19	1999
15,6	21,9	0,6	3	2	21	2000
12,4	11	0,6	2	2	26	2006
12,4	11	0,6	2	2	26	2006
12,8	12,1	0,6	2	2	23	2009
12,8	12,1	0,6	2	2	20	2010
12,8	12,1	0,6	2	2	20	2010
12,8	12,1	0,6	2	2	18	2014
17,4	30,4	0,6	8	2	23	2001
13,5	14,2	0,6	2	2	20	2000
13,5	14,2	0,6	4	2	23	2008
13,5	14,2	0,6	2	2	21	2010
13,5	14,2	0,6	2	2	21	2010
13,5	14,2	0,6	2	2	18	2014
13,9	15,5	0,6	3	2	25	2002
13,9	15,5	0,6	3	2	25	2007
13,9	15,5	0,6	3	2	18	2015
15,9	23,2	0,6	6	2	19	2008
16,3	25	0,6	10	2	23	2010
16,3	25	0,6	10	2	23	2010
16,3	25	0,6	3	2	18	2015
16,3	25	0,6	8	2	23	2015
14,5	17,6	0,6	3	2	18	2000
15,5	21,5	0,6	7	2	20	2009

15,5	21,5	0,6	3	2	17	2014
14,9	19,1	0,6	4	2	19	2012
13,4	13,9	0,6	2	2	18	1999
15	19,5	0,6	4	2	21	2009
15	19,5	0,6	4	2	15	2015
15	19,5	0,6	4	2	16	2015
10,4	6,5	0,6	2	2	23	2004
16,8	27,4	0,6	6	2	21	2011
14,1	16,2	0,6	2	2	20	2001
14,1	16,2	0,6	3	2	20	2008
14,1	16,2	0,6	2	2	20	2010
14,1	16,2	0,6	3	2	21	2010
14,1	16,2	0,6	2	2	20	2010
14,1	16,2	0,6	3	2	21	2010
14,1	16,2	0,6	3	2	21	2015
14,3	16,9	0,6	2	2	18	1999
14,3	16,9	0,6	4	2	18	2000
14,3	16,9	0,6	4	2	22	2005
14,3	16,9	0,6	6	2	23	2006
14,3	16,9	0,6	3	2	19	2008
14,3	16,9	0,6	3	2	21	2008
14,3	16,9	0,6	4	2	23	2011
14,3	16,9	0,6	3	2	19	2013
15,3	20,7	0,6	4	2	19	1999
15,3	20,7	0,6	4	2	19	2004
15,3	20,7	0,6	4	2	18	2015
15,1	19,9	0,6	6	2	23	2006
15,1	19,9	0,6	4	2	19	2013

15,8	22,8	0,6	5	2	25	2004
15,8	22,8	0,6	6	2	25	2004
15,2	20,3	0,6	3	2	22	2001
17	28,4	0,6	6	2	20	2013
17	28,4	0,6	6	2	21	2014
17	28,4	0,6	5	2	21	2015
13	12,7	0,6	2	2	18	2014
13,3	13,6	0,6	2	2	20	2002
13,3	13,6	0,6	3	2	23	2011
13,3	13,6	0,6	2	2	20	2013
13,3	13,6	0,6	3	2	21	2014
16,4	25,5	0,6	6	2	22	2004
16,4	25,5	0,6	5	2	22	2005
13,1	13	0,6	3	2	20	2007
13,1	13	0,6	2	2	21	2009
13,1	13	0,6	3	2	21	2011
13,2	13,3	0,6	2	2	19	2014
13,8	15,2	0,6	2	2	20	2001
13,8	15,2	0,6	3	2	23	2008
14,6	18	0,6	2	2	24	2001
14,6	18	0,6	2	2	20	2003
14,6	18	0,6	3	2	26	2003
14,6	18	0,6	3	2	19	2013
14,6	18	0,6	3	2	18	2014
12,5	11,3	0,6	2	2	26	2006
16	23,7	0,6	5	2	19	2002
11,5	8,8	0,6	2	2	20	2004
11,5	8,8	0,6	2	2	20	2004

15,7	22,4	0,6	3	2	21	2003
15,7	22,4	0,6	3	2	16	2015
17,4	30,5	0,6	7	2	23	2015
14,7	18,4	0,6	3	2	23	2010
14,7	18,4	0,6	3	2	23	2010
14,7	18,4	0,6	4	2	15	2015
14,4	17,3	0,6	3	2	18	2008
14	15,9	0,6	4	2	22	2007
13,7	14,9	0,6	3	2	20	2008
13,7	14,9	0,6	3	2	23	2015
15,9	23,3	0,6	4	2	20	2001
16,7	27	0,6	4	2	18	2015
14,2	16,6	0,6	4	2	25	2008
14,2	16,6	0,6	5	2	20	2010
14,2	16,6	0,6	5	2	20	2010
12,6	11,6	0,6	2	2	23	2005
14,8	18,8	0,6	2	2	23	2003
14,8	18,8	0,6	4	2	23	2006
14,8	18,8	0,6	5	2	17	2009
14,8	18,8	0,6	3	2	19	2015
17,1	29	0,6	10	2	17	2009
15,5	21,6	0,6	4	2	21	2003
15,5	21,6	0,6	4	2	19	2011
17	28,5	0,6	7	2	19	2014
12,3	10,8	0,6	2	2	21	2006
16,4	25,6	0,6	3	2	18	2001
22	61,8	0,6	10	2	16	2015
14,9	19,2	0,6	4	2	21	2008

18,2	35	0,6	5	2	18	2015
14,5	17,7	0,6	3	2	24	2001
14,5	17,7	0,6	2	2	22	2003
14,5	17,7	0,6	5	2	23	2007
11,7	9,3	0,6	1	2	26	2001
11,7	9,3	0,6	2	2	23	2004
11,7	9,3	0,6	2	2	20	2007
15,3	20,8	0,6	3	2	22	2003
15,1	20	0,6	4	2	25	2004
15,1	20	0,6	3	2	19	2012
15,2	20,4	0,6	3	2	20	2003
12,7	11,9	0,6	2	2	20	2002
12,7	11,9	0,6	2	2	19	2013
12,7	11,9	0,6	2	2	20	2013
16,2	24,7	0,6	3	2	22	2003
16,2	24,7	0,6	5	2	19	2010
16,2	24,7	0,6	5	2	19	2010
16,5	26,1	0,6	3	2	18	2000
13,5	14,3	0,6	3	2	23	2010
13,5	14,3	0,6	3	2	23	2010
14,3	17	0,6	2	2	20	2001
14,3	17	0,6	4	2	23	2008
14,3	17	0,6	7	2	19	2009
14,3	17	0,6	5	2	23	2010
14,3	17	0,6	5	2	23	2010
12,1	10,3	0,6	2	2	20	2013
14,1	16,3	0,6	3	2	21	2000
14,1	16,3	0,6	3	2	20	2008

14,1	16,3	0,6	4	2	19	2009
14,1	16,3	0,6	2	2	22	2009
14,1	16,3	0,6	3	2	18	2015
14,6	18,1	0,6	3	2	18	2000
12,8	12,2	0,6	2	2	26	2006
13,4	14	0,6	2	2	22	2009
16,7	27,1	0,6	5	2	22	2007
17,1	29,1	0,6	7	2	19	2014
15,6	22,1	0,6	4	2	22	2011
15,6	22,1	0,6	3	2	16	2015
17	28,6	0,6	7	2	17	2014
11,3	8,4	0,6	2	2	18	2007
16,9	28,1	0,6	7	2	17	2014
13,8	15,3	0,6	2	2	17	2002
13,8	15,3	0,6	2	2	22	2003
13,8	15,3	0,6	3	2	18	2015
16,1	24,3	0,6	6	2	17	2008
12,9	12,5	0,6	3	2	21	2005
12,9	12,5	0,6	2	2	22	2013
13,3	13,7	0,6	3	2	19	2008
13,3	13,7	0,6	2	2	21	2008
13,3	13,7	0,6	2	2	26	2009
13,3	13,7	0,6	3	2	15	2015
14,7	18,5	0,6	2	2	22	2003
13	12,8	0,6	2	2	25	2003
13	12,8	0,6	3	2	23	2005
13	12,8	0,6	3	2	20	2007
13	12,8	0,6	5	2	20	2007

19,5	43,2	0,6	5	2	21	2010
19,5	43,2	0,6	5	2	21	2010
13,2	13,4	0,6	2	2	21	2001
13,2	13,4	0,6	3	2	19	2007
13,2	13,4	0,6	3	2	19	2010
13,2	13,4	0,6	3	2	19	2010
13,2	13,4	0,6	2	2	19	2013
16,4	25,7	0,6	6	2	22	2001
16,4	25,7	0,6	3	2	20	2003
16,4	25,7	0,6	5	2	19	2010
16,4	25,7	0,6	5	2	19	2010
13,1	13,1	0,6	2	2	19	2013
13,1	13,1	0,6	2	2	19	2013
14,4	17,4	0,6	3	2	19	2002
14,4	17,4	0,6	2	2	22	2003
14,4	17,4	0,6	4	2	15	2015
14,4	17,4	0,6	3	2	18	2015
11,6	9,1	0,6	2	2	20	2004
14	16	0,6	2	2	22	2003
14	16	0,6	4	2	20	2009
14	16	0,6	5	2	22	2009
14	16	0,6	3	2	23	2010
14	16	0,6	3	2	23	2010
14	16	0,6	3	2	19	2012
14	16	0,6	2	2	18	2015
15,8	23	0,6	3	2	18	2001
15,8	23	0,6	3	2	20	2001
15,8	23	0,6	4	2	22	2004

19	40	0,6	8	2	19	2013
14,2	16,7	0,6	2	2	20	2003
14,2	16,7	0,6	4	2	19	2005
13,7	15	0,6	2	2	21	2013
14,9	19,3	0,6	3	2	17	2014
15,3	20,9	0,6	9	2	25	2005
15,3	20,9	0,6	6	2	22	2011
15,3	20,9	0,6	4	2	19	2015
12,5	11,4	0,6	1	2	26	2001
12,5	11,4	0,6	2	2	21	2006
12,5	11,4	0,6	3	2	19	2008
12,5	11,4	0,6	4	2	26	2009
12,5	11,4	0,6	2	2	21	2011
12,5	11,4	0,6	2	2	22	2011
16,6	26,7	0,6	5	2	23	2001
15	19,7	0,6	3	2	19	2008
15	19,7	0,6	4	2	19	2013
15	19,7	0,6	4	2	18	2015
12,2	10,6	0,6	2	2	22	2011
15,1	20,1	0,6	3	2	18	2004
15,1	20,1	0,6	4	2	20	2007
15,1	20,1	0,6	4	2	19	2012
14,5	17,8	0,6	3	2	20	2000
14,5	17,8	0,6	4	2	21	2009
14,5	17,8	0,6	3	2	21	2015
15,7	22,6	0,6	4	2	22	2011
20,7	51,8	0,6	8	2	21	2010
20,7	51,8	0,6	8	2	21	2010

17	28,7	0,6	6	2	19	2013
16,8	27,7	0,6	6	2	22	2001
16,8	27,7	0,6	6	2	17	2008
13,6	14,7	0,6	2	2	18	2000
13,6	14,7	0,6	2	2	23	2009
13,9	15,7	0,6	3	2	23	2008
13,9	15,7	0,6	3	2	21	2014
13,9	15,7	0,6	3	2	18	2015
18	34,1	0,6	5	2	20	2013
15,6	22,2	0,6	5	2	19	2012
15,6	22,2	0,6	7	2	20	2012
14,3	17,1	0,6	2	2	22	2001
14,3	17,1	0,6	4	2	23	2004
14,3	17,1	0,6	3	2	23	2010
14,3	17,1	0,6	3	2	23	2010
14,3	17,1	0,6	5	2	18	2012
14,3	17,1	0,6	3	2	18	2015
12,6	11,7	0,6	2	2	19	2006
12,6	11,7	0,6	2	2	22	2009
14,1	16,4	0,6	2	2	17	2012
14,1	16,4	0,6	3	2	20	2013
14,1	16,4	0,6	3	2	17	2014
14,1	16,4	0,6	3	2	19	2014
13,5	14,4	0,6	2	2	18	2000
13,5	14,4	0,6	2	2	20	2001
13,5	14,4	0,6	2	2	20	2009
13,5	14,4	0,6	2	2	23	2009
13,5	14,4	0,6	2	2	26	2009

13,5	14,4	0,6	3	2	22	2011
13,5	14,4	0,6	2	2	19	2013
15,5	21,8	0,6	8	2	26	2009
16,5	26,3	0,6	4	2	18	2015
14,7	18,6	0,6	5	2	19	1999
14,7	18,6	0,6	4	2	22	2009
12,3	10,9	0,6	2	2	26	2001
12,7	12	0,6	2	2	22	2000
12,7	12	0,6	2	2	19	2002
12,7	12	0,6	2	2	20	2006
12,7	12	0,6	3	2	18	2014
16,6	26,8	0,6	5	2	19	2013
16,6	26,8	0,6	3	2	17	2014
16	24	0,6	6	2	19	2001
16	24	0,6	10	2	18	2015
15,4	21,4	0,6	3	2	15	2015
17,1	29,3	0,6	4	2	19	2013
13,8	15,4	0,6	2	2	20	2002
13,8	15,4	0,6	3	2	17	2014
14,8	19	0,6	3	2	18	2007
14,8	19	0,6	3	2	20	2013
16,7	27,3	0,6	3	2	19	2000
17	28,8	0,6	5	2	18	2000
17	28,8	0,6	6	2	21	2010
17	28,8	0,6	6	2	21	2010
17	28,8	0,6	4	2	19	2013
15,3	21	0,6	4	2	20	2012
15,3	21	0,6	6	2	19	2013

15,3	21	0,6	4	2	19	2015
14,9	19,4	0,6	4	2	17	2009
12,8	12,3	0,6	2	2	17	2002
12,8	12,3	0,6	2	2	18	2014
12,8	12,3	0,6	2	2	21	2014
13,3	13,8	0,6	2	2	22	2005
13,3	13,8	0,6	3	2	18	2008
15,7	22,7	0,6	4	2	25	2004
15,7	22,7	0,6	5	2	19	2010
15,7	22,7	0,6	5	2	19	2010
15,2	20,6	0,6	3	2	25	2003
15	19,8	0,6	4	2	24	2006
15	19,8	0,6	6	2	20	2008
15,1	20,2	0,6	4	2	19	2014
14	16,1	0,6	2	2	18	2000
14	16,1	0,6	3	2	25	2003
14	16,1	0,6	3	2	19	2006
14	16,1	0,6	3	2	20	2008
14	16,1	0,6	4	2	21	2011
14	16,1	0,6	4	2	22	2011
14	16,1	0,6	4	2	19	2012
14,2	16,8	0,6	5	2	22	1999
14,2	16,8	0,6	2	2	22	2003
14,2	16,8	0,6	4	2	20	2004
14,2	16,8	0,6	6	2	18	2008
14,2	16,8	0,6	4	2	23	2011
14,2	16,8	0,6	2	2	19	2013
14,2	16,8	0,6	2	2	20	2013

14,2	16,8	0,6	3	2	18	2014
14,2	16,8	0,6	3	2	21	2014
12,9	12,6	0,6	4	2	26	2006
12,9	12,6	0,6	3	2	18	2007
12,9	12,6	0,6	2	2	23	2007
12,9	12,6	0,6	2	2	18	2014
13,2	13,5	0,6	2	2	19	2001
13,2	13,5	0,6	3	2	20	2010
13,2	13,5	0,6	3	2	20	2010
13,2	13,5	0,6	5	2	21	2011
13,2	13,5	0,6	2	2	19	2013
13,2	13,5	0,6	2	2	19	2013
19,1	40,9	0,6	8	2	18	2015
12,1	10,4	0,6	2	2	22	2007
12,1	10,4	0,6	2	2	22	2015
16,1	24,5	0,6	3	2	20	2001
15,9	23,6	0,6	3	2	20	2003
15,9	23,6	0,6	4	2	19	2007
14,5	17,9	0,6	4	2	20	2011
13	12,9	0,6	3	2	23	2007
13	12,9	0,6	2	2	23	2010
13	12,9	0,6	2	2	23	2010
13	12,9	0,6	2	2	22	2011
13	12,9	0,6	2	2	19	2013
13,1	13,2	0,6	2	2	24	2006
13,1	13,2	0,6	2	2	23	2011
13,7	15,1	0,6	3	2	21	2003
13,7	15,1	0,6	6	2	19	2009

13,7	15,1	0,6	3	2	20	2009
13,7	15,1	0,6	4	2	20	2009
13,7	15,1	0,6	3	2	20	2011
13,7	15,1	0,6	4	2	15	2015
10,5	6,8	0,6	1	2	25	2005
12,4	11,2	0,6	2	2	19	2002
17,2	29,9	0,6	5	2	21	2010
17,2	29,9	0,6	5	2	21	2010
17,2	29,9	0,6	4	2	17	2014
17,5	31,5	0,6	5	2	20	2010
17,5	31,5	0,6	5	2	20	2010
14,6	18,3	0,6	4	2	25	2002
14,6	18,3	0,6	3	2	23	2002
14,6	18,3	0,6	3	2	20	2008
16,2	25	0,6	4	2	19	2013
16,6	26,9	0,6	4	2	21	2014
15,8	23,2	0,6	6	2	16	2008
15,8	23,2	0,6	3	2	19	2010
15,8	23,2	0,6	3	2	19	2010
15,8	23,2	0,6	4	2	18	2015
14,3	17,2	0,6	3	2	23	2010
14,3	17,2	0,6	3	2	23	2010
14,3	17,2	0,6	4	2	20	2011
14,3	17,2	0,6	4	2	19	2011
14,3	17,2	0,6	3	2	18	2014
14,3	17,2	0,6	3	2	16	2015
16,7	27,4	0,6	4	2	18	2001
13,9	15,8	0,6	2	2	26	2003

13,9	15,8	0,6	4	2	21	2008
13,9	15,8	0,6	2	2	19	2014
13,6	14,8	0,6	2	2	22	2003
16	24,1	0,6	4	2	21	2003
16,9	28,4	0,6	5	2	21	2013
14,1	16,5	0,6	3	2	26	2003
14,1	16,5	0,6	3	2	19	2006
14,1	16,5	0,6	5	2	22	2007
14,1	16,5	0,6	5	2	22	2007
14,1	16,5	0,6	3	2	23	2015
15,4	21,5	0,6	4	2	16	2008
15,3	21,1	0,6	3	2	18	2014
15,7	22,8	0,6	4	2	19	2001
14,8	19,1	0,6	4	2	26	2009
14,8	19,1	0,6	4	2	23	2010
14,8	19,1	0,6	4	2	23	2010
13,5	14,5	0,6	2	2	19	2002
13,5	14,5	0,6	3	2	26	2006
13,5	14,5	0,6	2	2	19	2009
13,5	14,5	0,6	3	2	22	2011
13,5	14,5	0,6	2	2	19	2013
13,5	14,5	0,6	2	2	19	2013
15,2	20,7	0,6	3	2	18	2001
15,2	20,7	0,6	3	2	19	2010
15,2	20,7	0,6	3	2	19	2010
15,2	20,7	0,6	4	2	21	2011
15,2	20,7	0,6	3	2	15	2015
15,2	20,7	0,6	4	2	21	2015

16,4	26	0,6	6	2	23	2008
16,1	24,6	0,6	5	2	19	2012
14,9	19,5	0,6	4	2	19	2012
15,1	20,3	0,6	4	2	19	1999
15,1	20,3	0,6	4	2	20	2001
15	19,9	0,6	3	2	23	2001
15	19,9	0,6	4	2	25	2004
15	19,9	0,6	5	2	22	2005
15	19,9	0,6	3	2	20	2012
15	19,9	0,6	3	2	18	2014
15	19,9	0,6	3	2	19	2014
13,8	15,5	0,6	2	2	24	2006
13,8	15,5	0,6	3	2	18	2014
13,8	15,5	0,6	3	2	16	2015
18	34,4	0,6	4	2	19	2013
12,6	11,8	0,6	2	2	21	2010
12,6	11,8	0,6	2	2	21	2010
16,5	26,5	0,6	5	2	20	2003
18,8	39,2	0,6	4	2	19	2010
18,8	39,2	0,6	4	2	19	2010
17,1	29,5	0,6	5	2	21	2000
15,6	22,4	0,6	6	2	23	2010
15,6	22,4	0,6	6	2	23	2010
15,6	22,4	0,6	3	2	19	2013
15,6	22,4	0,6	4	2	15	2015
13,4	14,2	0,6	2	2	24	2002
13,4	14,2	0,6	2	2	23	2010
13,4	14,2	0,6	2	2	23	2010

13,4	14,2	0,6	2	2	20	2013
13,4	14,2	0,6	2	2	20	2013
14,2	16,9	0,6	2	2	26	2000
14,2	16,9	0,6	4	2	21	2001
14,2	16,9	0,6	3	2	18	2008
14,2	16,9	0,6	3	2	23	2010
14,2	16,9	0,6	3	2	23	2010
14,2	16,9	0,6	2	2	19	2013
14,2	16,9	0,6	3	2	17	2014
17	29	0,6	5	2	20	2010
17	29	0,6	5	2	20	2010
12	10,2	0,6	2	2	25	2007
12	10,2	0,6	2	2	19	2013
16,2	25,1	0,6	10	2	23	2005
14	16,2	0,6	3	2	23	2005
14	16,2	0,6	4	2	23	2011
14	16,2	0,6	2	2	19	2013
14,5	18	0,6	4	2	20	1999
14,5	18	0,6	5	2	21	1999
14,5	18	0,6	5	2	23	2009
14,5	18	0,6	3	2	19	2014
14,5	18	0,6	4	2	18	2015
16,9	28,5	0,6	4	2	20	2001
12,7	12,1	0,6	2	2	21	2005
12,7	12,1	0,6	2	2	22	2009
15,8	23,3	0,6	3	2	18	2000
16	24,2	0,6	5	2	20	2000
16	24,2	0,6	3	2	22	2003

13,3	13,9	0,6	2	2	18	2000
13,3	13,9	0,6	5	2	19	2007
13,3	13,9	0,6	2	2	19	2009
13,3	13,9	0,6	6	2	24	2009
13,3	13,9	0,6	2	2	23	2010
13,3	13,9	0,6	8	2	23	2010
13,3	13,9	0,6	2	2	23	2010
13,3	13,9	0,6	8	2	23	2010
13,3	13,9	0,6	3	2	21	2011
13,3	13,9	0,6	2	2	23	2011
13,3	13,9	0,6	2	2	19	2013
13,3	13,9	0,6	2	2	18	2014
16,3	25,6	0,6	5	2	22	2004
12,3	11	0,6	2	2	20	2002
12,3	11	0,6	3	2	25	2004
12,3	11	0,6	2	2	23	2010
12,3	11	0,6	2	2	23	2010
13,7	15,2	0,6	2	2	22	1999
13,7	15,2	0,6	2	2	21	2008
13,7	15,2	0,6	3	2	23	2010
13,7	15,2	0,6	3	2	23	2010
13,2	13,6	0,6	3	2	20	2008
13,2	13,6	0,6	3	2	19	2010
13,2	13,6	0,6	3	2	19	2010
13,2	13,6	0,6	2	2	19	2013
13,2	13,6	0,6	2	2	19	2013
15,4	21,6	0,6	3	2	19	2002
18	34,5	0,6	8	2	20	2010

18	34,5	0,6	8	2	20	2010
12,9	12,7	0,6	2	2	19	2013
12,9	12,7	0,6	2	2	17	2014
12,9	12,7	0,6	2	2	18	2014
13,1	13,3	0,6	2	2	21	2000
13,1	13,3	0,6	2	2	23	2003
13,1	13,3	0,6	2	2	19	2013
13,1	13,3	0,6	3	2	15	2015
14,3	17,3	0,6	3	2	18	2008
14,3	17,3	0,6	2	2	21	2008
14,3	17,3	0,6	4	2	26	2009
14,3	17,3	0,6	4	2	22	2011
16,4	26,1	0,6	5	2	19	2000
13	13	0,6	2	2	21	2006
13	13	0,6	2	2	17	2014
13	13	0,6	2	2	19	2014
15,7	22,9	0,6	3	2	18	2000
15,7	22,9	0,6	5	2	26	2000
15,7	22,9	0,6	4	2	19	2002
15,7	22,9	0,6	4	2	22	2003
16,1	24,7	0,6	3	2	21	2010
16,1	24,7	0,6	3	2	21	2010
15,3	21,2	0,6	3	2	21	2000
15,3	21,2	0,6	4	2	22	2004
13,9	15,9	0,6	2	2	24	2001
13,9	15,9	0,6	3	2	25	2005
15,9	23,8	0,6	4	2	21	2005
16,5	26,6	0,6	6	2	17	2009

16,5	26,6	0,6	4	2	18	2015
14,1	16,6	0,6	3	2	21	2001
14,1	16,6	0,6	4	2	20	2009
14,1	16,6	0,6	5	2	23	2010
14,1	16,6	0,6	5	2	23	2010
14,1	16,6	0,6	3	2	16	2015
17,4	31,2	0,6	6	2	19	2010
17,4	31,2	0,6	6	2	19	2010
17,4	31,2	0,6	7	2	17	2014
14,8	19,2	0,6	2	2	19	2013
15,2	20,8	0,6	4	2	18	2001
15,2	20,8	0,6	4	2	19	2006
15,2	20,8	0,6	4	2	17	2009
13,6	14,9	0,6	4	2	19	1999
13,6	14,9	0,6	4	2	26	2006
13,6	14,9	0,6	2	2	22	2011
13,6	14,9	0,6	2	2	19	2014
14,9	19,6	0,6	4	2	19	2014
15,1	20,4	0,6	5	2	21	1999
15	20	0,6	4	2	23	2004
15	20	0,6	4	2	19	2007
15	20	0,6	4	2	22	2011
16,7	27,6	0,6	5	2	18	2000
16,7	27,6	0,6	7	2	18	2014
12,4	11,3	0,6	2	2	23	2007
12,1	10,5	0,6	2	2	26	2006
16,2	25,2	0,6	6	2	20	2003
14,4	17,7	0,6	4	2	20	2011

15,8	23,4	0,6	4	2	22	2004
15,8	23,4	0,6	5	2	24	2006
15,8	23,4	0,6	3	2	21	2015
16	24,3	0,6	5	2	20	2001
16	24,3	0,6	6	2	20	2010
16	24,3	0,6	6	2	20	2010
16	24,3	0,6	5	2	18	2012
16	24,3	0,6	4	2	19	2014
16	24,3	0,6	7	2	21	2014
16	24,3	0,6	4	2	21	2015
13,5	14,6	0,6	3	2	23	2005
13,5	14,6	0,6	3	2	19	2007
13,5	14,6	0,6	2	2	17	2008
13,5	14,6	0,6	3	2	20	2008
16,3	25,7	0,6	6	2	18	2014
15,5	22,1	0,6	7	2	21	1999
15,5	22,1	0,6	5	2	18	2000
13,8	15,6	0,6	2	2	25	2003
13,8	15,6	0,6	3	2	19	2007
18,6	38,2	0,6	6	2	15	2015
14,5	18,1	0,6	3	2	20	2008
14,5	18,1	0,6	4	2	19	2013
14,2	17	0,6	5	2	19	2010
14,2	17	0,6	5	2	19	2010
14,2	17	0,6	3	2	21	2014
12,5	11,6	0,6	2	2	23	2010
12,5	11,6	0,6	2	2	23	2010
14	16,3	0,6	3	2	19	2005

14	16,3	0,6	2	2	17	2012
14	16,3	0,6	3	2	20	2012
14	16,3	0,6	3	2	18	2014
14	16,3	0,6	3	2	21	2014
20,3	49,7	0,6	5	2	19	2010
20,3	49,7	0,6	5	2	19	2010
15,4	21,7	0,6	3	2	25	2004
15,4	21,7	0,6	4	2	22	2011
15,4	21,7	0,6	4	2	16	2015
16,1	24,8	0,6	5	2	19	2004
16,1	24,8	0,6	4	2	21	2013
13,4	14,3	0,6	5	2	22	2009
13,4	14,3	0,6	2	2	23	2009
13,4	14,3	0,6	3	2	24	2009
13,4	14,3	0,6	2	2	19	2010
13,4	14,3	0,6	2	2	19	2010
13,4	14,3	0,6	2	2	22	2011
13,4	14,3	0,6	2	2	19	2013
13,4	14,3	0,6	3	2	18	2014
13,4	14,3	0,6	2	2	21	2014
16,5	26,7	0,6	5	2	20	2010
16,5	26,7	0,6	6	2	21	2010
16,5	26,7	0,6	5	2	20	2010
16,5	26,7	0,6	6	2	21	2010
21,6	59,9	0,6	10	2	19	2010
21,6	59,9	0,6	10	2	19	2010
14,6	18,5	0,6	3	2	18	2014
14,6	18,5	0,6	3	2	18	2014

15,9	23,9	0,6	3	2	19	2014
16,9	28,7	0,6	4	2	19	2013
16,6	27,2	0,6	4	2	19	2002
15,3	21,3	0,6	3	2	22	1999
15,3	21,3	0,6	5	2	20	2007
16,7	27,7	0,6	6	2	23	2003
12,2	10,8	0,6	2	2	22	2007
14,7	18,9	0,6	6	2	24	2006
13,7	15,3	0,6	2	2	22	2000
13,7	15,3	0,6	2	2	22	2003
13,7	15,3	0,6	4	2	20	2009
13,7	15,3	0,6	3	2	21	2010
13,7	15,3	0,6	3	2	21	2010
13,7	15,3	0,6	2	2	20	2013
13,7	15,3	0,6	2	2	20	2013
13,3	14	0,6	2	2	19	2002
13,3	14	0,6	2	2	25	2003
16,2	25,3	0,6	5	2	19	2004
15,2	20,9	0,6	3	2	20	2001
15,2	20,9	0,6	3	2	20	2001
15,2	20,9	0,6	3	2	17	2008
15,2	20,9	0,6	5	2	23	2009
15,2	20,9	0,6	4	2	19	2013
15,2	20,9	0,6	3	2	18	2015
15,6	22,6	0,6	3	2	20	2003
15,6	22,6	0,6	3	2	19	2004
15,6	22,6	0,6	5	2	18	2014
14,8	19,3	0,6	5	2	19	2007

15,1	20,5	0,6	3	2	20	2003
15,1	20,5	0,6	4	2	21	2003
17,2	30,3	0,6	5	2	21	2010
17,2	30,3	0,6	5	2	21	2010
18,2	35,9	0,6	7	2	17	2014
15	20,1	0,6	3	2	20	2000
15	20,1	0,6	3	2	25	2003
15	20,1	0,6	3	2	20	2011
15	20,1	0,6	4	2	17	2012
15	20,1	0,6	3	2	15	2015
15	20,1	0,6	3	2	16	2015
12,7	12,2	0,6	2	2	19	2002
13,2	13,7	0,6	2	2	20	2000
13,2	13,7	0,6	3	2	21	2008
13,2	13,7	0,6	2	2	23	2009
13,2	13,7	0,6	3	2	23	2010
13,2	13,7	0,6	3	2	23	2010
16	24,4	0,6	6	2	25	2007
14,1	16,7	0,6	2	2	17	2002
14,1	16,7	0,6	4	2	20	2009
14,1	16,7	0,6	3	2	19	2014
13,9	16	0,6	2	2	22	2001
13,9	16	0,6	2	2	18	2014
17,8	33,6	0,6	5	2	20	2010
17,8	33,6	0,6	5	2	20	2010
12,8	12,5	0,6	2	2	21	2005
12,8	12,5	0,6	2	2	17	2014
13,1	13,4	0,6	3	2	19	2005

13,1	13,4	0,6	2	2	26	2006
13,1	13,4	0,6	2	2	21	2008
13,1	13,4	0,6	2	2	20	2009
13,1	13,4	0,6	2	2	20	2011
13,1	13,4	0,6	2	2	23	2011
13,1	13,4	0,6	2	2	17	2014
13,1	13,4	0,6	2	2	18	2014
13,1	13,4	0,6	2	2	18	2014
13,1	13,4	0,6	2	2	18	2014
13,1	13,4	0,6	2	2	19	2014
12	10,3	0,6	2	2	23	2007
12	10,3	0,6	2	2	25	2007
12	10,3	0,6	2	2	19	2013
14,4	17,8	0,6	2	2	21	2000
14,4	17,8	0,6	4	2	21	2015
15,5	22,2	0,6	4	2	20	2013
15,5	22,2	0,6	7	2	18	2014
15,5	22,2	0,6	4	2	15	2015
15,5	22,2	0,6	3	2	18	2015
16,4	26,3	0,6	6	2	26	2001
13	13,1	0,6	2	2	26	2006
13	13,1	0,6	2	2	19	2013
13	13,1	0,6	1	2	22	2013
13	13,1	0,6	2	2	19	2014
13,6	15	0,6	2	2	19	2001
13,6	15	0,6	3	2	22	2007
13,6	15	0,6	2	2	23	2008
13,6	15	0,6	2	2	19	2013

13,6	15	0,6	3	2	19	2014
12,3	11,1	0,6	2	2	18	2014
16,5	26,8	0,6	3	2	20	2003
18	34,8	0,6	4	2	19	2012
15,4	21,8	0,6	5	2	18	1999
15,4	21,8	0,6	6	2	19	2010
15,4	21,8	0,6	6	2	19	2010
17,7	33,1	0,6	6	2	20	2003
15,7	23,1	0,6	4	2	20	2011
14,5	18,2	0,6	2	2	20	2001
14,5	18,2	0,6	3	2	20	2013
14,5	18,2	0,6	2	2	17	2014
14,5	18,2	0,6	4	2	15	2015
14,5	18,2	0,6	3	2	18	2015
15,9	24	0,6	3	2	17	2014
14,2	17,1	0,6	2	2	20	2008
14,2	17,1	0,6	3	2	23	2008
14,2	17,1	0,6	4	2	20	2009
14,2	17,1	0,6	3	2	23	2015
13,8	15,7	0,6	3	2	22	2005
13,8	15,7	0,6	3	2	17	2014
13,5	14,7	0,6	2	2	23	2010
13,5	14,7	0,6	2	2	23	2010
13,5	14,7	0,6	2	2	19	2014
13,5	14,7	0,6	2	2	21	2014
13,5	14,7	0,6	2	2	23	2014
13,5	14,7	0,6	4	2	18	2015
15,3	21,4	0,6	3	2	21	2001

14,6	18,6	0,6	3	2	21	2000
14,6	18,6	0,6	3	2	19	2008
14	16,4	0,6	2	2	21	2000
14	16,4	0,6	2	2	24	2001
14	16,4	0,6	3	2	18	2008
14	16,4	0,6	4	2	20	2012
14	16,4	0,6	3	2	19	2013
14	16,4	0,6	2	2	18	2014
14	16,4	0,6	3	2	21	2014
12,4	11,4	0,6	2	2	20	2004
15,6	22,7	0,6	4	2	22	2011
17,4	31,5	0,6	5	2	19	2013
15,2	21	0,6	5	2	17	2002
15,2	21	0,6	6	2	20	2007
15,2	21	0,6	3	2	15	2015
16,3	25,9	0,6	4	2	18	1999
16,3	25,9	0,6	4	2	22	2001
14,7	19	0,6	3	2	20	2008
14,7	19	0,6	4	2	21	2010
14,7	19	0,6	4	2	21	2010
14,7	19	0,6	4	2	23	2011
16	24,5	0,6	5	2	19	2014
16	24,5	0,6	4	2	21	2014
15,1	20,6	0,6	4	2	21	2006
15,1	20,6	0,6	3	2	20	2008
15,1	20,6	0,6	5	2	19	2009
11,5	9,1	0,6	2	2	23	2004
12,1	10,6	0,6	2	2	19	2007

12,1	10,6	0,6	2	2	19	2007
14,8	19,4	0,6	5	2	18	2000
14,8	19,4	0,6	3	2	20	2001
14,3	17,5	0,6	4	2	23	2011
14,3	17,5	0,6	2	2	19	2012
14,3	17,5	0,6	3	2	18	2014
14,3	17,5	0,6	3	2	19	2014
14,3	17,5	0,6	3	2	21	2015
13,4	14,4	0,6	3	2	22	2005
13,4	14,4	0,6	3	2	20	2007
13,4	14,4	0,6	2	2	25	2008
16,4	26,4	0,6	5	2	18	2000
16,4	26,4	0,6	3	2	20	2003
15	20,2	0,6	6	2	23	2011
15	20,2	0,6	3	2	18	2014
14,9	19,8	0,6	5	2	21	2000
16,9	28,9	0,6	5	2	19	2014
16,5	26,9	0,6	5	2	18	2000
15,5	22,3	0,6	3	2	22	2001
15,5	22,3	0,6	5	2	22	2007
15,5	22,3	0,6	3	2	18	2015
13,7	15,4	0,6	2	2	22	2001
13,7	15,4	0,6	3	2	22	2004
13,7	15,4	0,6	3	2	24	2009
13,7	15,4	0,6	3	2	23	2011
13,7	15,4	0,6	2	2	16	2015
17,5	32,1	0,6	8	2	19	2015
16,7	27,9	0,6	4	2	19	2001

14,1	16,8	0,6	3	2	20	2007
14,1	16,8	0,6	4	2	21	2011
14,1	16,8	0,6	3	2	16	2015
13,3	14,1	0,6	4	2	25	2005
13,3	14,1	0,6	5	2	22	2007
13,3	14,1	0,6	2	2	19	2013
11,9	10,1	0,6	2	2	18	2007
13,9	16,1	0,6	4	2	23	2001
13,9	16,1	0,6	3	2	25	2003
15,7	23,2	0,6	3	2	24	2001
15,9	24,1	0,6	3	2	20	2001
15,4	21,9	0,6	3	2	18	2000
15,4	21,9	0,6	3	2	19	2002
15,4	21,9	0,6	3	2	19	2004
16,2	25,5	0,6	4	2	19	2002
12,6	12	0,6	2	2	19	2009
12,6	12	0,6	2	2	20	2013
17,1	30	0,6	5	2	19	2014
13,2	13,8	0,6	2	2	17	2002
13,2	13,8	0,6	2	2	19	2002
13,2	13,8	0,6	3	2	22	2007
13,2	13,8	0,6	2	2	21	2009
13,2	13,8	0,6	2	2	26	2009
13,2	13,8	0,6	2	2	19	2014
13,2	13,8	0,6	3	2	19	2014
12,2	10,9	0,6	2	2	22	2011
12,2	10,9	0,6	2	2	18	2014
14,5	18,3	0,6	5	2	22	2002

14,5	18,3	0,6	3	2	21	2003
14,5	18,3	0,6	3	2	19	2008
13,6	15,1	0,6	3	2	17	2014
15,3	21,5	0,6	5	2	23	1999
15,3	21,5	0,6	4	2	25	2004
16,3	26	0,6	3	2	24	2001
16,3	26	0,6	3	2	20	2003
16,3	26	0,6	4	2	21	2003
19,2	42,5	0,6	7	2	17	2012
12,7	12,3	0,6	2	2	18	2014
13,1	13,5	0,6	2	2	21	2000
13,1	13,5	0,6	2	2	20	2002
15,6	22,8	0,6	4	2	20	2003
15,6	22,8	0,6	8	2	20	2008
16	24,6	0,6	8	2	20	2013
16	24,6	0,6	3	2	15	2015
17,3	31,1	0,6	4	2	19	2014
19	41,2	0,6	7	2	19	2014
14,2	17,2	0,6	5	2	26	1999
14,2	17,2	0,6	2	2	23	2001
14,2	17,2	0,6	4	2	22	2005
14,2	17,2	0,6	4	2	26	2006
14,2	17,2	0,6	3	2	23	2010
14,2	17,2	0,6	3	2	23	2010
14,2	17,2	0,6	3	2	21	2014
14,2	17,2	0,6	3	2	21	2015
11,4	8,9	0,6	2	2	25	2004
16,9	29	0,6	10	2	23	2007

12,8	12,6	0,6	2	2	20	2001
13	13,2	0,6	2	2	22	1999
13	13,2	0,6	3	2	23	2008
13	13,2	0,6	2	2	23	2009
13	13,2	0,6	2	2	23	2011
13	13,2	0,6	2	2	18	2014
13	13,2	0,6	2	2	18	2014
15,2	21,1	0,6	4	2	25	2003
15,2	21,1	0,6	4	2	23	2004
15,2	21,1	0,6	5	2	23	2010
15,2	21,1	0,6	5	2	23	2010
15,2	21,1	0,6	5	2	17	2014
15,2	21,1	0,6	3	2	18	2015
15,2	21,1	0,6	3	2	21	2015
15,8	23,7	0,6	3	2	21	2003
15,8	23,7	0,6	4	2	25	2004
15,8	23,7	0,6	5	2	19	2012
14,6	18,7	0,6	5	2	19	2007
14,6	18,7	0,6	4	2	23	2009
11	8	0,6	2	2	20	2004
13,8	15,8	0,6	2	2	18	2000
13,8	15,8	0,6	2	2	21	2001
13,8	15,8	0,6	3	2	19	2014
13,8	15,8	0,6	2	2	21	2014
15,1	20,7	0,6	4	2	20	1999
15,1	20,7	0,6	4	2	25	2004
15,1	20,7	0,6	6	2	21	2005
15,1	20,7	0,6	4	2	19	2006

15,1	20,7	0,6	4	2	23	2006
15,1	20,7	0,6	5	2	19	2012
14,7	19,1	0,6	3	2	20	2000
14,7	19,1	0,6	3	2	20	2003
14	16,5	0,6	4	2	20	1999
14	16,5	0,6	4	2	21	2005
14	16,5	0,6	2	2	22	2009
14	16,5	0,6	3	2	17	2014
14	16,5	0,6	3	2	15	2015
16,1	25,1	0,6	5	2	20	1999
15	20,3	0,6	3	2	19	2011
15	20,3	0,6	3	2	17	2012
15	20,3	0,6	3	2	18	2015
15	20,3	0,6	4	2	22	2015
14,8	19,5	0,6	2	2	21	2000
14,8	19,5	0,6	6	2	19	2011
15,5	22,4	0,6	5	2	21	2000
15,5	22,4	0,6	3	2	21	2001
13,5	14,8	0,6	4	2	23	2010
13,5	14,8	0,6	4	2	23	2010
13,5	14,8	0,6	3	2	21	2011
14,9	19,9	0,6	3	2	21	2000
14,9	19,9	0,6	5	2	24	2009
19,6	45,3	0,6	5	2	19	2010
19,6	45,3	0,6	5	2	19	2010
12	10,4	0,6	3	2	22	2005
12	10,4	0,6	1	2	24	2006
12	10,4	0,6	2	2	20	2012

12	10,4	0,6	2	2	19	2014
12	10,4	0,6	2	2	19	2014
12,3	11,2	0,6	2	2	17	2014
12,3	11,2	0,6	2	2	19	2014
14,3	17,6	0,6	4	2	20	1999
14,3	17,6	0,6	6	2	21	2013
14,3	17,6	0,6	3	2	19	2014
15,9	24,2	0,6	5	2	20	1999
15,7	23,3	0,6	7	2	23	2009
15,7	23,3	0,6	4	2	21	2015
11,6	9,4	0,6	2	2	23	2007
17,7	33,4	0,6	5	2	19	2010
17,7	33,4	0,6	5	2	19	2010
15,4	22	0,6	6	2	21	1999
15,4	22	0,6	3	2	22	2001
15,4	22	0,6	3	2	21	2003
15,4	22	0,6	4	2	23	2007
15,4	22	0,6	4	2	16	2015
15,4	22	0,6	4	2	16	2015
13,4	14,5	0,6	2	2	22	2001
13,4	14,5	0,6	2	2	20	2006
13,4	14,5	0,6	3	2	25	2008
13,4	14,5	0,6	2	2	18	2014
16,3	26,1	0,6	3	2	18	2015
13,7	15,5	0,6	2	2	24	2001
13,7	15,5	0,6	3	2	15	2015
14,4	18	0,6	3	2	24	2002
14,4	18	0,6	3	2	17	2008

14,4	18	0,6	4	2	19	2009
14,1	16,9	0,6	2	2	17	2002
14,1	16,9	0,6	2	2	25	2003
14,1	16,9	0,6	3	2	18	2014
16	24,7	0,6	6	2	26	2000
16	24,7	0,6	6	2	23	2011
15,3	21,6	0,6	7	2	26	1999
15,3	21,6	0,6	4	2	20	2009
15,3	21,6	0,6	3	2	15	2015
15,6	22,9	0,6	5	2	22	2000
15,6	22,9	0,6	3	2	18	2015
13,9	16,2	0,6	3	2	21	2006
10,8	7,6	0,6	1	2	18	2007
16,7	28,1	0,6	3	2	22	2003
14,5	18,4	0,6	3	2	20	2000
14,5	18,4	0,6	4	2	22	2005
14,5	18,4	0,6	4	2	22	2005
13,3	14,2	0,6	3	2	20	2007
13,3	14,2	0,6	3	2	20	2008
15,2	21,2	0,6	4	2	19	2002
15,2	21,2	0,6	4	2	22	2004
15,2	21,2	0,6	5	2	20	2007
15,2	21,2	0,6	6	2	20	2011
15,2	21,2	0,6	3	2	15	2015
17,1	30,2	0,6	8	2	17	2009
12,1	10,7	0,6	4	2	20	2006
12,1	10,7	0,6	2	2	25	2007
10,7	7,4	0,6	2	2	23	2004

14,6	18,8	0,6	2	2	19	1999
14,6	18,8	0,6	6	2	21	2006
14,6	18,8	0,6	3	2	20	2008
15,1	20,8	0,6	6	2	24	2006
15,1	20,8	0,6	5	2	26	2006
12,5	11,8	0,6	2	2	22	2011
12,5	11,8	0,6	2	2	19	2013
14,2	17,3	0,6	3	2	22	2003
14,2	17,3	0,6	3	2	19	2005
14,2	17,3	0,6	3	2	17	2008
14,2	17,3	0,6	6	2	20	2008
14,2	17,3	0,6	4	2	20	2008
14,2	17,3	0,6	2	2	20	2008
15,5	22,5	0,6	4	2	17	2009
13,6	15,2	0,6	3	2	21	2005
13,6	15,2	0,6	3	2	21	2011
18,9	40,8	0,6	5	2	20	2012
13,2	13,9	0,6	3	2	20	2007
13,2	13,9	0,6	2	2	23	2009
13,2	13,9	0,6	2	2	21	2013
14,7	19,2	0,6	2	2	21	2000
14,7	19,2	0,6	3	2	19	2001
14,7	19,2	0,6	3	2	19	2001
14,7	19,2	0,6	3	2	18	2015
15	20,4	0,6	4	2	21	2001
15	20,4	0,6	3	2	23	2003
15	20,4	0,6	4	2	17	2009
15	20,4	0,6	3	2	19	2012

16,2	25,7	0,6	4	2	21	2001
17	29,7	0,6	3	2	16	2015
14,8	19,6	0,6	5	2	19	2009
14,9	20	0,6	3	2	20	2003
14,9	20	0,6	3	2	17	2012
15,7	23,4	0,6	4	2	18	2000
11,5	9,2	0,6	2	2	23	2011
16,9	29,2	0,6	5	2	19	2014
14	16,6	0,6	4	2	22	1999
14	16,6	0,6	2	2	20	2000
14	16,6	0,6	4	2	25	2005
14	16,6	0,6	4	2	19	2006
14	16,6	0,6	3	2	20	2011
14	16,6	0,6	2	2	21	2012
14	16,6	0,6	2	2	19	2013
14	16,6	0,6	3	2	15	2015
13,1	13,6	0,6	3	2	20	2007
13,1	13,6	0,6	2	2	26	2009
16,3	26,2	0,6	6	2	18	2000
13,8	15,9	0,6	4	2	20	2011
13,8	15,9	0,6	3	2	19	2012
11,9	10,2	0,6	2	2	26	2006
14,3	17,7	0,6	3	2	20	2002
14,3	17,7	0,6	4	2	20	2012
13	13,3	0,6	2	2	23	2007
13	13,3	0,6	2	2	19	2013
13	13,3	0,6	2	2	19	2013
13	13,3	0,6	2	2	19	2014

13	13,3	0,6	2	2	21	2014
13	13,3	0,6	3	2	21	2015
16	24,8	0,6	5	2	19	2010
16	24,8	0,6	5	2	19	2010
16	24,8	0,6	3	2	18	2015
16,5	27,2	0,6	4	2	18	2002
12,8	12,7	0,6	3	2	22	2005
13,5	14,9	0,6	2	2	20	2006
13,5	14,9	0,6	2	2	19	2010
13,5	14,9	0,6	2	2	19	2010
13,5	14,9	0,6	3	2	19	2013
11,7	9,7	0,6	1	2	26	2001
12,2	11	0,6	4	2	26	2006
12,2	11	0,6	2	2	18	2007
15,6	23	0,6	3	2	21	2000
15,6	23	0,6	3	2	20	2003
15,6	23	0,6	7	2	19	2012
15,6	23	0,6	5	2	19	2013
15,3	21,7	0,6	5	2	19	1999
15,3	21,7	0,6	3	2	26	2003
15,3	21,7	0,6	4	2	20	2006
15,3	21,7	0,6	10	2	19	2009
15,3	21,7	0,6	3	2	21	2010
15,3	21,7	0,6	3	2	21	2010
18,6	39	0,6	5	2	20	2010
18,6	39	0,6	5	2	20	2010
14,4	18,1	0,6	3	2	26	2003
14,4	18,1	0,6	3	2	16	2008

16,1	25,3	0,6	4	2	17	2012
20,6	53	0,6	5	2	17	2012
14,1	17	0,6	2	2	20	2000
14,1	17	0,6	3	2	20	2001
14,1	17	0,6	2	2	21	2001
14,1	17	0,6	4	2	21	2001
14,1	17	0,6	2	2	23	2003
14,1	17	0,6	3	2	21	2014
15,2	21,3	0,6	3	2	17	2014
15,2	21,3	0,6	4	2	17	2014
15,2	21,3	0,6	3	2	18	2015
15,2	21,3	0,6	3	2	19	2015
17	29,8	0,6	4	2	16	2008
13,4	14,6	0,6	2	2	18	2014
14,5	18,5	0,6	3	2	23	2001
14,5	18,5	0,6	4	2	23	2011
15,5	22,6	0,6	3	2	22	2003
15,5	22,6	0,6	3	2	22	2003
13,9	16,3	0,6	3	2	25	2007
16,9	29,3	0,6	4	2	17	2008
12,3	11,3	0,6	2	2	26	2006
12,3	11,3	0,6	3	2	21	2011
12,3	11,3	0,6	2	2	20	2013
16,3	26,3	0,6	4	2	19	2002
16,3	26,3	0,6	4	2	20	2013
14,6	18,9	0,6	3	2	16	2015
16,8	28,8	0,6	9	2	21	2000
16,8	28,8	0,6	7	2	21	2014

15	20,5	0,6	3	2	22	2003
15	20,5	0,6	4	2	25	2004
15	20,5	0,6	4	2	20	2011
15	20,5	0,6	3	2	17	2014
15	20,5	0,6	4	2	21	2015
11,4	9	0,6	2	2	20	2004
11,4	9	0,6	2	2	20	2004
11,4	9	0,6	2	2	25	2004
20	48,6	0,6	10	2	18	2012
16,4	26,8	0,6	4	2	21	2012
14,7	19,3	0,6	4	2	21	1999
14,7	19,3	0,6	4	2	23	2003
14,7	19,3	0,6	3	2	22	2009
14,7	19,3	0,6	3	2	20	2013
14,7	19,3	0,6	3	2	18	2014
14,7	19,3	0,6	3	2	18	2015
16,7	28,3	0,6	3	2	18	2000
16,7	28,3	0,6	7	2	17	2014
14,9	20,1	0,6	3	2	19	2012
12	10,5	0,6	2	2	23	2007
12	10,5	0,6	2	2	20	2013
14,8	19,7	0,6	3	2	21	2001
14,8	19,7	0,6	5	2	19	2008
14,8	19,7	0,6	4	2	23	2010
14,8	19,7	0,6	4	2	23	2010
14,2	17,4	0,6	2	2	21	2001
14,2	17,4	0,6	4	2	23	2009
16,6	27,8	0,6	6	2	20	2001

13,3	14,3	0,6	2	2	19	2002
13,3	14,3	0,6	2	2	23	2009
13,3	14,3	0,6	2	2	18	2014
15,4	22,2	0,6	3	2	19	2013
16	24,9	0,6	5	2	19	2000
16	24,9	0,6	3	2	19	2012
17,1	30,4	0,6	3	2	16	2015
13,6	15,3	0,6	4	2	25	1999
13,6	15,3	0,6	2	2	17	2014
15,8	24	0,6	3	2	19	2000
15,8	24	0,6	4	2	18	2001
15,8	24	0,6	7	2	21	2006
14	16,7	0,6	2	2	24	2001
14	16,7	0,6	2	2	24	2006
14	16,7	0,6	3	2	22	2009
14	16,7	0,6	4	2	22	2009
14	16,7	0,6	4	2	22	2011
14	16,7	0,6	2	2	17	2014
11,8	10	0,6	2	2	19	2013
16,1	25,4	0,6	4	2	25	1999
16,1	25,4	0,6	3	2	22	2003
16,1	25,4	0,6	6	2	21	2011
16,1	25,4	0,6	4	2	17	2012
16,1	25,4	0,6	4	2	18	2012
15,3	21,8	0,6	3	2	20	2000
15,3	21,8	0,6	2	2	24	2002
15,3	21,8	0,6	4	2	22	2004
15,3	21,8	0,6	6	2	19	2006

15,3	21,8	0,6	3	2	16	2008
15,3	21,8	0,6	4	2	18	2008
15,3	21,8	0,6	6	2	19	2013
13,2	14	0,6	2	2	18	2002
13,2	14	0,6	2	2	23	2003
13,2	14	0,6	4	2	26	2009
13,2	14	0,6	2	2	19	2014
14,3	17,8	0,6	2	2	26	2001
14,3	17,8	0,6	2	2	24	2006
14,3	17,8	0,6	3	2	20	2008
13,8	16	0,6	2	2	18	2000
16,9	29,4	0,6	7	2	18	2001
16,2	25,9	0,6	8	2	26	2005
16,2	25,9	0,6	6	2	20	2013
17,2	31	0,6	5	2	17	2014
12,5	11,9	0,6	3	2	22	2005
12,5	11,9	0,6	2	2	20	2010
12,5	11,9	0,6	2	2	20	2010
15,2	21,4	0,6	6	2	18	2000
15,2	21,4	0,6	3	2	17	2002
15,2	21,4	0,6	3	2	21	2003
15,2	21,4	0,6	6	2	22	2007
15,2	21,4	0,6	3	2	22	2007
14,4	18,2	0,6	2	2	19	2000
14,4	18,2	0,6	3	2	21	2001
14,4	18,2	0,6	5	2	23	2009
14,4	18,2	0,6	4	2	20	2011
14,4	18,2	0,6	3	2	20	2013

15,5	22,7	0,6	4	2	18	2014
16,3	26,4	0,6	7	2	19	2012
12,1	10,8	0,6	2	2	23	2006
12,1	10,8	0,6	2	2	21	2011
12,1	10,8	0,6	2	2	22	2011
15,7	23,6	0,6	4	2	20	2011
15,7	23,6	0,6	3	2	19	2014
11,3	8,8	0,6	2	2	24	2006
13	13,4	0,6	2	2	22	2001
13	13,4	0,6	2	2	19	2002
13	13,4	0,6	2	2	21	2006
13	13,4	0,6	2	2	21	2008
13	13,4	0,6	2	2	21	2008
13	13,4	0,6	2	2	22	2011
13	13,4	0,6	2	2	19	2013
13	13,4	0,6	2	2	19	2013
13	13,4	0,6	2	2	19	2014
15,1	21	0,6	3	2	24	2002
15,1	21	0,6	3	2	21	2003
16,5	27,4	0,6	5	2	20	1999
16,5	27,4	0,6	9	2	22	2003
14,1	17,1	0,6	6	2	22	2003
14,1	17,1	0,6	4	2	21	2005
14,1	17,1	0,6	3	2	19	2014
14,5	18,6	0,6	5	2	23	1999
14,5	18,6	0,6	4	2	25	2004
14,5	18,6	0,6	3	2	18	2008
12,7	12,5	0,6	2	2	18	2002

12,9	13,1	0,6	2	2	23	2001
12,9	13,1	0,6	3	2	18	2014
18,3	37,4	0,6	4	2	16	2015
18,3	37,4	0,6	6	2	18	2015
12,8	12,8	0,6	5	2	25	1999
12,8	12,8	0,6	2	2	20	2004
12,8	12,8	0,6	2	2	23	2006
12,8	12,8	0,6	2	2	19	2013
12,8	12,8	0,6	2	2	21	2013
16	25	0,6	7	2	23	2002
16	25	0,6	4	2	20	2003
15	20,6	0,6	5	2	19	1999
15	20,6	0,6	3	2	20	2003
15	20,6	0,6	6	2	25	2004
15	20,6	0,6	3	2	20	2006
15	20,6	0,6	7	2	19	2007
15	20,6	0,6	3	2	19	2007
15	20,6	0,6	5	2	22	2007
15	20,6	0,6	4	2	23	2010
15	20,6	0,6	4	2	23	2010
15	20,6	0,6	4	2	18	2014
14,6	19	0,6	4	2	19	2009
14,6	19	0,6	3	2	16	2015
13,7	15,7	0,6	3	2	22	2001
13,7	15,7	0,6	2	2	23	2003
13,7	15,7	0,6	3	2	25	2004
13,7	15,7	0,6	2	2	24	2009
13,7	15,7	0,6	3	2	23	2010

13,7	15,7	0,6	3	2	23	2010
15,4	22,3	0,6	5	2	21	1999
17	30	0,6	4	2	17	2012
14,9	20,2	0,6	3	2	21	2001
14,9	20,2	0,6	3	2	25	2003
13,9	16,4	0,6	3	2	20	2011
14,7	19,4	0,6	6	2	24	2001
14,7	19,4	0,6	3	2	20	2003
19	41,9	0,6	5	2	18	2012
13,4	14,7	0,6	2	2	23	2006
16,1	25,5	0,6	4	2	19	2013
15,6	23,2	0,6	3	2	22	2015
14,2	17,5	0,6	2	2	19	2000
14,2	17,5	0,6	5	2	18	2007
14,2	17,5	0,6	3	2	17	2008
14,2	17,5	0,6	4	2	21	2011
14,2	17,5	0,6	3	2	21	2011
14,2	17,5	0,6	3	2	19	2012
14,2	17,5	0,6	3	2	18	2014
14,2	17,5	0,6	3	2	15	2015
17,2	31,1	0,6	4	2	21	2015
12,2	11,1	0,6	2	2	22	2005
12,2	11,1	0,6	2	2	18	2014
15,3	21,9	0,6	7	2	24	2001
15,3	21,9	0,6	6	2	19	2008
15,3	21,9	0,6	6	2	21	2008
15,3	21,9	0,6	3	2	26	2009
16,2	26	0,6	4	2	17	2002

16,2	26	0,6	4	2	19	2013
19,2	43,3	0,6	4	2	19	2013
13,3	14,4	0,6	2	2	20	1999
13,3	14,4	0,6	2	2	19	2002
13,3	14,4	0,6	3	2	23	2010
13,3	14,4	0,6	3	2	23	2010
13,3	14,4	0,6	2	2	19	2013
14,3	17,9	0,6	3	2	21	2000
14,3	17,9	0,6	4	2	22	2009
14,3	17,9	0,6	3	2	19	2012
19,4	44,7	0,6	6	2	15	2015
13,6	15,4	0,6	3	2	19	2013
15,2	21,5	0,6	7	2	22	2009
15,2	21,5	0,6	4	2	20	2013
17,3	31,7	0,6	8	2	21	2010
17,3	31,7	0,6	8	2	21	2010
14	16,8	0,6	2	2	24	2001
14	16,8	0,6	3	2	23	2003
14	16,8	0,6	2	2	19	2013
14	16,8	0,6	3	2	18	2014
15,5	22,8	0,6	3	2	19	2001
15,7	23,7	0,6	4	2	23	2015
13,8	16,1	0,6	3	2	22	2002
13,8	16,1	0,6	2	2	22	2011
13,8	16,1	0,6	2	2	19	2013
12,3	11,4	0,6	2	2	19	2014
17	30,1	0,6	6	2	19	2011
16	25,1	0,6	7	2	22	2004

16	25,1	0,6	4	2	18	2014
15,1	21,1	0,6	3	2	17	2008
14,4	18,3	0,6	2	2	22	2009
14,4	18,3	0,6	4	2	22	2011
13,2	14,1	0,6	2	2	21	2001
13,2	14,1	0,6	2	2	19	2002
13,2	14,1	0,6	2	2	21	2005
13,2	14,1	0,6	2	2	19	2006
13,2	14,1	0,6	4	2	21	2008
13,2	14,1	0,6	2	2	19	2012
13,2	14,1	0,6	2	2	17	2014
17,7	34	0,6	5	2	19	2010
17,7	34	0,6	5	2	19	2010
18,9	41,4	0,6	6	2	17	2014
15,4	22,4	0,6	3	2	21	2001
15,4	22,4	0,6	3	2	22	2001
15,4	22,4	0,6	3	2	21	2003
15	20,7	0,6	4	2	26	2006
15	20,7	0,6	3	2	20	2007
15	20,7	0,6	5	2	19	2012
15	20,7	0,6	3	2	19	2012
15	20,7	0,6	5	2	21	2014
14,5	18,7	0,6	3	2	17	2008
10,4	6,9	0,6	2	2	20	2004
10,4	6,9	0,6	1	2	26	2009
12	10,6	0,6	2	2	25	2003
12	10,6	0,6	2	2	21	2006
12	10,6	0,6	2	2	21	2006

12	10,6	0,6	2	2	20	2007
12	10,6	0,6	2	2	21	2013
16,1	25,6	0,6	5	2	17	2012
16,1	25,6	0,6	4	2	18	2012
17,8	34,6	0,6	6	2	18	2002
15,8	24,2	0,6	4	2	22	2003
15,8	24,2	0,6	3	2	21	2015
14,1	17,2	0,6	2	2	26	2000
14,1	17,2	0,6	5	2	19	2002
14,1	17,2	0,6	3	2	19	2007
14,1	17,2	0,6	6	2	17	2008
14,1	17,2	0,6	4	2	23	2008
14,1	17,2	0,6	3	2	23	2008
14,1	17,2	0,6	3	2	18	2015
16,8	29,1	0,6	4	2	19	2011
14,6	19,1	0,6	3	2	25	2004
14,6	19,1	0,6	3	2	22	2011
14,6	19,1	0,6	3	2	21	2015
13,5	15,1	0,6	2	2	23	1999
13,5	15,1	0,6	3	2	22	2000
13,5	15,1	0,6	5	2	20	2007
13,5	15,1	0,6	3	2	19	2010
13,5	15,1	0,6	3	2	19	2010
13,5	15,1	0,6	6	2	19	2015
13,1	13,8	0,6	3	2	20	2000
13,1	13,8	0,6	2	2	20	2009
13,1	13,8	0,6	2	2	19	2012
13,1	13,8	0,6	2	2	17	2014

13,1	13,8	0,6	2	2	19	2014
18	35,8	0,6	6	2	21	2014
14,8	19,9	0,6	2	2	23	2003
14,8	19,9	0,6	3	2	21	2014
14,7	19,5	0,6	3	2	21	2015
16,2	26,1	0,6	5	2	23	2008
16,2	26,1	0,6	7	2	19	2012
16,2	26,1	0,6	5	2	19	2013
16,3	26,6	0,6	4	2	18	2015
15,3	22	0,6	4	2	22	1999
15,3	22	0,6	7	2	21	2010
15,3	22	0,6	7	2	21	2010
15,3	22	0,6	3	2	16	2015
16,4	27,1	0,6	8	2	20	2010
16,4	27,1	0,6	8	2	20	2010
13,9	16,5	0,6	3	2	25	2007
13,9	16,5	0,6	3	2	18	2014
12,5	12	0,6	4	2	26	2006
12,5	12	0,6	5	2	20	2007
12,5	12	0,6	2	2	18	2014
16,5	27,6	0,6	4	2	20	2003
16,5	27,6	0,6	6	2	23	2011
13,7	15,8	0,6	2	2	19	1999
13,7	15,8	0,6	3	2	20	2002
13,7	15,8	0,6	2	2	24	2006
13,7	15,8	0,6	3	2	22	2011
13,7	15,8	0,6	3	2	18	2014
13	13,5	0,6	3	2	21	2005

13	13,5	0,6	4	2	25	2005
13	13,5	0,6	2	2	19	2014
17,6	33,5	0,6	5	2	18	2015
20,8	55,3	0,6	5	2	18	2012
14,2	17,6	0,6	7	2	22	2005
14,2	17,6	0,6	3	2	18	2014
17	30,2	0,6	4	2	18	2015
12,6	12,3	0,6	3	2	23	2008
12,9	13,2	0,6	2	2	20	2002
12,9	13,2	0,6	2	2	22	2011
12,9	13,2	0,6	2	2	19	2013
12,9	13,2	0,6	2	2	18	2014
15,5	22,9	0,6	3	2	18	2001
15,5	22,9	0,6	3	2	20	2003
15,5	22,9	0,6	6	2	23	2011
15,5	22,9	0,6	7	2	21	2014
15,5	22,9	0,6	3	2	15	2015
15,7	23,8	0,6	7	2	20	2006
11,6	9,6	0,6	2	2	25	2004
11,6	9,6	0,6	2	2	25	2005
15,2	21,6	0,6	4	2	18	1999
15,2	21,6	0,6	6	2	21	2008
15,2	21,6	0,6	4	2	19	2012
15,2	21,6	0,6	4	2	19	2013
13,4	14,8	0,6	2	2	20	2003
13,4	14,8	0,6	3	2	21	2006
13,4	14,8	0,6	2	2	20	2009
12,7	12,6	0,6	2	2	18	2002

12,7	12,6	0,6	3	2	22	2005
12,7	12,6	0,6	2	2	20	2013
12,8	12,9	0,6	2	2	18	2002
12,8	12,9	0,6	2	2	19	2014
19	42,2	0,6	8	2	21	2010
19	42,2	0,6	8	2	21	2010
12,1	10,9	0,6	2	2	19	2007
12,1	10,9	0,6	2	2	18	2014
18,1	36,5	0,6	5	2	17	2014
14,3	18	0,6	6	2	21	2000
14,3	18	0,6	4	2	23	2006
14,3	18	0,6	2	2	23	2006
14,3	18	0,6	3	2	19	2011
14,3	18	0,6	3	2	18	2014
15,1	21,2	0,6	3	2	16	2015
15,1	21,2	0,6	4	2	22	2015
16,1	25,7	0,6	3	2	19	2012
16,1	25,7	0,6	4	2	18	2014
14	16,9	0,6	4	2	22	1999
14	16,9	0,6	2	2	17	2002
14	16,9	0,6	3	2	23	2010
14	16,9	0,6	3	2	23	2010
14	16,9	0,6	3	2	17	2014
14	16,9	0,6	3	2	19	2015
15,4	22,5	0,6	4	2	24	2001
13,6	15,5	0,6	6	2	21	2011
14,4	18,4	0,6	2	2	17	2002
14,4	18,4	0,6	3	2	23	2003

16,7	28,7	0,6	3	2	21	2015
16,2	26,2	0,6	5	2	18	1999
16,2	26,2	0,6	4	2	20	2003
16,2	26,2	0,6	4	2	22	2003
16,2	26,2	0,6	4	2	16	2008
13,3	14,5	0,6	2	2	19	2002
13,3	14,5	0,6	4	2	18	2015
15,6	23,4	0,6	3	2	18	2000
13,8	16,2	0,6	3	2	25	2004
13,8	16,2	0,6	8	2	19	2010
13,8	16,2	0,6	3	2	23	2010
13,8	16,2	0,6	8	2	19	2010
13,8	16,2	0,6	3	2	23	2010
13,8	16,2	0,6	3	2	23	2011
13,8	16,2	0,6	2	2	17	2014
13,8	16,2	0,6	3	2	17	2014
18,4	38,4	0,6	4	2	15	2015
16,6	28,2	0,6	5	2	22	2003
16,3	26,7	0,6	9	2	22	2004
16,5	27,7	0,6	4	2	18	1999
14,5	18,8	0,6	4	2	20	2001
14,5	18,8	0,6	4	2	18	2015
14,9	20,4	0,6	3	2	25	2003
17	30,3	0,6	5	2	18	2000
17	30,3	0,6	4	2	20	2001
12,2	11,2	0,6	2	2	25	2007
12,2	11,2	0,6	2	2	18	2014
14,8	20	0,6	4	2	24	2006

14,8	20	0,6	4	2	20	2013
14,8	20	0,6	3	2	19	2015
14,7	19,6	0,6	3	2	18	2002
15,3	22,1	0,6	4	2	20	2000
15,3	22,1	0,6	3	2	19	2000
15,3	22,1	0,6	4	2	23	2010
15,3	22,1	0,6	4	2	23	2010
15,3	22,1	0,6	4	2	23	2011
15,3	22,1	0,6	5	2	21	2012
17,8	34,8	0,6	5	2	17	2012
17,2	31,4	0,6	5	2	19	2013
14,1	17,3	0,6	2	2	18	2000
14,1	17,3	0,6	5	2	21	2011
14,1	17,3	0,6	3	2	19	2013
14,1	17,3	0,6	3	2	18	2015
17,9	35,4	0,6	5	2	17	2014
18	36	0,6	6	2	17	2014
16,9	29,8	0,6	4	2	18	2012
13,2	14,2	0,6	2	2	19	2002
13,2	14,2	0,6	3	2	22	2004
13,2	14,2	0,6	3	2	21	2005
13,2	14,2	0,6	4	2	22	2005
13,2	14,2	0,6	5	2	20	2007
13,2	14,2	0,6	3	2	20	2008
15,7	23,9	0,6	4	2	25	2004
15,7	23,9	0,6	5	2	18	2014
15,5	23	0,6	5	2	20	2012
15,5	23	0,6	4	2	21	2012

15,5	23	0,6	3	2	20	2013
15,5	23	0,6	3	2	15	2015
16	25,3	0,6	3	2	21	2003
16	25,3	0,6	5	2	20	2006
16	25,3	0,6	5	2	18	2012
16	25,3	0,6	5	2	21	2014
16	25,3	0,6	5	2	23	2014
13,5	15,2	0,6	2	2	26	2001
13,5	15,2	0,6	2	2	23	2002
13,5	15,2	0,6	2	2	22	2011
13,5	15,2	0,6	2	2	20	2013
13,5	15,2	0,6	2	2	20	2013
13,5	15,2	0,6	2	2	17	2014
15,2	21,7	0,6	4	2	25	2004
15,2	21,7	0,6	3	2	16	2008
15,2	21,7	0,6	4	2	17	2009
15,2	21,7	0,6	4	2	21	2015
12,3	11,5	0,6	3	2	25	2004
12,3	11,5	0,6	4	2	22	2005
12,3	11,5	0,6	3	2	23	2005
18,4	38,5	0,6	6	2	19	2013
17,3	32	0,6	9	2	25	2004
11,5	9,4	0,6	2	2	23	2004
17,6	33,7	0,6	4	2	21	2015
14,2	17,7	0,6	4	2	20	1999
14,2	17,7	0,6	4	2	20	2010
14,2	17,7	0,6	4	2	20	2010
14,2	17,7	0,6	3	2	16	2015

13,1	13,9	0,6	2	2	21	2001
13,1	13,9	0,6	2	2	25	2003
13,1	13,9	0,6	2	2	17	2009
13,1	13,9	0,6	3	2	23	2010
13,1	13,9	0,6	3	2	23	2010
13,1	13,9	0,6	2	2	19	2013
13,7	15,9	0,6	2	2	20	2000
13,7	15,9	0,6	2	2	26	2000
13,7	15,9	0,6	2	2	21	2001
13,7	15,9	0,6	3	2	18	2015
13,7	15,9	0,6	2	2	21	2015
17,7	34,3	0,6	4	2	19	2000
16,2	26,3	0,6	7	2	21	2014
15,1	21,3	0,6	5	2	25	2002
15,1	21,3	0,6	4	2	18	2015
18,2	37,3	0,6	4	2	19	2012
17	30,4	0,6	3	2	19	2013
17	30,4	0,6	5	2	20	2013
15,4	22,6	0,6	3	2	15	2015
16,3	26,8	0,6	6	2	23	2011
16,3	26,8	0,6	4	2	18	2014
16,5	27,8	0,6	5	2	18	2002
16,5	27,8	0,6	5	2	19	2004
12,4	11,8	0,6	2	2	18	2000
12,4	11,8	0,6	2	2	20	2001
16,4	27,3	0,6	5	2	22	2001
16,4	27,3	0,6	4	2	19	2002
16,4	27,3	0,6	6	2	20	2013

14,3	18,1	0,6	5	2	23	1999
14,3	18,1	0,6	4	2	23	2002
14,3	18,1	0,6	3	2	25	2004
14,3	18,1	0,6	3	2	23	2010
14,3	18,1	0,6	3	2	23	2010
14,3	18,1	0,6	4	2	15	2015
15,6	23,5	0,6	5	2	19	2012
13	13,6	0,6	2	2	25	1999
13	13,6	0,6	3	2	23	2005
13	13,6	0,6	4	2	23	2006
13	13,6	0,6	3	2	21	2008
13	13,6	0,6	2	2	17	2014
12	10,7	0,6	2	2	20	2006
12	10,7	0,6	2	2	25	2007
11,2	8,7	0,6	2	2	20	2004
11,2	8,7	0,6	2	2	20	2004
13,4	14,9	0,6	2	2	20	2000
13,4	14,9	0,6	2	2	25	2008
13,4	14,9	0,6	2	2	19	2009
15	20,9	0,6	3	2	19	2007
15	20,9	0,6	4	2	22	2015
15,9	24,9	0,6	3	2	19	2000
16,9	29,9	0,6	7	2	22	1999
22,3	68,7	0,6	10	2	18	2012
12,5	12,1	0,6	3	2	25	2004
12,5	12,1	0,6	3	2	15	2015
14	17	0,6	5	2	18	2007
14	17	0,6	3	2	17	2014

14	17	0,6	3	2	18	2015
12,9	13,3	0,6	2	2	24	2006
12,9	13,3	0,6	2	2	24	2006
12,9	13,3	0,6	3	2	23	2010
12,9	13,3	0,6	3	2	23	2010
14,4	18,5	0,6	5	2	21	1999
14,4	18,5	0,6	5	2	18	2000
14,4	18,5	0,6	2	2	26	2000
14,4	18,5	0,6	3	2	20	2003
14,4	18,5	0,6	3	2	18	2008
14,4	18,5	0,6	4	2	20	2013
14,4	18,5	0,6	3	2	16	2015
14,4	18,5	0,6	3	2	21	2015
14,9	20,5	0,6	3	2	20	2006
14,9	20,5	0,6	4	2	19	2012
14,9	20,5	0,6	3	2	17	2014
15,3	22,2	0,6	4	2	23	1999
15,3	22,2	0,6	4	2	18	2000
15,3	22,2	0,6	5	2	26	2000
15,3	22,2	0,6	4	2	20	2003
15,3	22,2	0,6	6	2	20	2011
15,3	22,2	0,6	4	2	15	2015
15,3	22,2	0,6	4	2	16	2015
12,6	12,4	0,6	2	2	26	1999
12,6	12,4	0,6	2	2	20	2009
12,8	13	0,6	2	2	23	2007
14,5	18,9	0,6	3	2	26	2003
14,5	18,9	0,6	5	2	19	2007

14,5	18,9	0,6	4	2	20	2013
19,1	43,2	0,6	10	2	21	2013
14,8	20,1	0,6	3	2	19	2012
16	25,4	0,6	3	2	18	2000
16	25,4	0,6	4	2	22	2004
14,6	19,3	0,6	3	2	21	2003
14,6	19,3	0,6	3	2	22	2003
14,6	19,3	0,6	4	2	22	2004
14,6	19,3	0,6	4	2	20	2013
13,6	15,6	0,6	5	2	25	2007
13,6	15,6	0,6	2	2	20	2008
13,6	15,6	0,6	6	2	25	2008
13,6	15,6	0,6	2	2	19	2012
15,7	24	0,6	5	2	18	1999
15,7	24	0,6	5	2	26	2006
14,7	19,7	0,6	6	2	26	1999
14,7	19,7	0,6	3	2	26	2003
14,7	19,7	0,6	4	2	20	2004
14,7	19,7	0,6	4	2	20	2009
14,7	19,7	0,6	3	2	20	2013
13,8	16,3	0,6	2	2	18	2002
13,8	16,3	0,6	4	2	17	2009
13,8	16,3	0,6	3	2	22	2011
15,5	23,1	0,6	3	2	23	2003
15,5	23,1	0,6	3	2	20	2011
15,5	23,1	0,6	3	2	18	2014
10,7	7,6	0,6	1	2	23	2009
13,3	14,6	0,6	4	2	23	2009

13,3	14,6	0,6	2	2	26	2009
13,3	14,6	0,6	2	2	19	2013
13,3	14,6	0,6	3	2	19	2014
13,3	14,6	0,6	2	2	21	2014
18	36,2	0,6	6	2	19	2000
14,1	17,4	0,6	3	2	21	2001
14,1	17,4	0,6	3	2	19	2004
14,1	17,4	0,6	5	2	18	2007
14,1	17,4	0,6	4	2	19	2008
14,1	17,4	0,6	3	2	19	2008
14,1	17,4	0,6	3	2	19	2008
14,1	17,4	0,6	3	2	20	2011
14,1	17,4	0,6	3	2	19	2014
15,2	21,8	0,6	4	2	21	2013
15,2	21,8	0,6	7	2	19	2014
11,8	10,2	0,6	2	2	18	2014
16,6	28,4	0,6	8	2	20	2010
16,6	28,4	0,6	8	2	20	2010
12,1	11	0,6	3	2	26	2005
12,1	11	0,6	2	2	23	2011
16,2	26,4	0,6	5	2	20	2011
11,4	9,2	0,6	1	2	26	2001
16,5	27,9	0,6	4	2	18	2000
16,5	27,9	0,6	6	2	26	2001
16,5	27,9	0,6	3	2	19	2013
16,3	26,9	0,6	5	2	20	2004
16,4	27,4	0,6	5	2	20	2000
16,4	27,4	0,6	4	2	22	2004

16,4	27,4	0,6	3	2	16	2015
18,4	38,7	0,6	6	2	19	2013
22,7	72,7	0,6	10	2	18	2012
15,1	21,4	0,6	5	2	19	1999
15,1	21,4	0,6	7	2	22	2001
15,1	21,4	0,6	3	2	17	2002
15,6	23,6	0,6	5	2	20	2001
15,6	23,6	0,6	4	2	19	2004
15,6	23,6	0,6	5	2	20	2007
14,2	17,8	0,6	3	2	21	2005
14,2	17,8	0,6	3	2	19	2007
14,2	17,8	0,6	4	2	23	2011
14,2	17,8	0,6	3	2	17	2014
14,2	17,8	0,6	3	2	17	2014
13,2	14,3	0,6	2	2	18	2002
13,2	14,3	0,6	2	2	20	2013
13,2	14,3	0,6	2	2	22	2013
13,2	14,3	0,6	2	2	21	2014
13,2	14,3	0,6	2	2	23	2014
13,9	16,7	0,6	3	2	20	2002
13,9	16,7	0,6	3	2	18	2008
13,9	16,7	0,6	3	2	19	2012
13,9	16,7	0,6	3	2	23	2015
13,5	15,3	0,6	2	2	18	2002
13,5	15,3	0,6	3	2	20	2008
18,2	37,5	0,6	7	2	17	2014
18,2	37,5	0,6	4	2	18	2015
15	21	0,6	6	2	19	2001

15	21	0,6	6	2	19	2013
13,7	16	0,6	3	2	22	1999
13,7	16	0,6	2	2	24	2001
13,7	16	0,6	6	2	22	2005
13,7	16	0,6	2	2	22	2009
13,7	16	0,6	8	2	22	2011
18,1	36,9	0,6	7	2	19	2012
12,2	11,3	0,6	2	2	20	2002
12,2	11,3	0,6	3	2	22	2005
12,2	11,3	0,6	2	2	22	2011
12,2	11,3	0,6	2	2	19	2014
14,3	18,2	0,6	4	2	26	1999
14,3	18,2	0,6	3	2	23	2003
14,3	18,2	0,6	5	2	19	2007
14,3	18,2	0,6	6	2	20	2007
14,3	18,2	0,6	3	2	18	2015
19	42,7	0,6	5	2	19	2012
16	25,5	0,6	4	2	21	2001
16	25,5	0,6	5	2	20	2007
16	25,5	0,6	4	2	20	2012
15,3	22,3	0,6	3	2	22	2003
14,9	20,6	0,6	4	2	20	1999
14,9	20,6	0,6	4	2	26	2006
14,9	20,6	0,6	4	2	19	2011
14,9	20,6	0,6	3	2	21	2012
13,1	14	0,6	5	2	20	2000
13,1	14	0,6	3	2	25	2005
13,1	14	0,6	3	2	19	2013

13,1	14	0,6	2	2	19	2014
13,1	14	0,6	2	2	21	2015
15,7	24,1	0,6	5	2	22	1999
15,7	24,1	0,6	6	2	22	2000
15,7	24,1	0,6	3	2	20	2003
17	30,6	0,6	3	2	15	2015
14,4	18,6	0,6	3	2	20	2000
14,4	18,6	0,6	4	2	24	2006
14,4	18,6	0,6	3	2	15	2015
16,1	26	0,6	5	2	19	2014
14,8	20,2	0,6	3	2	21	2000
14,8	20,2	0,6	4	2	23	2004
14	17,1	0,6	3	2	22	2000
14	17,1	0,6	2	2	18	2002
14	17,1	0,6	4	2	20	2008
14	17,1	0,6	3	2	20	2011
14	17,1	0,6	3	2	21	2014
14	17,1	0,6	3	2	23	2014
14,5	19	0,6	5	2	22	1999
14,5	19	0,6	4	2	21	2011
14,5	19	0,6	3	2	20	2013
14,5	19	0,6	4	2	18	2015
16,2	26,5	0,6	4	2	20	2000
16,2	26,5	0,6	5	2	18	2012
16,5	28	0,6	4	2	19	2001
14,7	19,8	0,6	4	2	20	1999
14,7	19,8	0,6	3	2	21	2000
14,7	19,8	0,6	2	2	24	2001

14,7	19,8	0,6	3	2	16	2015
12,3	11,6	0,6	2	2	20	2004
12,3	11,6	0,6	2	2	20	2013
14,6	19,4	0,6	3	2	23	2001
13,4	15	0,6	2	2	19	2000
13,4	15	0,6	2	2	26	2000
13,4	15	0,6	2	2	20	2002
13,4	15	0,6	4	2	20	2009
13,4	15	0,6	2	2	22	2009
16,3	27	0,6	4	2	19	2013
16,3	27	0,6	4	2	19	2015
16,4	27,5	0,6	5	2	20	1999
16,4	27,5	0,6	7	2	18	2014
13	13,7	0,6	2	2	19	2002
13	13,7	0,6	3	2	21	2008
13	13,7	0,6	4	2	26	2009
13	13,7	0,6	2	2	19	2013
13	13,7	0,6	2	2	19	2013
13	13,7	0,6	2	2	19	2013
13	13,7	0,6	2	2	19	2013
13	13,7	0,6	2	2	18	2014
15,2	21,9	0,6	5	2	21	2000
15,2	21,9	0,6	4	2	18	2015
15,8	24,6	0,6	4	2	22	2004
15,8	24,6	0,6	4	2	18	2012
15,8	24,6	0,6	4	2	20	2013
11,3	9	0,6	2	2	18	2007
17,3	32,3	0,6	4	2	20	2003

17,3	32,3	0,6	3	2	22	2003
17,3	32,3	0,6	4	2	17	2012
18,1	37	0,6	10	2	17	2008
17,1	31,2	0,6	4	2	18	2012
13,8	16,4	0,6	3	2	25	2008
13,8	16,4	0,6	3	2	15	2015
12,4	11,9	0,6	2	2	19	2000
12,4	11,9	0,6	2	2	20	2002
12,4	11,9	0,6	2	2	20	2002
12,4	11,9	0,6	2	2	15	2015
13,6	15,7	0,6	2	2	23	2002
13,6	15,7	0,6	2	2	25	2003
13,6	15,7	0,6	3	2	21	2011
13,6	15,7	0,6	3	2	18	2014
18	36,4	0,6	5	2	19	2013
17,9	35,8	0,6	9	2	20	2011
12,9	13,4	0,6	3	2	18	2014
16,8	29,6	0,6	4	2	19	2013
15,4	22,8	0,6	6	2	23	2011
15,6	23,7	0,6	5	2	21	2009
14,1	17,5	0,6	5	2	21	2000
14,1	17,5	0,6	2	2	23	2001
14,1	17,5	0,6	2	2	26	2003
14,1	17,5	0,6	2	2	20	2013
20,1	50,7	0,6	7	2	17	2014
11,7	10	0,6	2	2	20	2004
11,7	10	0,6	2	2	23	2004
15,9	25,1	0,6	4	2	26	2003

12,5	12,2	0,6	3	2	22	2005
12,5	12,2	0,6	2	2	25	2007
12,5	12,2	0,6	2	2	19	2009
12,5	12,2	0,6	2	2	26	2009
12,8	13,1	0,6	2	2	24	2006
12,8	13,1	0,6	2	2	23	2011
12,8	13,1	0,6	2	2	19	2013
13,3	14,7	0,6	2	2	19	2002
13,3	14,7	0,6	2	2	20	2002
13,3	14,7	0,6	3	2	23	2005
13,3	14,7	0,6	3	2	25	2008
13,3	14,7	0,6	2	2	19	2013
13,3	14,7	0,6	2	2	18	2014
12,6	12,5	0,6	2	2	20	2004
12,6	12,5	0,6	2	2	23	2006
12,6	12,5	0,6	2	2	19	2013
12,7	12,8	0,6	3	2	22	2005
12,7	12,8	0,6	3	2	23	2010
12,7	12,8	0,6	3	2	23	2010
12,7	12,8	0,6	2	2	19	2014
12,5	12,21	0,6	2	2	23	2010
12,5	12,21	0,6	2	2	23	2010
14,2	17,9	0,6	4	2	22	2001
14,2	17,9	0,6	3	2	19	2007
14,2	17,9	0,6	8	2	24	2009
15	21,1	0,6	6	2	24	2001
15	21,1	0,6	3	2	20	2002
15	21,1	0,6	6	2	18	2008

15	21,1	0,6	5	2	19	2014
15,7	24,2	0,6	4	2	19	2008
15,7	24,2	0,6	4	2	23	2011
15,3	22,4	0,6	3	2	21	2003
15,3	22,4	0,6	7	2	26	2009
15,3	22,4	0,6	4	2	20	2011
15,3	22,4	0,6	4	2	18	2014
16,5	28,1	0,6	7	2	21	1999
16,5	28,1	0,6	6	2	19	2001
13,9	16,8	0,6	3	2	25	2007
15,5	23,3	0,6	7	2	21	2012
16,3	27,1	0,6	5	2	23	2002
17,3	32,4	0,6	4	2	17	2014
17,3	32,4	0,6	5	2	19	2014
14,9	20,7	0,6	5	2	19	2007
14,3	18,3	0,6	3	2	23	2003
14,3	18,3	0,6	4	2	20	2006
14,3	18,3	0,6	4	2	20	2009
14,3	18,3	0,6	4	2	19	2014
17,8	35,3	0,6	4	2	15	2015
13,5	15,4	0,6	2	2	25	2005
13,2	14,4	0,6	2	2	26	1999
13,2	14,4	0,6	3	2	22	2005
13,2	14,4	0,6	3	2	18	2007
13,2	14,4	0,6	2	2	19	2014
13,7	16,1	0,6	3	2	23	1999
13,7	16,1	0,6	2	2	19	2000
13,7	16,1	0,6	3	2	23	2008

14,8	20,3	0,6	4	2	18	2000
14,8	20,3	0,6	6	2	20	2000
14,8	20,3	0,6	3	2	19	2002
14,8	20,3	0,6	3	2	24	2002
15,8	24,7	0,6	3	2	23	2003
15,8	24,7	0,6	4	2	26	2003
14,4	18,7	0,6	4	2	21	1999
14,4	18,7	0,6	4	2	22	1999
14,4	18,7	0,6	4	2	23	1999
14,4	18,7	0,6	3	2	18	2000
14,4	18,7	0,6	2	2	19	2000
11,2	8,8	0,6	2	2	25	2004
15,2	22	0,6	3	2	21	2001
15,2	22	0,6	4	2	25	2003
15,2	22	0,6	4	2	19	2007
15,2	22	0,6	6	2	22	2011
15,2	22	0,6	4	2	20	2013
14,7	19,9	0,6	3	2	23	2003
14,7	19,9	0,6	3	2	16	2015
14,5	19,1	0,6	3	2	18	2000
14,5	19,1	0,6	4	2	23	2002
14,5	19,1	0,6	4	2	19	2012
12,1	11,1	0,6	3	2	21	2005
12,1	11,1	0,6	2	2	26	2006
12,1	11,1	0,6	2	2	23	2007
12,1	11,1	0,6	2	2	23	2007
12,1	11,1	0,6	2	2	23	2007
12,1	11,1	0,6	2	2	23	2010

12,1	11,1	0,6	2	2	23	2010
14,6	19,5	0,6	3	2	18	2000
14,6	19,5	0,6	3	2	18	2000
14	17,2	0,6	3	2	18	1999
14	17,2	0,6	4	2	25	1999
14	17,2	0,6	3	2	23	2007
14	17,2	0,6	3	2	21	2014
14	17,2	0,6	3	2	23	2014
11,8	10,3	0,6	2	2	20	2002
11,8	10,3	0,6	2	2	20	2004
15,6	23,8	0,6	4	2	25	2004
17,2	31,9	0,6	10	2	20	2011
19	43	0,6	7	2	18	2012
15,9	25,2	0,6	5	2	21	1999
17,5	33,6	0,6	4	2	21	2015
17,5	33,6	0,6	6	2	22	2015
15,4	22,9	0,6	3	2	21	2001
15,4	22,9	0,6	3	2	19	2012
15,4	22,9	0,6	3	2	17	2014
13,1	14,1	0,6	3	2	21	2005
13,1	14,1	0,6	2	2	26	2009
13,1	14,1	0,6	2	2	22	2011
13,1	14,1	0,6	4	2	18	2015
16	25,7	0,6	5	2	21	1999
16	25,7	0,6	4	2	19	2013
13,4	15,1	0,6	2	2	22	2000
13,4	15,1	0,6	2	2	23	2001
13,4	15,1	0,6	3	2	21	2005

16,5	28,2	0,6	8	2	21	2015
12,2	11,4	0,6	2	2	19	2014
12,2	11,4	0,6	2	2	21	2014
13,8	16,5	0,6	3	2	23	2010
13,8	16,5	0,6	3	2	23	2010
11,6	9,8	0,6	2	2	20	2004
11,6	9,8	0,6	2	2	25	2004
11,6	9,8	0,6	2	2	18	2007
14,1	17,6	0,6	2	2	21	2000
14,1	17,6	0,6	4	2	24	2009
14,1	17,6	0,6	3	2	18	2014
17,1	31,4	0,6	4	2	19	2012
17,1	31,4	0,6	4	2	16	2015
16,4	27,7	0,6	7	2	21	2001
16,2	26,7	0,6	5	2	19	2000
18,9	42,4	0,6	9	2	19	2012
13,6	15,8	0,6	3	2	21	2008
13,6	15,8	0,6	3	2	19	2015
13	13,8	0,6	3	2	21	2005
13	13,8	0,6	2	2	23	2006
13	13,8	0,6	3	2	23	2007
13	13,8	0,6	3	2	23	2007
13	13,8	0,6	3	2	21	2008
13	13,8	0,6	2	2	22	2009
13	13,8	0,6	2	2	21	2014
13	13,8	0,6	2	2	23	2014
15	21,2	0,6	6	2	22	2000
15	21,2	0,6	5	2	19	2000

15	21,2	0,6	6	2	19	2001
15	21,2	0,6	5	2	22	2007
15,3	22,5	0,6	2	2	24	2001
15,3	22,5	0,6	3	2	21	2003
15,3	22,5	0,6	4	2	19	2012
15,3	22,5	0,6	5	2	21	2014
15,3	22,5	0,6	3	2	18	2015
19	43,1	0,6	5	2	19	2012
15,5	23,4	0,6	3	2	18	2014
15,5	23,4	0,6	5	2	19	2014
16,8	29,8	0,6	5	2	20	2011
16,8	29,8	0,6	3	2	21	2014
14,2	18	0,6	4	2	23	2006
14,2	18	0,6	3	2	23	2011
14,2	18	0,6	3	2	18	2015
14,2	18	0,6	3	2	22	2015
15,8	24,8	0,6	5	2	23	2010
15,8	24,8	0,6	5	2	23	2010
11,1	8,6	0,6	2	2	25	2004
17,2	32	0,6	8	2	20	2013
17	30,9	0,6	9	2	24	2001
17	30,9	0,6	9	2	21	2014
13,3	14,8	0,6	2	2	24	2001
13,3	14,8	0,6	2	2	18	2002
13,3	14,8	0,6	3	2	23	2010
13,3	14,8	0,6	3	2	23	2010
13,3	14,8	0,6	2	2	22	2011
13,3	14,8	0,6	2	2	21	2014

16,7	29,3	0,6	8	2	23	2002
17,6	34,3	0,6	6	2	20	2010
17,6	34,3	0,6	6	2	20	2010
14,3	18,4	0,6	3	2	22	2001
14,3	18,4	0,6	3	2	24	2001
14,3	18,4	0,6	5	2	22	2004
14,3	18,4	0,6	4	2	23	2011
14,3	18,4	0,6	4	2	19	2013
14,3	18,4	0,6	3	2	17	2014
14,3	18,4	0,6	3	2	18	2015
14,3	18,4	0,6	3	2	21	2015
13,9	16,9	0,6	3	2	21	2008
14,8	20,4	0,6	4	2	15	2015
15,2	22,1	0,6	5	2	21	2000
15,2	22,1	0,6	3	2	20	2012
15,2	22,1	0,6	3	2	18	2015
12,4	12	0,6	3	2	21	2001
12,4	12	0,6	2	2	26	2001
12,4	12	0,6	2	2	24	2006
12,4	12	0,6	2	2	26	2009
12,8	13,2	0,6	2	2	25	2007
12,8	13,2	0,6	2	2	19	2013
15,6	23,9	0,6	6	2	19	2011
14,4	18,8	0,6	4	2	19	2011
14,4	18,8	0,6	3	2	17	2014
14,4	18,8	0,6	3	2	19	2014
14,7	20	0,6	3	2	18	2001
14,7	20	0,6	3	2	20	2004

17,3	32,6	0,6	3	2	17	2014
10,6	7,5	0,6	2	2	25	2004
15,4	23	0,6	4	2	18	2001
15,4	23	0,6	3	2	17	2014
12,5	12,3	0,6	2	2	18	2014
12,5	12,3	0,6	3	2	19	2014
12,7	12,9	0,6	4	2	19	2008
12,7	12,9	0,6	2	2	21	2011
14,5	19,2	0,6	3	2	18	1999
14,5	19,2	0,6	3	2	21	2003
14,6	19,6	0,6	5	2	24	2009
14,6	19,6	0,6	3	2	18	2014
19	43,2	0,6	8	2	22	2013
19	43,2	0,6	5	2	17	2014
16	25,8	0,6	6	2	18	2001
13,5	15,5	0,6	2	2	20	2003
13,5	15,5	0,6	3	2	17	2014
16,5	28,3	0,6	4	2	19	2013
16,5	28,3	0,6	4	2	15	2015
10	6,3	0,6	1	2	20	2004
13,7	16,2	0,6	4	2	20	2009
13,7	16,2	0,6	3	2	15	2015
16,4	27,8	0,6	5	2	20	2000
16,4	27,8	0,6	4	2	24	2001
15,1	21,7	0,6	3	2	18	2001
15,1	21,7	0,6	4	2	20	2002
15,1	21,7	0,6	4	2	22	2004
15,1	21,7	0,6	4	2	25	2004

15,1	21,7	0,6	4	2	16	2008
18,2	38	0,6	4	2	17	2012
16,2	26,8	0,6	6	2	18	2007
16,2	26,8	0,6	7	2	17	2014
13,2	14,5	0,6	2	2	22	2000
13,2	14,5	0,6	2	2	19	2013
14	17,3	0,6	6	2	18	2000
14	17,3	0,6	3	2	19	2012
14	17,3	0,6	2	2	19	2013
11,3	9,1	0,6	2	2	22	2004
12	10,9	0,6	2	2	23	2004
12	10,9	0,6	2	2	21	2006
12	10,9	0,6	2	2	23	2007
12	10,9	0,6	2	2	23	2007
12	10,9	0,6	2	2	20	2009
12	10,9	0,6	2	2	22	2011
12	10,9	0,6	2	2	17	2014
12	10,9	0,6	2	2	19	2014
17,2	32,1	0,6	4	2	18	2015
17	31	0,6	6	2	22	2001
17	31	0,6	4	2	19	2012
17	31	0,6	7	2	19	2012
17	31	0,6	7	2	21	2014
15,3	22,6	0,6	4	2	19	1999
15,3	22,6	0,6	3	2	23	2003
15,5	23,5	0,6	5	2	18	1999
15,5	23,5	0,6	3	2	15	2015
11	8,4	0,6	2	2	23	2004

15	21,3	0,6	5	2	20	2007
15	21,3	0,6	4	2	19	2009
15	21,3	0,6	4	2	19	2013
11,5	9,6	0,6	1	2	26	1999
11,5	9,6	0,6	1	2	26	2001
19,4	46,1	0,6	5	2	23	2010
19,4	46,1	0,6	5	2	23	2010
14,1	17,7	0,6	3	2	21	2008
13,8	16,6	0,6	3	2	25	2004
13,8	16,6	0,6	4	2	26	2006
13,8	16,6	0,6	3	2	18	2007
13,1	14,2	0,6	3	2	23	2011
13,1	14,2	0,6	2	2	19	2013
13,4	15,2	0,6	2	2	26	2001
13,4	15,2	0,6	2	2	25	2003
14,9	20,9	0,6	3	2	21	2001
14,9	20,9	0,6	6	2	17	2008
17,1	31,6	0,6	4	2	19	2000
13,6	15,9	0,6	3	2	22	2000
13,6	15,9	0,6	3	2	21	2011
14,2	18,1	0,6	4	2	26	2006
14,2	18,1	0,6	3	2	19	2014
14,2	18,1	0,6	3	2	15	2015
15,2	22,2	0,6	4	2	22	2004
15,2	22,2	0,6	4	2	20	2009
15,2	22,2	0,6	7	2	18	2015
15,6	24	0,6	3	2	18	2000
12,1	11,2	0,6	2	2	23	1999

12,1	11,2	0,6	2	2	22	2007
12,1	11,2	0,6	2	2	20	2009
12,1	11,2	0,6	2	2	19	2014
16	25,9	0,6	5	2	22	2004
18,1	37,5	0,6	8	2	23	2002
15,4	23,1	0,6	5	2	21	1999
15,4	23,1	0,6	4	2	19	2001
15,4	23,1	0,6	4	2	23	2002
15,4	23,1	0,6	3	2	25	2003
16,1	26,4	0,6	3	2	20	2003
16,1	26,4	0,6	5	2	17	2014
14,3	18,5	0,6	3	2	21	2000
14,3	18,5	0,6	4	2	22	2011
13	13,9	0,6	3	2	21	2005
13	13,9	0,6	3	2	23	2007
13	13,9	0,6	3	2	25	2007
13	13,9	0,6	2	2	19	2013
16,3	27,4	0,6	5	2	18	1999
16,3	27,4	0,6	7	2	24	2001
16,2	26,9	0,6	4	2	22	2001
16,2	26,9	0,6	3	2	22	2001
16,2	26,9	0,6	4	2	18	2002
16,2	26,9	0,6	3	2	16	2015
14,7	20,1	0,6	3	2	20	1999
14,7	20,1	0,6	7	2	23	2004
14,4	18,9	0,6	4	2	25	2008
11,8	10,4	0,6	2	2	20	2004
13,9	17	0,6	5	2	25	2007

13,9	17	0,6	3	2	21	2012
13,9	17	0,6	3	2	21	2014
14,6	19,7	0,6	4	2	19	2000
14,6	19,7	0,6	3	2	20	2003
14,6	19,7	0,6	4	2	24	2009
14,5	19,3	0,6	5	2	22	2000
14,5	19,3	0,6	3	2	20	2007
14,5	19,3	0,6	4	2	15	2015
15,7	24,5	0,6	5	2	22	2005
15,1	21,8	0,6	3	2	19	2001
15,1	21,8	0,6	4	2	23	2011
15,1	21,8	0,6	5	2	19	2013
13,3	14,9	0,6	2	2	23	2002
13,3	14,9	0,6	3	2	19	2008
21,1	59,5	0,6	6	2	17	2012
15,5	23,6	0,6	3	2	20	2003
15,5	23,6	0,6	6	2	22	2011
15,5	23,6	0,6	3	2	19	2015
15,5	23,6	0,6	3	2	19	2015
15,3	22,7	0,6	4	2	19	2008
15,3	22,7	0,6	4	2	23	2011
15,8	25	0,6	5	2	20	2010
15,8	25	0,6	5	2	20	2010
15,8	25	0,6	5	2	19	2012
15,8	25	0,6	7	2	18	2012
13,7	16,3	0,6	2	2	22	2000
13,7	16,3	0,6	3	2	25	2007
13,7	16,3	0,6	3	2	15	2015

13,5	15,6	0,6	2	2	17	2002
13,5	15,6	0,6	4	2	21	2006
13,5	15,6	0,6	2	2	19	2009
13,5	15,6	0,6	3	2	20	2009
13,5	15,6	0,6	3	2	23	2009
13,5	15,6	0,6	2	2	19	2013
15	21,4	0,6	4	2	22	1999
15	21,4	0,6	4	2	26	1999
15	21,4	0,6	3	2	18	2001
15	21,4	0,6	4	2	23	2004
15	21,4	0,6	4	2	24	2006
15	21,4	0,6	3	2	19	2012
15	21,4	0,6	4	2	19	2014
15	21,4	0,6	4	2	15	2015
14	17,4	0,6	2	2	20	2000
14	17,4	0,6	2	2	24	2002
14	17,4	0,6	3	2	21	2008
14	17,4	0,6	2	2	19	2014
14	17,4	0,6	3	2	19	2015
14	17,4	0,6	3	2	23	2015
12,3	11,8	0,6	2	2	19	2002
12,3	11,8	0,6	2	2	26	2006
12,3	11,8	0,6	2	2	21	2009
12,8	13,3	0,6	2	2	23	2006
12,8	13,3	0,6	2	2	26	2006
12,8	13,3	0,6	2	2	19	2010
12,8	13,3	0,6	2	2	19	2010
11,6	9,9	0,6	2	2	21	2006

19,7	48,5	0,6	6	2	19	2015
18	37	0,6	5	2	19	2013
11,4	9,4	0,6	2	2	22	2004
11,4	9,4	0,6	2	2	18	2007
12,4	12,1	0,6	2	2	23	1999
12,4	12,1	0,6	3	2	25	2004
12,4	12,1	0,6	2	2	19	2014
12,7	13	0,6	2	2	20	2013
12,7	13	0,6	2	2	18	2014
16	26	0,6	5	2	22	1999
16	26	0,6	4	2	17	2009
17,2	32,3	0,6	6	2	21	2013
16,4	28	0,6	3	2	20	2003
13,2	14,6	0,6	3	2	26	2003
13,2	14,6	0,6	3	2	21	2008
13,2	14,6	0,6	2	2	23	2010
13,2	14,6	0,6	2	2	23	2010
13,2	14,6	0,6	2	2	19	2013
13,2	14,6	0,6	2	2	17	2014
15,6	24,1	0,6	3	2	20	2003
14,9	21	0,6	5	2	22	1999
14,9	21	0,6	7	2	22	2007
12,5	12,4	0,6	2	2	19	2014
12,6	12,7	0,6	2	2	25	1999
12,6	12,7	0,6	3	2	22	2005
12,6	12,7	0,6	2	2	24	2006
12,6	12,7	0,6	2	2	21	2011
11,9	10,7	0,6	2	2	25	2007

14,1	17,8	0,6	3	2	25	2007
16,1	26,5	0,6	4	2	19	2012
15,2	22,3	0,6	3	2	18	2000
15,2	22,3	0,6	4	2	19	2011
15,2	22,3	0,6	4	2	19	2012
15,2	22,3	0,6	3	2	20	2012
16,2	27	0,6	9	2	23	2004
16,2	27	0,6	6	2	20	2013
15,4	23,2	0,6	3	2	21	2003
13,8	16,7	0,6	4	2	24	2006
13,8	16,7	0,6	3	2	21	2011
13,8	16,7	0,6	3	2	18	2014
14,8	20,6	0,6	4	2	20	2001
14,2	18,2	0,6	5	2	23	1999
14,2	18,2	0,6	3	2	21	2001
14,2	18,2	0,6	4	2	23	2005
14,2	18,2	0,6	4	2	20	2012
14,2	18,2	0,6	3	2	19	2012
14,2	18,2	0,6	3	2	18	2014
14,2	18,2	0,6	3	2	15	2015
13,4	15,3	0,6	3	2	25	2004
13,4	15,3	0,6	3	2	22	2011
17,1	31,8	0,6	7	2	18	2012
17,1	31,8	0,6	3	2	21	2013
13,6	16	0,6	2	2	18	2000
13,6	16	0,6	3	2	20	2002
14,3	18,6	0,6	3	2	26	2003
14,3	18,6	0,6	4	2	20	2011

14,3	18,6	0,6	3	2	15	2015
14,3	18,6	0,6	4	2	15	2015
15,1	21,9	0,6	5	2	21	1999
15,1	21,9	0,6	6	2	23	2006
15,1	21,9	0,6	3	2	18	2012
13,1	14,3	0,6	3	2	22	2005
13,1	14,3	0,6	3	2	25	2007
13,1	14,3	0,6	4	2	25	2007
13,1	14,3	0,6	3	2	20	2013
11,1	8,7	0,6	2	2	23	2004
16,6	29,1	0,6	4	2	21	2015
14,6	19,8	0,6	3	2	21	2008
14,4	19	0,6	3	2	22	2000
14,4	19	0,6	2	2	22	2001
14,4	19	0,6	4	2	24	2009
14,5	19,4	0,6	3	2	25	2003
15,8	25,1	0,6	5	2	20	1999
12	11	0,6	2	2	22	2007
12	11	0,6	2	2	23	2007
12	11	0,6	1	2	19	2014
15,3	22,8	0,6	5	2	18	1999
15,3	22,8	0,6	5	2	21	1999
15,3	22,8	0,6	4	2	24	2001
15,3	22,8	0,6	4	2	23	2011
15,3	22,8	0,6	4	2	19	2013
15,3	22,8	0,6	3	2	21	2015
16,5	28,6	0,6	4	2	19	2012
13,9	17,1	0,6	3	2	20	2008

11,7	10,2	0,6	2	2	21	2006
15	21,5	0,6	5	2	19	2000
15	21,5	0,6	4	2	26	2001
16,4	28,1	0,6	4	2	19	2000
16,4	28,1	0,6	5	2	20	2000
19	43,7	0,6	10+	2	17	2014
16	26,1	0,6	5	2	18	1999
19,1	44,4	0,6	7	2	17	2012
13	14	0,6	3	2	22	2000
13	14	0,6	2	2	25	2003
13	14	0,6	3	2	23	2008
13	14	0,6	2	2	26	2009
13	14	0,6	2	2	19	2013
13	14	0,6	2	2	17	2014
13	14	0,6	2	2	18	2014
16,3	27,6	0,6	4	2	24	2002
16,3	27,6	0,6	8	2	23	2010
16,3	27,6	0,6	8	2	23	2010
16,1	26,6	0,6	5	2	19	2000
16,1	26,6	0,6	3	2	21	2015
16,2	27,1	0,6	9	2	19	2004
16,2	27,1	0,6	4	2	19	2013
15,6	24,2	0,6	4	2	19	2014
18,1	37,8	0,6	4	2	18	2012
13,3	15	0,6	2	2	25	1999
13,3	15	0,6	2	2	19	2002
13,3	15	0,6	3	2	25	2004
13,3	15	0,6	3	2	23	2007

13,3	15	0,6	2	2	23	2010
13,3	15	0,6	2	2	23	2010
13,3	15	0,6	2	2	21	2014
11,3	9,2	0,6	2	2	23	2004
16,7	29,7	0,6	5	2	20	2003
14	17,5	0,6	3	2	19	2001
14	17,5	0,6	7	2	19	2007
14	17,5	0,6	5	2	19	2007
13,7	16,4	0,6	5	2	26	1999
13,7	16,4	0,6	3	2	24	2009
13,7	16,4	0,6	4	2	23	2010
13,7	16,4	0,6	4	2	23	2010
13,7	16,4	0,6	3	2	15	2015
13,7	16,4	0,6	3	2	22	2015
15,2	22,4	0,6	3	2	18	2000
15,2	22,4	0,6	4	2	25	2005
15,2	22,4	0,6	5	2	20	2012
15,2	22,4	0,6	3	2	15	2015
12,1	11,3	0,6	2	2	20	2004
12,1	11,3	0,6	2	2	22	2011
14,9	21,1	0,6	6	2	19	2007
15,4	23,3	0,6	4	2	23	1999
15,4	23,3	0,6	4	2	22	2004
17,1	31,9	0,6	5	2	19	2014
13,5	15,7	0,6	4	2	22	1999
12,9	13,7	0,6	2	2	19	2002
12,9	13,7	0,6	2	2	17	2014
15,7	24,7	0,6	6	2	22	2011

15,7	24,7	0,6	4	2	19	2015
19,7	48,8	0,6	5	2	19	2013
16,6	29,2	0,6	5	2	19	2012
14,8	20,7	0,6	5	2	19	2007
14,8	20,7	0,6	4	2	19	2008
14,1	17,9	0,6	3	2	20	2008
14,1	17,9	0,6	4	2	19	2011
14,1	17,9	0,6	3	2	15	2015
11	8,5	0,6	2	2	23	2004
19,1	44,5	0,6	7	2	19	2012
17,2	32,5	0,6	5	2	17	2012
12,2	11,6	0,6	2	2	18	2014
15,8	25,2	0,6	3	2	19	2014
16,5	28,7	0,6	7	2	23	2004
16,5	28,7	0,6	5	2	19	2010
16,5	28,7	0,6	5	2	19	2010
12,8	13,4	0,6	2	2	23	2006
12,8	13,4	0,6	3	2	23	2007
12,8	13,4	0,6	3	2	19	2009
12,8	13,4	0,6	2	2	20	2013
12,8	13,4	0,6	2	2	17	2014
15,1	22	0,6	4	2	21	1999
15,1	22	0,6	4	2	20	2000
15,1	22	0,6	4	2	20	2003
15,1	22	0,6	3	2	21	2003
15,1	22	0,6	6	2	20	2011
15,1	22	0,6	4	2	19	2013
16,8	30,3	0,6	5	2	19	2013

11,8	10,5	0,6	3	2	20	2004
14,7	20,3	0,6	3	2	21	2003
15,5	23,8	0,6	3	2	22	2001
15,5	23,8	0,6	3	2	20	2003
14,2	18,3	0,6	3	2	20	2006
14,2	18,3	0,6	6	2	26	2006
14,2	18,3	0,6	3	2	21	2011
14,2	18,3	0,6	6	2	19	2013
13,2	14,7	0,6	2	2	20	2002
13,2	14,7	0,6	4	2	26	2006
13,2	14,7	0,6	3	2	20	2009
13,2	14,7	0,6	2	2	17	2014
13,8	16,8	0,6	2	2	26	2001
13,8	16,8	0,6	2	2	21	2015
16,4	28,2	0,6	3	2	19	2012
15,9	25,7	0,6	4	2	18	1999
15,9	25,7	0,6	4	2	22	2013
15,3	22,9	0,6	3	2	18	2000
15,3	22,9	0,6	3	2	22	2003
15,3	22,9	0,6	4	2	24	2006
15,3	22,9	0,6	5	2	18	2007
15,3	22,9	0,6	4	2	15	2015
14,6	19,9	0,6	4	2	20	2004
14,6	19,9	0,6	3	2	21	2014
12,3	11,9	0,6	2	2	19	2002
12,3	11,9	0,6	2	2	22	2007
12,3	11,9	0,6	2	2	23	2007
14,3	18,7	0,6	4	2	20	2006

14,3	18,7	0,6	4	2	23	2008
14,3	18,7	0,6	4	2	20	2011
14,3	18,7	0,6	3	2	15	2015
14,3	18,7	0,6	3	2	21	2015
12,7	13,1	0,6	2	2	18	2000
12,7	13,1	0,6	2	2	22	2009
16,3	27,7	0,6	7	2	23	1999
14,5	19,5	0,6	4	2	27	1999
14,5	19,5	0,6	2	2	20	2000
14,5	19,5	0,6	3	2	19	2000
14,5	19,5	0,6	3	2	19	2014
14,5	19,5	0,6	4	2	16	2015
14,5	19,5	0,6	4	2	21	2015
16	26,2	0,6	5	2	19	2000
16	26,2	0,6	6	2	19	2011
14,4	19,1	0,6	5	2	23	1999
14,4	19,1	0,6	3	2	22	2001
14,4	19,1	0,6	3	2	23	2003
14,4	19,1	0,6	6	2	23	2008
16,1	26,7	0,6	5	2	19	2013
16,1	26,7	0,6	7	2	22	2013
12,4	12,2	0,6	2	2	24	2006
12,4	12,2	0,6	2	2	23	2011
12,4	12,2	0,6	2	2	23	2011
12,4	12,2	0,6	2	2	18	2014
12,6	12,8	0,6	2	2	18	2000
12,6	12,8	0,6	3	2	19	2006
17,9	36,7	0,6	6	2	18	2012

17,1	32	0,6	5	2	18	2000
15	21,6	0,6	5	2	23	1999
15	21,6	0,6	4	2	27	1999
12,5	12,5	0,6	2	2	20	2002
12,5	12,5	0,6	3	2	22	2005
12,5	12,5	0,6	2	2	25	2007
15	21,6	0,6	5	2	19	2007
15	21,6	0,6	3	2	18	2012
15	21,6	0,6	3	2	19	2012
12,5	12,5	0,6	2	2	19	2014
15	21,6	0,6	3	2	21	2015
13,4	15,4	0,6	3	2	21	2005
13,4	15,4	0,6	3	2	21	2005
13,4	15,4	0,6	2	2	19	2006
13,4	15,4	0,6	3	2	23	2009
13,4	15,4	0,6	2	2	26	2009
13,4	15,4	0,6	2	2	19	2013
13,4	15,4	0,6	3	2	22	2015
13,6	16,1	0,6	2	2	20	2000
13,6	16,1	0,6	2	2	22	2000
13,6	16,1	0,6	2	2	26	2000
13,6	16,1	0,6	5	2	26	2000
13,6	16,1	0,6	5	2	21	2005
13,6	16,1	0,6	2	2	25	2008
15,6	24,3	0,6	4	2	15	2015
13,9	17,2	0,6	3	2	21	2008
13,1	14,4	0,6	3	2	23	2005
13,1	14,4	0,6	4	2	23	2006

13,1	14,4	0,6	2	2	26	2006
13,1	14,4	0,6	2	2	23	2007
13,1	14,4	0,6	3	2	20	2008
13,1	14,4	0,6	2	2	25	2008
13,1	14,4	0,6	2	2	24	2009
13,1	14,4	0,6	2	2	21	2014
11,2	9	0,6	2	2	25	2004
11,6	10	0,6	2	2	20	2004
15,2	22,5	0,6	4	2	21	2015
18,1	38	0,6	5	2	19	2013
14,9	21,2	0,6	4	2	20	2001
10,9	8,3	0,6	2	2	23	2004
16,8	30,4	0,6	6	2	20	2013
17,3	33,2	0,6	6	2	20	2002
17,3	33,2	0,6	5	2	19	2013
11,4	9,5	0,6	2	2	23	2004
14	17,6	0,6	3	2	23	2005
14	17,6	0,6	4	2	24	2006
14	17,6	0,6	4	2	23	2009
14	17,6	0,6	2	2	19	2013
14	17,6	0,6	3	2	20	2013
14	17,6	0,6	3	2	17	2014
14,8	20,8	0,6	4	2	20	1999
13,7	16,5	0,6	5	2	19	1999
13,7	16,5	0,6	3	2	22	2011
13	14,1	0,6	2	2	18	2002
13	14,1	0,6	5	2	26	2006
13	14,1	0,6	2	2	19	2013

13	14,1	0,6	2	2	19	2013
15,5	23,9	0,6	3	2	20	2003
15,5	23,9	0,6	5	2	21	2012
13,3	15,1	0,6	2	2	21	2000
13,3	15,1	0,6	2	2	18	2002
13,3	15,1	0,6	3	2	20	2007
13,3	15,1	0,6	2	2	24	2009
13,3	15,1	0,6	2	2	19	2013
13,3	15,1	0,6	2	2	17	2014
17,5	34,4	0,6	8	2	18	2015
15,1	22,1	0,6	3	2	18	2015
20,5	55,3	0,6	6	2	19	2013
14,1	18	0,6	2	2	27	1999
14,1	18	0,6	3	2	21	2012
16,2	27,3	0,6	5	2	18	2000
16,2	27,3	0,6	4	2	17	2014
15,3	23	0,6	4	2	23	2003
15,3	23	0,6	4	2	21	2005
15,3	23	0,6	7	2	17	2009
13,5	15,8	0,6	2	2	24	2001
13,5	15,8	0,6	2	2	23	2002
13,5	15,8	0,6	3	2	19	2008
13,5	15,8	0,6	3	2	15	2015
14,7	20,4	0,6	4	2	22	1999
14,7	20,4	0,6	2	2	26	2001
14,7	20,4	0,6	3	2	24	2002
14,7	20,4	0,6	3	2	23	2003
12	11,1	0,6	2	2	23	2006

12	11,1	0,6	3	2	22	2007
12	11,1	0,6	2	2	23	2007
12	11,1	0,6	2	2	25	2007
12	11,1	0,6	2	2	22	2011
14,2	18,4	0,6	3	2	17	2012
14,6	20	0,6	4	2	23	2011
15,6	24,4	0,6	3	2	19	2001
14,3	18,8	0,6	2	2	19	2000
14,3	18,8	0,6	3	2	22	2003
14,3	18,8	0,6	4	2	22	2011
14,3	18,8	0,6	4	2	21	2013
14,5	19,6	0,6	3	2	26	2000
14,5	19,6	0,6	3	2	17	2008
14,5	19,6	0,6	4	2	21	2009
14,5	19,6	0,6	4	2	19	2012
19,1	44,8	0,6	7	2	18	2012
15	21,7	0,6	3	2	20	2001
15	21,7	0,6	4	2	23	2004
15	21,7	0,6	3	2	17	2014
15	21,7	0,6	3	2	15	2015
14,4	19,2	0,6	4	2	26	2006
10,8	8,1	0,6	2	2	20	2007
13,8	16,9	0,6	4	2	23	2004
13,8	16,9	0,6	4	2	21	2006
13,8	16,9	0,6	3	2	25	2008
11,7	10,3	0,6	1	2	26	2001
11,7	10,3	0,6	2	2	25	2004
17	31,6	0,6	4	2	19	2012

16,5	28,9	0,6	4	2	22	2003
16,5	28,9	0,6	7	2	24	2009
16,5	28,9	0,6	5	2	21	2014
15,7	24,9	0,6	5	2	25	2003
15,7	24,9	0,6	4	2	19	2011
15,4	23,5	0,6	5	2	20	2007
11,1	8,8	0,6	2	2	23	2004
13,2	14,8	0,6	2	2	25	1999
13,2	14,8	0,6	2	2	22	2000
13,2	14,8	0,6	3	2	20	2008
13,2	14,8	0,6	3	2	20	2008
12,1	11,4	0,6	2	2	26	2005
12,1	11,4	0,6	2	2	19	2011
12,1	11,4	0,6	2	2	21	2011
15,2	22,6	0,6	3	2	22	2000
15,2	22,6	0,6	5	2	17	2009
15,2	22,6	0,6	3	2	21	2013
12,8	13,5	0,6	3	2	22	2004
12,8	13,5	0,6	2	2	23	2007
12,8	13,5	0,6	2	2	21	2014
20	51,5	0,6	5	2	20	2013
14,9	21,3	0,6	4	2	21	2006
14,9	21,3	0,6	5	2	19	2012
15,8	25,4	0,6	4	2	20	2003
15,8	25,4	0,6	5	2	23	2005
15,8	25,4	0,6	3	2	18	2008
15,8	25,4	0,6	4	2	18	2015
13,6	16,2	0,6	3	2	21	2009

13,6	16,2	0,6	2	2	20	2013
13,6	16,2	0,6	2	2	21	2014
13,6	16,2	0,6	2	2	23	2014
18,8	42,8	0,6	7	2	21	2014
16,7	30	0,6	4	2	22	2000
19,6	48,5	0,6	8	2	22	2013
13,9	17,3	0,6	3	2	23	1999
13,9	17,3	0,6	3	2	25	2005
13,4	15,5	0,6	3	2	24	2002
13,4	15,5	0,6	2	2	24	2002
13,4	15,5	0,6	2	2	17	2014
16,3	27,9	0,6	7	2	19	2014
12,2	11,7	0,6	2	2	19	2013
12,2	11,7	0,6	2	2	19	2013
11,5	9,8	0,6	2	2	20	2002
12,7	13,2	0,6	2	2	20	2002
12,7	13,2	0,6	2	2	20	2004
12,7	13,2	0,6	2	2	23	2009
12,7	13,2	0,6	2	2	17	2014
16,1	26,9	0,6	7	2	21	2012
17,2	32,8	0,6	6	2	20	2013
14,8	20,9	0,6	5	2	19	2007
14,8	20,9	0,6	3	2	19	2012
18	37,6	0,6	4	2	19	2013
15,1	22,2	0,6	5	2	19	1999
15,1	22,2	0,6	5	2	21	2006
12,3	12	0,6	2	2	20	2013
12,3	12	0,6	2	2	19	2014

10,7	7,9	0,6	2	2	20	2004
12,6	12,9	0,6	2	2	23	2002
16,6	29,5	0,6	7	2	19	2012
13,1	14,5	0,6	2	2	20	2002
13,1	14,5	0,6	3	2	21	2008
13,1	14,5	0,6	2	2	19	2013
20	51,6	0,6	5	2	17	2014
14	17,7	0,6	3	2	19	2013
14	17,7	0,6	3	2	17	2014
14	17,7	0,6	3	2	19	2015
12,4	12,3	0,6	3	2	21	2005
12,4	12,3	0,6	3	2	22	2005
12,5	12,6	0,6	3	2	23	2005
12,5	12,6	0,6	3	2	25	2005
12,5	12,6	0,6	2	2	25	2007
11,8	10,6	0,6	1	2	26	2001
11,8	10,6	0,6	3	2	26	2006
11,8	10,6	0,6	2	2	23	2007
18,7	42,2	0,6	8	2	20	2010
18,7	42,2	0,6	8	2	20	2010
15,6	24,5	0,6	3	2	25	2003
15,6	24,5	0,6	5	2	23	2005
14,7	20,5	0,6	3	2	23	2003
14,7	20,5	0,6	5	2	26	2006
16,5	29	0,6	7	2	21	2014
13,7	16,6	0,6	2	2	24	2002
13,7	16,6	0,6	4	2	24	2002
13,7	16,6	0,6	3	2	22	2005

14,1	18,1	0,6	4	2	20	2011
14,1	18,1	0,6	3	2	22	2015
15	21,8	0,6	4	2	23	1999
15	21,8	0,6	5	2	19	2000
15	21,8	0,6	3	2	22	2004
15	21,8	0,6	3	2	18	2015
15,7	25	0,6	5	2	26	2000
15,7	25	0,6	4	2	16	2015
13,3	15,2	0,6	2	2	21	2000
13,3	15,2	0,6	4	2	23	2009
14,2	18,5	0,6	3	2	24	2002
16,4	28,5	0,6	5	2	21	2015
15,4	23,6	0,6	5	2	20	1999
15,4	23,6	0,6	4	2	23	2003
14,5	19,7	0,6	7	2	25	2002
14,5	19,7	0,6	2	2	20	2003
14,5	19,7	0,6	4	2	24	2009
14,5	19,7	0,6	3	2	19	2012
14,5	19,7	0,6	4	2	18	2012
14,5	19,7	0,6	4	2	15	2015
13,5	15,9	0,6	3	2	23	2015
14,3	18,9	0,6	4	2	23	2009
14,3	18,9	0,6	2	2	21	2013
13	14,2	0,6	3	2	23	2008
13	14,2	0,6	2	2	19	2012
13	14,2	0,6	2	2	20	2013
14,4	19,3	0,6	2	2	19	2000
14,4	19,3	0,6	5	2	21	2000

14,4	19,3	0,6	4	2	15	2015
15,2	22,7	0,6	4	2	16	2008
18,6	41,6	0,6	4	2	19	2012
16,3	28	0,6	5	2	21	2000
16,3	28	0,6	7	2	21	2006
16,3	28	0,6	4	2	17	2012
16,2	27,5	0,6	4	2	18	2001
13,8	17	0,6	3	2	19	2012
14,9	21,4	0,6	5	2	25	2007
14,9	21,4	0,6	5	2	25	2007
14,9	21,4	0,6	3	2	22	2015
16	26,5	0,6	8	2	18	2008
16,1	27	0,6	7	2	25	1999
16,1	27	0,6	4	2	17	2009
17,3	33,5	0,6	5	2	19	2010
17,3	33,5	0,6	5	2	19	2010
11,6	10,1	0,6	2	2	22	2007
15,5	24,1	0,6	3	2	25	2003
15,5	24,1	0,6	4	2	19	2007
15,5	24,1	0,6	3	2	18	2014
19	44,4	0,6	6	2	15	2015
16,8	30,7	0,6	4	2	20	2013
17,5	34,7	0,6	5	2	19	2012
15,1	22,3	0,6	3	2	21	2001
15,1	22,3	0,6	4	2	19	2012
20,1	52,6	0,6	5	2	19	2010
20,1	52,6	0,6	5	2	19	2010
15,3	23,2	0,6	5	2	19	2000

15,3	23,2	0,6	3	2	18	2008
15,3	23,2	0,6	4	2	22	2015
14,8	21	0,6	4	2	21	2006
14,8	21	0,6	10	2	19	2013
13,2	14,9	0,6	2	2	20	2000
13,2	14,9	0,6	2	2	23	2002
13,2	14,9	0,6	5	2	22	2005
13,2	14,9	0,6	3	2	21	2008
13,2	14,9	0,6	3	2	19	2009
13,2	14,9	0,6	2	2	19	2013
13,2	14,9	0,6	2	2	17	2014
13,2	14,9	0,6	2	2	18	2014
10,5	7,5	0,6	1	2	22	2009
11,4	9,6	0,6	2	2	20	2004
11,4	9,6	0,6	2	2	23	2004
15,6	24,6	0,6	5	2	18	2000
15,6	24,6	0,6	5	2	20	2012
13,6	16,3	0,6	2	2	18	2002
13,6	16,3	0,6	3	2	22	2015
12	11,2	0,6	2	2	20	2002
12	11,2	0,6	5	2	23	2007
12	11,2	0,6	2	2	23	2007
12	11,2	0,6	2	2	23	2007
13,4	15,6	0,6	3	2	23	2011
16,7	30,2	0,6	6	2	21	2011
19,9	51,1	0,6	5	2	17	2014
12,8	13,6	0,6	2	2	21	2000
14,7	20,6	0,6	3	2	22	2003

14,7	20,6	0,6	4	2	21	2011
14,7	20,6	0,6	3	2	18	2015
15,7	25,1	0,6	5	2	23	1999
15,7	25,1	0,6	4	2	24	2002
21,8	67,2	0,6	8	2	17	2012
18,8	43,1	0,6	6	2	21	2013
14	17,8	0,6	2	2	21	2008
14	17,8	0,6	4	2	19	2009
14	17,8	0,6	5	2	19	2012
14	17,8	0,6	3	2	17	2014
19	44,5	0,6	7	2	18	2015
15	21,9	0,6	4	2	22	1999
15	21,9	0,6	5	2	24	2002
15	21,9	0,6	4	2	22	2004
15	21,9	0,6	3	2	22	2004
15	21,9	0,6	4	2	18	2012
15	21,9	0,6	3	2	18	2012
15	21,9	0,6	4	2	19	2012
15	21,9	0,6	4	2	21	2012
15	21,9	0,6	3	2	20	2013
15	21,9	0,6	3	2	21	2014
15,4	23,7	0,6	5	2	19	2012
17,8	36,6	0,6	8	2	20	2003
10,4	7,3	0,6	2	2	25	2004
20,8	58,4	0,6	7	2	18	2012
15,8	25,6	0,6	3	2	18	2000
15,8	25,6	0,6	5	2	19	2000
14,6	20,2	0,6	5	2	22	1999

14,6	20,2	0,6	3	2	20	2003
14,6	20,2	0,6	3	2	23	2003
12,1	11,5	0,6	2	2	23	2005
16,2	27,6	0,6	4	2	19	2012
15,2	22,8	0,6	6	2	26	2006
15,2	22,8	0,6	7	2	24	2009
15,2	22,8	0,6	4	2	20	2013
14,1	18,2	0,6	3	2	19	2000
14,1	18,2	0,6	2	2	26	2003
14,1	18,2	0,6	4	2	21	2011
16,6	29,7	0,6	6	2	26	2001
12,7	13,3	0,6	2	2	22	1999
12,7	13,3	0,6	2	2	20	2002
12,7	13,3	0,6	2	2	20	2002
12,7	13,3	0,6	2	2	23	2007
11,7	10,4	0,6	2	2	21	2014
16,1	27,1	0,6	5	2	21	2000
16	26,6	0,6	5	2	22	1999
16	26,6	0,6	6	2	22	2000
16	26,6	0,6	6	2	24	2001
16	26,6	0,6	5	2	21	2005
16	26,6	0,6	4	2	19	2014
18,3	39,8	0,6	10	2	18	2015
13,1	14,6	0,6	3	2	25	2007
13,1	14,6	0,6	2	2	17	2014
13,7	16,7	0,6	3	2	25	1999
13,7	16,7	0,6	3	2	23	2010
13,7	16,7	0,6	3	2	23	2010

13,7	16,7	0,6	2	2	20	2011
14,5	19,8	0,6	3	2	24	2001
14,5	19,8	0,6	4	2	26	2001
14,5	19,8	0,6	3	2	20	2002
14,5	19,8	0,6	2	2	17	2012
14,2	18,6	0,6	3	2	16	2008
14,2	18,6	0,6	3	2	19	2012
14,2	18,6	0,6	3	2	17	2014
14,4	19,4	0,6	4	2	22	2004
14,4	19,4	0,6	2	2	21	2006
14,4	19,4	0,6	4	2	20	2009
14,4	19,4	0,6	3	2	20	2011
14,4	19,4	0,6	3	2	21	2015
12,2	11,8	0,6	2	2	26	2001
15,5	24,2	0,6	5	2	18	2000
15,5	24,2	0,6	3	2	19	2000
15,5	24,2	0,6	6	2	19	2000
15,5	24,2	0,6	4	2	19	2011
15,5	24,2	0,6	4	2	19	2012
12,6	13	0,6	2	2	22	2001
14,9	21,5	0,6	6	2	19	2008
16,5	29,2	0,7	4	2	18	2001
16,5	29,2	0,7	4	2	22	2004
16,5	29,2	0,7	4	2	20	2007
18,2	39,2	0,7	5	2	17	2012
12,5	12,7	0,7	2	2	20	2002
19	44,6	0,7	5	2	19	2013
13,5	16	0,7	2	2	23	2005

13,5	16	0,7	4	2	23	2006
13,3	15,3	0,7	3	2	20	2000
13,3	15,3	0,7	2	2	22	2000
13,3	15,3	0,7	2	2	20	2002
13,3	15,3	0,7	2	2	23	2002
13,3	15,3	0,7	2	2	26	2003
13,3	15,3	0,7	3	2	22	2007
13,3	15,3	0,7	3	2	26	2009
12,4	12,4	0,7	2	2	21	2005
12,4	12,4	0,7	4	2	21	2006
12,4	12,4	0,7	2	2	19	2014
17,2	33,1	0,7	5	2	19	2012
16,9	31,4	0,7	5	2	19	2013
15,3	23,3	0,7	4	2	21	2003
15,3	23,3	0,7	3	2	16	2015
15,1	22,4	0,7	3	2	22	1999
15,1	22,4	0,7	6	2	23	2006
15,1	22,4	0,7	4	2	20	2013
15,6	24,7	0,7	4	2	21	2003
16,4	28,7	0,7	5	2	18	2000
16,4	28,7	0,7	4	2	19	2012
13,8	17,1	0,7	4	2	19	2011
17,3	33,7	0,7	5	2	18	2000
14,8	21,1	0,7	3	2	19	2000
14,8	21,1	0,7	3	2	19	2008
14,8	21,1	0,7	3	2	17	2014
13	14,3	0,7	2	2	24	2002
11,5	9,9	0,7	2	2	20	2004

16,3	28,2	0,7	2	2	23	1999
16,3	28,2	0,7	3	2	20	2003
15,7	25,2	0,7	4	2	19	2011
17,5	34,9	0,7	6	2	20	2013
11,8	10,7	0,7	2	2	23	2007
19,2	46,1	0,7	6	2	19	2010
19,2	46,1	0,7	6	2	19	2010
20,1	52,9	0,7	7	2	19	2012
11,3	9,4	0,7	1	2	26	2001
11,3	9,4	0,7	2	2	23	2007
13,9	17,5	0,7	3	2	25	1999
13,9	17,5	0,7	3	2	18	2008
13,9	17,5	0,7	2	2	21	2014
15,4	23,8	0,7	5	2	22	2000
15,4	23,8	0,7	3	2	20	2003
15,4	23,8	0,7	4	2	23	2015
14,7	20,7	0,7	5	2	21	1999
14,7	20,7	0,7	3	2	18	2000
14,7	20,7	0,7	5	2	19	2000
16,1	27,2	0,7	3	2	19	2012
16,1	27,2	0,7	5	2	21	2013
15,9	26,2	0,7	5	2	19	2013
15	22	0,7	7	2	25	2002
15	22	0,7	3	2	23	2003
15	22	0,7	3	2	20	2006
15	22	0,7	5	2	19	2007
15	22	0,7	3	2	20	2011
15	22	0,7	3	2	19	2013

15	22	0,7	3	2	18	2015
16	26,7	0,7	3	2	22	2003
16	26,7	0,7	4	2	19	2007
15,2	22,9	0,7	4	2	24	2001
15,2	22,9	0,7	4	2	26	2003
15,2	22,9	0,7	4	2	17	2012
12,9	14	0,7	5	2	25	2007
12,9	14	0,7	2	2	22	2009
13,2	15	0,7	3	2	19	2008
13,2	15	0,7	3	2	19	2008
13,2	15	0,7	2	2	21	2014
16,5	29,3	0,7	10	2	23	2007
16,5	29,3	0,7	6	2	20	2011
14,6	20,3	0,7	3	2	18	2000
14,6	20,3	0,7	3	2	20	2000
14,6	20,3	0,7	4	2	20	2011
14,6	20,3	0,7	3	2	19	2014
14,6	20,3	0,7	3	2	18	2015
14	17,9	0,7	3	2	21	2006
14	17,9	0,7	4	2	24	2006
14	17,9	0,7	5	2	19	2007
14	17,9	0,7	4	2	21	2008
14	17,9	0,7	4	2	19	2012
13,4	15,7	0,7	3	2	19	2000
13,4	15,7	0,7	3	2	18	2008
13,4	15,7	0,7	2	2	24	2009
13,4	15,7	0,7	3	2	23	2010
13,4	15,7	0,7	3	2	23	2010

15,5	24,3	0,7	3	2	23	2003
15,5	24,3	0,7	5	2	20	2006
19,2	46,2	0,7	6	2	18	2012
14,5	19,9	0,7	3	2	26	2003
14,1	18,3	0,7	3	2	21	2000
14,1	18,3	0,7	2	2	26	2001
14,1	18,3	0,7	4	2	22	2003
14,1	18,3	0,7	3	2	23	2005
14,1	18,3	0,7	3	2	23	2007
14,1	18,3	0,7	4	2	17	2009
14,1	18,3	0,7	4	2	24	2009
14,1	18,3	0,7	3	2	19	2012
14,1	18,3	0,7	4	2	19	2012
16,4	28,8	0,7	7	2	17	2012
14,9	21,6	0,7	4	2	23	2015
10,7	8	0,7	2	2	23	2004
14,4	19,5	0,7	4	2	23	1999
14,4	19,5	0,7	5	2	20	2000
14,4	19,5	0,7	3	2	22	2001
14,4	19,5	0,7	6	2	21	2008
14,2	18,7	0,7	5	2	20	2007
14,2	18,7	0,7	3	2	21	2008
14,2	18,7	0,7	3	2	18	2014
14,3	19,1	0,7	4	2	23	2003
14,3	19,1	0,7	3	2	21	2008
14,3	19,1	0,7	4	2	23	2009
14,3	19,1	0,7	3	2	19	2014
14,3	19,1	0,7	4	2	19	2015

12,8	13,7	0,7	2	2	26	1999
12,8	13,7	0,7	3	2	19	2008
12,8	13,7	0,7	2	2	26	2009
12,8	13,7	0,7	3	2	23	2010
12,8	13,7	0,7	3	2	23	2010
12,8	13,7	0,7	2	2	18	2014
15,3	23,4	0,7	3	2	19	2012
13,7	16,8	0,7	2	2	21	2000
11,6	10,2	0,7	2	2	23	2007
15,1	22,5	0,7	4	2	17	2009
15,1	22,5	0,7	3	2	18	2012
18,2	39,4	0,7	5	2	17	2014
11	8,7	0,7	2	2	20	2004
11	8,7	0,7	1	2	24	2009
15,7	25,3	0,7	3	2	18	2015
16,8	31	0,7	4	2	25	2004
17,9	37,5	0,7	6	2	17	2012
16,2	27,8	0,7	5	2	22	1999
16,2	27,8	0,7	5	2	22	2009
13,1	14,7	0,7	2	2	18	2002
13,1	14,7	0,7	2	2	18	2002
13,1	14,7	0,7	3	2	25	2007
13,1	14,7	0,7	3	2	19	2008
12	11,3	0,7	2	2	26	2001
12	11,3	0,7	2	2	25	2005
12	11,3	0,7	2	2	23	2007
12	11,3	0,7	2	2	23	2007
14,8	21,2	0,7	3	2	21	2003

14,8	21,2	0,7	5	2	17	2009
15,8	25,8	0,7	5	2	20	1999
16,1	27,3	0,7	4	2	17	2008
12,7	13,4	0,7	2	2	20	2002
12,7	13,4	0,7	2	2	22	2013
12,7	13,4	0,7	2	2	20	2013
15,9	26,3	0,7	6	2	20	2011
16	26,8	0,7	5	2	21	2000
13,5	16,1	0,7	2	2	22	2002
13,5	16,1	0,7	2	2	25	2003
13,5	16,1	0,7	3	2	23	2010
13,5	16,1	0,7	3	2	23	2010
15,4	23,9	0,7	3	2	18	2001
15,4	23,9	0,7	5	2	19	2011
15,4	23,9	0,7	7	2	21	2014
13,3	15,4	0,7	2	2	22	2000
13,3	15,4	0,7	2	2	24	2002
13,3	15,4	0,7	3	2	22	2005
13,3	15,4	0,7	3	2	26	2006
13,3	15,4	0,7	3	2	19	2015
11,4	9,7	0,7	2	2	23	2004
19,6	49,3	0,7	8	2	22	2013
12,1	11,6	0,7	2	2	19	2006
14,7	20,8	0,7	5	2	25	1999
14,7	20,8	0,7	3	2	24	2001
14,7	20,8	0,7	4	2	23	2011
14,7	20,8	0,7	5	2	19	2012
14,7	20,8	0,7	3	2	19	2012

15	22,1	0,7	3	2	20	2003
15	22,1	0,7	4	2	26	2003
15	22,1	0,7	5	2	21	2006
17,6	35,7	0,7	6	2	19	2010
17,6	35,7	0,7	6	2	19	2010
11,2	9,2	0,7	2	2	25	2005
11,2	9,2	0,7	2	2	22	2007
12,6	13,1	0,7	2	2	20	2001
12,6	13,1	0,7	2	2	21	2005
12,6	13,1	0,7	2	2	18	2014
17,5	35,1	0,7	4	2	19	2010
17,5	35,1	0,7	4	2	19	2010
15,2	23	0,7	4	2	23	2001
15,2	23	0,7	4	2	23	2003
15,2	23	0,7	3	2	20	2006
15,2	23	0,7	4	2	22	2011
16,4	28,9	0,7	5	2	19	2000
16,4	28,9	0,7	7	2	20	2007
15,5	24,4	0,7	6	2	20	1999
15,5	24,4	0,7	4	2	20	2003
12,2	11,9	0,7	1	2	26	2001
12,2	11,9	0,7	2	2	23	2006
12,2	11,9	0,7	2	2	24	2006
12,2	11,9	0,7	2	2	23	2007
12,2	11,9	0,7	2	2	22	2011
13,9	17,6	0,7	4	2	23	2005
12,5	12,8	0,7	2	2	19	2013
17	32,2	0,7	5	2	17	2002

13	14,4	0,7	2	2	20	2002
13	14,4	0,7	2	2	21	2008
13	14,4	0,7	2	2	21	2013
12,3	12,2	0,7	2	2	22	2000
12,3	12,2	0,7	3	2	22	2007
12,4	12,5	0,7	2	2	24	2009
16,6	30	0,7	7	2	19	2012
13,6	16,5	0,7	3	2	21	2001
13,6	16,5	0,7	3	2	22	2011
14	18	0,7	5	2	27	1999
14	18	0,7	4	2	23	2004
14	18	0,7	3	2	23	2007
14	18	0,7	2	2	22	2009
14	18	0,7	2	2	19	2012
14,9	21,7	0,7	4	2	20	2011
14,9	21,7	0,7	3	2	19	2012
14,9	21,7	0,7	3	2	21	2015
14,5	20	0,7	3	2	22	2000
14,5	20	0,7	2	2	22	2003
14,5	20	0,7	3	2	21	2014
15,3	23,5	0,7	6	2	24	2001
15,3	23,5	0,7	4	2	20	2008
15,3	23,5	0,7	5	2	22	2015
18,8	43,6	0,7	6	2	19	2013
16,2	27,9	0,7	4	2	22	2004
14,1	18,4	0,7	2	2	24	2002
14,1	18,4	0,7	5	2	21	2006
14,1	18,4	0,7	3	2	18	2008

14,1	18,4	0,7	3	2	19	2014
14,1	18,4	0,7	3	2	22	2015
17,7	36,4	0,7	5	2	19	2013
13,2	15,1	0,7	2	2	21	2012
19,3	47,2	0,7	7	2	17	2012
14,2	18,8	0,7	5	2	23	2006
14,2	18,8	0,7	6	2	23	2008
14,3	19,2	0,7	4	2	22	2001
14,3	19,2	0,7	4	2	25	2002
14,3	19,2	0,7	3	2	20	2008
14,3	19,2	0,7	3	2	20	2012
14,3	19,2	0,7	3	2	15	2015
14,3	19,2	0,7	3	2	21	2015
15,8	25,9	0,7	6	2	23	2005
13,4	15,8	0,7	2	2	27	1999
13,4	15,8	0,7	2	2	23	2001
13,4	15,8	0,7	3	2	20	2002
13,4	15,8	0,7	3	2	25	2008
16,5	29,5	0,7	5	2	25	2004
16	26,9	0,7	5	2	26	2000
16	26,9	0,7	6	2	23	2011
16	26,9	0,7	4	2	19	2012
16,9	31,7	0,7	6	2	17	2012
17,4	34,6	0,7	4	2	19	2012
19,2	46,5	0,7	7	2	18	2012
19,2	46,5	0,7	6	2	15	2015
16,7	30,6	0,7	7	2	19	2012
14,8	21,3	0,7	5	2	23	1999

14,8	21,3	0,7	5	2	26	1999
14,8	21,3	0,7	4	2	22	2004
14,8	21,3	0,7	4	2	23	2005
15,4	24	0,7	6	2	21	1999
13,7	16,9	0,7	2	2	27	1999
13,7	16,9	0,7	2	2	25	2008
11,5	10	0,7	3	2	20	2004
20,6	57,5	0,7	8	2	15	2015
15	22,2	0,7	3	2	19	2012
15,2	23,1	0,7	3	2	17	2002
15,2	23,1	0,7	5	2	19	2007
15,2	23,1	0,7	4	2	23	2008
10,4	7,4	0,7	2	2	23	2004
15,5	24,5	0,7	6	2	20	2013
19,3	47,3	0,7	10	2	19	2013
14,7	20,9	0,7	4	2	20	1999
14,7	20,9	0,7	4	2	18	2001
17,1	32,9	0,7	7	2	18	2000
12,8	13,8	0,7	2	2	19	2002
12,8	13,8	0,7	2	2	26	2009
11,1	9	0,7	2	2	23	2004
16,3	28,5	0,7	5	2	20	1999
16,3	28,5	0,7	4	2	20	2003
16,3	28,5	0,7	7	2	20	2012
13,8	17,3	0,7	2	2	21	2000
13,8	17,3	0,7	3	2	21	2008
13,8	17,3	0,7	2	2	21	2015
13,1	14,8	0,7	3	2	23	2007

19,2	46,6	0,7	8	2	19	2013
11,3	9,5	0,7	2	2	25	2004
13,5	16,2	0,7	2	2	26	2003
13,5	16,2	0,7	3	2	19	2007
13,5	16,2	0,7	4	2	24	2009
18	38,4	0,7	5	2	18	2015
15,6	25	0,7	4	2	19	2007
18,2	39,7	0,7	5	2	17	2014
19,9	51,9	0,7	8	2	19	2008
17,4	34,7	0,7	8	2	26	2001
14,6	20,5	0,7	4	2	19	2000
14,6	20,5	0,7	3	2	25	2007
13,3	15,5	0,7	2	2	23	2002
13,3	15,5	0,7	3	2	21	2008
13,3	15,5	0,7	3	2	21	2008
13,3	15,5	0,7	3	2	18	2015
13,9	17,7	0,7	3	2	18	2001
13,9	17,7	0,7	3	2	21	2003
18,8	43,8	0,7	6	2	19	2013
15,8	26	0,7	5	2	19	2000
16	27	0,7	5	2	21	1999
16	27	0,7	5	2	20	2001
15,9	26,5	0,7	5	2	25	2003
15,9	26,5	0,7	6	2	19	2013
14,5	20,1	0,7	6	2	25	1999
14,5	20,1	0,7	7	2	24	2009
15,1	22,7	0,7	6	2	22	2013
14	18,1	0,7	3	2	25	2004

14	18,1	0,7	4	2	21	2006
14	18,1	0,7	4	2	21	2006
14	18,1	0,7	4	2	20	2009
14	18,1	0,7	4	2	24	2009
12	11,4	0,7	2	2	25	2007
16,4	29,1	0,7	6	2	17	2012
14,4	19,7	0,7	2	2	21	2000
14,4	19,7	0,7	3	2	21	2003
14,4	19,7	0,7	4	2	24	2006
14,4	19,7	0,7	3	2	15	2015
15,4	24,1	0,7	4	2	22	2011
12,6	13,2	0,7	2	2	19	2000
11,6	10,3	0,7	2	2	25	2007
13,6	16,6	0,7	2	2	23	2002
14,1	18,5	0,7	3	2	24	2002
14,1	18,5	0,7	4	2	24	2006
13	14,5	0,7	2	2	20	2000
13	14,5	0,7	3	2	25	2007
13	14,5	0,7	2	2	24	2009
13	14,5	0,7	2	2	23	2010
13	14,5	0,7	2	2	23	2010
10	6,6	0,7	1	2	25	2005
10	6,6	0,7	1	2	23	2007
14,3	19,3	0,7	3	2	22	2004
21,5	65,6	0,7	5	2	18	2012
14,2	18,9	0,7	3	2	25	2003
14,2	18,9	0,7	5	2	24	2006
14,2	18,9	0,7	2	2	25	2008

19,4	48,2	0,7	10+	2	17	2014
12,1	11,7	0,7	3	2	25	2003
12,1	11,7	0,7	2	2	21	2011
12,5	12,9	0,7	2	2	23	1999
15,2	23,2	0,7	3	2	26	2000
15,2	23,2	0,7	4	2	17	2012
15,2	23,2	0,7	5	2	19	2013
15,2	23,2	0,7	5	2	20	2013
15,2	23,2	0,7	3	2	21	2015
15	22,3	0,7	4	2	17	2009
13,4	15,9	0,7	2	2	22	1999
13,4	15,9	0,7	2	2	23	2002
13,4	15,9	0,7	2	2	23	2003
13,4	15,9	0,7	3	2	24	2009
13,4	15,9	0,7	3	2	23	2010
13,4	15,9	0,7	3	2	23	2010
13,4	15,9	0,7	3	2	21	2011
21	61,2	0,7	6	2	18	2012
12,2	12	0,7	2	2	24	2006
12,2	12	0,7	3	2	23	2010
12,2	12	0,7	3	2	23	2010
12,2	12	0,7	2	2	19	2013
12,4	12,6	0,7	2	2	22	2000
12,4	12,6	0,7	2	2	26	2003
12,4	12,6	0,7	2	2	20	2013
13,2	15,2	0,7	5	2	23	1999
13,2	15,2	0,7	2	2	20	2002
13,2	15,2	0,7	3	2	21	2005

13,2	15,2	0,7	2	2	23	2006
13,2	15,2	0,7	3	2	25	2007
13,2	15,2	0,7	3	2	21	2008
13,2	15,2	0,7	2	2	26	2009
13,2	15,2	0,7	3	2	22	2011
13,2	15,2	0,7	3	2	22	2011
13,2	15,2	0,7	3	2	19	2014
13,2	15,2	0,7	3	2	19	2014
12,3	12,3	0,7	2	2	21	2009
14,7	21	0,7	3	2	25	2004
14,7	21	0,7	4	2	18	2014
13,7	17	0,7	5	2	26	1999
13,7	17	0,7	2	2	20	2000
13,7	17	0,7	3	2	25	2004
13,7	17	0,7	3	2	22	2005
13,7	17	0,7	2	2	24	2009
11	8,8	0,7	2	2	23	2004
16,1	27,6	0,7	6	2	19	2013
12,9	14,2	0,7	4	2	21	2006
12,9	14,2	0,7	2	2	20	2013
17	32,5	0,7	6	2	20	2013
15,7	25,6	0,7	3	2	19	2011

15,7	25,6	0,7	4	2	21	2013
16	27,1	0,7	5	2	22	2013
15,3	23,7	0,7	4	2	23	1999
15,3	23,7	0,7	5	2	18	2000
15,3	23,7	0,7	4	2	23	2015
15,9	26,6	0,7	4	2	18	1999
11,7	10,6	0,7	1	2	22	2001
19	45,4	0,7	6	2	19	2013
14,6	20,6	0,7	4	2	25	1999
11,2	9,3	0,7	2	2	25	2004
17,1	33,1	0,7	5	2	21	2015
14,9	21,9	0,7	4	2	19	2012
13,8	17,4	0,7	3	2	25	2007
13,8	17,4	0,7	3	2	25	2008
19,8	51,4	0,7	9	2	20	2010
19,8	51,4	0,7	9	2	20	2010
16,8	31,4	0,7	4	2	19	2004
15,1	22,8	0,7	3	2	19	2007
17,2	33,7	0,7	5	2	19	2014

17,3	34,3	0,7	6	2	20	2003
13,5	16,3	0,7	2	2	27	1999
13,5	16,3	0,7	2	2	21	2000
13,5	16,3	0,7	2	2	19	2000
13,5	16,3	0,7	3	2	22	2005
13,5	16,3	0,7	3	2	23	2007
13,5	16,3	0,7	3	2	24	2009
13,5	16,3	0,7	2	2	21	2015
14,5	20,2	0,7	4	2	21	1999
14,5	20,2	0,7	5	2	22	2007
14,5	20,2	0,7	4	2	15	2015
15,4	24,2	0,7	4	2	19	2011
15,4	24,2	0,7	6	2	19	2012
16,3	28,7	0,7	4	2	24	2002
19,6	49,9	0,7	7	2	18	2012
13,1	14,9	0,7	2	2	19	2000
13,1	14,9	0,7	2	2	20	2006
13,1	14,9	0,7	2	2	20	2008
13,1	14,9	0,7	2	2	23	2015

13,9	17,8	0,7	3	2	22	2000
13,3	15,6	0,7	2	2	19	1999
13,3	15,6	0,7	3	2	19	2002
13,3	15,6	0,7	2	2	23	2002
13,3	15,6	0,7	2	2	23	2003
13,3	15,6	0,7	2	2	23	2010
13,3	15,6	0,7	2	2	23	2010
14,4	19,8	0,7	4	2	23	1999
14,4	19,8	0,7	5	2	25	1999
14,4	19,8	0,7	3	2	22	2004
14,4	19,8	0,7	6	2	22	2011
14,4	19,8	0,7	3	2	21	2015
14,8	21,5	0,7	5	2	18	1999
14,8	21,5	0,7	4	2	25	1999
14,8	21,5	0,7	4	2	18	2015
15,5	24,7	0,7	5	2	23	1999
14,3	19,4	0,7	4	2	24	2006
14,3	19,4	0,7	3	2	20	2012
16,7	30,9	0,7	4	2	18	2001

16,7	30,9	0,7	4	2	18	2002
15,2	23,3	0,7	5	2	20	1999
14,1	18,6	0,7	3	2	19	2006
14,1	18,6	0,7	3	2	25	2007
14,1	18,6	0,7	6	2	20	2008
14,1	18,6	0,7	3	2	21	2008
15	22,4	0,7	6	2	22	1999
15	22,4	0,7	4	2	19	2007
15	22,4	0,7	4	2	20	2011
16,1	27,7	0,7	4	2	24	2001
16,1	27,7	0,7	3	2	20	2003
16,1	27,7	0,7	5	2	20	2003
15,6	25,2	0,7	3	2	23	2003
15,6	25,2	0,7	4	2	21	2015
13,6	16,7	0,7	3	2	23	1999
13,6	16,7	0,7	3	2	25	2005
12,7	13,6	0,7	3	2	23	2004
12,7	13,6	0,7	2	2	23	2007
17,1	33,2	0,7	5	2	18	2000

17,1	33,2	0,7	6	2	19	2013
11,5	10,1	0,7	1	2	26	2001
11,5	10,1	0,7	3	2	25	2004
15,7	25,7	0,7	4	2	26	2001
15,8	26,2	0,7	5	2	18	2014
14,7	21,1	0,7	4	2	20	1999
14,7	21,1	0,7	3	2	21	2015
17,2	33,8	0,7	5	2	17	2002
17,2	33,8	0,7	5	2	18	2014
16,4	29,3	0,7	6	2	23	2005
17,4	35	0,7	5	2	19	2000
15,3	23,8	0,7	5	2	23	1999
15,3	23,8	0,7	5	2	22	2000
15,3	23,8	0,7	7	2	19	2012
13	14,6	0,7	3	2	19	2006
13	14,6	0,7	2	2	23	2007
13	14,6	0,7	2	2	21	2008
13	14,6	0,7	2	2	19	2009
13	14,6	0,7	2	2	21	2009

11,9	11,2	0,7	1	2	26	2001
13,4	16	0,7	2	2	22	2000
13,4	16	0,7	2	2	23	2002
13,4	16	0,7	3	2	25	2003
13,4	16	0,7	2	2	19	2009
13,7	17,1	0,7	4	2	22	2004
16,9	32,1	0,7	6	2	19	2000
15,1	22,9	0,7	6	2	21	1999
15,1	22,9	0,7	3	2	22	2004
14,6	20,7	0,7	4	2	22	2004
14,6	20,7	0,7	3	2	19	2012
10,5	7,7	0,7	2	2	20	2004
18,2	40,1	0,7	7	2	18	2012
13,2	15,3	0,7	2	2	26	2000
13,2	15,3	0,7	3	2	25	2007
13,2	15,3	0,7	3	2	21	2014
11,3	9,6	0,7	2	2	25	2004
15,4	24,3	0,7	3	2	22	2001
15,4	24,3	0,7	5	2	21	2003

15,4	24,3	0,7	4	2	23	2003
15,4	24,3	0,7	5	2	25	2005
12	11,5	0,7	2	2	19	2013
17	32,7	0,7	4	2	18	2015
12,5	13	0,7	2	2	19	2006
12,5	13	0,7	2	2	21	2013
12,5	13	0,7	2	2	22	2013
17,6	36,3	0,7	5	2	18	2000
17,6	36,3	0,7	4	2	19	2013
14,5	20,3	0,7	3	2	23	2004
14,5	20,3	0,7	3	2	22	2011
14,5	20,3	0,7	5	2	19	2013
14,5	20,3	0,7	3	2	21	2014
12,1	11,8	0,7	3	2	23	2005
12,1	11,8	0,7	2	2	21	2009
12,1	11,8	0,7	2	2	24	2009
12,1	11,8	0,7	2	2	23	2010
12,1	11,8	0,7	2	2	23	2010
16,1	27,8	0,7	3	2	24	2001

16,1	27,8	0,7	4	2	26	2003
20,1	54,1	0,7	8	2	18	2012
20,1	54,1	0,7	10	2	19	2013
11,6	10,4	0,7	1	2	23	2015
14,8	21,6	0,7	5	2	21	2001
14,8	21,6	0,7	8	2	19	2011
14,8	21,6	0,7	3	2	19	2013
17,3	34,5	0,7	5	2	21	2000
15,2	23,4	0,7	8	2	17	2009
15,2	23,4	0,7	5	2	18	2012
12,2	12,1	0,7	2	2	23	2007
16,8	31,6	0,7	4	2	17	2002
14,4	19,9	0,7	4	2	24	2006
16	27,3	0,7	6	2	26	2006
16	27,3	0,7	5	2	20	2012
13,9	17,9	0,7	3	2	23	2010
13,9	17,9	0,7	3	2	23	2010
13,5	16,4	0,7	2	2	27	1999
13,5	16,4	0,7	3	2	19	2000

13,5	16,4	0,7	5	2	19	2000
13,5	16,4	0,7	2	2	26	2001
13,5	16,4	0,7	2	2	26	2001
13,5	16,4	0,7	2	2	23	2008
15	22,5	0,7	6	2	19	2001
15	22,5	0,7	3	2	19	2012
15	22,5	0,7	4	2	15	2015
15,7	25,8	0,7	5	2	22	1999
15,7	25,8	0,7	5	2	23	1999
15,7	25,8	0,7	4	2	23	2002
15,9	26,8	0,7	4	2	21	2012
10,4	7,5	0,7	2	2	23	2004
16,6	30,5	0,7	7	2	19	2012
10,8	8,4	0,7	2	2	25	2004
14,3	19,5	0,7	3	2	23	2003
14,3	19,5	0,7	4	2	23	2003
14,3	19,5	0,7	2	2	23	2003
14,3	19,5	0,7	5	2	21	2012
14	18,3	0,7	4	2	26	1999

14	18,3	0,7	3	2	18	2000
14	18,3	0,7	4	2	23	2008
14	18,3	0,7	4	2	20	2012
14	18,3	0,7	3	2	22	2015
18	38,9	0,7	5	2	20	2013
18	38,9	0,7	4	2	18	2015
14,2	19,1	0,7	5	2	22	1999
14,2	19,1	0,7	3	2	22	2000
14,2	19,1	0,7	7	2	21	2009
14,2	19,1	0,7	2	2	20	2013
14,1	18,7	0,7	4	2	23	1999
14,1	18,7	0,7	5	2	21	2009
14,1	18,7	0,7	3	2	19	2012
16,9	32,2	0,7	6	2	22	2013
13,1	15	0,7	2	2	25	2003
13,1	15	0,7	2	2	19	2009
15,3	23,9	0,7	3	2	17	2008
16,3	28,9	0,7	3	2	26	2001
16,3	28,9	0,7	4	2	18	2012

16,3	28,9	0,7	6	2	21	2013
13,3	15,7	0,7	2	2	25	1999
13,3	15,7	0,7	3	2	19	2000
13,3	15,7	0,7	3	2	21	2008
19,1	46,5	0,7	7	2	17	2012
18,3	40,9	0,7	8	2	18	2015
14,7	21,2	0,7	4	2	20	2012
21,3	64,5	0,7	10	2	20	2013
13,6	16,8	0,7	2	2	27	1999
13,6	16,8	0,7	3	2	23	2006
13,6	16,8	0,7	2	2	20	2013
15,1	23	0,7	4	2	24	2001
15,1	23	0,7	5	2	23	2006
15,1	23	0,7	5	2	19	2008
15,1	23	0,7	4	2	20	2008
15,1	23	0,7	3	2	19	2012
11,7	10,7	0,7	2	2	20	2004
11,4	9,9	0,7	2	2	23	2004
19,2	47,3	0,7	7	2	18	2012

16,8	31,7	0,7	5	2	20	2002
15,5	24,9	0,7	5	2	19	2000
11	8,9	0,7	2	2	23	2004
18	39	0,7	8	2	18	2015
13,7	17,2	0,7	4	2	26	1999
13,7	17,2	0,7	4	2	23	2009
13,7	17,2	0,7	4	2	19	2011
13,7	17,2	0,7	3	2	21	2011
16	27,4	0,7	3	2	20	2003
16	27,4	0,7	4	2	21	2013
16,6	30,6	0,7	4	2	18	2012
15,6	25,4	0,7	5	2	18	1999
15,6	25,4	0,7	5	2	18	1999
11,2	9,4	0,7	2	2	20	2004
11,2	9,4	0,7	2	2	25	2004
13	14,7	0,7	3	2	22	2013
13	14,7	0,7	2	2	18	2014
13,4	16,1	0,7	3	2	15	2015
14,5	20,4	0,7	3	2	22	2003

15,2	23,5	0,7	4	2	26	2006
15,2	23,5	0,7	7	2	24	2009
15,2	23,5	0,7	5	2	19	2012
15,9	26,9	0,7	4	2	24	2002
15,7	25,9	0,7	5	2	20	1999
15,8	26,4	0,7	5	2	18	1999
13,2	15,4	0,7	3	2	25	1999
13,2	15,4	0,7	2	2	22	2000
13,2	15,4	0,7	3	2	25	2007
13,2	15,4	0,7	2	2	23	2009
15	22,6	0,7	3	2	20	2003
15	22,6	0,7	7	2	22	2011
15	22,6	0,7	4	2	19	2012
15	22,6	0,7	3	2	19	2013
16,3	29	0,7	5	2	18	1999
17	32,9	0,7	7	2	25	2002
17	32,9	0,7	5	2	19	2013
17	32,9	0,7	4	2	21	2013
19,2	47,4	0,7	5	2	18	2012

13,8	17,6	0,7	3	2	25	2007
14,4	20	0,7	3	2	26	2000
12,6	13,4	0,7	3	2	25	2005
12,6	13,4	0,7	5	2	21	2012
16,5	30,1	0,7	5	2	17	2014
17,4	35,3	0,7	7	2	19	2012
17,4	35,3	0,7	5	2	18	2014
15,3	24	0,7	3	2	19	2000
15,3	24	0,7	4	2	26	2003
17,3	34,7	0,7	10	2	20	2013
14,3	19,6	0,7	3	2	26	2000
14,3	19,6	0,7	3	2	24	2002
14,3	19,6	0,7	2	2	20	2012
14,3	19,6	0,7	3	2	22	2015
14	18,4	0,7	2	2	19	2001
14	18,4	0,7	3	2	23	2002
14	18,4	0,7	3	2	21	2008
14,2	19,2	0,7	3	2	23	2007
14,2	19,2	0,7	5	2	25	2007

11,9	11,3	0,7	2	2	19	2006
13,5	16,5	0,7	2	2	26	2000
13,5	16,5	0,7	3	2	22	2005
13,5	16,5	0,7	2	2	20	2013
19	46	0,7	6	2	19	2013
14,1	18,8	0,7	2	2	20	2003
11,5	10,2	0,7	2	2	20	2004
12,5	13,1	0,7	2	2	25	2007
12,5	13,1	0,7	2	2	22	2009
12,9	14,4	0,7	5	2	20	2012
15,4	24,5	0,7	5	2	26	2009
16,1	28	0,7	4	2	22	2009
15,1	23,1	0,7	5	2	19	1999
15,1	23,1	0,7	3	2	17	2014
16,4	29,6	0,7	5	2	22	2001
16,4	29,6	0,7	5	2	19	2013
12	11,6	0,7	2	2	23	2007
12	11,6	0,7	2	2	21	2008
12	11,6	0,7	2	2	23	2010

12	11,6	0,7	2	2	23	2010
15,5	25	0,7	4	2	21	1999
15,5	25	0,7	5	2	22	2009
12,4	12,8	0,7	2	2	20	2013
12,4	12,8	0,7	2	2	19	2014
15,5	25	0,7	3	2	15	2015
16	27,5	0,7	4	2	21	2012
14,6	20,9	0,7	3	2	16	2008
13,3	15,8	0,7	2	2	23	2002
13,1	15,1	0,7	2	2	23	2002
13,1	15,1	0,7	4	2	23	2005
13,1	15,1	0,7	2	2	18	2014
13,1	15,1	0,7	3	2	19	2014
15,6	25,5	0,7	5	2	23	2003
12,1	11,9	0,7	2	2	23	2007
12,1	11,9	0,7	2	2	23	2007
13,6	16,9	0,7	2	2	26	2001
13,6	16,9	0,7	5	2	25	2007
15,7	26	0,7	7	2	19	2011

15,8	26,5	0,7	8	2	23	2015
12,2	12,2	0,7	3	2	23	2007
12,2	12,2	0,7	2	2	26	2009
16,3	29,1	0,7	7	2	19	2013
15,2	23,6	0,7	5	2	20	2006
12,8	14,1	0,7	2	2	21	2000
12,8	14,1	0,7	2	2	17	2002
14,5	20,5	0,7	5	2	18	2000
14,5	20,5	0,7	5	2	20	2000
14,5	20,5	0,7	4	2	20	2011
14,5	20,5	0,7	4	2	20	2011
14,8	21,8	0,7	3	2	17	2008
15	22,7	0,7	3	2	23	2002
15	22,7	0,7	3	2	19	2006
17,7	37,3	0,7	4	2	16	2015
11,1	9,2	0,7	2	2	23	2005
16,2	28,6	0,7	6	2	18	2000
16,2	28,6	0,7	6	2	26	2001
16,2	28,6	0,7	5	2	19	2012

16,2	28,6	0,7	5	2	17	2014
16,8	31,9	0,7	5	2	17	2012
13,7	17,3	0,7	2	2	22	2000
13,7	17,3	0,7	3	2	19	2009
15,3	24,1	0,7	4	2	22	2011
14,4	20,1	0,7	4	2	22	2011
13,4	16,2	0,7	2	2	19	2000
13,4	16,2	0,7	3	2	25	2007
13,4	16,2	0,7	3	2	15	2015
16,4	29,7	0,7	5	2	21	1999
16,6	30,8	0,7	6	2	23	2010
16,6	30,8	0,7	6	2	23	2010
13,8	17,7	0,7	3	2	23	2015
15,4	24,6	0,7	4	2	18	1999
15,4	24,6	0,7	5	2	24	2002
13	14,8	0,7	2	2	26	1999
13	14,8	0,7	3	2	25	2005
14,3	19,7	0,7	3	2	21	2000
14,3	19,7	0,7	4	2	23	2011

14,3	19,7	0,7	3	2	19	2012
14,7	21,4	0,7	3	2	19	2002
12,7	13,8	0,7	2	2	19	2009
10,5	7,8	0,7	7	2	20	2004
10,5	7,8	0,7	2	2	23	2004
16	27,6	0,7	5	2	18	2012
15,1	23,2	0,7	4	2	22	2000
15,1	23,2	0,7	5	2	19	2012
15,1	23,2	0,7	4	2	19	2012
18	39,3	0,7	5	2	18	2012
13,2	15,5	0,7	3	2	24	2006
13,9	18,1	0,7	3	2	21	2008
14,2	19,3	0,7	3	2	17	2008
14,9	22,3	0,7	5	2	19	2012
14,9	22,3	0,7	3	2	21	2014
14,9	22,3	0,7	4	2	22	2015
14	18,5	0,7	4	2	21	1999
14	18,5	0,7	3	2	24	2002
14	18,5	0,7	2	2	23	2002

14	18,5	0,7	2	2	23	2003
14	18,5	0,7	6	2	21	2008
14,1	18,9	0,7	3	2	17	2014
14,1	18,9	0,7	4	2	22	2015
11,7	10,8	0,7	2	2	22	2000
11,7	10,8	0,7	2	2	25	2004
11,7	10,8	0,7	2	2	25	2004
15,6	25,6	0,7	6	2	25	2004
15,6	25,6	0,7	4	2	18	2008
13,5	16,6	0,7	2	2	18	2002
13,5	16,6	0,7	4	2	19	2009
21,8	69,9	0,7	7	2	18	2012
10,8	8,5	0,7	1	2	26	2001
14,6	21	0,7	3	2	23	2007
15,2	23,7	0,7	4	2	22	2013
12,6	13,5	0,7	2	2	25	2007
12,6	13,5	0,7	2	2	21	2013
11,4	10	0,7	2	2	25	2005
16,2	28,7	0,7	5	2	18	2008

14,8	21,9	0,7	3	2	19	1999
14,8	21,9	0,7	4	2	24	2006
15	22,8	0,7	3	2	25	2007
15	22,8	0,7	4	2	21	2011
11,8	11,1	0,7	2	2	25	2007
18	39,4	0,7	5	2	19	2013
16,4	29,8	0,7	7	2	19	2012
15,3	24,2	0,7	3	2	19	2013
14,5	20,6	0,7	3	2	21	2003
14,5	20,6	0,7	7	2	24	2009
16,1	28,2	0,7	6	2	20	2011
13,3	15,9	0,7	2	2	22	2000
13,3	15,9	0,7	3	2	22	2005
13,3	15,9	0,7	3	2	23	2010
13,3	15,9	0,7	3	2	23	2010
13,3	15,9	0,7	3	2	15	2015
17,1	33,8	0,7	3	2	18	2012
13,1	15,2	0,7	2	2	26	2006
11	9	0,7	2	2	22	2004

11	9	0,7	2	2	23	2004
16	27,7	0,7	4	2	18	2000
16	27,7	0,7	4	2	23	2002
16	27,7	0,7	4	2	19	2011
16	27,7	0,7	6	2	20	2013
15,4	24,7	0,7	6	2	24	2001
15,4	24,7	0,7	3	2	21	2003
15,4	24,7	0,7	7	2	26	2006
14,4	20,2	0,7	2	2	27	1999
14,4	20,2	0,7	3	2	22	2003
14,4	20,2	0,7	4	2	24	2009
11,9	11,4	0,7	2	2	25	2008
17,9	38,8	0,7	5	2	19	2012
15,9	27,2	0,7	5	2	18	1999
13,7	17,4	0,7	2	2	26	2000
13,7	17,4	0,7	4	2	22	2001
13,7	17,4	0,7	2	2	25	2005
15,5	25,2	0,7	4	2	19	2000
15,1	23,3	0,7	5	2	21	1999

15,1	23,3	0,7	3	2	18	2012
15,1	23,3	0,7	4	2	21	2012
12	11,7	0,7	2	2	23	2005
14,3	19,8	0,7	5	2	26	2000
14,3	19,8	0,7	4	2	25	2004
12,8	14,2	0,7	2	2	22	2002
12,8	14,2	0,7	2	2	19	2006
14,9	22,4	0,7	4	2	20	2011
10,3	7,4	0,7	2	2	23	2004
13,8	17,8	0,7	3	2	25	2005
13,8	17,8	0,7	3	2	20	2009
12,2	12,3	0,7	3	2	23	2007
16,2	28,8	0,7	6	2	19	2011
16,2	28,8	0,7	4	2	19	2012
13,4	16,3	0,7	2	2	22	2000
13,4	16,3	0,7	3	2	23	2008
13,4	16,3	0,7	3	2	25	2008
20	54,2	0,7	10	2	22	2013
10,7	8,3	0,7	2	2	23	2004

10,7	8,3	0,7	2	2	25	2004
14,2	19,4	0,7	3	2	23	2001
14,2	19,4	0,7	4	2	20	2011
14,2	19,4	0,7	3	2	21	2014
14,2	19,4	0,7	3	2	23	2014
16,6	31	0,7	4	2	20	2003
15,2	23,8	0,7	4	2	24	2001
15,2	23,8	0,7	3	2	24	2002
15,2	23,8	0,7	4	2	17	2012
14,1	19	0,7	3	2	19	2000
14,1	19	0,7	3	2	23	2002
14,1	19	0,7	6	2	18	2008
17	33,3	0,7	4	2	19	2014
14	18,6	0,7	5	2	23	1999
17,3	35,1	0,7	5	2	19	2014
14,6	21,1	0,7	5	2	27	1999
14,6	21,1	0,7	4	2	20	2011
16,1	28,3	0,7	4	2	22	2003
16,1	28,3	0,7	6	2	23	2003

16,1	28,3	0,7	6	2	23	2007
13	14,9	0,7	5	2	25	1999
13	14,9	0,7	3	2	23	2008
13	14,9	0,7	4	2	24	2009
13	14,9	0,7	2	2	22	2011
13,2	15,6	0,7	5	2	26	1999
13,2	15,6	0,7	2	2	26	2000
13,2	15,6	0,7	2	2	23	2006
13,2	15,6	0,7	4	2	22	2009
13,2	15,6	0,7	3	2	23	2011
15,3	24,3	0,7	6	2	22	2001
15,3	24,3	0,7	4	2	23	2002
15,3	24,3	0,7	5	2	25	2004
15	22,9	0,7	4	2	20	1999
15	22,9	0,7	8	2	23	2008
12,7	13,9	0,7	4	2	20	2006
12,7	13,9	0,7	2	2	20	2013
12,7	13,9	0,7	2	2	21	2014
14,8	22	0,7	5	2	25	1999

14,8	22	0,7	4	2	26	2001
14,8	22	0,7	3	2	23	2003
16	27,8	0,7	8	2	25	2008
13,5	16,7	0,7	3	2	20	2002
13,5	16,7	0,7	4	2	25	2004
13,5	16,7	0,7	3	2	23	2009
16,3	29,4	0,7	5	2	18	2012
18	39,6	0,7	7	2	19	2013
16,8	32,2	0,7	5	2	18	2000
11,6	10,6	0,7	2	2	23	2004
11,3	9,8	0,7	2	2	23	2004
15,8	26,8	0,7	6	2	24	2001
15,6	25,8	0,7	5	2	23	1999
15,7	26,3	0,7	5	2	22	2001
15,7	26,3	0,7	5	2	23	2003
15,1	23,4	0,7	5	2	25	2003
15,1	23,4	0,7	3	2	18	2012
13,6	17,1	0,7	2	2	20	2000
13,6	17,1	0,7	2	2	22	2000

13,6	17,1	0,7	3	2	21	2003
13,6	17,1	0,7	2	2	25	2003
17	33,4	0,7	5	2	19	2013
14,4	20,3	0,7	3	2	19	2012
12,6	13,6	0,7	2	2	20	2002
12,6	13,6	0,7	2	2	20	2002
12,6	13,6	0,7	2	2	26	2009
14,7	21,6	0,7	4	2	19	2012
11,1	9,3	0,7	2	2	25	2004
13,3	16	0,7	2	2	19	2012
18,2	41	0,7	6	2	19	2013
12,9	14,6	0,7	2	2	17	2008
16,4	30	0,7	5	2	26	2001
14,9	22,5	0,7	5	2	25	2007
14,3	19,9	0,7	4	2	15	2015
14,3	19,9	0,7	3	2	22	2015
15,2	23,9	0,7	4	2	21	2000
15,2	23,9	0,7	3	2	20	2001
11,7	10,9	0,7	1	2	22	2009

13,7	17,5	0,7	2	2	23	2002
13,1	15,3	0,7	4	2	24	2009
13,1	15,3	0,7	2	2	21	2014
20,2	56,1	0,7	4	2	18	2012
16,7	31,7	0,7	5	2	19	2000
12,5	13,3	0,7	3	2	22	2005
14,2	19,5	0,7	4	2	24	2006
14,2	19,5	0,7	4	2	25	2008
13,8	17,9	0,7	4	2	21	2006
13,8	17,9	0,7	2	2	22	2009
13,8	17,9	0,7	3	2	20	2011
16,5	30,6	0,7	6	2	23	2003
14,6	21,2	0,7	6	2	20	2011
14,6	21,2	0,7	3	2	20	2011
15,3	24,4	0,7	4	2	20	1999
15,3	24,4	0,7	3	2	23	2002
14,1	19,1	0,7	3	2	22	2007
14,1	19,1	0,7	4	2	21	2009
14,1	19,1	0,7	4	2	24	2009

15	23	0,7	5	2	18	2000
15	23	0,7	3	2	19	2013
14	18,7	0,7	5	2	26	2000
14	18,7	0,7	3	2	19	2006
14	18,7	0,7	3	2	21	2014
13,4	16,4	0,7	3	2	25	2005
17,7	37,8	0,7	7	2	21	2003
11,4	10,1	0,7	2	2	22	2007
14,8	22,1	0,7	4	2	24	2006
15,4	24,9	0,7	6	2	18	2008
12,4	13	0,7	2	2	20	2000
12,4	13	0,7	3	2	25	2004
17,1	34,1	0,7	8	2	19	2013
15,8	26,9	0,7	4	2	23	2002
15,8	26,9	0,7	4	2	23	2005
15,8	26,9	0,7	6	2	20	2011
15,5	25,4	0,7	5	2	18	1999
15,5	25,4	0,7	4	2	20	1999
15,5	25,4	0,7	3	2	23	2001

15,7	26,4	0,7	4	2	21	1999
15,6	25,9	0,7	3	2	21	2003
14,5	20,8	0,7	3	2	23	2002
14,5	20,8	0,7	3	2	21	2012
16,4	30,1	0,7	6	2	20	2013
19,2	48,3	0,7	6	2	22	2013
10,5	7,9	0,7	2	2	20	2004
10,5	7,9	0,7	2	2	25	2004
12,3	12,7	0,7	2	2	19	2011
15,1	23,5	0,7	5	2	20	2013
13,2	15,7	0,7	3	2	26	2005
13,2	15,7	0,7	3	2	20	2011
13	15	0,7	3	2	25	2007
13	15	0,7	3	2	25	2007
16,7	31,8	0,7	4	2	20	2011
13,5	16,8	0,7	2	2	22	2000
13,5	16,8	0,7	4	2	24	2009
12	11,8	0,7	2	2	22	2007
12	11,8	0,7	2	2	23	2007

12,2	12,4	0,7	2	2	22	2000
14,7	21,7	0,7	4	2	20	1999
14,7	21,7	0,7	6	2	20	1999
11,2	9,6	0,7	2	2	17	2014
15,2	24	0,7	4	2	19	2000
18,2	41,2	0,7	5	2	17	2012
16,5	30,7	0,7	5	2	17	2012
12,7	14	0,7	3	2	15	2015
18,7	44,7	0,7	10	2	16	2015
17,3	35,4	0,7	5	2	19	2000
17,3	35,4	0,7	5	2	20	2013
13,6	17,2	0,7	2	2	22	2000
17	33,6	0,7	9	2	17	2012
14,3	20	0,7	6	2	26	1999
14,3	20	0,7	3	2	21	2015
15,9	27,5	0,7	5	2	19	2013
16,6	31,3	0,7	6	2	22	2001
14,6	21,3	0,7	4	2	23	1999
14,6	21,3	0,7	3	2	22	2000

14,6	21,3	0,7	2	2	19	2000
15	23,1	0,7	5	2	19	2007
15	23,1	0,7	4	2	25	2007
15	23,1	0,7	4	2	20	2011
16,2	29,1	0,7	6	2	22	2011
16,2	29,1	0,7	7	2	17	2012
13,7	17,6	0,7	3	2	21	2000
13,7	17,6	0,7	2	2	18	2002
13,7	17,6	0,7	2	2	23	2002
13,7	17,6	0,7	4	2	15	2015
10,4	7,7	0,7	1	2	26	2001
10,4	7,7	0,7	2	2	23	2004
15,8	27	0,7	5	2	25	1999
15,8	27	0,7	4	2	22	2011
16,4	30,2	0,7	6	2	19	2012
15,5	25,5	0,7	4	2	19	2001
15,7	26,5	0,7	4	2	21	1999
15,7	26,5	0,7	7	2	26	1999
15,7	26,5	0,7	4	2	19	2012

12,9	14,7	0,7	3	2	23	2005
15,6	26	0,7	8	2	20	2013
12,6	13,7	0,7	2	2	26	2009
13,8	18	0,7	2	2	26	2000
14,1	19,2	0,7	4	2	24	2006
13,1	15,4	0,7	3	2	22	2013
18,2	41,3	0,7	7	2	19	2012
14	18,8	0,7	4	2	26	1999
14	18,8	0,7	3	2	20	2001
14	18,8	0,7	3	2	22	2004
14	18,8	0,7	4	2	25	2007
15,1	23,6	0,7	3	2	20	2003
14,5	20,9	0,7	4	2	26	1999
14,5	20,9	0,7	4	2	24	2002
14,5	20,9	0,7	3	2	25	2004
14,5	20,9	0,7	3	2	18	2012
16,5	30,8	0,7	7	2	23	2011
10,7	8,4	0,7	2	2	23	2004
13,4	16,5	0,7	2	2	23	2002

13,4	16,5	0,7	3	2	25	2005
18,9	46,3	0,7	5	2	22	2013
17,1	34,3	0,7	7	2	24	2009
16	28,1	0,7	4	2	21	2003
12,5	13,4	0,7	3	2	21	2005
12,5	13,4	0,7	2	2	20	2013
12,5	13,4	0,7	2	2	19	2014
11,3	9,9	0,7	2	2	25	2004
11,3	9,9	0,7	2	2	25	2004
16,6	31,4	0,7	8	2	23	2003
14,4	20,5	0,7	3	2	24	2002
14,4	20,5	0,7	4	2	17	2012
14,4	20,5	0,7	3	2	23	2015
12,8	14,4	0,7	2	2	23	1999
12,8	14,4	0,7	2	2	25	2008
15,3	24,6	0,7	5	2	19	2012
13,5	16,9	0,7	2	2	24	2009
16,7	32	0,7	6	2	23	2004
16,7	32	0,7	5	2	19	2013

12,4	13,1	0,7	2	2	21	2014
18,9	46,4	0,7	7	2	19	2012
13	15,1	0,7	2	2	26	2009
15,7	26,6	0,7	3	2	22	2001
14,3	20,1	0,7	3	2	25	2003
15	23,2	0,7	4	2	18	2000
15	23,2	0,7	4	2	20	2011
15,5	25,6	0,7	3	2	19	2000
15,5	25,6	0,7	4	2	19	2012
16,8	32,6	0,7	5	2	20	2013
13,6	17,3	0,7	3	2	24	2002
16,9	33,2	0,7	5	2	17	2012
17,2	35	0,7	5	2	19	2000
12,3	12,8	0,7	3	2	23	2007
12,3	12,8	0,7	2	2	25	2007
12,3	12,8	0,7	2	2	22	2011
14,8	22,3	0,7	4	2	24	2006
17	33,8	0,7	4	2	21	2013
17,1	34,4	0,7	7	2	19	2012

14,2	19,7	0,7	3	2	17	2008
14,2	19,7	0,7	3	2	17	2012
16,3	29,8	0,7	6	2	25	1999
16,3	29,8	0,7	6	2	22	2001
16,3	29,8	0,7	4	2	19	2012
19	47,2	0,7	4	2	16	2015
12,7	14,1	0,7	2	2	20	2002
12,7	14,1	0,7	2	2	24	2009
12,7	14,1	0,7	2	2	20	2013
11,9	11,6	0,7	4	2	26	2006
15,1	23,7	0,7	5	2	26	2000
15,1	23,7	0,7	4	2	22	2013
12,2	12,5	0,7	2	2	20	2004
12,2	12,5	0,7	3	2	23	2005
11,4	10,2	0,7	2	2	26	2009
14,1	19,3	0,7	2	2	26	2000
14,1	19,3	0,7	4	2	23	2006
14,1	19,3	0,7	3	2	18	2008
17,5	36,9	0,7	10	2	21	2013

13,3	16,2	0,7	2	2	22	2002
13,3	16,2	0,7	4	2	23	2008
13,3	16,2	0,7	6	2	24	2009
18,5	43,6	0,7	6	2	22	2013
12	11,9	0,7	2	2	19	2000
12	11,9	0,7	2	2	23	2005
12,1	12,2	0,7	2	2	23	2007
12,1	12,2	0,7	2	2	25	2007
13,8	18,1	0,7	5	2	19	2012
14,5	21	0,7	3	2	18	2000
21	63,8	0,7	7	2	18	2012
15,2	24,2	0,7	3	2	19	2013
16,2	29,3	0,7	4	2	22	2001
16,2	29,3	0,7	6	2	20	2013
16,7	32,1	0,7	6	2	19	2013
14,7	21,9	0,7	5	2	24	2002
14,7	21,9	0,7	3	2	23	2002
14,7	21,9	0,7	3	2	22	2004
13,1	15,5	0,7	3	2	23	2010

13,1	15,5	0,7	3	2	23	2010
15,8	27,2	0,7	3	2	20	2003
15,3	24,7	0,7	4	2	24	2002
18,1	40,9	0,7	4	2	19	2012
12,6	13,8	0,7	2	2	25	2007
14,4	20,6	0,7	3	2	25	2005
13,4	16,6	0,7	4	2	18	2015
15,4	25,2	0,7	3	2	25	2003
15,4	25,2	0,7	5	2	19	2011
20	55,2	0,7	6	2	15	2015
16,5	31	0,7	6	2	24	2001
15,5	25,7	0,7	5	2	18	1999
15	23,3	0,7	5	2	23	2002
15	23,3	0,7	3	2	26	2003
15	23,3	0,7	7	2	19	2012
11,5	10,5	0,7	2	2	21	2006
16,3	29,9	0,7	5	2	25	2005
19,2	48,9	0,7	10	2	21	2013
13,5	17	0,7	3	2	22	2005

13,5	17	0,7	3	2	25	2005
13,5	17	0,7	2	2	20	2013
17,4	36,4	0,7	5	2	21	2012
14,8	22,4	0,7	3	2	25	2003
14,8	22,4	0,7	4	2	25	2004
10,5	8	0,7	2	2	22	2004
12,5	13,5	0,7	2	2	20	2002
12,5	13,5	0,7	2	2	21	2012
11	9,2	0,7	3	2	23	2005
15,1	23,8	0,7	4	2	24	2006
15,1	23,8	0,7	5	2	24	2009
13,2	15,9	0,7	3	2	25	2007
13,2	15,9	0,7	3	2	20	2008
13,2	15,9	0,7	2	2	18	2014
16,7	32,2	0,7	4	2	21	2003
12,8	14,5	0,7	2	2	26	1999
12,8	14,5	0,7	2	2	27	1999
14,2	19,8	0,7	4	2	27	1999
14,2	19,8	0,7	3	2	17	2008

14,2	19,8	0,7	4	2	22	2011
16,2	29,4	0,7	4	2	18	2002
16,2	29,4	0,7	9	2	23	2004
16,2	29,4	0,7	6	2	19	2011
16,2	29,4	0,7	6	2	19	2013
15,9	27,8	0,7	4	2	21	2008
18,2	41,7	0,7	8	2	18	2012
13,6	17,4	0,7	2	2	24	2002
13	15,2	0,7	3	2	18	1999
13	15,2	0,7	3	2	21	2000
13	15,2	0,7	2	2	21	2012
13	15,2	0,7	2	2	17	2014
15,2	24,3	0,7	4	2	24	2001
17,1	34,6	0,7	4	2	19	2014
17	34	0,7	5	2	21	2013
14,1	19,4	0,7	3	2	21	2012
14,1	19,4	0,7	3	2	17	2014
14,5	21,1	0,7	4	2	21	2012
13,7	17,8	0,7	2	2	22	2000

13,7	17,8	0,7	3	2	23	2004
13,7	17,8	0,7	4	2	21	2012
19,2	49	0,7	6	2	21	2013
20,8	62,3	0,7	8	2	19	2013
12,4	13,2	0,7	3	2	25	2005
15,3	24,8	0,7	4	2	22	2000
15,3	24,8	0,7	4	2	18	2012
15,3	24,8	0,7	4	2	21	2015
15,7	26,8	0,7	3	2	19	2000
13,9	18,6	0,7	2	2	23	2002
15,6	26,3	0,7	6	2	26	2006
15,6	26,3	0,7	4	2	16	2015
11,3	10	0,7	2	2	22	2007
11,7	11,1	0,7	2	2	25	2004
11,7	11,1	0,7	2	2	21	2009
18,9	46,8	0,7	5	2	18	2012
20,6	60,6	0,7	5	2	18	2012
12,7	14,2	0,7	2	2	19	2002
12,7	14,2	0,7	2	2	20	2002

12,7	14,2	0,7	2	2	19	2006
16	28,4	0,7	7	2	20	1999
16	28,4	0,7	5	2	21	2000
16	28,4	0,7	5	2	25	2004
16,4	30,6	0,7	6	2	20	2013
11,8	11,4	0,7	2	2	25	2007
16,2	29,5	0,7	6	2	23	2011
16,2	29,5	0,7	4	2	17	2012
12,2	12,6	0,7	3	2	22	2007
13,1	15,6	0,7	3	2	21	2005
13,1	15,6	0,7	3	2	21	2005
19	47,6	0,7	8	2	21	2015
16,9	33,5	0,7	7	2	17	2012
14,6	21,6	0,7	5	2	20	2013
14,8	22,5	0,7	5	2	25	1999
14,8	22,5	0,7	5	2	25	1999
14,8	22,5	0,7	3	2	23	2015
13,4	16,7	0,7	2	2	21	2000
13,4	16,7	0,7	3	2	23	2007

13,4	16,7	0,7	3	2	22	2015
15,9	27,9	0,7	10	2	23	2007
15,1	23,9	0,7	3	2	26	2003
15,1	23,9	0,7	3	2	19	2012
14,3	20,3	0,7	5	2	25	1999
14,3	20,3	0,7	6	2	22	2003
12,1	12,3	0,7	3	2	25	2004
12,1	12,3	0,7	3	2	23	2005
12,1	12,3	0,7	2	2	21	2006
12,1	12,3	0,7	2	2	23	2006
12,1	12,3	0,7	2	2	25	2007
12	12	0,7	2	2	25	2005
12	12	0,7	2	2	19	2011
12	12	0,7	2	2	21	2014
15,8	27,4	0,7	5	2	24	2006
17,4	36,6	0,7	4	2	16	2015
15,2	24,4	0,7	4	2	23	2005
12,6	13,9	0,7	2	2	19	2000
14,2	19,9	0,7	2	2	23	2002

14,2	19,9	0,7	3	2	26	2003
14,2	19,9	0,7	4	2	17	2008
14,2	19,9	0,7	4	2	23	2008
13,5	17,1	0,7	3	2	25	2007
15,7	26,9	0,7	4	2	23	1999
16,6	31,8	0,7	5	2	20	2001
16,6	31,8	0,7	5	2	24	2001
11,4	10,3	0,7	2	2	23	2004
15,3	24,9	0,7	3	2	21	2013
15,3	24,9	0,7	3	2	15	2015
14,5	21,2	0,7	4	2	19	2000
14,5	21,2	0,7	3	2	22	2015
15,4	25,4	0,7	5	2	26	1999
15,4	25,4	0,7	4	2	21	2013
15,5	25,9	0,7	6	2	19	2013
14,1	19,5	0,7	4	2	25	1999
14,1	19,5	0,7	3	2	25	2003
14,1	19,5	0,7	4	2	22	2011
14,1	19,5	0,7	2	2	20	2013

13,2	16	0,7	4	2	21	2011
13,2	16	0,7	3	2	21	2011
17,2	35,4	0,7	8	2	22	2013
14,7	22,1	0,7	3	2	23	2002
16	28,5	0,7	5	2	21	2000
17,1	34,8	0,7	6	2	17	2012
14	19,1	0,7	2	2	22	2000
14	19,1	0,7	3	2	25	2003
14	19,1	0,7	3	2	25	2007
14	19,1	0,7	3	2	22	2015
17	34,2	0,7	5	2	21	2000
17	34,2	0,7	6	2	20	2003
13,7	17,9	0,7	2	2	24	2002
12,8	14,6	0,7	2	2	22	2013
20	55,7	0,7	10	2	19	2012
13,8	18,3	0,7	5	2	26	1999
13,8	18,3	0,7	3	2	25	2008
13	15,3	0,7	2	2	23	2009
15,9	28	0,7	5	2	19	2012

14,4	20,8	0,7	4	2	21	2006
18,3	42,7	0,7	7	2	19	2014
16,5	31,3	0,7	8	2	23	2007
14,8	22,6	0,7	3	2	19	2012
17,3	36,1	0,7	6	2	19	2014
14,6	21,7	0,7	4	2	21	1999
14,6	21,7	0,7	3	2	24	2001
14,6	21,7	0,7	3	2	21	2014
16,6	31,9	0,7	6	2	20	2013
11,2	9,8	0,7	2	2	22	2004
11,2	9,8	0,7	2	2	25	2007
12,4	13,3	0,7	2	2	25	2007
12,4	13,3	0,7	2	2	25	2007
20,2	57,5	0,7	6	2	19	2012
15,2	24,5	0,7	4	2	20	2011
15,7	27	0,7	3	2	23	2003
19,2	49,4	0,7	10	2	22	2013
15,3	25	0,7	3	2	18	2002
15,3	25	0,7	4	2	18	2008

15,3	25	0,7	3	2	19	2012
15,3	25	0,7	3	2	21	2015
16,8	33,1	0,7	6	2	20	2011
12,7	14,3	0,7	2	2	25	2005
17	34,3	0,7	4	2	20	2003
18,1	41,4	0,7	4	2	18	2012
15,4	25,5	0,7	4	2	20	2013
13,4	16,8	0,7	3	2	23	1999
13,4	16,8	0,7	2	2	25	2008
16	28,6	0,7	4	2	20	2003
11,6	10,9	0,7	1	2	26	2001
18,2	42,1	0,7	6	2	22	2013
13,1	15,7	0,7	3	2	25	2007
13,1	15,7	0,7	3	2	23	2008
14,2	20	0,7	4	2	19	2006
14,2	20	0,7	3	2	18	2012
14,2	20	0,7	4	2	15	2015
10,8	8,8	0,7	2	2	23	2004
12,3	13	0,7	2	2	21	2006

12,3	13	0,7	2	2	26	2006
11	9,3	0,7	2	2	22	2004
12,9	15	0,7	3	2	21	2000
10,1	7,2	0,7	1	2	25	2005
17,6	38,1	0,7	8	2	23	2015
15,9	28,1	0,7	4	2	25	2004
13,5	17,2	0,7	3	2	22	1999
13,5	17,2	0,7	2	2	24	2002
15	23,6	0,7	5	2	25	1999
15	23,6	0,7	5	2	21	2000
11,7	11,2	0,7	2	2	25	2004
11,7	11,2	0,7	2	2	25	2008
12,2	12,7	0,7	3	2	21	2005
12,2	12,7	0,7	3	2	23	2005
12,2	12,7	0,7	2	2	23	2007
19,8	54,3	0,7	6	2	20	2013
16,3	30,3	0,7	6	2	20	2013
13,6	17,6	0,7	4	2	25	2008
10,5	8,1	0,7	2	2	25	2004

14	19,2	0,7	4	2	21	2006
17,5	37,5	0,7	6	2	21	2013
15,8	27,6	0,7	5	2	19	2012
19	48	0,7	5	2	18	2012
12,6	14	0,7	2	2	20	2013
12,1	12,4	0,7	2	2	25	2007
17,1	35	0,7	5	2	19	2000
11,3	10,1	0,7	2	2	25	2004
11,3	10,1	0,7	2	2	25	2004
15,1	24,1	0,7	10	2	26	2001
13,7	18	0,7	3	2	26	2000
13,7	18	0,7	3	2	19	2012
13,7	18	0,7	3	2	22	2015
13,9	18,8	0,7	4	2	26	2009
13,8	18,4	0,7	4	2	21	2006
14,8	22,7	0,7	4	2	20	1999
15,7	27,1	0,7	4	2	21	2003
14,6	21,8	0,7	4	2	27	1999
14,6	21,8	0,7	3	2	23	2003

15,6	26,6	0,7	4	2	27	1999
15,6	26,6	0,7	8	2	23	2015
15,3	25,1	0,7	7	2	23	1999
15,3	25,1	0,7	5	2	24	2002
16,2	29,8	0,7	4	2	24	2002
16,2	29,8	0,7	4	2	17	2012
15,4	25,6	0,7	6	2	19	2013
12,8	14,7	0,7	2	2	25	2005
13	15,4	0,7	2	2	22	2002
13	15,4	0,7	2	2	23	2002
13	15,4	0,7	5	2	23	2007
14,3	20,5	0,7	3	2	23	2010
14,3	20,5	0,7	3	2	23	2010
13,3	16,5	0,7	3	2	25	1999
13,3	16,5	0,7	2	2	27	1999
12,5	13,7	0,7	3	2	22	2005
15,9	28,2	0,7	5	2	17	2012
18,3	43	0,7	6	2	19	2013
14,5	21,4	0,7	3	2	26	2000

16,3	30,4	0,7	10	2	19	2011
14,2	20,1	0,7	6	2	21	2008
14,2	20,1	0,7	3	2	20	2012
10,7	8,6	0,7	2	2	22	2004
14,7	22,3	0,7	2	2	26	2000
14,7	22,3	0,7	4	2	25	2005
15	23,7	0,7	5	2	23	1999
15,8	27,7	0,7	4	2	21	2013
16,8	33,3	0,7	10	2	21	2013
19,7	53,7	0,7	8	2	20	2013
14,1	19,7	0,7	5	2	24	2009
14,1	19,7	0,7	4	2	19	2011
16,4	31	0,7	4	2	18	2012
12,4	13,4	0,7	2	2	25	2007
13,1	15,8	0,7	3	2	21	2015
15,7	27,2	0,7	4	2	18	2000
15,7	27,2	0,7	4	2	22	2013
12,7	14,4	0,7	6	2	25	2008
17,3	36,4	0,7	5	2	19	2013

21,4	68,9	0,7	9	2	20	2013
13,5	17,3	0,7	2	2	21	2000
14,8	22,8	0,7	4	2	24	2006
15,2	24,7	0,7	3	2	26	2003
15,2	24,7	0,7	7	2	19	2014
14	19,3	0,7	2	2	27	1999
14	19,3	0,7	3	2	21	2000
14	19,3	0,7	3	2	25	2007
12,9	15,1	0,7	2	2	25	2007
12,9	15,1	0,7	2	2	24	2009
17,5	37,7	0,7	4	2	19	2013
16,5	31,6	0,7	5	2	16	2015
15,5	26,2	0,7	3	2	18	2012
13,6	17,7	0,7	3	2	25	2002
14,6	21,9	0,7	3	2	21	2012
20	56,3	0,7	9	2	18	2015
13,7	18,1	0,7	3	2	24	2001
17,8	39,7	0,7	7	2	22	2001
13,2	16,2	0,7	2	2	21	2000

13,2	16,2	0,7	3	2	23	2005
13,2	16,2	0,7	5	2	21	2006
17,9	40,4	0,7	5	2	19	2012
16,1	29,4	0,7	6	2	19	2014
12,6	14,1	0,7	2	2	19	2000
18,3	43,2	0,7	6	2	22	2013
20	56,4	0,7	7	2	18	2012
16,4	31,1	0,7	8	2	22	1999
16,4	31,1	0,7	6	2	17	2012
15	23,8	0,7	4	2	17	2012
14,5	21,5	0,7	5	2	25	2005
14,2	20,2	0,7	4	2	20	2011
13	15,5	0,7	3	2	25	2007
11,7	11,3	0,7	2	2	21	2009
16	28,9	0,7	7	2	19	2014
13,3	16,6	0,7	5	2	26	1999
13,3	16,6	0,7	3	2	25	2005
13,3	16,6	0,7	3	2	21	2014
13,3	16,6	0,7	3	2	23	2014

12,1	12,5	0,7	2	2	19	2000
16,2	30	0,7	5	2	25	2002
16,2	30	0,7	5	2	21	2012
16,5	31,7	0,7	4	2	21	2003
16,5	31,7	0,7	6	2	22	2004
12,8	14,8	0,7	2	2	22	2002
12,8	14,8	0,7	3	2	23	2010
12,8	14,8	0,7	3	2	23	2010
12,8	14,8	0,7	2	2	22	2013
11,8	11,6	0,7	2	2	19	2011
12	12,2	0,7	3	2	21	2011
15,2	24,8	0,7	5	2	26	1999
15,5	26,3	0,7	4	2	22	2000
17	34,7	0,7	6	2	22	2013
14,1	19,8	0,7	5	2	19	2000
14,1	19,8	0,7	4	2	22	2004
15,3	25,3	0,7	6	2	25	1999
16,7	32,9	0,7	4	2	22	2002
14,8	22,9	0,7	4	2	20	2011

14,8	22,9	0,7	4	2	21	2012
18	41,2	0,7	7	2	17	2012
13,4	17	0,7	4	2	23	1999
12,5	13,8	0,7	2	2	24	2006
12,5	13,8	0,7	2	2	25	2007
14,6	22	0,7	5	2	23	1999
14,6	22	0,7	4	2	23	1999
14,6	22	0,7	3	2	19	2012
14	19,4	0,7	6	2	20	2008
14	19,4	0,7	4	2	21	2008
13,5	17,4	0,7	5	2	26	1999
13,5	17,4	0,7	2	2	22	2000
13,1	15,9	0,7	2	2	24	2009
13,1	15,9	0,7	3	2	18	2015
16,4	31,2	0,7	4	2	26	2001
16,4	31,2	0,7	6	2	20	2011
14,9	23,4	0,7	4	2	25	1999
13,9	19	0,7	2	2	22	2000
13,9	19	0,7	2	2	19	2000

17,2	36	0,7	5	2	19	2012
18,5	44,8	0,7	6	2	19	2013
13,6	17,8	0,7	2	2	27	1999
13,8	18,6	0,7	3	2	22	2000
13,8	18,6	0,7	3	2	20	2002
13,7	18,2	0,7	3	2	25	1999
13,7	18,2	0,7	3	2	25	2005
13,7	18,2	0,7	3	2	25	2005
12,7	14,5	0,7	2	2	24	2009
14,3	20,7	0,7	5	2	27	1999
14,3	20,7	0,7	5	2	23	2002
14,3	20,7	0,7	3	2	19	2008
16	29	0,7	6	2	19	2013
15	23,9	0,7	3	2	18	2012
14,7	22,5	0,7	4	2	23	2004
14,5	21,6	0,7	3	2	21	2000
15,6	26,9	0,7	3	2	19	2012
15,1	24,4	0,7	4	2	22	2013
13,2	16,3	0,7	2	2	18	2002

18,8	47,1	0,7	5	2	18	2012
16,3	30,7	0,7	4	2	15	2015
15,5	26,4	0,7	6	2	22	2013
14,2	20,3	0,7	5	2	26	2000
15,2	24,9	0,7	5	2	22	2007
15,2	24,9	0,7	3	2	19	2011
17,5	38	0,7	6	2	20	2013
15,3	25,4	0,7	5	2	19	2012
12,3	13,2	0,7	2	2	25	2002
14,8	23	0,7	3	2	21	2012
16,4	31,3	0,7	7	2	20	2006
13,3	16,7	0,7	2	2	22	2002
13,3	16,7	0,7	3	2	21	2008
12,6	14,2	0,7	2	2	20	2000
18,2	42,8	0,7	6	2	20	2013
14,4	21,2	0,7	5	2	18	2000
13	15,6	0,7	2	2	20	2003
13	15,6	0,7	3	2	25	2005
16,5	31,9	0,7	6	2	24	2001

16,2	30,2	0,7	5	2	22	2011
17	34,9	0,7	6	2	20	2013
12,2	12,9	0,7	3	2	23	2007
12,2	12,9	0,7	2	2	25	2007
16	29,1	0,7	6	2	20	2011
16	29,1	0,7	6	2	20	2013
15,7	27,5	0,7	5	2	25	2007
14	19,5	0,7	5	2	25	1999
14	19,5	0,7	3	2	22	2000
14	19,5	0,7	3	2	25	2002
14	19,5	0,7	4	2	19	2009
13,4	17,1	0,7	2	2	23	2002
13,4	17,1	0,7	3	2	25	2007
13,9	19,1	0,7	5	2	25	2007
15,6	27	0,7	6	2	25	1999
13,5	17,5	0,7	6	2	20	2011
14,3	20,8	0,7	6	2	18	2000
14,7	22,6	0,7	5	2	22	1999
15,9	28,6	0,7	5	2	19	2012

15,1	24,5	0,7	5	2	25	2007
13,6	17,9	0,7	2	2	23	2002
15,5	26,5	0,7	5	2	19	2000
16,1	29,7	0,7	5	2	19	2012
12,5	13,9	0,7	3	2	21	2005
12,5	13,9	0,7	2	2	22	2011
13,7	18,3	0,7	3	2	20	2002
13,7	18,3	0,7	2	2	25	2008
11,2	10	0,7	3	2	23	2005
11,7	11,4	0,7	2	2	22	2011
14,5	21,7	0,7	3	2	23	2015
12	12,3	0,7	3	2	25	2005
12	12,3	0,7	3	2	25	2005
12	12,3	0,7	2	2	25	2007
12	12,3	0,7	2	2	21	2009
12	12,3	0,7	2	2	22	2011
19,9	56,1	0,7	8	2	17	2012
15,2	25	0,7	3	2	21	2000
15,4	26	0,7	4	2	22	2011

15,3	25,5	0,7	5	2	18	2000
14,2	20,4	0,7	4	2	23	2002
14,2	20,4	0,7	4	2	23	2009
14,2	20,4	0,7	3	2	18	2012
14,2	20,4	0,7	4	2	22	2013
16,2	30,3	0,7	6	2	20	2013
12,7	14,6	0,7	2	2	25	1999
12,7	14,6	0,7	2	2	22	2002
16	29,2	0,7	6	2	21	2013
15,7	27,6	0,7	4	2	22	2001
18,2	43	0,7	6	2	19	2013
12,4	13,6	0,7	2	2	18	2002
12,4	13,6	0,7	2	2	25	2005
14,4	21,3	0,7	6	2	25	1999
14,6	22,2	0,7	3	2	22	2011
20,8	64,2	0,7	7	2	18	2012
14,1	20	0,7	4	2	23	2005
16,3	30,9	0,7	5	2	21	2012
16,3	30,9	0,7	5	2	22	2013

20	57,1	0,7	6	2	22	2013
15	24,1	0,7	6	2	19	2000
13,3	16,8	0,7	5	2	25	2007
17,7	39,6	0,7	7	2	20	2013
14	19,6	0,7	2	2	24	2002
14	19,6	0,7	7	2	20	2012
15,1	24,6	0,7	5	2	20	1999
15,1	24,6	0,7	3	2	21	2014
15,1	24,6	0,7	3	2	23	2014
17,3	37	0,7	4	2	18	2012
13	15,7	0,7	3	2	25	2007
14,3	20,9	0,7	5	2	26	2000
14,3	20,9	0,7	3	2	24	2002
14,3	20,9	0,7	4	2	21	2005
14,3	20,9	0,7	3	2	19	2012
15,2	25,1	0,7	4	2	24	2006
15,3	25,6	0,7	4	2	19	1999
13,9	19,2	0,7	2	2	27	1999
18,2	43,1	0,7	7	2	18	2012

15,8	28,2	0,7	4	2	18	2000
14,5	21,8	0,7	4	2	22	2011
16	29,3	0,7	4	2	25	2002
16	29,3	0,7	5	2	19	2012
13,5	17,6	0,7	2	2	24	2002
13,8	18,8	0,7	2	2	27	1999
13,8	18,8	0,7	4	2	26	2005
13,6	18	0,7	2	2	18	1999
13,7	18,4	0,7	3	2	27	1999
14,8	23,2	0,7	4	2	24	2002
12,2	13	0,7	3	2	25	2007
19,1	49,9	0,7	8	2	19	2013
13,1	16,1	0,7	3	2	20	2011
17,8	40,4	0,7	6	2	19	2000
14,6	22,3	0,7	4	2	23	2002
14,4	21,4	0,7	2	2	24	2002
11,5	10,9	0,7	2	2	25	2004
12,5	14	0,7	2	2	26	1999
12,5	14	0,7	2	2	25	2007

12,5	14	0,7	3	2	23	2008
16,9	34,6	0,7	6	2	19	2011
12,1	12,7	0,7	2	2	25	2003
15,5	26,7	0,7	10	2	23	2002
15,5	26,7	0,7	4	2	19	2011
14,1	20,1	0,7	3	2	21	2000
14,1	20,1	0,7	3	2	24	2001
14,1	20,1	0,7	4	2	22	2002
14,1	20,1	0,7	3	2	24	2002
15	24,2	0,7	3	2	25	2002
16,6	32,8	0,7	4	2	18	2015
12,9	15,4	0,7	2	2	22	2002
15,8	28,3	0,7	3	2	21	2003
11,6	11,2	0,7	2	2	25	2004
15,3	25,7	0,7	5	2	19	2006
15,2	25,2	0,7	5	2	19	2012
15,2	25,2	0,7	4	2	20	2013
12	12,4	0,7	2	2	23	2007
12,7	14,7	0,7	3	2	25	2007

14	19,7	0,7	4	2	19	2011
17,1	35,9	0,7	3	2	18	2012
11,7	11,5	0,7	2	2	26	2006
14,3	21	0,7	5	2	27	1999
14,3	21	0,7	3	2	26	2000
14,3	21	0,7	3	2	25	2002
14,3	21	0,7	4	2	23	2002
14,3	21	0,7	4	2	25	2004
13,3	16,9	0,7	3	2	25	2004
14,5	21,9	0,7	3	2	26	2000
14,5	21,9	0,7	3	2	24	2002
14,5	21,9	0,7	4	2	19	2011
18,1	42,6	0,7	7	2	18	2012
15,9	28,9	0,7	4	2	22	2011
15,9	28,9	0,7	5	2	21	2013
16,5	32,3	0,7	4	2	18	2012
15,6	27,3	0,7	3	2	24	2001
13,8	18,9	0,7	4	2	26	2009
16,6	32,9	0,7	6	2	19	2013

13,5	17,7	0,7	3	2	18	2000
14,2	20,6	0,7	3	2	21	2012
13,7	18,5	0,7	2	2	27	1999
14,9	23,8	0,7	3	2	27	1999
14,9	23,8	0,7	4	2	26	2005
18,6	46,3	0,7	6	2	21	2013
17,7	39,9	0,7	6	2	20	2013
14,6	22,4	0,7	5	2	23	2007
12,6	14,4	0,7	2	2	22	2011
18,2	43,4	0,7	6	2	17	2012
17,1	36	0,7	5	2	21	2012
15	24,3	0,7	4	2	20	2006
15	24,3	0,7	4	2	24	2006
12,3	13,4	0,7	3	2	22	2005
12,3	13,4	0,7	2	2	26	2006
18	42	0,7	8	2	21	2013
16	29,5	0,7	9	2	19	2000
15,2	25,3	0,7	5	2	20	2011
16,3	31,2	0,7	5	2	19	2000

14,1	20,2	0,7	2	2	18	2000
14,1	20,2	0,7	5	2	21	2000
14,1	20,2	0,7	5	2	26	2000
14,1	20,2	0,7	4	2	23	2006
14,1	20,2	0,7	4	2	21	2009
14,1	20,2	0,7	3	2	15	2015
16,4	31,8	0,7	6	2	24	2002
12,2	13,1	0,7	2	2	25	2003
12,2	13,1	0,7	2	2	25	2007
14	19,8	0,7	2	2	24	2002
14	19,8	0,7	3	2	23	2002
14	19,8	0,7	4	2	21	2011
14,5	22	0,7	3	2	23	2003
14,5	22	0,7	4	2	19	2012
14,5	22	0,7	3	2	21	2012
13,2	16,6	0,7	3	2	23	1999
13,2	16,6	0,7	3	2	23	2007
18,7	47,2	0,7	8	2	21	2013
14,8	23,4	0,7	4	2	20	1999

14,8	23,4	0,7	5	2	21	2000
12,5	14,1	0,7	2	2	25	2008
12,9	15,5	0,7	2	2	19	2013
13,9	19,4	0,7	5	2	21	2000
13,9	19,4	0,7	2	2	26	2000
12,7	14,8	0,7	4	2	27	1999
12,7	14,8	0,7	2	2	24	2009
16	29,6	0,7	6	2	24	2006
16	29,6	0,7	4	2	19	2011
14,2	20,7	0,7	3	2	19	2012
15	24,4	0,7	4	2	23	2006
13,8	19	0,7	3	2	19	2012
16,9	34,9	0,7	9	2	22	2001
15,3	25,9	0,7	5	2	18	2000
15,3	25,9	0,7	4	2	18	2012
13,4	17,4	0,7	3	2	25	2008
11,5	11	0,7	2	2	23	2005
15,2	25,4	0,7	5	2	19	1999
19,3	52	0,7	7	2	18	2012

18,7	47,3	0,7	5	2	18	2012
14,4	21,6	0,7	4	2	25	1999
12	12,5	0,7	2	2	22	2000
12	12,5	0,7	2	2	23	2010
12	12,5	0,7	2	2	23	2010
18	42,2	0,7	6	2	20	2013
13	15,9	0,7	3	2	25	2005
15,9	29,1	0,7	6	2	19	2006
11,6	11,3	0,7	2	2	20	2002
14,7	23	0,7	5	2	23	1999
11,7	11,6	0,7	2	2	20	2013
19	49,7	0,7	5	2	18	2012
17	35,6	0,7	6	2	21	2013
18,2	43,7	0,7	4	2	18	2012
14,8	23,5	0,7	5	2	27	1999
14,5	22,1	0,7	4	2	21	2006
14,5	22,1	0,7	4	2	20	2011
14,5	22,1	0,7	4	2	22	2011
13,1	16,3	0,7	3	2	23	2005

16	29,7	0,7	4	2	22	2001
14	19,9	0,7	5	2	23	1999
14	19,9	0,7	3	2	20	2003
14	19,9	0,7	4	2	26	2009
14	19,9	0,7	4	2	21	2012
16,8	34,4	0,7	5	2	18	2012
15,4	26,5	0,7	4	2	19	2012
16,5	32,6	0,7	8	2	25	1999
16,5	32,6	0,7	5	2	19	2012
15,3	26	0,7	5	2	21	1999
15,3	26	0,7	4	2	26	2005
13,2	16,7	0,7	3	2	22	2002
15,7	28,1	0,7	4	2	20	2011
14,6	22,6	0,7	4	2	20	2011
17	35,7	0,7	5	2	20	2000
14,4	21,7	0,7	8	2	21	1999
14,4	21,7	0,7	8	2	26	1999
16,2	30,9	0,7	7	2	19	2012
13,3	17,1	0,7	5	2	26	1999

13,3	17,1	0,7	4	2	26	1999
12,5	14,2	0,7	2	2	26	2001
16,9	35,1	0,7	7	2	19	2012
14,7	23,1	0,7	3	2	22	2004
13,7	18,7	0,7	4	2	21	1999
13,4	17,5	0,7	3	2	25	2004
13,4	17,5	0,7	4	2	25	2005
12,7	14,9	0,7	2	2	25	1999
12,7	14,9	0,7	6	2	25	2008
13,6	18,3	0,7	4	2	19	2006
13,6	18,3	0,7	3	2	23	2007
13,6	18,3	0,7	3	2	25	2007
16	29,8	0,7	4	2	20	2003
19,1	50,7	0,7	8	2	19	2013
11,3	10,5	0,7	2	2	20	2004
16,7	33,9	0,7	6	2	20	2013
17,3	37,7	0,7	7	2	19	2011
17,3	37,7	0,7	5	2	19	2012
14,5	22,2	0,7	2	2	23	2002

14,5	22,2	0,7	6	2	20	2011
13	16	0,7	3	2	25	2007
13	16	0,7	3	2	25	2007
13	16	0,7	3	2	21	2008
15,4	26,6	0,7	5	2	25	1999
15,4	26,6	0,7	6	2	17	2012
14,3	21,3	0,7	3	2	17	2012
16,1	30,4	0,7	10+	2	17	2014
18,1	43,2	0,7	5	2	21	2013
15,3	26,1	0,7	4	2	18	2012
18	42,5	0,7	6	2	15	2015
11	9,7	0,7	2	2	20	2009
17,9	41,8	0,7	10	2	18	2012
14	20	0,7	4	2	24	2009
15	24,6	0,7	4	2	20	2008
15,1	25,1	0,7	3	2	25	2002
12,4	13,9	0,7	3	2	25	2007
16,9	35,2	0,7	5	2	22	2013
14,6	22,7	0,7	5	2	25	2007

13,1	16,4	0,7	2	2	26	2006
16,3	31,6	0,7	5	2	17	2008
18,2	44	0,7	6	2	21	2015
16	29,9	0,7	5	2	18	2012
16	29,9	0,7	8	2	20	2013
16,4	32,2	0,7	4	2	17	2008
14,4	21,8	0,7	5	2	19	2000
14,4	21,8	0,7	4	2	15	2015
16,5	32,8	0,7	5	2	25	1999
14,7	23,2	0,7	5	2	25	1999
18,6	47	0,7	10	2	20	2013
15,5	27,2	0,7	5	2	21	2000
13,2	16,8	0,7	3	2	25	2005
18	42,6	0,7	6	2	20	2013
13,8	19,2	0,7	2	2	26	2000
12,3	13,6	0,7	2	2	25	1999
12,3	13,6	0,7	2	2	21	2008
13,3	17,2	0,7	2	2	24	2009
13,7	18,8	0,7	4	2	24	2002

17,5	39,2	0,7	5	2	21	2012
14,5	22,3	0,7	5	2	19	2000
13,6	18,4	0,7	3	2	15	2015
16,2	31,1	0,7	6	2	22	2013
15,3	26,2	0,7	6	2	20	2011
15,3	26,2	0,7	3	2	15	2015
13,5	18	0,7	3	2	23	1999
13,5	18	0,7	2	2	26	2001
13,5	18	0,7	3	2	25	2005
15,2	25,7	0,7	5	2	22	1999
15,1	25,2	0,7	5	2	19	2000
10,3	8	0,7	2	2	23	2004
16,7	34,1	0,7	5	2	23	2002
16,4	32,3	0,7	5	2	21	2014
12,7	15	0,7	3	2	25	2008
12,7	15	0,7	3	2	22	2011
20,5	63,1	0,7	7	2	21	2012
12,2	13,3	0,7	3	2	25	2005
14	20,1	0,7	5	2	18	1999

14	20,1	0,7	4	2	25	1999
14	20,1	0,7	2	2	25	2002
14,6	22,8	0,7	5	2	22	1999
18,3	44,9	0,7	6	2	19	2013
13	16,1	0,7	2	2	20	2013
15,5	27,3	0,7	4	2	20	2010
15,5	27,3	0,7	4	2	20	2010
14,4	21,9	0,7	4	2	23	2011
18,1	43,5	0,7	7	2	18	2012
17,6	40	0,7	6	2	20	2013
16,2	31,2	0,7	6	2	21	2012
13,1	16,5	0,7	5	2	23	2006
17,1	36,7	0,7	6	2	19	2013
17,8	41,4	0,7	5	2	19	2012
10,5	8,5	0,7	2	2	23	2004
12,4	14	0,7	2	2	21	2000
16,7	34,2	0,7	5	2	24	2001
15,3	26,3	0,7	4	2	17	2012
13,8	19,3	0,7	3	2	19	2012

14,9	24,3	0,7	3	2	26	2003
11,3	10,6	0,7	3	2	25	2005
15,2	25,8	0,7	4	2	19	2012
14,5	22,4	0,7	5	2	27	1999
14,5	22,4	0,7	4	2	21	2011
15	24,8	0,7	5	2	22	2002
15	24,8	0,7	3	2	24	2002
12,6	14,7	0,7	2	2	25	1999
15,6	27,9	0,7	5	2	19	2012
12	12,7	0,7	2	2	19	2011
18,7	48,1	0,7	4	2	18	2012
13,5	18,1	0,7	3	2	23	2002
13,5	18,1	0,7	4	2	24	2009
11,4	10,9	0,7	2	2	25	2004
15,5	27,4	0,7	4	2	20	2013
14,6	22,9	0,7	6	2	20	2013
18,5	46,6	0,7	10+	2	17	2014
15,9	29,6	0,7	5	2	19	2000
11,5	11,2	0,7	2	2	25	2005

11,8	12,1	0,7	2	2	25	2002
11,7	11,8	0,7	2	2	25	2007
14,4	22	0,7	3	2	23	2008
12,7	15,1	0,7	2	2	21	2011
15,2	25,9	0,7	6	2	21	1999
15,2	25,9	0,7	5	2	23	1999
15,2	25,9	0,7	4	2	25	2002
19	50,6	0,7	10	2	17	2012
15,8	29,1	0,7	5	2	19	2012
17,3	38,2	0,7	7	2	18	2012
12,2	13,4	0,7	2	2	19	2006
12,2	13,4	0,7	3	2	25	2008
17,1	36,9	0,7	5	2	17	2012
13,1	16,6	0,7	3	2	24	2002
13,1	16,6	0,7	2	2	24	2002
15,5	27,5	0,7	5	2	22	1999
15,5	27,5	0,7	5	2	25	1999
16,3	32	0,7	6	2	19	2011
15,7	28,6	0,7	4	2	21	2012

14,6	23	0,7	5	2	25	1999
16,5	33,2	0,7	6	2	19	2011
13,2	17	0,7	2	2	27	1999
15,4	27	0,7	5	2	26	2000
12,1	13,1	0,7	2	2	25	2007
13,4	17,8	0,7	5	2	26	2006
14	20,3	0,7	5	2	18	1999
14	20,3	0,7	2	2	27	1999
14	20,3	0,7	3	2	19	2012
15,3	26,5	0,7	4	2	25	2008
14,4	22,1	0,7	5	2	21	1999
14,4	22,1	0,7	4	2	22	2000
15,6	28,1	0,7	6	2	21	2001
15,8	29,2	0,7	5	2	23	2011
15,8	29,2	0,7	5	2	19	2011
15,2	26	0,7	4	2	25	1999
15,2	26	0,7	7	2	17	2012
15,1	25,5	0,7	4	2	23	2006
15,1	25,5	0,7	4	2	20	2012

12,9	15,9	0,7	2	2	22	2013
18,5	46,9	0,7	8	2	21	2013
15	25	0,7	6	2	26	2001
16,2	31,5	0,7	7	2	21	2013
14,5	22,6	0,7	4	2	27	1999
15,9	29,8	0,7	5	2	23	2003
12,3	13,8	0,7	2	2	21	2000
12,3	13,8	0,7	3	2	26	2005
15,7	28,7	0,7	4	2	24	2002
11,9	12,5	0,7	3	2	19	2006
13	16,3	0,7	2	2	26	2000
13	16,3	0,7	2	2	26	2009
14,1	20,8	0,7	5	2	25	2007
14,3	21,7	0,7	2	2	26	2000
12,5	14,5	0,7	2	2	25	2007
15,3	26,6	0,7	7	2	22	2007
17,9	42,6	0,7	8	2	20	2013
15,6	28,2	0,7	5	2	22	2013
13,1	16,7	0,7	2	2	23	2005

14,7	23,6	0,7	7	2	22	1999
15,2	26,1	0,7	4	2	23	2002
14,4	22,2	0,7	2	2	21	2000
16,3	32,2	0,7	6	2	22	2011
16,3	32,2	0,7	6	2	21	2012
16,5	33,4	0,7	4	2	21	2012
15,1	25,6	0,7	5	2	25	1999
15	25,1	0,7	4	2	26	2006
15	25,1	0,7	5	2	20	2012
13,3	17,5	0,7	2	2	26	1999
15,5	27,7	0,7	6	2	19	2011
15,4	27,2	0,7	4	2	19	2006
12,4	14,2	0,7	3	2	22	2005
12,1	13,2	0,7	2	2	22	2011
12,9	16	0,7	2	2	25	2002
15,3	26,7	0,7	4	2	17	2012
14,3	21,8	0,7	3	2	25	2002
13,8	19,6	0,7	2	2	25	2002
14,7	23,7	0,7	5	2	25	1999

15,1	25,7	0,7	5	2	27	1999
15,1	25,7	0,7	4	2	25	2007
19	51,2	0,7	5	2	20	2013
13	16,4	0,7	4	2	26	2009
14,8	24,2	0,7	5	2	19	2012
15	25,2	0,7	8	2	26	1999
13,7	19,2	0,7	3	2	21	2000
13,7	19,2	0,7	3	2	22	2002
13,7	19,2	0,7	3	2	21	2012
15,7	28,9	0,7	6	2	20	2011
12,7	15,3	0,7	2	2	21	2000
14	20,5	0,7	5	2	25	1999
14	20,5	0,7	3	2	23	2007
14	20,5	0,7	4	2	25	2007
13,1	16,8	0,7	2	2	23	2002
13,1	16,8	0,7	3	2	23	2005
15,4	27,3	0,7	4	2	20	2011
12,5	14,6	0,7	2	2	22	2009
13,3	17,6	0,7	2	2	25	2003

13,4	18	0,7	3	2	25	2005
13,4	18	0,7	4	2	25	2008
13,4	18	0,7	4	2	22	2011
15,3	26,8	0,7	4	2	17	2012
13,9	20,1	0,7	2	2	25	2002
16,8	35,5	0,7	5	2	18	2000
14,3	21,9	0,7	5	2	26	1999
14,3	21,9	0,7	2	2	21	2000
14,1	21	0,7	3	2	25	2002
14,7	23,8	0,7	3	2	22	2011
16,7	34,9	0,7	6	2	20	2013
15,1	25,8	0,7	4	2	25	1999
20,2	61,8	0,7	5	2	18	2012
10	7,5	0,8	2	2	23	2004
16,1	31,3	0,8	9	2	24	2009
12,4	14,3	0,8	2	2	25	2008
15,4	27,4	0,8	6	2	19	2011
16,3	32,5	0,8	6	2	23	2011
14	20,6	0,8	2	2	26	2000

14,2	21,5	0,8	4	2	23	1999
13	16,5	0,8	2	2	22	2000
13	16,5	0,8	2	2	25	2005
13	16,5	0,8	3	2	26	2005
17	36,9	0,8	10	2	19	2012
15,9	30,2	0,8	7	2	25	2007
13,6	18,9	0,8	2	2	21	2000
15,2	26,4	0,8	5	2	20	1999
18,5	47,6	0,8	6	2	21	2013
13,2	17,3	0,8	2	2	26	1999
13,2	17,3	0,8	3	2	26	1999
13,4	18,1	0,8	2	2	21	2000
13,4	18,1	0,8	2	2	26	2000
12	13	0,8	2	2	25	2007
19,8	58,4	0,8	5	2	18	2012
13,3	17,7	0,8	2	2	22	2011
14,7	23,9	0,8	4	2	26	1999
14,7	23,9	0,8	6	2	27	1999
16,4	33,2	0,8	4	2	24	2002

18	43,9	0,8	10	2	19	2012
15,4	27,5	0,8	5	2	26	2000
11,1	10,3	0,8	2	2	25	2007
13,8	19,8	0,8	3	2	24	2002
17,8	42,5	0,8	9	2	19	2013
11,9	12,7	0,8	3	2	26	2005
15,3	27	0,8	5	2	21	2012
17,6	41,1	0,8	7	2	18	2012
14,2	21,6	0,8	9	2	25	1999
14,2	21,6	0,8	6	2	25	1999
14,2	21,6	0,8	3	2	25	2002
14,5	23	0,8	4	2	26	2009
19,2	53,4	0,8	7	2	17	2012
12,2	13,7	0,8	2	2	22	2002
16,5	33,9	0,8	6	2	21	2013
12,6	15,1	0,8	3	2	24	2002
17,9	43,3	0,8	7	2	17	2012
16,2	32,1	0,8	4	2	21	2012
12,4	14,4	0,8	2	2	19	2009

13	16,6	0,8	4	2	22	2002
17,5	40,5	0,8	5	2	20	2013
13,9	20,3	0,8	5	2	18	2012
11,4	11,2	0,8	2	2	22	2002
13,1	17	0,8	2	2	25	2002
13,1	17	0,8	2	2	25	2008
14,1	21,2	0,8	5	2	23	1999
13,2	17,4	0,8	4	2	21	2006
15,3	27,1	0,8	4	2	22	2002
12,7	15,5	0,8	2	2	22	2002
16	31	0,8	5	2	20	2000
14,4	22,6	0,8	4	2	19	2000
15,7	29,3	0,8	4	2	19	2011
15,5	28,2	0,8	5	2	21	2000
15,5	28,2	0,8	5	2	21	2012
16,2	32,2	0,8	6	2	19	2012
14	20,8	0,8	4	2	26	2006
15,1	26,1	0,8	4	2	21	1999
14,6	23,6	0,8	3	2	23	2003

14,6	23,6	0,8	6	2	25	2005
13,7	19,5	0,8	3	2	22	2000
15	25,6	0,8	4	2	18	2002
15,6	28,8	0,8	4	2	20	2011
14,7	24,1	0,8	3	2	25	2002
20	60,7	0,8	10	2	18	2012
14,9	25,1	0,8	4	2	27	1999
14,9	25,1	0,8	4	2	21	2000
20,9	69,3	0,8	10	2	22	2013
16	31,1	0,8	5	2	22	2002
13,6	19,1	0,8	4	2	19	2006
17,5	40,7	0,8	6	2	19	2013
14,1	21,3	0,8	3	2	25	2002
14,1	21,3	0,8	3	2	25	2004
14,1	21,3	0,8	3	2	19	2011
12,2	13,8	0,8	3	2	25	2005
13,5	18,7	0,8	2	2	21	2000
13,2	17,5	0,8	4	2	25	1999
20,4	64,6	0,8	10	2	21	2013

15,6	28,9	0,8	4	2	23	2002
17	37,4	0,8	7	2	19	2012
18	44,4	0,8	6	2	19	2013
14,2	21,8	0,8	4	2	22	2004
14,2	21,8	0,8	4	2	22	2011
12,7	15,6	0,8	2	2	27	1999
14	20,9	0,8	3	2	19	2011
11,7	12,2	0,8	3	2	20	2004
23,4	97,6	0,8	9	2	18	2012
14,7	24,2	0,8	3	2	21	2000
17,9	43,7	0,8	6	2	18	2012
16,1	31,8	0,8	6	2	25	1999
13,7	19,6	0,8	2	2	22	2002
18,1	45,2	0,8	6	2	20	2013
18,4	47,5	0,8	7	2	18	2012
14,3	22,3	0,8	6	2	26	1999
11,4	11,3	0,8	2	2	20	2009
12,3	14,2	0,8	2	2	22	2002
15,8	30,1	0,8	5	2	19	2012

14,1	21,4	0,8	3	2	17	2008
16,5	34,3	0,8	5	2	20	2000
16	31,3	0,8	7	2	19	2012
15	25,8	0,8	6	2	22	2011
16,9	36,9	0,8	5	2	21	2013
13	16,8	0,8	3	2	21	2011
13,4	18,4	0,8	2	2	25	2002
13,8	20,1	0,8	2	2	27	1999
14,9	25,3	0,8	6	2	27	1999
13,1	17,2	0,8	3	2	25	2007
13,3	18	0,8	3	2	21	2000
13,3	18	0,8	3	2	22	2002
13,3	18	0,8	2	2	25	2002
17,1	38,3	0,8	5	2	19	2000
13,7	19,7	0,8	3	2	22	2002
15,9	30,8	0,8	4	2	19	2011
15,9	30,8	0,8	5	2	19	2012
16,4	33,8	0,8	4	2	20	2003
18,3	47	0,8	7	2	19	2012

13,6	19,3	0,8	2	2	27	1999
13,6	19,3	0,8	4	2	22	2002
16,6	35,1	0,8	7	2	19	2012
18,5	48,6	0,8	8	2	20	2013
12,1	13,6	0,8	3	2	23	2005
15,3	27,5	0,8	5	2	25	1999
15,5	28,6	0,8	5	2	25	1999
14,2	22	0,8	3	2	27	1999
11,2	10,8	0,8	2	2	25	2007
20,2	63,4	0,8	10	2	18	2012
14,3	22,5	0,8	4	2	26	1999
14,3	22,5	0,8	4	2	23	2007
14,4	23	0,8	4	2	19	2012
14,1	21,6	0,8	5	2	25	1999
15,3	27,6	0,8	4	2	22	2013
16,4	34	0,8	6	2	22	2013
14,5	23,5	0,8	5	2	26	1999
20,1	62,6	0,8	7	2	15	2015
14,7	24,5	0,8	5	2	19	2012

12,7	15,8	0,8	2	2	24	2002
17,9	44,3	0,8	6	2	21	2013
17,4	40,7	0,8	7	2	18	2002
17,4	40,7	0,8	10	2	26	2006
15,1	26,6	0,8	5	2	20	2007
14	21,2	0,8	3	2	22	2011
16,9	37,3	0,8	7	2	19	2012
12,9	16,6	0,8	2	2	25	2002
10,4	8,7	0,8	2	2	23	2004
13,3	18,2	0,8	2	2	25	2002
18,1	45,9	0,8	5	2	17	2012
14,6	24,1	0,8	3	2	25	2002
14,7	24,6	0,8	3	2	23	2003
15,2	27,2	0,8	4	2	26	2005
15,2	27,2	0,8	4	2	19	2012
12	13,4	0,8	2	2	25	2007
11,5	11,8	0,8	2	2	25	2002
15,5	28,9	0,8	6	2	25	1999
15,5	28,9	0,8	4	2	22	2002

11,3	11,2	0,8	2	2	21	2006
13,8	20,4	0,8	3	2	25	2007
14	21,3	0,8	3	2	18	2012
12,4	14,8	0,8	2	2	23	1999
12,4	14,8	0,8	3	2	25	2005
13,5	19,1	0,8	3	2	23	2002
16,1	32,4	0,8	5	2	21	2012
15,2	27,3	0,8	6	2	22	2000
14,5	23,7	0,8	4	2	22	2015
13,7	20	0,8	4	2	27	1999
13,7	20	0,8	3	2	26	2009
12,9	16,7	0,8	3	2	25	2007
13,2	17,9	0,8	2	2	25	2002
14,2	22,3	0,8	3	2	15	2015
12,3	14,5	0,8	3	2	22	2005
14,3	22,8	0,8	7	2	26	1999
19	53,5	0,8	6	2	18	2012
15,2	27,4	0,8	4	2	20	2011
21,4	76,5	0,8	10	2	16	2015

17,7	43,3	0,8	9	2	19	2000
15,5	29,1	0,8	5	2	21	2000
15,3	28	0,8	3	2	26	2000
15,3	28	0,8	6	2	19	2011
14,3	22,9	0,8	3	2	22	2002
18,3	48	0,8	6	2	19	2013
15,9	31,5	0,8	4	2	22	2002
13,8	20,6	0,8	6	2	25	2008
13,5	19,3	0,8	5	2	26	1999
16,8	37,2	0,8	5	2	21	2012
15,3	28,1	0,8	4	2	22	2011
21,5	78	0,8	8	2	16	2015
19,5	58,2	0,8	10	2	17	2012
15,7	30,4	0,8	8	2	23	1999
13,9	21,1	0,8	2	2	25	2002
15,4	28,7	0,8	7	2	18	2012
16,4	34,7	0,8	4	2	26	2000
15,1	27,1	0,8	4	2	22	1999
15,3	28,2	0,8	4	2	22	2011

13	17,3	0,8	4	2	26	1999
13	17,3	0,8	2	2	22	2002
12,2	14,3	0,8	3	2	26	1999
15	26,6	0,8	2	2	22	2013
13,5	19,4	0,8	2	2	22	2002
16	32,3	0,8	7	2	19	2013
13,4	19	0,8	2	2	25	2002
12,3	14,7	0,8	2	2	25	2002
10	7,9	0,8	2	2	19	2007
16,1	33	0,8	6	2	25	2005
15	26,7	0,8	4	2	26	2003
15,4	28,9	0,8	5	2	21	2012
13	17,4	0,8	2	2	22	2002
15,7	30,7	0,8	5	2	25	2005
12,6	15,9	0,8	2	2	22	2002
15,5	29,6	0,8	7	2	17	2012
12,3	14,8	0,8	2	2	25	2008
14,8	25,8	0,8	5	2	22	2002
16	32,6	0,8	6	2	20	2011

12,1	14,1	0,8	2	2	25	2002
13,1	17,9	0,8	3	2	22	2002
12,8	16,7	0,8	4	2	27	1999
13	17,5	0,8	2	2	22	2002
14,3	23,3	0,8	5	2	20	2007
17,9	45,7	0,8	10	2	18	2012
13,9	21,4	0,8	3	2	26	2000
15,5	29,7	0,8	8	2	22	2002
16,2	34	0,8	5	2	21	2012
14,2	22,9	0,8	3	2	25	2002
9,7	7,3	0,8	2	2	23	2004
14,5	24,4	0,8	3	2	25	2002
15,7	31	0,8	7	2	23	1999
14,8	26	0,8	5	2	22	1999
17,9	46	0,8	7	2	18	2012
13,6	20,2	0,8	6	2	25	1999
13,6	20,2	0,8	5	2	27	1999
14,4	24	0,8	2	2	22	2003
12,2	14,6	0,8	3	2	21	2014

11,7	12,9	0,8	3	2	25	2005
13	17,7	0,8	2	2	25	2002
12,3	15	0,8	3	2	17	2012
16,2	34,3	0,8	6	2	22	2011
15,3	28,9	0,8	4	2	19	2000
14,5	24,6	0,8	4	2	25	2002
13,6	20,3	0,8	2	2	25	2002
17,3	41,8	0,8	6	2	24	2006
17,1	40,4	0,8	8	2	18	2012
13,7	20,8	0,8	5	2	27	1999
12,5	15,8	0,8	3	2	26	2005
14	22,2	0,8	3	2	25	2002
12	14	0,8	3	2	22	2000
16,5	36,4	0,8	7	2	26	1999
13,8	21,3	0,8	3	2	25	2002
12,3	15,1	0,8	2	2	26	2006
15,2	28,5	0,8	4	2	27	1999
16,4	35,8	0,8	7	2	18	2012
15	27,4	0,8	5	2	17	2014

14,7	25,8	0,8	4	2	22	2011
11,9	13,7	0,8	2	2	26	2005
13,2	18,7	0,8	3	2	26	1999
13,2	18,7	0,8	2	2	25	2002
13,2	18,7	0,8	4	2	26	2006
16,6	37,2	0,8	6	2	21	2013
14,2	23,3	0,8	2	2	21	2000
14,3	23,8	0,8	3	2	25	2007
16,3	35,3	0,8	6	2	20	2011
12,7	16,7	0,8	2	2	22	2002
17,1	40,8	0,8	6	2	22	2013
11,2	11,5	0,8	2	2	22	2007
13,4	19,7	0,8	4	2	25	2005
13,4	19,7	0,8	4	2	25	2005
15,1	28,2	0,8	6	2	21	2013
21,9	86,1	0,8	10	2	18	2012
15,9	33	0,8	10	2	26	2006
13,5	20,2	0,8	5	2	25	1999
13,8	21,6	0,8	4	2	26	2005

17,4	43,3	0,8	6	2	20	2013
14,4	24,6	0,8	4	2	20	2011
22,9	99	0,8	7	2	18	2012
15,6	31,3	0,8	8	2	21	2010
15,6	31,3	0,8	8	2	21	2010
17,4	43,5	0,8	10	2	18	2012
12,3	15,4	0,8	2	2	22	2002
14,4	24,9	0,8	4	2	27	1999
14,4	24,9	0,8	10	2	26	2006
12,5	16,3	0,8	2	2	17	2012
18,3	51,2	0,8	7	2	18	2012
13	18,4	0,8	2	2	26	2006
15,1	28,9	0,8	4	2	20	2012
12,6	16,8	0,8	3	2	26	2005
16,4	37,2	0,8	7	2	25	1999
12,2	15,4	0,8	2	2	25	2002
13,4	20,5	0,9	4	2	26	2006
14,5	26,1	0,9	7	2	19	2012
14,3	25,1	0,9	5	2	26	2005

13	18,9	0,9	2	2	23	1999
13,4	20,8	0,9	2	2	25	2002
14,2	25,1	0,9	9	2	26	2006
13,4	21,1	0,9	4	2	25	1999
16,2	37,5	0,9	5	2	19	2012
17,1	44,3	0,9	8	2	21	2013
12,5	17,4	0,9	3	2	20	2003
11,9	15,1	0,9	2	2	17	2012
20,6	78,8	0,9	10	2	19	2012
11,9	15,2	0,9	3	2	26	2005
15,9	36,5	0,9	7	2	26	2006
15,1	31,4	0,9	5	2	23	2006
13,1	20,7	0,9	2	2	25	2002
13,7	23,8	0,9	9	2	20	2013
15,6	35,2	0,9	5	2	21	2012
14,7	29,5	0,9	7	2	26	1999
20,2	76,6	0,9	10	2	18	2012
13,4	22,4	0,9	3	2	20	2003
17	46,8	1,0	7	2	18	2012

13,7	24,7	1,0	5	2	25	1999
16,9	49,2	1,0	7	2	18	2012
13	22,4	1,0	4	2	23	2008
16,3	48,2	1,1	9	2	18	2000
14,2	36,5	1,3	7	2	17	2014
16,7	62,5	1,3	6	2	19	2002
14,7	11,8	0,4	2	1	20	2002
14,8	12,7	0,4	4	1	22	2004
18,7	26,5	0,4	9	1	21	2014
14,7	12,9	0,4	6	1	21	2013
12,5	8	0,4	1	1	20	2013
18,3	25,4	0,4	5	1	15	2015
16,7	19,9	0,4	6	1	20	2013
12,6	8,6	0,4	1	1	20	2013
14,9	14,4	0,4	4	1	19	2004
16,2	18,7	0,4	4	1	19	2002
14,3	12,9	0,4	3	1	20	2013
14,5	13,5	0,4	2	1	20	2013
15,1	15,3	0,4	4	1	19	2004

15	15,1	0,4	6	1	20	2013
21	41,7	0,5	8	1	19	2010
21	41,7	0,5	8	1	19	2010
15,7	17,5	0,5	4	1	19	2015
14,2	13	0,5	4	1	21	2009
15,4	16,6	0,5	3	1	19	2005
14,4	13,6	0,5	5	1	26	2006
14,8	14,8	0,5	4	1	26	2006
12,2	8,3	0,5	1	1	20	2013
17,3	23,7	0,5	8	1	15	2015
14,3	13,4	0,5	3	1	23	2009
14,7	14,7	0,5	2	1	20	2013
16	19	0,5	4	1	19	2015
14,3	13,6	0,5	3	1	23	2010
14,3	13,6	0,5	3	1	23	2010
14,5	14,2	0,5	5	1	20	2013
16	19,2	0,5	4	1	26	2006
17,1	23,5	0,5	5	1	17	2002
14	12,9	0,5	4	1	23	2009

15,7	18,2	0,5	4	1	20	2001
17,6	25,7	0,5	5	1	21	2010
17,6	25,7	0,5	5	1	21	2010
15,5	17,6	0,5	4	1	23	2001
13,9	12,7	0,5	2	1	20	2010
13,9	12,7	0,5	2	1	20	2010
13,6	11,9	0,5	2	1	20	2013
15,4	17,3	0,5	6	1	21	2011
13,4	11,4	0,5	3	1	23	2010
13,4	11,4	0,5	3	1	23	2010
14,9	15,7	0,5	3	1	20	2012
12,9	10,2	0,5	3	1	18	2008
15,9	19,1	0,5	4	1	23	2001
14,7	15,1	0,5	2	1	21	2010
14,7	15,1	0,5	2	1	21	2010
14,7	15,1	0,5	6	1	20	2013
13,5	11,7	0,5	3	1	20	2009
13,9	12,8	0,5	2	1	18	2000
13,9	12,8	0,5	4	1	23	2006

14	13,1	0,5	4	1	15	2015
17,8	27	0,5	5	1	18	2015
15,4	17,5	0,5	4	1	19	2009
13,8	12,6	0,5	2	1	20	2000
17	23,6	0,5	5	1	23	2010
17	23,6	0,5	5	1	23	2010
14	13,2	0,5	4	1	26	2006
14	13,2	0,5	4	1	21	2011
14	13,2	0,5	2	1	19	2014
14,8	15,6	0,5	4	1	22	2011
13,4	11,6	0,5	2	1	20	2004
15,8	19,1	0,5	4	1	20	2001
15,1	16,7	0,5	5	1	21	2009
12,5	9,5	0,5	1	1	20	2013
15,2	17,1	0,5	4	1	23	2005
12,4	9,3	0,5	2	1	20	2013
15,1	16,8	0,5	5	1	19	2010
15,1	16,8	0,5	5	1	19	2010
14	13,4	0,5	5	1	23	2010

14	13,4	0,5	5	1	23	2010
14,6	15,2	0,5	4	1	26	2006
15,3	17,5	0,5	3	1	26	2006
16,1	20,4	0,5	4	1	21	2001
16,3	21,2	0,5	3	1	18	2002
12,6	9,8	0,5	2	1	20	2013
17,1	24,5	0,5	6	1	22	2011
15,7	19	0,5	5	1	20	2013
16,1	20,5	0,5	5	1	21	2010
16,1	20,5	0,5	5	1	21	2010
17,2	25	0,5	6	1	18	2015
13,2	11,3	0,5	2	1	26	2006
15,5	18,3	0,5	4	1	21	2010
15,5	18,3	0,5	4	1	21	2010
12,5	9,6	0,5	2	1	20	2004
13,5	12,1	0,5	3	1	22	2005
15	16,6	0,5	4	1	20	2013
14	13,5	0,5	7	1	21	2009
14,2	14,1	0,5	2	1	20	2013

14,3	14,4	0,5	4	1	21	2009
17,5	26,4	0,5	8	1	15	2015
12,7	10,1	0,5	2	1	25	2007
15,7	19,1	0,5	6	1	19	2000
14,8	16	0,5	3	1	19	2015
16,3	21,4	0,5	4	1	23	2010
16,3	21,4	0,5	4	1	23	2010
16,4	21,8	0,5	4	1	19	2015
13,4	11,9	0,5	3	1	23	2004
13,4	11,9	0,5	3	1	21	2009
15,2	17,4	0,5	4	1	22	2004
16	20,3	0,5	4	1	19	2013
14	13,6	0,5	5	1	23	2007
14,1	13,9	0,5	3	1	20	2002
14,1	13,9	0,5	3	1	19	2015
14,3	14,5	0,5	3	1	19	2015
14,2	14,2	0,5	6	1	26	2006
14,2	14,2	0,5	5	1	22	2007
13	10,9	0,5	2	1	23	2009

13	10,9	0,5	2	1	20	2013
17	24,4	0,5	5	1	26	1999
15,8	19,6	0,5	5	1	19	2014
15,8	19,6	0,5	8	1	18	2015
13,6	12,5	0,5	3	1	23	2010
13,6	12,5	0,5	3	1	23	2010
12	8,6	0,5	2	1	18	2007
12	8,6	0,5	2	1	18	2007
13,7	12,8	0,5	8	1	23	2010
13,7	12,8	0,5	8	1	23	2010
16	20,4	0,5	8	1	15	2015
14,6	15,5	0,5	4	1	20	2011
16,1	20,8	0,5	5	1	21	2010
16,1	20,8	0,5	5	1	21	2010
12,9	10,7	0,5	2	1	20	2001
13,8	13,1	0,5	3	1	23	2010
13,8	13,1	0,5	3	1	23	2010
15,7	19,3	0,5	4	1	21	2009
20,5	43	0,5	6	1	20	2012

14	13,7	0,5	3	1	23	2004
14,1	14	0,5	4	1	25	2004
14,1	14	0,5	5	1	23	2007
14,1	14	0,5	4	1	22	2009
14,2	14,3	0,5	5	1	23	2010
14,2	14,3	0,5	5	1	23	2010
15,3	17,9	0,5	5	1	18	2012
15,3	17,9	0,5	5	1	20	2013
13,2	11,5	0,5	3	1	23	2010
13,2	11,5	0,5	3	1	23	2010
14,7	15,9	0,5	3	1	22	2003
16,4	22,1	0,5	9	1	25	2004
15,2	17,6	0,5	4	1	23	2009
15,7	19,4	0,5	3	1	17	2008
13,7	12,9	0,5	3	1	23	2010
13,7	12,9	0,5	3	1	23	2010
15,5	18,7	0,5	4	1	18	2015
15,3	18	0,5	3	1	20	2013
13,1	11,3	0,5	2	1	17	2008

13,4	12,1	0,5	2	1	26	2006
14,2	14,4	0,5	3	1	23	2010
14,2	14,4	0,5	3	1	23	2010
12,9	10,8	0,5	2	1	21	2006
16,1	21	0,5	4	1	20	2013
16,2	21,4	0,5	6	1	25	2003
12,4	9,6	0,5	2	1	25	2004
20,1	40,9	0,5	4	1	21	2010
20,1	40,9	0,5	4	1	21	2010
15	17	0,5	4	1	22	2004
15	17	0,5	4	1	15	2015
15,4	18,4	0,5	3	1	22	2003
13,5	12,4	0,5	2	1	21	2010
13,5	12,4	0,5	2	1	21	2010
15,2	17,7	0,5	3	1	18	2007
15,8	19,9	0,5	6	1	23	2010
15,8	19,9	0,5	6	1	23	2010
17	24,8	0,5	8	1	18	2008
14,5	15,4	0,5	3	1	22	2011

13	11,1	0,5	2	1	20	2001
16,5	22,7	0,5	7	1	16	2015
17,5	27,1	0,5	5	1	19	2013
13,3	11,9	0,5	2	1	20	2013
16,8	24	0,5	4	1	18	2012
11,5	7,7	0,5	2	1	25	2004
15,4	18,5	0,5	4	1	19	2006
14	13,9	0,5	2	1	17	2014
14,1	14,2	0,5	3	1	19	2009
14,1	14,2	0,5	2	1	20	2012
12,2	9,2	0,5	2	1	21	2011
16,7	23,6	0,5	4	1	20	2004
16,7	23,6	0,5	4	1	21	2010
16,7	23,6	0,5	4	1	21	2010
14,7	16,1	0,5	5	1	23	2010
14,7	16,1	0,5	5	1	23	2010
16,4	22,4	0,5	8	1	20	2010
16,4	22,4	0,5	8	1	20	2010
16,4	22,4	0,5	6	1	23	2011

15,1	17,5	0,5	4	1	22	2011
17	25	0,5	6	1	21	2010
17	25	0,5	6	1	21	2010
14,8	16,5	0,5	2	1	21	2000
15,7	19,7	0,5	4	1	20	2009
12	8,8	0,5	1	1	21	2005
18	29,7	0,5	5	1	20	2010
18	29,7	0,5	5	1	20	2010
13,7	13,1	0,5	2	1	23	2006
14,3	14,9	0,5	2	1	18	2002
14,3	14,9	0,5	4	1	22	2009
15,2	17,9	0,5	5	1	18	2000
13,8	13,4	0,5	3	1	21	2005
14,2	14,6	0,5	4	1	19	2015
14,2	14,6	0,5	3	1	19	2015
13,3	12	0,5	3	1	22	2001
14,1	14,3	0,5	3	1	20	2004
14,1	14,3	0,5	7	1	22	2009
14,1	14,3	0,5	3	1	16	2015

16,1	21,3	0,5	4	1	20	2012
12,3	9,5	0,5	2	1	23	2010
12,3	9,5	0,5	2	1	23	2010
14,9	16,9	0,5	3	1	23	2001
14,6	15,9	0,5	4	1	20	2008
15,3	18,3	0,5	4	1	18	2015
13,4	12,3	0,5	3	1	19	2015
13,1	11,5	0,5	2	1	20	2013
13,1	11,5	0,5	2	1	20	2013
14,5	15,6	0,5	5	1	23	1999
17,3	26,5	0,5	4	1	15	2015
12,5	10	0,5	2	1	19	2007
17,7	28,4	0,5	7	1	18	2015
12,2	9,3	0,5	2	1	18	2007
14,4	15,3	0,5	6	1	19	2008
14,4	15,3	0,5	4	1	20	2009
15,9	20,6	0,5	5	1	19	1999
11,6	8	0,5	2	1	22	2009
15	17,3	0,5	5	1	26	2006

15	17,3	0,5	5	1	23	2010
15	17,3	0,5	5	1	23	2010
16	21	0,5	4	1	20	2012
16	21	0,5	4	1	16	2015
17	25,2	0,5	4	1	17	2014
14,3	15	0,5	3	1	18	2002
14,3	15	0,5	3	1	19	2008
14,2	14,7	0,5	2	1	20	2010
14,2	14,7	0,5	2	1	20	2010
14,2	14,7	0,5	2	1	20	2013
15,6	19,5	0,5	5	1	20	2012
15,6	19,5	0,5	5	1	20	2012
15,3	18,4	0,5	5	1	21	2009
13,9	13,8	0,5	4	1	22	2013
14	14,1	0,5	3	1	26	2006
16,8	24,4	0,5	6	1	23	2005
15,4	18,8	0,5	6	1	26	2006
12,8	10,8	0,5	2	1	18	2007
12,8	10,8	0,5	3	1	18	2015

16,2	21,9	0,5	7	1	21	2014
16,2	21,9	0,5	7	1	23	2014
16	21,1	0,5	3	1	19	2010
16	21,1	0,5	3	1	19	2010
14,8	16,7	0,5	4	1	23	2011
16,7	24	0,5	7	1	18	2015
15,2	18,1	0,5	5	1	19	1999
15,5	19,2	0,5	6	1	15	2015
18,2	31,1	0,5	10	1	21	2015
17,9	29,6	0,5	7	1	20	2012
17,4	27,2	0,5	4	1	18	2015
15,7	20	0,5	4	1	22	2011
15,7	20	0,5	3	1	20	2013
14,2	14,8	0,5	2	1	20	2013
15,8	20,4	0,5	4	1	19	2004
13,2	11,9	0,5	2	1	25	2003
16,7	24,1	0,5	6	1	22	2011
12,7	10,6	0,5	2	1	20	2013
13,8	13,6	0,5	3	1	20	2013

14	14,2	0,5	3	1	17	2014
14	14,2	0,5	4	1	21	2015
16	21,2	0,5	6	1	20	2013
16,1	21,6	0,5	4	1	20	2013
16,1	21,6	0,5	3	1	15	2015
17,1	25,9	0,5	4	1	17	2014
17,1	25,9	0,5	4	1	18	2015
14,8	16,8	0,5	3	1	16	2008
14,5	15,8	0,5	4	1	21	2011
15,5	19,3	0,5	5	1	23	2010
15,5	19,3	0,5	5	1	23	2010
15	17,5	0,5	3	1	26	2006
15,6	19,7	0,5	5	1	21	2010
15,6	19,7	0,5	5	1	21	2010
19,9	40,9	0,5	6	1	20	2013
14,4	15,5	0,5	4	1	21	2009
12,4	9,9	0,5	2	1	23	2010
12,4	9,9	0,5	2	1	23	2010
15,3	18,6	0,5	5	1	25	2004

15,3	18,6	0,5	10	1	26	2006
15,3	18,6	0,5	5	1	18	2007
15,3	18,6	0,5	9	1	19	2011
13,4	12,5	0,5	2	1	21	2009
13,4	12,5	0,5	2	1	22	2011
16,3	22,5	0,5	5	1	18	2002
12,8	10,9	0,5	2	1	18	2007
14,3	15,2	0,5	5	1	22	2011
15,1	17,9	0,5	5	1	23	2010
15,1	17,9	0,5	5	1	23	2010
15,1	17,9	0,5	5	1	20	2012
14,9	17,2	0,5	4	1	23	2001
14,2	14,9	0,5	3	1	21	2005
14,2	14,9	0,5	3	1	19	2008
14,2	14,9	0,5	4	1	20	2009
14,2	14,9	0,5	3	1	20	2009
13,1	11,7	0,5	2	1	20	2013
14,6	16,2	0,5	6	1	26	2006
14,6	16,2	0,5	6	1	23	2010

14,6	16,2	0,5	6	1	23	2010
14,6	16,2	0,5	3	1	16	2015
16,5	23,4	0,5	6	1	19	2011
17,6	28,4	0,5	10	1	19	2012
13,7	13,4	0,5	5	1	20	2011
14	14,3	0,5	2	1	23	2001
14	14,3	0,5	2	1	18	2008
13,8	13,7	0,5	2	1	23	2001
13,8	13,7	0,5	4	1	19	2015
13,9	14	0,5	2	1	26	2006
14,8	16,9	0,5	3	1	23	2001
15	17,6	0,5	5	1	19	2007
14,5	15,9	0,5	2	1	23	2001
14,5	15,9	0,5	3	1	21	2010
14,5	15,9	0,5	3	1	21	2010
13,2	12	0,5	3	1	18	2007
15,7	20,2	0,5	8	1	22	2004
15,3	18,7	0,5	3	1	20	2010
15,3	18,7	0,5	3	1	20	2010

16,2	22,2	0,5	4	1	20	2013
12,7	10,7	0,5	2	1	26	2006
14,7	16,6	0,5	3	1	20	2010
14,7	16,6	0,5	3	1	20	2010
15,4	19,1	0,5	4	1	19	2004
14,9	17,3	0,5	4	1	20	2004
14,3	15,3	0,5	4	1	25	2004
14,3	15,3	0,5	3	1	21	2010
14,3	15,3	0,5	3	1	21	2010
14,3	15,3	0,5	5	1	20	2013
14,6	16,3	0,5	3	1	19	2009
14,2	15	0,5	3	1	19	2004
14,2	15	0,5	2	1	19	2012
16,7	24,4	0,5	4	1	22	2009
16,7	24,4	0,5	3	1	22	2015
14,1	14,7	0,5	3	1	20	2010
14,1	14,7	0,5	3	1	20	2010
15	17,7	0,5	4	1	19	2007
16,2	22,3	0,5	5	1	21	2000

13,6	13,2	0,5	2	1	20	2012
13,6	13,2	0,5	4	1	21	2015
14	14,4	0,5	4	1	20	2004
15,8	20,7	0,5	4	1	17	2014
14,5	16	0,5	3	1	18	2000
14,5	16	0,5	2	1	23	2001
14,5	16	0,5	4	1	21	2009
13,1	11,8	0,5	2	1	23	2001
13,1	11,8	0,5	2	1	21	2009
15,3	18,8	0,5	3	1	20	1999
15,3	18,8	0,5	3	1	18	2015
15,9	21,1	0,5	4	1	18	2015
13,7	13,5	0,5	5	1	21	2000
13,7	13,5	0,5	2	1	21	2009
13,7	13,5	0,5	2	1	21	2009
13,7	13,5	0,5	5	1	21	2011
13,8	13,8	0,5	3	1	23	2010
13,8	13,8	0,5	3	1	23	2010
13,8	13,8	0,5	2	1	19	2013

14,7	16,7	0,5	5	1	22	2009
14,7	16,7	0,5	3	1	22	2011
14,9	17,4	0,5	4	1	19	2004
11,5	8	0,5	1	1	21	2009
16,7	24,5	0,5	5	1	19	2009
15,5	19,6	0,5	4	1	17	2002
16,3	22,8	0,5	3	1	18	2015
14,3	15,4	0,5	3	1	23	2008
15,2	18,5	0,5	4	1	22	2005
15,2	18,5	0,5	3	1	22	2011
15,6	20	0,5	4	1	22	2011
16,6	24,1	0,5	4	1	20	2010
16,6	24,1	0,5	4	1	20	2010
16,2	22,4	0,5	4	1	18	2008
16,2	22,4	0,5	6	1	20	2013
16,2	22,4	0,5	3	1	17	2014
13,3	12,4	0,5	2	1	20	2009
15,7	20,4	0,5	4	1	19	2013
15,7	20,4	0,5	3	1	18	2015

15,8	20,8	0,5	3	1	20	2010
15,8	20,8	0,5	3	1	20	2010
16	21,6	0,5	4	1	19	2004
14,2	15,1	0,5	3	1	16	2015
15	17,8	0,5	3	1	20	2010
15	17,8	0,5	3	1	20	2010
15	17,8	0,5	4	1	18	2015
15	17,8	0,5	4	1	18	2015
14,8	17,1	0,5	3	1	23	2001
13,4	12,7	0,5	2	1	19	2007
13,4	12,7	0,5	4	1	21	2009
13,4	12,7	0,5	4	1	21	2009
14,1	14,8	0,5	3	1	15	2015
13	11,6	0,5	2	1	23	2009
13	11,6	0,5	2	1	20	2013
13,5	13	0,5	2	1	19	2012
14	14,5	0,5	2	1	18	2008
14	14,5	0,5	2	1	20	2013
15,1	18,2	0,5	5	1	18	2007

15,1	18,2	0,5	4	1	20	2012
13,9	14,2	0,5	2	1	26	2006
14,7	16,8	0,5	4	1	21	2009
15,5	19,7	0,5	5	1	26	2006
15,5	19,7	0,5	4	1	20	2010
15,5	19,7	0,5	4	1	20	2010
15,5	19,7	0,5	4	1	20	2013
14,4	15,8	0,5	3	1	26	2006
17	26	0,5	4	1	20	2013
16,2	22,5	0,5	4	1	21	1999
16,2	22,5	0,5	4	1	20	2013
13,1	11,9	0,5	3	1	20	2004
13,1	11,9	0,5	2	1	20	2013
12,4	10,1	0,5	3	1	19	2015
16	21,7	0,5	4	1	19	2012
15,9	21,3	0,5	4	1	19	2004
17,9	30,4	0,5	6	1	23	2001
16,7	24,7	0,5	8	1	22	2015
15	17,9	0,5	4	1	19	2009

15	17,9	0,5	4	1	23	2011
15	17,9	0,5	2	1	19	2013
15,3	19	0,5	4	1	18	2000
14,8	17,2	0,5	4	1	20	2009
14,8	17,2	0,5	3	1	20	2013
12,1	9,4	0,5	2	1	18	2007
17,3	27,5	0,5	4	1	16	2015
15,4	19,4	0,5	3	1	21	2001
15,4	19,4	0,5	4	1	22	2001
15,4	19,4	0,5	3	1	22	2003
13,3	12,5	0,5	3	1	22	2005
14,5	16,2	0,5	4	1	19	1999
14,5	16,2	0,5	4	1	21	2011
14,5	16,2	0,5	4	1	23	2011
15,1	18,3	0,5	3	1	26	2003
15,1	18,3	0,5	3	1	21	2008
15,5	19,8	0,5	4	1	19	1999
15,5	19,8	0,5	5	1	20	2010
15,5	19,8	0,5	5	1	20	2010

13,4	12,8	0,5	2	1	22	2009
13,4	12,8	0,5	2	1	20	2013
12,3	9,9	0,5	2	1	26	2006
14,9	17,6	0,5	3	1	20	2007
16	21,8	0,5	6	1	20	2013
15,8	21	0,5	5	1	23	1999
15,8	21	0,5	4	1	18	2015
13,5	13,1	0,5	2	1	26	2006
16,9	25,7	0,5	5	1	20	2012
14,4	15,9	0,5	3	1	19	2015
15,2	18,7	0,5	5	1	23	2011
13	11,7	0,5	2	1	26	2006
13	11,7	0,5	2	1	26	2006
13	11,7	0,5	2	1	20	2013
13,8	14	0,5	2	1	19	2001
13,8	14	0,5	2	1	20	2006
13,8	14	0,5	3	1	18	2015
16,4	23,5	0,5	4	1	19	2001
13,7	13,7	0,5	3	1	21	2009

17,3	27,6	0,5	7	1	23	2001
14,6	16,6	0,5	3	1	18	2002
14,6	16,6	0,5	3	1	20	2008
14,3	15,6	0,5	4	1	20	2004
16,2	22,7	0,5	5	1	22	2009
12,8	11,2	0,5	2	1	22	2009
11,9	9	0,5	2	1	18	2007
14,2	15,3	0,5	4	1	21	2005
14,2	15,3	0,5	2	1	23	2006
14,2	15,3	0,5	4	1	21	2009
14,2	15,3	0,5	3	1	19	2015
17,2	27,2	0,5	8	1	25	2004
14,5	16,3	0,5	5	1	23	2010
14,5	16,3	0,5	5	1	23	2010
14,5	16,3	0,5	4	1	15	2015
16	21,9	0,5	6	1	20	2013
15,6	20,3	0,5	4	1	23	2009
15,7	20,7	0,5	5	1	19	2010
15,7	20,7	0,5	3	1	23	2010

15,7	20,7	0,5	5	1	19	2010
15,7	20,7	0,5	3	1	23	2010
12,6	10,7	0,5	3	1	22	2005
12,6	10,7	0,5	2	1	20	2013
15,8	21,1	0,5	4	1	26	2003
15,8	21,1	0,5	5	1	19	2004
12,4	10,2	0,5	2	1	26	2006
14,1	15	0,5	4	1	24	2006
14,1	15	0,5	3	1	22	2011
15,2	18,8	0,5	3	1	18	2015
17,8	30,2	0,5	10	1	21	2010
17,8	30,2	0,5	10	1	21	2010
13,3	12,6	0,5	2	1	20	2013
13,3	12,6	0,5	3	1	18	2015
16,3	23,2	0,5	7	1	20	2013
14	14,7	0,5	2	1	19	2000
14	14,7	0,5	2	1	15	2015
14	14,7	0,5	3	1	19	2015
14,4	16	0,5	2	1	19	2001

15,3	19,2	0,5	3	1	23	2015
13,4	12,9	0,5	3	1	19	2008
13,4	12,9	0,5	3	1	22	2011
16,2	22,8	0,5	4	1	20	2012
15	18,1	0,5	5	1	20	2010
15	18,1	0,5		1	20	2010
15	18,1	0,5	5	1	20	2010
15	18,1	0,5		1	20	2010
13,5	13,2	0,5	4	1	19	2009
13,5	13,2	0,5	5	1	21	2009
13,5	13,2	0,5	2	1	20	2013
13,8	14,1	0,5	4	1	26	2006
15,4	19,6	0,5	3	1	20	2010
15,4	19,6	0,5	3	1	20	2010
13,6	13,5	0,5	2	1	23	2006
13,6	13,5	0,5	4	1	26	2006
13,7	13,8	0,5	3	1	21	2001
13,7	13,8	0,5	4	1	22	2009
13,7	13,8	0,5	3	1	19	2015

14,8	17,4	0,5	2	1	19	2001
16,1	22,4	0,5	8	1	18	2015
14,3	15,7	0,5	4	1	22	2011
12,7	11	0,5	3	1	23	2010
12,7	11	0,5	3	1	23	2010
15,5	20	0,5	4	1	22	2005
15,5	20	0,5	5	1	20	2010
15,5	20	0,5	5	1	20	2010
13	11,8	0,5	2	1	23	2011
16	22	0,5	6	1	22	2001
16	22	0,5	3	1	18	2015
17,6	29,3	0,5	4	1	20	2010
17,6	29,3	0,5	4	1	20	2010
15,7	20,8	0,5	3	1	21	2001
15,8	21,2	0,5	3	1	19	2014
14,2	15,4	0,5	3	1	26	2005
14,9	17,8	0,5	3	1	23	2001
14,9	17,8	0,5	4	1	18	2014
14,9	17,8	0,5	3	1	18	2015

15,2	18,9	0,5	5	1	21	2013
15,2	18,9	0,5	5	1	17	2014
13,1	12,1	0,5	2	1	18	2008
13,1	12,1	0,5	2	1	18	2014
16,2	22,9	0,5	3	1	20	2001
16,2	22,9	0,5	3	1	18	2015
16,9	26	0,5	8	1	20	2013
17,4	28,4	0,5	6	1	19	2013
13,2	12,4	0,5	2	1	19	2011
13,2	12,4	0,5	2	1	17	2014
17,5	28,9	0,5	5	1	18	2012
15	18,2	0,5	3	1	20	2000
15	18,2	0,5	5	1	20	2012
14	14,8	0,5	2	1	19	2013
15,4	19,7	0,5	4	1	21	2014
16	22,1	0,5	9	1	17	2009
15,5	20,1	0,5	6	1	19	2004
15,5	20,1	0,5	4	1	22	2015
13,3	12,7	0,5	4	1	25	2004

14,6	16,8	0,5	4	1	23	2010
14,6	16,8	0,5	4	1	23	2010
14,8	17,5	0,5	5	1	18	2007
14,8	17,5	0,5	5	1	20	2012
13,9	14,5	0,5	2	1	19	2002
16,6	24,7	0,5	10	1	18	2015
15,6	20,5	0,5	6	1	19	2007
15,8	21,3	0,5	5	1	21	2010
15,8	21,3	0,5	5	1	21	2010
15,7	20,9	0,5	4	1	19	2014
15,1	18,6	0,5	4	1	26	2006
14,3	15,8	0,5	5	1	26	2006
16,3	23,4	0,5	4	1	20	2013
13,5	13,3	0,5	2	1	23	2009
13,5	13,3	0,5	2	1	20	2013
16,2	23	0,5	6	1	23	2010
16,2	23	0,5	6	1	23	2010
14,9	17,9	0,5	7	1	23	2001
14,9	17,9	0,5	6	1	26	2003

14,5	16,5	0,5	3	1	19	2015
14,2	15,5	0,5	3	1	18	2015
14,2	15,5	0,5	6	1	19	2015
14,7	17,2	0,5	4	1	22	2005
14,7	17,2	0,5	4	1	19	2015
13	11,9	0,5	3	1	19	2012
13	11,9	0,5	2	1	19	2014
15,3	19,4	0,5	4	1	25	2004
16,4	23,9	0,5	5	1	19	1999
12,7	11,1	0,5	2	1	26	2009
12,7	11,1	0,5	2	1	23	2010
12,7	11,1	0,5	2	1	23	2010
12,7	11,1	0,5	2	1	19	2013
12,7	11,1	0,5	2	1	20	2013
16	22,2	0,5	5	1	19	1999
16,8	25,7	0,5	6	1	19	2004
16,8	25,7	0,5	4	1	21	2010
16,8	25,7	0,5	4	1	21	2010
16,8	25,7	0,5	4	1	19	2015

15,4	19,8	0,5	4	1	15	2015
14,1	15,2	0,5	5	1	21	2005
14,1	15,2	0,5	4	1	23	2009
14,1	15,2	0,5	5	1	23	2010
14,1	15,2	0,5	5	1	23	2010
14,4	16,2	0,5	5	1	20	2004
14,4	16,2	0,5	4	1	20	2009
15,8	21,4	0,5	3	1	23	2001
15,6	20,6	0,5	3	1	19	2010
15,6	20,6	0,5	3	1	19	2010
15,6	20,6	0,5	5	1	19	2014
13,1	12,2	0,5	2	1	23	2001
13,1	12,2	0,5	2	1	19	2009
13,1	12,2	0,5	3	1	17	2014
13,1	12,2	0,5	2	1	17	2014
14,8	17,6	0,5	3	1	20	2010
14,8	17,6	0,5	3	1	20	2010
14,8	17,6	0,5	4	1	21	2015
17,6	29,6	0,5	6	1	21	2015

14	14,9	0,5	4	1	25	2004
14	14,9	0,5	4	1	26	2006
14	14,9	0,5	3	1	20	2010
14	14,9	0,5	3	1	23	2010
14	14,9	0,5	3	1	20	2010
14	14,9	0,5	3	1	23	2010
15,1	18,7	0,5	3	1	16	2008
15,1	18,7	0,5	7	1	21	2009
16,5	24,4	0,5	5	1	18	2002
16,2	23,1	0,5	6	1	21	2005
16,2	23,1	0,5	3	1	19	2013
12,8	11,4	0,5	2	1	20	2007
14,3	15,9	0,5	4	1	19	1999
14,3	15,9	0,5	5	1	19	2010
14,3	15,9	0,5	5	1	19	2010
15,2	19,1	0,5	3	1	20	2001
15,2	19,1	0,5	4	1	19	2004
13,8	14,3	0,5	2	1	19	2014
16,6	24,9	0,5	5	1	21	2015

16	22,3	0,5	4	1	19	2013
13,4	13,1	0,5	2	1	21	2009
15,3	19,5	0,5	3	1	22	2007
15,3	19,5	0,5	3	1	18	2015
13,7	14	0,5	4	1	26	2006
13,7	14	0,5	3	1	21	2010
13,7	14	0,5	3	1	21	2010
13,7	14	0,5	2	1	20	2012
14,5	16,6	0,5	3	1	19	2010
14,5	16,6	0,5	3	1	19	2010
14,5	16,6	0,5	4	1	22	2011
14,7	17,3	0,5	4	1	23	2004
13,5	13,4	0,5	4	1	19	2015
13,6	13,7	0,5	2	1	20	2013
12,9	11,7	0,5	2	1	19	2009
15,8	21,5	0,5	4	1	19	2011
15	18,4	0,5	9	1	20	2011
12,2	9,9	0,5	2	1	26	2006
15,6	20,7	0,5	6	1	19	2007

15,6	20,7	0,5	5	1	20	2012
16,5	24,5	0,5	6	1	20	2011
18,4	34	0,5	4	1	18	2014
14,4	16,3	0,5	3	1	18	2015
14,8	17,7	0,5	4	1	23	2006
14,8	17,7	0,5	3	1	22	2011
15,1	18,8	0,5	3	1	20	2003
15,1	18,8	0,5	5	1	23	2005
13	12	0,5	5	1	26	2006
13	12	0,5	7	1	23	2009
14,6	17	0,5	2	1	19	2002
14,6	17	0,5	3	1	25	2004
17,2	27,8	0,5	4	1	21	2010
17,2	27,8	0,5	4	1	21	2010
21,3	52,8	0,5	5	1	18	2012
14	15	0,5	5	1	21	2000
14	15	0,5	2	1	19	2001
14	15	0,5	2	1	24	2006
14	15	0,5	3	1	18	2008

15,2	19,2	0,5	3	1	20	2010
15,2	19,2	0,5	3	1	20	2010
16	22,4	0,5	7	1	22	2009
14,9	18,1	0,5	3	1	20	2001
14,9	18,1	0,5	3	1	21	2010
14,9	18,1	0,5	3	1	21	2010
15,3	19,6	0,5	5	1	20	2006
15,9	22	0,5	4	1	20	2006
15,9	22	0,5	3	1	15	2015
13,9	14,7	0,5	3	1	20	2008
15,4	20	0,5	4	1	21	1999
15,4	20	0,5	3	1	18	2002
15,4	20	0,5	4	1	20	2013
15,4	20	0,5	4	1	16	2015
15,8	21,6	0,5	5	1	19	2012
14,7	17,4	0,5	4	1	22	2011
13,2	12,6	0,5	2	1	20	2001
13,2	12,6	0,5	3	1	23	2007
13,2	12,6	0,5	3	1	22	2009

13,2	12,6	0,5	2	1	20	2013
13,2	12,6	0,5	2	1	18	2014
16,2	23,3	0,5	7	1	17	2014
15	18,5	0,5	5	1	23	1999
14,2	15,7	0,5	5	1	19	2004
13,3	12,9	0,5	5	1	20	2007
13,3	12,9	0,5	2	1	26	2009
13,7	14,1	0,5	2	1	19	2008
12,8	11,5	0,5	2	1	17	2014
13,4	13,2	0,5	3	1	21	2009
13,6	13,8	0,5	3	1	22	2009
13,5	13,5	0,5	3	1	21	2011
15,1	18,9	0,5	3	1	18	2002
14,8	17,8	0,5	4	1	25	2004
14,8	17,8	0,5	4	1	21	2010
14,8	17,8	0,5	4	1	21	2010
14,4	16,4	0,5	4	1	23	1999
14,4	16,4	0,5	3	1	19	2004
14,4	16,4	0,5	4	1	23	2006

14,1	15,4	0,5	3	1	26	2006
14,1	15,4	0,5	4	1	26	2009
14,1	15,4	0,5	4	1	19	2015
14,6	17,1	0,5	5	1	20	2012
15,2	19,3	0,5	3	1	20	2001
15,2	19,3	0,5	5	1	24	2006
16,7	25,6	0,5	7	1	21	2009
16,5	24,7	0,5	3	1	20	2001
16,5	24,7	0,5	3	1	15	2015
12,6	11	0,5	2	1	20	2007
15,3	19,7	0,6	3	1	25	2003
15,3	19,7	0,6	5	1	21	2010
15,3	19,7	0,6	5	1	21	2010
15,8	21,7	0,6	3	1	21	2000
15,8	21,7	0,6	9	1	23	2001
14,9	18,2	0,6	3	1	17	2002
14	15,1	0,6	2	1	19	2001
14	15,1	0,6	3	1	18	2008
14	15,1	0,6	2	1	19	2013

14	15,1	0,6	3	1	16	2015
15,4	20,1	0,6	3	1	17	2014
16,2	23,4	0,6	4	1	20	2012
16,8	26,1	0,6	5	1	19	2002
16,8	26,1	0,6	8	1	22	2011
15,5	20,5	0,6	4	1	20	1999
15,5	20,5	0,6	4	1	25	2004
15,5	20,5	0,6	4	1	19	2009
15,6	20,9	0,6	3	1	19	2001
15,6	20,9	0,6	4	1	23	2001
15,6	20,9	0,6	4	1	20	2012
14,3	16,1	0,6	4	1	21	2011
12,4	10,5	0,6	2	1	21	2009
12,4	10,5	0,6	2	1	22	2009
13	12,1	0,6	2	1	26	2006
13	12,1	0,6	3	1	23	2008
13	12,1	0,6	2	1	19	2013
16,4	24,3	0,6	5	1	19	2005
16,4	24,3	0,6	5	1	20	2012

14,7	17,5	0,6	5	1	19	2000
14,7	17,5	0,6	2	1	17	2002
14,7	17,5	0,6	3	1	26	2003
14,5	16,8	0,6	3	1	23	2011
13,9	14,8	0,6	2	1	20	2013
15	18,6	0,6	4	1	19	2002
15	18,6	0,6	4	1	23	2009
15	18,6	0,6	3	1	21	2010
15	18,6	0,6	3	1	21	2010
15	18,6	0,6	4	1	23	2011
16,1	23	0,6	5	1	20	2012
18,1	32,7	0,6	4	1	16	2015
13,1	12,4	0,6	2	1	20	2011
12,7	11,3	0,6	2	1	25	2008
12,7	11,3	0,6	2	1	19	2013
14,2	15,8	0,6	7	1	26	2006
14,2	15,8	0,6	4	1	21	2011
14,2	15,8	0,6	3	1	19	2013
15,1	19	0,6	4	1	21	1999

15,1	19	0,6	4	1	19	2013
11,9	9,3	0,6	2	1	22	2007
17,1	27,6	0,6	5	1	17	2002
14,8	17,9	0,6	3	1	16	2015
13,2	12,7	0,6	3	1	21	2011
13,7	14,2	0,6	4	1	19	2009
13,7	14,2	0,6	6	1	21	2009
13,7	14,2	0,6	2	1	22	2009
13,7	14,2	0,6	3	1	23	2010
13,7	14,2	0,6	3	1	23	2010
13,7	14,2	0,6	7	1	22	2011
13,7	14,2	0,6	3	1	18	2015
15,9	22,2	0,6	3	1	21	2014
15,2	19,4	0,6	3	1	21	2001
15,2	19,4	0,6	5	1	26	2006
15,2	19,4	0,6	6	1	20	2012
13,3	13	0,6	2	1	26	2006
14,4	16,5	0,6	3	1	23	2001
14,4	16,5	0,6	2	1	20	2010

14,4	16,5	0,6	2	1	20	2010
14,6	17,2	0,6	3	1	19	2012
15,8	21,8	0,6	4	1	20	2001
16,2	23,5	0,6	4	1	19	2014
13,5	13,6	0,6	2	1	20	2001
13,5	13,6	0,6	2	1	21	2014
13,5	13,6	0,6	2	1	23	2014
15,3	19,8	0,6	4	1	21	1999
15,3	19,8	0,6	4	1	23	2009
14,1	15,5	0,6	4	1	23	2001
14,1	15,5	0,6	2	1	19	2013
14,1	15,5	0,6	2	1	19	2014
15,4	20,2	0,6	3	1	18	2001
15,6	21	0,6	3	1	20	2013
16,4	24,4	0,6	4	1	15	2015
12,1	9,8	0,6	2	1	18	2014
15,5	20,6	0,6	10	1	19	2013
14,9	18,3	0,6	4	1	19	1999
12,3	10,3	0,6	2	1	21	2005

12,3	10,3	0,6	2	1	18	2007
14	15,2	0,6	2	1	21	2000
14	15,2	0,6	3	1	23	2001
14	15,2	0,6	2	1	21	2005
14	15,2	0,6	4	1	26	2006
14	15,2	0,6	2	1	19	2013
16,7	25,8	0,6	3	1	18	2001
14,3	16,2	0,6	2	1	20	2001
14,3	16,2	0,6	3	1	23	2006
14,3	16,2	0,6	2	1	20	2012
14,7	17,6	0,6	5	1	26	2006
15	18,7	0,6	3	1	20	2001
15	18,7	0,6	4	1	22	2005
15	18,7	0,6	7	1	22	2009
15	18,7	0,6	4	1	19	2015
14,5	16,9	0,6	3	1	19	2014
15,1	19,1	0,6	5	1	19	2009
15,9	22,3	0,6	5	1	19	2014
13,9	14,9	0,6	2	1	20	2001

13,9	14,9	0,6	3	1	19	2014
17,8	31,3	0,6	9	1	19	2011
16,9	26,8	0,6	4	1	17	2014
14,8	18	0,6	3	1	20	2003
14,8	18	0,6	3	1	25	2003
14,8	18	0,6	5	1	23	2007
14,8	18	0,6	3	1	15	2015
15,2	19,5	0,6	3	1	19	2010
15,2	19,5	0,6	3	1	19	2010
15,2	19,5	0,6	4	1	20	2012
16,6	25,4	0,6	6	1	21	2003
13	12,2	0,6	2	1	21	2009
13	12,2	0,6	3	1	21	2011
13	12,2	0,6	2	1	20	2013
14,2	15,9	0,6	4	1	23	2001
14,2	15,9	0,6	3	1	20	2010
14,2	15,9	0,6	3	1	20	2010
13,8	14,6	0,6	2	1	19	1999
15,3	19,9	0,6	4	1	20	2004

15,3	19,9	0,6	5	1	23	2010
15,3	19,9	0,6	5	1	23	2010
11,1	7,6	0,6	2	1	19	2007
15,6	21,1	0,6	3	1	19	2000
15,5	20,7	0,6	9	1	22	2001
15,5	20,7	0,6	3	1	22	2001
15,5	20,7	0,6	5	1	22	2005
15,5	20,7	0,6	3	1	20	2012
15,5	20,7	0,6	3	1	17	2014
14,6	17,3	0,6	3	1	21	2014
16,1	23,2	0,6	3	1	23	2001
10,2	5,9	0,6	1	1	21	2010
10,2	5,9	0,6	1	1	21	2010
17,1	27,8	0,6	7	1	18	2002
13,1	12,5	0,6	3	1	20	2007
13,1	12,5	0,6	2	1	19	2013
13,7	14,3	0,6	3	1	23	2010
13,7	14,3	0,6	3	1	23	2010
13,7	14,3	0,6	3	1	23	2011

12,2	10,1	0,6	2	1	22	2011
12,2	10,1	0,6	2	1	23	2011
14,1	15,6	0,6	3	1	20	2009
16,5	25	0,6	5	1	18	1999
16,5	25	0,6	5	1	21	2000
13,2	12,8	0,6	2	1	22	2009
12,7	11,4	0,6	2	1	22	2001
13,6	14	0,6	2	1	19	2000
16	22,8	0,6	6	1	20	2013
13,3	13,1	0,6	2	1	26	2006
13,5	13,7	0,6	2	1	23	2010
13,5	13,7	0,6	2	1	23	2010
13,4	13,4	0,6	4	1	21	2009
15	18,8	0,6	4	1	19	1999
15	18,8	0,6	2	1	21	2003
15	18,8	0,6	3	1	26	2006
15	18,8	0,6	3	1	20	2008
14,7	17,7	0,6	3	1	22	2011
14,7	17,7	0,6	3	1	19	2015

17,7	30,9	0,6	7	1	23	2001
14,3	16,3	0,6	6	1	19	2007
14,3	16,3	0,6	4	1	20	2009
14	15,3	0,6	3	1	18	2008
14,5	17	0,6	4	1	21	2006
14,5	17	0,6	4	1	19	2009
14,5	17	0,6	7	1	22	2009
14,5	17	0,6	3	1	19	2011
15,1	19,2	0,6	5	1	19	2000
15,1	19,2	0,6	2	1	19	2013
17	27,4	0,6	6	1	19	2013
18,6	35,9	0,6	5	1	21	2010
18,6	35,9	0,6	5	1	21	2010
12,8	11,7	0,6	2	1	20	2007
17,9	32	0,6	4	1	18	2012
15,2	19,6	0,6	4	1	20	1999
15,2	19,6	0,6	4	1	26	2001
15,7	21,6	0,6	4	1	25	2004
15,7	21,6	0,6	3	1	23	2010

15,7	21,6	0,6	3	1	23	2010
16,1	23,3	0,6	4	1	20	2012
16,1	23,3	0,6	4	1	20	2012
14,8	18,1	0,6	2	1	20	2001
14,8	18,1	0,6	4	1	21	2011
15,3	20	0,6	3	1	22	2001
15,3	20	0,6	3	1	23	2002
15,3	20	0,6	3	1	21	2003
15,3	20	0,6	4	1	19	2013
13,9	15	0,6	6	1	18	2008
15,4	20,4	0,6	5	1	23	2004
15,4	20,4	0,6	5	1	19	2009
15,5	20,8	0,6	3	1	18	2000
18,4	34,8	0,6	6	1	15	2015
14,2	16	0,6	3	1	19	2004
19,4	40,8	0,6	8	1	20	2013
12,3	10,4	0,6	3	1	26	2006
12,9	12	0,6	3	1	21	2005
16	22,9	0,6	3	1	24	2001

18,5	35,4	0,6	6	1	20	2013
14,6	17,4	0,6	3	1	16	2015
14,6	17,4	0,6	4	1	19	2015
19,3	40,2	0,6	5	1	18	2014
14,9	18,5	0,6	4	1	22	2009
14,4	16,7	0,6	2	1	21	2003
14,4	16,7	0,6	3	1	20	2013
15,9	22,5	0,6	4	1	20	2013
16,2	23,8	0,6	3	1	20	2001
16,2	23,8	0,6	6	1	20	2010
16,2	23,8	0,6	6	1	20	2010
12,6	11,2	0,6	3	1	26	2006
11,8	9,2	0,6	2	1	22	2007
11,8	9,2	0,6	2	1	22	2007
15	18,9	0,6	4	1	25	2004
15	18,9	0,6	4	1	26	2009
13,7	14,4	0,6	2	1	17	2002
13,7	14,4	0,6	2	1	19	2010
13,7	14,4	0,6	2	1	19	2010

13,7	14,4	0,6	3	1	19	2015
10,4	6,3	0,6	1	1	26	2009
14,1	15,7	0,6	4	1	19	1999
14,1	15,7	0,6	3	1	19	2002
14,1	15,7	0,6	3	1	18	2008
15,8	22,1	0,6	5	1	20	2000
14,7	17,8	0,6	5	1	19	2002
14,7	17,8	0,6	5	1	20	2002
13,1	12,6	0,6	3	1	25	2003
13,1	12,6	0,6	2	1	19	2006
13,1	12,6	0,6	5	1	19	2007
13,1	12,6	0,6	2	1	22	2011
15,7	21,7	0,6	5	1	22	2001
15,7	21,7	0,6	5	1	19	2010
15,7	21,7	0,6	5	1	21	2010
15,7	21,7	0,6	5	1	19	2010
15,7	21,7	0,6	5	1	21	2010
15,7	21,7	0,6	8	1	22	2011
14,3	16,4	0,6	4	1	24	2009

14,3	16,4	0,6	3	1	22	2011
13,2	12,9	0,6	2	1	21	2000
13,2	12,9	0,6	2	1	23	2010
13,2	12,9	0,6	2	1	23	2010
15,2	19,7	0,6	3	1	19	2008
13,3	13,2	0,6	4	1	23	2006
13,3	13,2	0,6	2	1	20	2013
13,4	13,5	0,6	5	1	21	2011
15,3	20,1	0,6	4	1	19	1999
14	15,4	0,6	3	1	26	2003
14	15,4	0,6	3	1	26	2003
14	15,4	0,6	4	1	24	2006
14	15,4	0,6	3	1	18	2008
15,5	20,9	0,6	5	1	20	1999
15,5	20,9	0,6	5	1	21	2010
15,5	20,9	0,6	5	1	21	2010
17,6	30,6	0,6	4	1	19	2013
15,4	20,5	0,6	4	1	24	2009
12	9,7	0,6	4	1	21	2009

14,8	18,2	0,6	3	1	18	2002
16	23	0,6	4	1	22	2004
17,5	30,1	0,6	4	1	19	2015
12,2	10,2	0,6	2	1	20	2004
12,2	10,2	0,6	2	1	18	2007
12,2	10,2	0,6	2	1	22	2011
12,2	10,2	0,6	2	1	20	2012
17	27,6	0,6	3	1	19	2010
17	27,6	0,6	3	1	19	2010
11,7	9	0,6	2	1	20	2004
17,3	29,1	0,6	8	1	20	2013
15,9	22,6	0,6	4	1	19	2014
16,4	24,8	0,6	4	1	20	2001
16,4	24,8	0,6	3	1	15	2015
13,9	15,1	0,6	2	1	21	2000
14,9	18,6	0,6	5	1	17	2009
14,6	17,5	0,6	3	1	22	2003
14,6	17,5	0,6	4	1	23	2006
14,6	17,5	0,6	6	1	19	2007

14,6	17,5	0,6	4	1	20	2009
14,6	17,5	0,6	3	1	19	2011
14,4	16,8	0,6	3	1	23	2005
12,8	11,8	0,6	2	1	21	2006
15,8	22,2	0,6	9	1	21	2010
15,8	22,2	0,6	9	1	21	2010
15,8	22,2	0,6	6	1	20	2013
15	19	0,6	7	1	19	2004
15	19	0,6	5	1	18	2007
15	19	0,6	3	1	20	2012
16,1	23,5	0,6	6	1	25	2003
13,8	14,8	0,6	7	1	21	2010
13,8	14,8	0,6	7	1	21	2010
13,8	14,8	0,6	2	1	17	2014
16,5	25,3	0,6	6	1	23	2001
15,7	21,8	0,6	4	1	18	2000
19,3	40,5	0,6	8	1	18	2012
15,1	19,4	0,6	4	1	19	1999
15,1	19,4	0,6	3	1	23	2001

15,1	19,4	0,6	3	1	17	2014
14,7	17,9	0,6	3	1	23	2010
14,7	17,9	0,6	3	1	23	2010
14,7	17,9	0,6	3	1	20	2013
14,7	17,9	0,6	4	1	21	2014
14,1	15,8	0,6	2	1	23	2003
12,9	12,1	0,6	2	1	19	2006
12,9	12,1	0,6	2	1	19	2013
15,6	21,4	0,6	6	1	19	2000
15,6	21,4	0,6	4	1	19	2004
17,4	29,7	0,6	4	1	18	2000
17,4	29,7	0,6	10	1	19	2011
15,2	19,8	0,6	4	1	25	2004
15,2	19,8	0,6	4	1	20	2010
15,2	19,8	0,6	4	1	20	2010
13,7	14,5	0,6	3	1	20	2010
13,7	14,5	0,6	3	1	20	2010
13,7	14,5	0,6	3	1	19	2015
15,5	21	0,6	6	1	21	2015

16	23,1	0,6	3	1	26	2003
15,3	20,2	0,6	3	1	26	2003
15,3	20,2	0,6	5	1	25	2004
15,4	20,6	0,6	3	1	25	2003
18	32,9	0,6	10	1	23	2015
14,5	17,2	0,6	3	1	19	2000
14,5	17,2	0,6	5	1	22	2007
14,3	16,5	0,6	6	1	22	2004
14,3	16,5	0,6	2	1	21	2014
13	12,4	0,6	2	1	23	2006
13	12,4	0,6	2	1	22	2009
14,8	18,3	0,6	6	1	19	2008
16,2	24	0,6	6	1	19	2010
16,2	24	0,6	6	1	19	2010
13,6	14,2	0,6	3	1	23	2001
15,9	22,7	0,6	5	1	19	2008
14	15,5	0,6	3	1	25	2004
14	15,5	0,6	3	1	20	2010
14	15,5	0,6	3	1	20	2010

14	15,5	0,6	3	1	23	2011
14	15,5	0,6	2	1	19	2013
12,6	11,3	0,6	2	1	26	2006
13,1	12,7	0,6	2	1	26	2003
13,1	12,7	0,6	4	1	21	2015
17,6	30,8	0,6	6	1	20	2001
13,5	13,9	0,6	2	1	18	2002
13,5	13,9	0,6	2	1	26	2003
13,5	13,9	0,6	2	1	22	2009
13,5	13,9	0,6	4	1	19	2015
19,7	43,2	0,6	6	1	19	2013
16,8	26,8	0,6	4	1	20	2001
13,2	13	0,6	3	1	22	2005
13,2	13	0,6	2	1	26	2006
13,4	13,6	0,6	2	1	25	2002
13,4	13,6	0,6	2	1	23	2002
13,4	13,6	0,6	3	1	20	2011
14,9	18,7	0,6	5	1	26	2006
13,3	13,3	0,6	3	1	23	2010

13,3	13,3	0,6	3	1	23	2010
13,3	13,3	0,6	4	1	21	2011
15,8	22,3	0,6	5	1	21	2010
15,8	22,3	0,6	5	1	21	2010
16,1	23,6	0,6	4	1	19	2013
14,6	17,6	0,6	3	1	26	2006
14,6	17,6	0,6	3	1	20	2008
14,6	17,6	0,6	3	1	21	2014
16,3	24,5	0,6	4	1	21	2000
14,2	16,2	0,6	3	1	23	2004
14,2	16,2	0,6	3	1	19	2008
14,2	16,2	0,6	6	1	22	2011
18	33	0,6	7	1	19	2014
15	19,1	0,6	4	1	25	2004
15	19,1	0,6	3	1	19	2014
13,9	15,2	0,6	2	1	20	2013
13,9	15,2	0,6	2	1	18	2014
13,9	15,2	0,6	3	1	19	2014
14,4	16,9	0,6	2	1	22	2001

14,4	16,9	0,6	3	1	26	2003
15,6	21,5	0,6	5	1	19	2000
15,6	21,5	0,6	4	1	26	2003
15,6	21,5	0,6	5	1	17	2014
15,1	19,5	0,6	3	1	19	2005
15,1	19,5	0,6	4	1	17	2009
15,1	19,5	0,6	7	1	19	2012
15,1	19,5	0,6	3	1	19	2013
15,1	19,5	0,6	3	1	18	2014
15,1	19,5	0,6	3	1	15	2015
16	23,2	0,6	6	1	26	2003
14,7	18	0,6	5	1	19	2000
14,7	18	0,6	5	1	22	2005
15,2	19,9	0,6	3	1	17	2002
15,2	19,9	0,6	4	1	21	2005
15,2	19,9	0,6	3	1	18	2008
15,2	19,9	0,6	4	1	20	2008
15,3	20,3	0,6	3	1	17	2002
15,3	20,3	0,6	6	1	26	2006

16,7	26,4	0,6	4	1	21	2001
13,8	14,9	0,6	2	1	19	2008
13,8	14,9	0,6	2	1	20	2013
12	9,8	0,6	2	1	22	2007
12	9,8	0,6	2	1	22	2007
12	9,8	0,6	2	1	21	2011
14,1	15,9	0,6	2	1	20	2006
14,1	15,9	0,6	4	1	23	2006
14,1	15,9	0,6	2	1	24	2006
14,1	15,9	0,6	4	1	19	2008
15,9	22,8	0,6	7	1	20	2012
12,2	10,3	0,6	2	1	20	2007
14,5	17,3	0,6	7	1	23	2009
14,5	17,3	0,6	6	1	19	2012
14,8	18,4	0,6	4	1	16	2015
14,8	18,4	0,6	4	1	21	2015
14,3	16,6	0,6	3	1	23	2007
13,7	14,6	0,6	3	1	21	2010
13,7	14,6	0,6	3	1	21	2010

17	27,9	0,6	7	1	19	2014
16,1	23,7	0,6	4	1	21	2000
16,1	23,7	0,6	6	1	23	2015
16,3	24,6	0,6	5	1	19	2002
12,9	12,2	0,6	2	1	26	2009
12,9	12,2	0,6	2	1	19	2013
12,5	11,1	0,6	2	1	20	2010
12,5	11,1	0,6	2	1	23	2010
12,5	11,1	0,6	2	1	20	2010
12,5	11,1	0,6	2	1	23	2010
12,5	11,1	0,6	2	1	21	2015
14,9	18,8	0,6	3	1	20	2001
14,9	18,8	0,6	4	1	20	2007
13,6	14,3	0,6	2	1	19	2013
15,7	22	0,6	3	1	18	2002
15,7	22	0,6	3	1	21	2010
15,7	22	0,6	3	1	21	2010
15,7	22	0,6	4	1	16	2015
14	15,6	0,6	5	1	21	2006

14,6	17,7	0,6	8	1	26	2006
16	23,3	0,6	5	1	21	1999
16	23,3	0,6	5	1	20	2001
16	23,3	0,6	4	1	18	2015
18,7	37,2	0,6	5	1	20	2013
15	19,2	0,6	4	1	20	2004
15	19,2	0,6	4	1	20	2011
20,4	48,3	0,6	8	1	19	2013
15,6	21,6	0,6	4	1	20	1999
13	12,5	0,6	3	1	22	2005
13	12,5	0,6	2	1	26	2009
13	12,5	0,6	2	1	21	2010
13	12,5	0,6	2	1	23	2010
13	12,5	0,6	2	1	21	2010
13	12,5	0,6	2	1	23	2010
15,6	21,6	0,6	3	1	19	2013
13,5	14	0,6	3	1	23	1999
13,5	14	0,6	3	1	22	2005
13,5	14	0,6	4	1	23	2009

13,5	14	0,6	3	1	23	2010
13,5	14	0,6	3	1	23	2010
13,5	14	0,6	3	1	23	2010
13,5	14	0,6	3	1	23	2010
16,4	25,1	0,6	4	1	18	2014
14,2	16,3	0,6	3	1	26	2003
15,1	19,6	0,6	5	1	20	2009
15,1	19,6	0,6	3	1	15	2015
15,5	21,2	0,6	4	1	19	2001
15,5	21,2	0,6	4	1	18	2015
14,4	17	0,6	4	1	26	2006
14,4	17	0,6	6	1	23	2010
14,4	17	0,6	6	1	23	2010
13,1	12,8	0,6	3	1	21	2005
13,4	13,7	0,6	2	1	21	2009
13,4	13,7	0,6	3	1	20	2011
13,4	13,7	0,6	2	1	23	2011
15,2	20	0,6	5	1	19	1999
15,2	20	0,6	5	1	21	2000

15,2	20	0,6	3	1	22	2001
15,2	20	0,6	3	1	25	2003
15,2	20	0,6	3	1	19	2014
15,4	20,8	0,6	5	1	22	2003
13,2	13,1	0,6	3	1	19	2007
13,2	13,1	0,6	5	1	22	2007
13,2	13,1	0,6	2	1	22	2009
13,2	13,1	0,6	2	1	20	2013
13,3	13,4	0,6	2	1	21	2006
13,3	13,4	0,6	3	1	21	2008
12,3	10,6	0,6	3	1	22	2005
12,3	10,6	0,6	2	1	23	2010
12,3	10,6	0,6	2	1	23	2010
12,3	10,6	0,6	2	1	21	2011
11,9	9,6	0,6	2	1	21	2005
11,9	9,6	0,6	2	1	19	2006
15,9	22,9	0,6	4	1	19	2012
13,9	15,3	0,6	3	1	20	2001
16,9	27,5	0,6	5	1	18	2004

16,9	27,5	0,6	7	1	17	2009
16,9	27,5	0,6	7	1	18	2012
14,7	18,1	0,6	5	1	20	1999
14,7	18,1	0,6	2	1	20	2000
16,5	25,6	0,6	5	1	21	1999
11,6	8,9	0,6	2	1	18	2007
16,1	23,8	0,6	6	1	21	2001
11,1	7,8	0,6	1	1	20	2007
14,8	18,5	0,6	3	1	18	2000
14,8	18,5	0,6	3	1	19	2010
14,8	18,5	0,6	3	1	19	2010
14,5	17,4	0,6	3	1	21	2003
14,5	17,4	0,6	3	1	19	2009
13,8	15	0,6	5	1	26	2006
13,8	15	0,6	3	1	19	2008
13,8	15	0,6	3	1	23	2010
13,8	15	0,6	3	1	23	2010
14,1	16	0,6	2	1	18	2002
14,1	16	0,6	3	1	20	2006

14,3	16,7	0,6	3	1	20	2002
14,3	16,7	0,6	3	1	20	2012
16,7	26,6	0,6	6	1	21	2003
16,7	26,6	0,6	6	1	22	2011
12,7	11,7	0,6	2	1	20	2013
12,7	11,7	0,6	2	1	18	2014
16	23,4	0,6	4	1	17	2002
18,4	35,6	0,6	7	1	19	2012
13,7	14,7	0,6	3	1	22	2004
13,7	14,7	0,6	2	1	21	2006
13,7	14,7	0,6	2	1	21	2010
13,7	14,7	0,6	3	1	23	2010
13,7	14,7	0,6	2	1	21	2010
13,7	14,7	0,6	3	1	23	2010
13,7	14,7	0,6	2	1	20	2013
15	19,3	0,6	3	1	22	2001
15	19,3	0,6	3	1	20	2006
15	19,3	0,6	3	1	19	2013
15	19,3	0,6	3	1	21	2015

17,2	29,1	0,6	6	1	21	2001
14,6	17,8	0,6	5	1	19	1999
14,6	17,8	0,6	4	1	22	2011
14,6	17,8	0,6	3	1	20	2013
14,6	17,8	0,6	3	1	18	2014
15,5	21,3	0,6	4	1	21	2000
11,8	9,4	0,6	2	1	26	2006
14	15,7	0,6	3	1	25	2003
14	15,7	0,6	3	1	25	2004
14	15,7	0,6	4	1	23	2009
14	15,7	0,6	3	1	22	2011
14	15,7	0,6	5	1	20	2013
15,1	19,7	0,6	3	1	26	2003
15,1	19,7	0,6	4	1	20	2013
12,8	12	0,6	4	1	23	2004
12,8	12	0,6	2	1	26	2006
15,4	20,9	0,6	4	1	23	2001
15,4	20,9	0,6	3	1	18	2014
15,4	20,9	0,6	4	1	18	2015

15,2	20,1	0,6	4	1	15	2015
15,3	20,5	0,6	4	1	18	2001
15,3	20,5	0,6	4	1	18	2001
15,3	20,5	0,6	5	1	26	2001
15,3	20,5	0,6	4	1	25	2004
13,6	14,4	0,6	2	1	21	2009
13,6	14,4	0,6	2	1	21	2010
13,6	14,4	0,6	2	1	21	2010
14,4	17,1	0,6	3	1	20	2011
16,1	23,9	0,6	6	1	21	2005
16,1	23,9	0,6	8	1	23	2005
14,2	16,4	0,6	3	1	18	2008
14,2	16,4	0,6	2	1	21	2015
12	9,9	0,6	2	1	18	2007
12	9,9	0,6	2	1	19	2009
14,7	18,2	0,6	4	1	20	2006
14,7	18,2	0,6	3	1	19	2010
14,7	18,2	0,6	3	1	19	2010
12,9	12,3	0,6	2	1	21	2005

13,5	14,1	0,6	2	1	22	2009
13,5	14,1	0,6	4	1	24	2009
13,5	14,1	0,6	2	1	20	2012
17,4	30,2	0,6	9	1	22	2004
18,1	34	0,6	7	1	19	2012
13	12,6	0,6	3	1	25	2005
13	12,6	0,6	2	1	19	2009
13	12,6	0,6	2	1	22	2009
13	12,6	0,6	2	1	23	2009
13	12,6	0,6	2	1	26	2009
13	12,6	0,6	2	1	19	2013
13	12,6	0,6	2	1	20	2013
13	12,6	0,6	2	1	23	2015
13,4	13,8	0,6	2	1	18	2014
16	23,5	0,6	4	1	20	2001
16	23,5	0,6	3	1	20	2003
14,8	18,6	0,6	4	1	23	2007
14,8	18,6	0,6	4	1	19	2012
13,3	13,5	0,6	3	1	25	2003

13,3	13,5	0,6	2	1	25	2003
13,3	13,5	0,6	3	1	25	2003
13,3	13,5	0,6	3	1	22	2011
17,2	29,2	0,6	5	1	20	2010
17,2	29,2	0,6	5	1	20	2010
16,2	24,4	0,6	4	1	18	2000
16,2	24,4	0,6	7	1	19	2014
13,2	13,2	0,6	2	1	20	2002
17,1	28,7	0,6	5	1	17	2002
17	28,2	0,6	6	1	18	1999
14,5	17,5	0,6	4	1	20	2009
17,6	31,3	0,6	5	1	20	2010
17,6	31,3	0,6	5	1	20	2010
14,1	16,1	0,6	2	1	22	2001
14,1	16,1	0,6	4	1	24	2001
14,1	16,1	0,6	2	1	19	2014
14,1	16,1	0,6	4	1	18	2015
14,3	16,8	0,6	3	1	23	2002
14,3	16,8	0,6	2	1	20	2006

14,3	16,8	0,6	3	1	21	2010
14,3	16,8	0,6	5	1	23	2010
14,3	16,8	0,6	3	1	21	2010
14,3	16,8	0,6	5	1	23	2010
14,3	16,8	0,6	4	1	20	2013
14,3	16,8	0,6	3	1	15	2015
14,3	16,8	0,6	3	1	18	2015
15,5	21,4	0,6	5	1	22	2009
15,5	21,4	0,6	5	1	21	2012
15,5	21,4	0,6	5	1	19	2014
15	19,4	0,6	3	1	26	2003
15	19,4	0,6	4	1	25	2004
15	19,4	0,6	4	1	19	2012
15	19,4	0,6	3	1	18	2014
12,6	11,5	0,6	2	1	23	2008
12,6	11,5	0,6	2	1	23	2009
16,3	24,9	0,6	3	1	19	2012
12,3	10,7	0,6	2	1	26	2006
15,3	20,6	0,6	3	1	20	2003

14,6	17,9	0,6	3	1	26	2003
14,6	17,9	0,6	4	1	21	2013
15,2	20,2	0,6	4	1	21	2000
15,2	20,2	0,6	4	1	20	2012
15,8	22,7	0,6	5	1	18	2000
15,8	22,7	0,6	4	1	20	2013
13,7	14,8	0,6	3	1	22	2015
12,1	10,2	0,6	2	1	18	2007
14	15,8	0,6	3	1	19	1999
14	15,8	0,6	2	1	23	2003
14	15,8	0,6	3	1	18	2008
14	15,8	0,6	4	1	20	2011
14	15,8	0,6	2	1	19	2014
14	15,8	0,6	2	1	21	2015
17,2	29,3	0,6	5	1	17	2014
16,4	25,4	0,6	4	1	19	2002
17	28,3	0,6	4	1	19	2015
14,4	17,2	0,6	3	1	17	2014
12,7	11,8	0,6	2	1	19	2000

12,7	11,8	0,6	2	1	26	2006
12,7	11,8	0,6	2	1	19	2010
12,7	11,8	0,6	2	1	19	2010
14,7	18,3	0,6	3	1	26	2006
14,7	18,3	0,6	3	1	20	2013
14,7	18,3	0,6	3	1	18	2015
16	23,6	0,6	5	1	21	2005
14,2	16,5	0,6	3	1	22	2003
14,2	16,5	0,6	3	1	20	2007
14,2	16,5	0,6	4	1	22	2009
14,2	16,5	0,6	3	1	19	2010
14,2	16,5	0,6	3	1	19	2010
13,6	14,5	0,6	2	1	23	2010
13,6	14,5	0,6	2	1	23	2010
13,6	14,5	0,6	3	1	23	2010
13,6	14,5	0,6	2	1	23	2010
13,6	14,5	0,6	2	1	23	2010
13,6	14,5	0,6	3	1	23	2010
16,5	25,9	0,6	7	1	26	1999

11,6	9	0,6	2	1	22	2009
15,6	21,9	0,6	6	1	26	2000
15,6	21,9	0,6	5	1	19	2010
15,6	21,9	0,6	5	1	19	2010
12,4	11	0,6	2	1	24	2006
12,4	11	0,6	2	1	22	2011
13,5	14,2	0,6	2	1	21	2000
13,5	14,2	0,6	2	1	21	2008
13,5	14,2	0,6	2	1	21	2010
13,5	14,2	0,6	2	1	21	2010
13,5	14,2	0,6	3	1	20	2011
13,9	15,5	0,6	3	1	18	2008
16,3	25	0,6	5	1	24	2009
14,5	17,6	0,6	4	1	20	2004
14,5	17,6	0,6	3	1	20	2010
14,5	17,6	0,6	3	1	20	2010
14,5	17,6	0,6	4	1	18	2015
15,5	21,5	0,6	3	1	23	2001
15,5	21,5	0,6	4	1	22	2004

15,5	21,5	0,6	3	1	21	2010
15,5	21,5	0,6	3	1	21	2010
15,5	21,5	0,6	3	1	19	2014
16,1	24,1	0,6	3	1	21	2015
12,9	12,4	0,6	2	1	24	2006
12,9	12,4	0,6	2	1	26	2006
12,9	12,4	0,6	3	1	20	2008
11,1	7,9	0,6	1	1	18	2007
13,4	13,9	0,6	2	1	20	2000
13,4	13,9	0,6	3	1	22	2005
13,4	13,9	0,6	2	1	18	2014
15,4	21,1	0,6	4	1	18	2002
15	19,5	0,6	4	1	22	2004
15	19,5	0,6	4	1	24	2006
15	19,5	0,6	5	1	21	2010
15	19,5	0,6	5	1	21	2010
17,2	29,4	0,6	4	1	20	2013
14,1	16,2	0,6	3	1	26	2003
14,1	16,2	0,6	4	1	24	2009

14,3	16,9	0,6	4	1	23	2011
14,3	16,9	0,6	4	1	23	2011
14,3	16,9	0,6	4	1	18	2015
14,3	16,9	0,6	4	1	18	2015
15,3	20,7	0,6	5	1	17	2002
15,3	20,7	0,6	3	1	20	2003
15,3	20,7	0,6	3	1	22	2004
15,3	20,7	0,6	5	1	26	2006
15,3	20,7	0,6	5	1	23	2010
15,3	20,7	0,6	5	1	23	2010
15,3	20,7	0,6	8	1	20	2013
15,3	20,7	0,6	3	1	18	2014
15,1	19,9	0,6	6	1	17	2008
15,1	19,9	0,6	4	1	22	2011
15,8	22,8	0,6	4	1	19	2004
15,8	22,8	0,6	6	1	19	2008
15,2	20,3	0,6	3	1	20	2001
15,2	20,3	0,6	5	1	20	2007
15,2	20,3	0,6	5	1	17	2008

13	12,7	0,6	3	1	21	2005
13	12,7	0,6	2	1	21	2006
13	12,7	0,6	2	1	26	2009
13	12,7	0,6	2	1	23	2011
13	12,7	0,6	3	1	18	2015
13,3	13,6	0,6	2	1	25	2003
13,3	13,6	0,6	2	1	20	2013
12,2	10,5	0,6	2	1	20	2010
12,2	10,5	0,6	2	1	20	2010
13,1	13	0,6	2	1	23	2006
13,1	13	0,6	2	1	19	2013
13,1	13	0,6	2	1	20	2013
13,2	13,3	0,6	2	1	20	2002
13,8	15,2	0,6	4	1	20	2009
13,8	15,2	0,6	3	1	18	2015
14,6	18	0,6	3	1	15	2015
12,5	11,3	0,6	2	1	23	1999
12,5	11,3	0,6	3	1	22	2005
12,5	11,3	0,6	3	1	22	2011

12,5	11,3	0,6	2	1	20	2013
16	23,7	0,6	4	1	19	2002
16,2	24,6	0,6	6	1	22	2007
12	10	0,6	2	1	23	2006
16,5	26	0,6	6	1	26	2006
15,7	22,4	0,6	5	1	20	1999
15,7	22,4	0,6	5	1	19	2002
14,7	18,4	0,6	5	1	19	1999
16,6	26,5	0,6	4	1	18	2015
14,4	17,3	0,6	3	1	21	2000
14,4	17,3	0,6	3	1	18	2015
14	15,9	0,6	2	1	20	2006
14	15,9	0,6	3	1	19	2008
14	15,9	0,6	2	1	17	2014
14	15,9	0,6	3	1	19	2014
13,7	14,9	0,6	2	1	20	2002
15,6	22	0,6	3	1	20	2003
15,6	22	0,6	5	1	21	2011
10,7	7,1	0,6	2	1	23	2004

16,3	25,1	0,6	4	1	21	2003
15,9	23,3	0,6	5	1	19	2007
16,7	27	0,6	4	1	23	2001
16,7	27	0,6	6	1	26	2001
16,7	27	0,6	7	1	17	2009
14,2	16,6	0,6	2	1	20	2000
14,2	16,6	0,6	3	1	23	2010
14,2	16,6	0,6	3	1	23	2010
12,6	11,6	0,6	2	1	21	2006
14,8	18,8	0,6	3	1	22	2001
14,8	18,8	0,6	4	1	17	2002
14,8	18,8	0,6	2	1	19	2013
15,5	21,6	0,6	4	1	20	2003
16,9	28	0,6	6	1	20	2013
12,3	10,8	0,6	2	1	23	2009
12,3	10,8	0,6	2	1	22	2011
12,3	10,8	0,6	2	1	18	2014
13,6	14,6	0,6	4	1	26	2006
14,9	19,2	0,6	3	1	20	2010

14,9	19,2	0,6	3	1	20	2010
15,4	21,2	0,6	4	1	20	2006
15,4	21,2	0,6	3	1	21	2010
15,4	21,2	0,6	3	1	21	2010
15,4	21,2	0,6	4	1	21	2013
11,4	8,6	0,6	1	1	26	2009
18,2	35	0,6	10	1	19	2015
14,5	17,7	0,6	5	1	18	2000
14,5	17,7	0,6	5	1	20	2000
14,5	17,7	0,6	2	1	22	2001
15	19,6	0,6	5	1	23	1999
15	19,6	0,6	3	1	18	2007
15	19,6	0,6	4	1	23	2007
15,3	20,8	0,6	3	1	20	2000
13,9	15,6	0,6	4	1	17	2008
13,9	15,6	0,6	3	1	23	2011
13,9	15,6	0,6	3	1	18	2014
13,9	15,6	0,6	2	1	18	2014
15,1	20	0,6	5	1	22	1999

15,1	20	0,6	4	1	23	2004
15,2	20,4	0,6	3	1	21	2003
12,7	11,9	0,6	1	1	26	2001
12,7	11,9	0,6	2	1	23	2009
16,2	24,7	0,6	5	1	18	2000
16,2	24,7	0,6	4	1	18	2014
16,2	24,7	0,6	4	1	19	2014
16	23,8	0,6	4	1	25	2003
16	23,8	0,6	5	1	22	2005
16	23,8	0,6	7	1	26	2006
16	23,8	0,6	3	1	16	2015
13,5	14,3	0,6	2	1	20	2002
18	33,9	0,6	6	1	23	2002
14,3	17	0,6	6	1	23	2010
14,3	17	0,6	6	1	23	2010
12,1	10,3	0,6	2	1	23	2004
15,7	22,5	0,6	5	1	19	2002
14,1	16,3	0,6	6	1	21	2005
14,1	16,3	0,6	4	1	19	2009

14,1	16,3	0,6	3	1	20	2010
14,1	16,3	0,6	3	1	20	2010
17,8	32,8	0,6	4	1	15	2015
14,6	18,1	0,6	3	1	26	2001
14,6	18,1	0,6	3	1	19	2008
12,8	12,2	0,6	2	1	15	2015
13,4	14	0,6	2	1	18	2000
13,4	14	0,6	3	1	19	2002
13,4	14	0,6	2	1	19	2009
16,8	27,6	0,6	3	1	17	2012
11,3	8,4	0,6	1	1	20	2000
16,9	28,1	0,6	5	1	18	2012
13,8	15,3	0,6	2	1	18	2000
13,8	15,3	0,6	3	1	23	2004
13,8	15,3	0,6	2	1	22	2009
13,8	15,3	0,6	3	1	20	2010
13,8	15,3	0,6	3	1	20	2010
12,4	11,1	0,6	2	1	26	2009
12,4	11,1	0,6	2	1	20	2013

16,1	24,3	0,6	4	1	19	2015
18,3	35,7	0,6	4	1	19	2012
13	12,8	0,6	2	1	18	2002
13	12,8	0,6	2	1	25	2007
13	12,8	0,6	2	1	26	2009
13	12,8	0,6	2	1	23	2010
13	12,8	0,6	2	1	23	2010
13	12,8	0,6	2	1	23	2011
13,2	13,4	0,6	2	1	20	2010
13,2	13,4	0,6	2	1	20	2010
13,2	13,4	0,6	2	1	17	2014
13,1	13,1	0,6	3	1	21	2000
13,1	13,1	0,6	3	1	18	2007
13,1	13,1	0,6	2	1	20	2010
13,1	13,1	0,6	2	1	20	2010
13,1	13,1	0,6	4	1	22	2011
13,1	13,1	0,6	2	1	18	2014
14,4	17,4	0,6	5	1	19	1999
14,4	17,4	0,6	7	1	22	2011

15,5	21,7	0,6	5	1	21	2010
15,5	21,7	0,6	5	1	21	2010
14	16	0,6	2	1	21	2000
14	16	0,6	2	1	26	2001
14	16	0,6	2	1	20	2003
14	16	0,6	3	1	18	2008
14	16	0,6	3	1	20	2008
14	16	0,6	4	1	26	2009
14	16	0,6	3	1	21	2014
16,5	26,2	0,6	4	1	22	2015
14,2	16,7	0,6	4	1	24	2009
14,2	16,7	0,6	4	1	26	2009
14,2	16,7	0,6	6	1	21	2011
14,2	16,7	0,6	3	1	23	2011
16,2	24,8	0,6	4	1	17	2014
13,7	15	0,6	2	1	18	2001
13,7	15	0,6	3	1	26	2001
13,7	15	0,6	3	1	21	2009
13,7	15	0,6	3	1	26	2009

13,7	15	0,6	3	1	22	2011
14,9	19,3	0,6	4	1	19	1999
16	23,9	0,6	4	1	15	2015
16	23,9	0,6	8	1	22	2015
15,3	20,9	0,6	7	1	26	2009
12,5	11,4	0,6	2	1	22	2011
15	19,7	0,6	5	1	21	1999
15	19,7	0,6	3	1	19	2008
12,2	10,6	0,6	2	1	23	2009
15,1	20,1	0,6	4	1	20	2008
14,5	17,8	0,6	4	1	19	2000
14,5	17,8	0,6	5	1	26	2006
14,5	17,8	0,6	4	1	24	2009
14,5	17,8	0,6	4	1	16	2015
15,7	22,6	0,6	4	1	22	2004
15,7	22,6	0,6	4	1	19	2011
16,3	25,3	0,6	5	1	24	2002
13,6	14,7	0,6	2	1	24	2001
13,6	14,7	0,6	2	1	18	2002

12	10,1	0,6	2	1	19	2011
16,1	24,4	0,6	5	1	19	2002
16,1	24,4	0,6	5	1	20	2009
15,6	22,2	0,6	5	1	19	2012
14,3	17,1	0,6	4	1	19	1999
14,3	17,1	0,6	3	1	26	2001
14,3	17,1	0,6	2	1	18	2002
14,3	17,1	0,6	4	1	22	2004
14,3	17,1	0,6	2	1	25	2004
14,3	17,1	0,6	4	1	20	2013
14,6	18,2	0,6	5	1	20	2007
12,6	11,7	0,6	2	1	18	2008
14,1	16,4	0,6	4	1	21	2006
14,1	16,4	0,6	2	1	19	2013
14,1	16,4	0,6	3	1	18	2014
14,1	16,4	0,6	3	1	19	2014
14,1	16,4	0,6	3	1	18	2015
11,5	8,9	0,6	2	1	20	2004
11,5	8,9	0,6	2	1	22	2005

17,3	30,3	0,6	5	1	20	2002
13,5	14,4	0,6	2	1	20	2010
13,5	14,4	0,6	2	1	20	2010
13,5	14,4	0,6	2	1	19	2014
13,5	14,4	0,6	2	1	23	2015
15,5	21,8	0,6	5	1	18	1999
15,5	21,8	0,6	3	1	20	2003
15,5	21,8	0,6	5	1	23	2010
15,5	21,8	0,6	5	1	23	2010
16,5	26,3	0,6	4	1	19	2002
14,7	18,6	0,6	4	1	22	2004
14,7	18,6	0,6	5	1	21	2005
14,7	18,6	0,6	3	1	22	2015
15,8	23,1	0,6	5	1	21	2000
15,8	23,1	0,6	4	1	21	2010
15,8	23,1	0,6	4	1	21	2010
12,7	12	0,6	2	1	22	1999
16,6	26,8	0,6	6	1	20	2010
16,6	26,8	0,6	6	1	20	2010

16	24	0,6	6	1	22	1999
16	24	0,6	3	1	22	2003
15,4	21,4	0,6	6	1	19	2000
17,1	29,3	0,6	6	1	21	2005
13,4	14,1	0,6	2	1	20	2002
13,4	14,1	0,6	5	1	21	2006
13,4	14,1	0,6	3	1	19	2009
14,4	17,5	0,6	5	1	26	2009
14,4	17,5	0,6	3	1	19	2015
15,3	21	0,6	3	1	19	2010
15,3	21	0,6	3	1	19	2010
14,9	19,4	0,6	3	1	26	2003
16,3	25,4	0,6	6	1	19	2000
13,3	13,8	0,6	2	1	20	2002
13,3	13,8	0,6	2	1	20	2002
13,3	13,8	0,6	3	1	20	2011
15,2	20,6	0,6	3	1	20	2003
15	19,8	0,6	5	1	20	1999
15	19,8	0,6	3	1	21	2000

15	19,8	0,6	4	1	20	2004
15	19,8	0,6	4	1	23	2006
15	19,8	0,6	4	1	20	2009
15	19,8	0,6	6	1	19	2011
15	19,8	0,6	4	1	22	2011
15	19,8	0,6	4	1	17	2012
15	19,8	0,6	3	1	19	2013
15	19,8	0,6	4	1	21	2014
15	19,8	0,6	3	1	18	2015
15,1	20,2	0,6	5	1	22	1999
15,1	20,2	0,6	6	1	20	2000
15,1	20,2	0,6	3	1	20	2001
14	16,1	0,6	3	1	26	2003
14	16,1	0,6	3	1	21	2005
14	16,1	0,6	2	1	20	2008
14	16,1	0,6	3	1	19	2014
14,2	16,8	0,6	5	1	26	2006
14,2	16,8	0,6	3	1	23	2008
11,7	9,4	0,6	1	1	23	2009

12,9	12,6	0,6	2	1	26	2005
13,2	13,5	0,6	2	1	20	2001
13,2	13,5	0,6	4	1	22	2004
13,2	13,5	0,6	3	1	23	2010
13,2	13,5	0,6	3	1	23	2010
13,2	13,5	0,6	2	1	19	2013
13,2	13,5	0,6	2	1	20	2013
13,2	13,5	0,6	2	1	18	2014
15,9	23,6	0,6	3	1	17	2008
17,3	30,4	0,6	3	1	21	2010
17,3	30,4	0,6	3	1	21	2010
14,5	17,9	0,6	3	1	20	2001
14,5	17,9	0,6	3	1	20	2002
14,5	17,9	0,6	3	1	18	2007
14,5	17,9	0,6	3	1	20	2013
13	12,9	0,6	2	1	21	2000
13	12,9	0,6	4	1	23	2001
13	12,9	0,6	3	1	22	2005
13	12,9	0,6	2	1	26	2006

13	12,9	0,6	4	1	26	2009
13	12,9	0,6	2	1	17	2014
13	12,9	0,6	2	1	21	2014
13	12,9	0,6	2	1	21	2014
13,1	13,2	0,6	2	1	21	2006
13,1	13,2	0,6	3	1	23	2008
13,7	15,1	0,6	4	1	25	2004
10,8	7,4	0,6	2	1	25	2004
16,5	26,4	0,6	6	1	18	2014
14,6	18,3	0,6	3	1	21	2000
14,6	18,3	0,6	3	1	25	2003
16,2	25	0,6	4	1	20	2012
15,5	21,9	0,6	7	1	23	2005
14,3	17,2	0,6	3	1	19	2008
14,3	17,2	0,6	2	1	20	2008
14,3	17,2	0,6	3	1	22	2011
14,3	17,2	0,6	3	1	19	2015
16,7	27,4	0,6	5	1	19	2000
16,7	27,4	0,6	4	1	18	2002

13,9	15,8	0,6	2	1	25	2002
13,9	15,8	0,6	4	1	21	2005
13,9	15,8	0,6	2	1	18	2015
13,6	14,8	0,6	2	1	22	2001
13,6	14,8	0,6	2	1	26	2006
13,6	14,8	0,6	3	1	19	2007
13,6	14,8	0,6	3	1	23	2009
16	24,1	0,6	7	1	19	2001
16	24,1	0,6	3	1	23	2001
16	24,1	0,6	7	1	25	2007
16	24,1	0,6	3	1	20	2012
16	24,1	0,6	3	1	15	2015
16	24,1	0,6	3	1	16	2015
14,1	16,5	0,6	2	1	24	2001
14,1	16,5	0,6	4	1	20	2009
14,1	16,5	0,6	3	1	18	2014
15,4	21,5	0,6	4	1	23	2003
14,7	18,7	0,6	3	1	23	2010
14,7	18,7	0,6	3	1	23	2010

14,7	18,7	0,6	3	1	17	2014
12,5	11,5	0,6	2	1	20	2013
16,3	25,5	0,6	4	1	19	2012
18,2	35,5	0,6	5	1	20	2010
18,2	35,5	0,6	5	1	20	2010
11,3	8,5	0,6	1	1	22	2007
15,3	21,1	0,6	5	1	23	1999
15,7	22,8	0,6	3	1	19	2001
14,8	19,1	0,6	3	1	26	2003
14,8	19,1	0,6	4	1	21	2006
14,8	19,1	0,6	4	1	22	2009
12,2	10,7	0,6	3	1	25	2004
13,5	14,5	0,6	3	1	19	2002
13,5	14,5	0,6	2	1	22	2011
13,5	14,5	0,6	3	1	21	2014
14,4	17,6	0,6	4	1	18	1999
14,4	17,6	0,6	3	1	19	2002
14,4	17,6	0,6	4	1	22	2004
15,2	20,7	0,6	5	1	17	2014

16,4	26	0,6	3	1	23	2001
16,1	24,6	0,6	6	1	20	2001
16,1	24,6	0,6	5	1	19	2010
16,1	24,6	0,6	5	1	19	2010
16,1	24,6	0,6	7	1	18	2014
14,9	19,5	0,6	3	1	21	2006
14,9	19,5	0,6	3	1	19	2012
17,2	30	0,6	5	1	20	2010
17,2	30	0,6	5	1	20	2010
15,1	20,3	0,6	5	1	24	2006
15,1	20,3	0,6	5	1	26	2006
15,1	20,3	0,6	7	1	20	2011
15	19,9	0,6	3	1	26	2006
15	19,9	0,6	7	1	20	2009
15	19,9	0,6	3	1	18	2012
15	19,9	0,6	5	1	19	2012
15	19,9	0,6	3	1	21	2014
13,8	15,5	0,6	3	1	25	2003
13,8	15,5	0,6	3	1	18	2008

13,8	15,5	0,6	3	1	18	2014
16,5	26,5	0,6	5	1	20	2013
10	5,9	0,6	1	1	24	2009
15,6	22,4	0,6	4	1	20	2008
13,4	14,2	0,6	4	1	19	2006
13,4	14,2	0,6	4	1	26	2009
14,2	16,9	0,6	2	1	26	2001
14,2	16,9	0,6	3	1	24	2002
16,6	27	0,6	3	1	22	2003
16,6	27	0,6	5	1	17	2014
12	10,2	0,6	2	1	20	2007
12	10,2	0,6	2	1	23	2007
11,8	9,7	0,6	2	1	23	2009
14	16,2	0,6	4	1	23	2006
14	16,2	0,6	2	1	22	2009
14	16,2	0,6	3	1	17	2014
14	16,2	0,6	3	1	19	2014
14,5	18	0,6	2	1	26	2001
14,5	18	0,6	2	1	22	2003

17,8	33,3	0,6	5	1	21	2013
12,7	12,1	0,6	2	1	26	2006
11,2	8,3	0,6	2	1	19	2006
16	24,2	0,6	3	1	20	2003
16	24,2	0,6	4	1	23	2003
16	24,2	0,6	4	1	19	2013
13,3	13,9	0,6	2	1	18	2000
13,3	13,9	0,6	4	1	22	2009
13,3	13,9	0,6	2	1	20	2013
13,3	13,9	0,6	3	1	18	2014
16,3	25,6	0,6	4	1	24	2001
16,3	25,6	0,6	4	1	18	2002
12,3	11	0,6	2	1	22	2005
13,7	15,2	0,6	3	1	23	2003
13,7	15,2	0,6	3	1	22	2005
13,7	15,2	0,6	3	1	19	2012
13,7	15,2	0,6	2	1	19	2013
19,3	42,5	0,6	5	1	20	2012
14,6	18,4	0,6	5	1	19	1999

14,6	18,4	0,6	4	1	21	1999
14,6	18,4	0,6	3	1	26	2003
12,8	12,4	0,6	3	1	23	2005
12,8	12,4	0,6	2	1	23	2005
12,8	12,4	0,6	2	1	23	2007
13,2	13,6	0,6	2	1	23	2001
13,2	13,6	0,6	3	1	21	2005
15,4	21,6	0,6	3	1	20	2003
15,4	21,6	0,6	3	1	15	2015
15,4	21,6	0,6	6	1	21	2015
13,1	13,3	0,6	3	1	21	2005
13,1	13,3	0,6	2	1	23	2006
13,1	13,3	0,6	3	1	20	2008
14,3	17,3	0,6	3	1	26	2003
14,3	17,3	0,6	2	1	20	2013
14,3	17,3	0,6	3	1	21	2014
14,3	17,3	0,6	3	1	23	2014
14,3	17,3	0,6	3	1	16	2015
16,4	26,1	0,6	6	1	24	2001

13	13	0,6	3	1	25	2007
13	13	0,6	4	1	20	2009
13	13	0,6	2	1	23	2009
13	13	0,6	2	1	20	2011
13	13	0,6	3	1	17	2014
15,7	22,9	0,6	3	1	23	2003
15,7	22,9	0,6	3	1	18	2014
14,7	18,8	0,6	3	1	22	2005
16,1	24,7	0,6	3	1	18	2001
15,3	21,2	0,6	3	1	19	2002
15,3	21,2	0,6	3	1	20	2003
13,9	15,9	0,6	3	1	20	2008
15,9	23,8	0,6	5	1	19	2005
15,9	23,8	0,6	4	1	19	2013
14,1	16,6	0,6	4	1	23	2007
14,1	16,6	0,6	3	1	15	2015
14,8	19,2	0,6	3	1	20	2001
14,8	19,2	0,6	2	1	25	2003
14,8	19,2	0,6	4	1	25	2004

11,1	8,1	0,6	2	1	22	2007
15,2	20,8	0,6	3	1	18	2002
15,2	20,8	0,6	4	1	22	2007
13,6	14,9	0,6	2	1	19	2013
13,6	14,9	0,6	3	1	23	2015
17,6	32,3	0,6	7	1	25	2004
14,9	19,6	0,6	8	1	26	2006
14,9	19,6	0,6	5	1	18	2007
15,1	20,4	0,6	3	1	21	2001
15,1	20,4	0,6	3	1	25	2002
15,1	20,4	0,6	6	1	20	2011
15	20	0,6	3	1	17	2002
15	20	0,6	3	1	17	2014
15	20	0,6	4	1	19	2014
15,6	22,5	0,6	4	1	21	2001
15,6	22,5	0,6	8	1	19	2002
15,6	22,5	0,6	5	1	24	2002
15,6	22,5	0,6	4	1	17	2009
15,6	22,5	0,6	4	1	22	2009

15,6	22,5	0,6	4	1	19	2013
12,1	10,5	0,6	2	1	24	2006
12,1	10,5	0,6	2	1	20	2007
12,1	10,5	0,6	3	1	20	2008
14,4	17,7	0,6	3	1	20	2001
14,4	17,7	0,6	3	1	19	2008
14,4	17,7	0,6	3	1	18	2012
11,7	9,5	0,6	2	1	20	2004
11,7	9,5	0,6	2	1	25	2004
15,8	23,4	0,6	8	1	21	2015
16	24,3	0,6	3	1	19	2013
13,5	14,6	0,6	2	1	21	2000
13,5	14,6	0,6	2	1	19	2008
13,5	14,6	0,6	2	1	18	2014
16,3	25,7	0,6	5	1	21	2010
16,3	25,7	0,6	5	1	21	2010
16,3	25,7	0,6	5	1	18	2015
15,5	22,1	0,6	4	1	20	2011
15,5	22,1	0,6	3	1	19	2011

15,5	22,1	0,6	3	1	21	2015
17,2	30,2	0,6	8	1	16	2008
17,2	30,2	0,6	5	1	19	2013
13,8	15,6	0,6	4	1	19	1999
13,8	15,6	0,6	3	1	22	2005
13,8	15,6	0,6	3	1	19	2014
18,1	35,2	0,6	5	1	17	2014
10,2	6,3	0,6	1	1	26	2009
14,5	18,1	0,6	3	1	23	2003
14,5	18,1	0,6	3	1	25	2003
14,5	18,1	0,6	3	1	19	2010
14,5	18,1	0,6	3	1	19	2010
14,2	17	0,6	3	1	25	2003
14,2	17	0,6	4	1	20	2004
14,2	17	0,6	4	1	23	2006
14,2	17	0,6	3	1	19	2007
14,2	17	0,6	3	1	23	2009
14,2	17	0,6	4	1	22	2011
12,5	11,6	0,6	2	1	23	2001

12,5	11,6	0,6	2	1	23	2010
12,5	11,6	0,6	2	1	23	2010
11,4	8,8	0,6	2	1	23	2004
14	16,3	0,6	3	1	20	2006
14	16,3	0,6	3	1	20	2007
14	16,3	0,6	5	1	25	2007
14	16,3	0,6	3	1	17	2009
14	16,3	0,6	3	1	20	2011
14	16,3	0,6	3	1	19	2013
15,4	21,7	0,6	3	1	19	2001
15,4	21,7	0,6	4	1	23	2005
13,4	14,3	0,6	2	1	22	2009
15,7	23	0,6	8	1	23	2010
15,7	23	0,6	8	1	23	2010
17	29,2	0,6	6	1	20	2011
16,5	26,7	0,6	5	1	20	1999
16,5	26,7	0,6	5	1	21	2010
16,5	26,7	0,6	5	1	21	2010
14,6	18,5	0,6	3	1	25	2003

14,6	18,5	0,6	3	1	22	2007
14,6	18,5	0,6	4	1	20	2011
15,9	23,9	0,6	3	1	26	2000
15,3	21,3	0,6	5	1	20	1999
15,3	21,3	0,6	3	1	18	2014
15,3	21,3	0,6	3	1	18	2015
16,7	27,7	0,6	6	1	20	2013
16,7	27,7	0,6	7	1	19	2014
12,2	10,8	0,6	3	1	21	2005
12,6	11,9	0,6	2	1	25	2007
12,6	11,9	0,6	2	1	23	2009
14,7	18,9	0,6	3	1	22	2001
14,7	18,9	0,6	4	1	19	2004
14,7	18,9	0,6	3	1	23	2011
14,7	18,9	0,6	3	1	19	2014
18	34,7	0,6	4	1	15	2015
13,7	15,3	0,6	3	1	22	2005
13,7	15,3	0,6	4	1	20	2009
13,7	15,3	0,6	3	1	15	2015

14,3	17,4	0,6	2	1	24	2001
14,3	17,4	0,6	3	1	21	2003
13,3	14	0,6	2	1	22	2000
13,3	14	0,6	4	1	23	2006
13,3	14	0,6	2	1	26	2006
13,3	14	0,6	2	1	26	2009
13,3	14	0,6	2	1	20	2011
13,3	14	0,6	3	1	17	2014
13,3	14	0,6	3	1	15	2015
16,2	25,3	0,6	5	1	19	1999
16,2	25,3	0,6	6	1	20	2010
16,2	25,3	0,6	6	1	20	2010
15,2	20,9	0,6	4	1	17	2002
15,2	20,9	0,6	3	1	19	2013
15,2	20,9	0,6	3	1	19	2014
15,6	22,6	0,6	5	1	19	2010
15,6	22,6	0,6	5	1	19	2010
15,6	22,6	0,6	4	1	20	2012
18,1	35,3	0,6	8	1	21	2013

14,8	19,3	0,6	5	1	26	2006
14,8	19,3	0,6	4	1	26	2006
14,8	19,3	0,6	3	1	19	2015
15,1	20,5	0,6	5	1	23	2007
15,1	20,5	0,6	5	1	19	2012
15,1	20,5	0,6	4	1	18	2015
15	20,1	0,6	3	1	23	2002
15	20,1	0,6	3	1	20	2003
15	20,1	0,6	4	1	24	2009
12,7	12,2	0,6	3	1	21	2005
12,7	12,2	0,6	2	1	21	2011
10,4	6,7	0,6	1	1	22	2009
13,2	13,7	0,6	2	1	18	2000
13,2	13,7	0,6	2	1	21	2001
13,2	13,7	0,6	2	1	19	2006
13,2	13,7	0,6	2	1	24	2006
13,2	13,7	0,6	2	1	19	2009
13,2	13,7	0,6	2	1	19	2013
13,2	13,7	0,6	2	1	21	2013

13,2	13,7	0,6	2	1	19	2014
16	24,4	0,6	5	1	21	2000
16,3	25,8	0,6	6	1	19	2013
16,3	25,8	0,6	4	1	20	2013
14,1	16,7	0,6	6	1	23	2008
14,1	16,7	0,6	4	1	20	2009
14,1	16,7	0,6	2	1	21	2015
13,9	16	0,6	2	1	26	2003
13,9	16	0,6	2	1	19	2014
20,9	54,4	0,6	5	1	18	2012
12,8	12,5	0,6	3	1	19	2000
12,8	12,5	0,6	2	1	26	2006
12,8	12,5	0,6	2	1	20	2009
13,1	13,4	0,6	2	1	21	2009
12	10,3	0,6	2	1	20	2007
12	10,3	0,6	2	1	22	2007
12	10,3	0,6	2	1	23	2007
14,4	17,8	0,6	5	1	22	1999
14,4	17,8	0,6	3	1	20	2010

14,4	17,8	0,6	3	1	20	2010
14,4	17,8	0,6	3	1	21	2014
14,4	17,8	0,6	3	1	15	2015
15,5	22,2	0,6	5	1	18	2000
15,5	22,2	0,6	5	1	18	2007
15,5	22,2	0,6	6	1	20	2013
16,4	26,3	0,6	6	1	19	2001
12,9	12,8	0,6	3	1	20	2007
12,9	12,8	0,6	2	1	20	2007
12,9	12,8	0,6	2	1	19	2013
12,9	12,8	0,6	2	1	17	2014
13	13,1	0,6	2	1	21	2000
13	13,1	0,6	2	1	21	2013
13,6	15	0,6	2	1	19	2001
17	29,3	0,6	7	1	18	2014
12,3	11,1	0,6	2	1	26	2006
12,3	11,1	0,6	2	1	20	2009
16,1	24,9	0,6	4	1	20	2011
16,1	24,9	0,6	5	1	19	2012

16,1	24,9	0,6	4	1	18	2015
16,9	28,8	0,6	5	1	20	2000
9,3	4,8	0,6	1	1	20	2004
15,4	21,8	0,6	3	1	24	2001
15,4	21,8	0,6	3	1	21	2003
15,4	21,8	0,6	5	1	19	2006
15,4	21,8	0,6	5	1	19	2007
16,7	27,8	0,6	3	1	15	2015
15,7	23,1	0,6	4	1	20	1999
15,7	23,1	0,6	4	1	25	2004
15,7	23,1	0,6	5	1	20	2013
15,9	24	0,6	4	1	21	2010
15,9	24	0,6	4	1	21	2010
14,2	17,1	0,6	3	1	18	1999
14,2	17,1	0,6	3	1	23	2003
14,2	17,1	0,6	3	1	22	2005
14,2	17,1	0,6	3	1	19	2014
13,8	15,7	0,6	4	1	23	2011
13,8	15,7	0,6	2	1	22	2013

13,5	14,7	0,6	4	1	22	2011
13,5	14,7	0,6	2	1	21	2014
15,3	21,4	0,6	5	1	25	2004
14,6	18,6	0,6	3	1	19	2000
14,6	18,6	0,6	3	1	23	2015
14	16,4	0,6	3	1	19	1999
14	16,4	0,6	4	1	20	2006
14	16,4	0,6	3	1	18	2007
14	16,4	0,6	5	1	23	2007
14	16,4	0,6	3	1	20	2008
14	16,4	0,6	4	1	20	2008
14	16,4	0,6	3	1	23	2008
14	16,4	0,6	3	1	19	2014
14	16,4	0,6	4	1	19	2014
12,4	11,4	0,6	3	1	25	2004
12,4	11,4	0,6	2	1	26	2009
12,4	11,4	0,6	2	1	22	2011
15,2	21	0,6	3	1	22	2001
15,2	21	0,6	5	1	24	2001

15,2	21	0,6	5	1	20	2012
16,3	25,9	0,6	8	1	21	2001
14,7	19	0,6	3	1	18	2001
14,7	19	0,6	2	1	24	2001
14,7	19	0,6	4	1	21	2011
16	24,5	0,6	4	1	23	2007
16	24,5	0,6	4	1	17	2012
15,8	23,6	0,6	3	1	20	2003
14,8	19,4	0,6	5	1	20	2001
14,8	19,4	0,6	4	1	23	2005
14,8	19,4	0,6	4	1	21	2015
14,3	17,5	0,6	3	1	19	2000
14,3	17,5	0,6	3	1	15	2015
13,4	14,4	0,6	2	1	20	2002
13,4	14,4	0,6	2	1	20	2002
13,4	14,4	0,6	3	1	21	2008
13,4	14,4	0,6	2	1	24	2009
13,4	14,4	0,6	2	1	19	2014
15	20,2	0,6	3	1	23	2003

15	20,2	0,6	4	1	19	2004
15	20,2	0,6	4	1	22	2011
15	20,2	0,6	3	1	19	2014
15	20,2	0,6	4	1	18	2015
14,9	19,8	0,6	5	1	24	2006
14,9	19,8	0,6	4	1	24	2006
16,9	28,9	0,6	7	1	18	2014
15,5	22,3	0,6	3	1	21	2003
15,5	22,3	0,6	5	1	19	2007
13,7	15,4	0,6	2	1	25	2002
13,7	15,4	0,6	2	1	24	2009
13,7	15,4	0,6	2	1	23	2011
16,7	27,9	0,6	4	1	18	2002
16,7	27,9	0,6	7	1	24	2009
12,5	11,7	0,6	2	1	23	2006
16,1	25	0,6	5	1	23	2001
16,1	25	0,6	5	1	21	2006
16,1	25	0,6	5	1	19	2007
14,1	16,8	0,6	4	1	23	2011

14,1	16,8	0,6	4	1	23	2011
14,1	16,8	0,6	2	1	19	2014
13,3	14,1	0,6	2	1	20	2000
13,3	14,1	0,6	2	1	20	2002
13,3	14,1	0,6	3	1	21	2005
13,3	14,1	0,6	3	1	21	2008
13,3	14,1	0,6	2	1	26	2009
13,3	14,1	0,6	2	1	23	2010
13,3	14,1	0,6	2	1	23	2010
11,7	9,6	0,6	2	1	20	2004
11,7	9,6	0,6	3	1	25	2004
17,2	30,5	0,6	4	1	17	2012
14,4	17,9	0,6	5	1	26	1999
14,4	17,9	0,6	3	1	24	2001
14,4	17,9	0,6	3	1	15	2015
13,9	16,1	0,6	3	1	19	2014
15,7	23,2	0,6	4	1	19	2013
15,7	23,2	0,6	3	1	20	2013
15,7	23,2	0,6	4	1	15	2015

15,9	24,1	0,6	4	1	20	2006
15,9	24,1	0,6	7	1	20	2011
15,4	21,9	0,6	4	1	18	1999
15,4	21,9	0,6	4	1	18	2015
15,4	21,9	0,6	4	1	18	2015
16,2	25,5	0,6	4	1	17	2014
17,4	31,6	0,6	5	1	19	2002
12,6	12	0,6	2	1	23	2010
12,6	12	0,6	2	1	23	2010
19,1	41,8	0,6	5	1	18	2012
13,2	13,8	0,6	2	1	22	2001
13,2	13,8	0,6	2	1	23	2002
13,2	13,8	0,6	2	1	18	2008
13,2	13,8	0,6	2	1	23	2009
13,2	13,8	0,6	2	1	22	2011
13,2	13,8	0,6	2	1	19	2014
18	35	0,6	6	1	19	2013
13,6	15,1	0,6	5	1	20	2000
13,6	15,1	0,6	3	1	19	2010

13,6	15,1	0,6	3	1	19	2010
13,6	15,1	0,6	2	1	19	2013
13,6	15,1	0,6	2	1	21	2013
15,3	21,5	0,6	5	1	19	1999
15,3	21,5	0,6	5	1	19	2002
15,3	21,5	0,6	5	1	23	2005
15,3	21,5	0,6	7	1	19	2006
16,3	26	0,6	5	1	18	2000
16,3	26	0,6	4	1	22	2004
16,3	26	0,6	4	1	19	2013
19,7	45,9	0,6	5	1	18	2014
17	29,5	0,6	7	1	21	2014
12,7	12,3	0,6	2	1	22	2009
13,1	13,5	0,6	3	1	21	2005
13,1	13,5	0,6	2	1	26	2006
13,1	13,5	0,6	4	1	22	2009
13,1	13,5	0,6	2	1	17	2012
17,7	33,3	0,6	5	1	19	2010
17,7	33,3	0,6	5	1	19	2010

15,6	22,8	0,6	3	1	20	2003
15,6	22,8	0,6	3	1	19	2010
15,6	22,8	0,6	3	1	19	2010
16	24,6	0,6	4	1	19	2001
16	24,6	0,6	4	1	20	2001
16	24,6	0,6	5	1	23	2005
16	24,6	0,6	4	1	20	2013
17,3	31,1	0,6	4	1	19	2010
17,3	31,1	0,6	4	1	19	2010
14,2	17,2	0,6	3	1	18	2008
14,2	17,2	0,6	3	1	23	2008
14,2	17,2	0,6	3	1	19	2010
14,2	17,2	0,6	5	1	23	2010
14,2	17,2	0,6	3	1	19	2010
14,2	17,2	0,6	5	1	23	2010
14,2	17,2	0,6	3	1	18	2014
14,2	17,2	0,6	4	1	19	2015
11,4	8,9	0,6	2	1	20	2004
16,4	26,5	0,6	5	1	26	2003

16,4	26,5	0,6	7	1	19	2009
12,8	12,6	0,6	2	1	18	2002
12,8	12,6	0,6	2	1	26	2006
12,8	12,6	0,6	2	1	19	2013
12,8	12,6	0,6	2	1	21	2014
13	13,2	0,6	2	1	26	2006
13	13,2	0,6	5	1	23	2007
13	13,2	0,6	2	1	22	2009
13	13,2	0,6	2	1	23	2009
13	13,2	0,6	2	1	23	2011
13	13,2	0,6	2	1	19	2013
15,2	21,1	0,6	4	1	18	2001
14,6	18,7	0,6	2	1	24	2001
14,6	18,7	0,6	2	1	19	2012
14,6	18,7	0,6	3	1	21	2015
12,9	12,9	0,6	2	1	24	2009
12,9	12,9	0,6	2	1	19	2013
16,5	27	0,6	4	1	15	2015
16,8	28,5	0,6	5	1	21	2010

16,8	28,5	0,6	5	1	21	2010
13,8	15,8	0,6	2	1	20	2008
15,1	20,7	0,6	4	1	20	2006
15,1	20,7	0,6	6	1	19	2008
14,7	19,1	0,6	3	1	18	2000
14	16,5	0,6	2	1	20	2013
14	16,5	0,6	3	1	17	2014
14	16,5	0,6	3	1	19	2014
15	20,3	0,6	3	1	19	2001
15	20,3	0,6	4	1	23	2006
15	20,3	0,6	4	1	18	2008
15	20,3	0,6	4	1	15	2015
14,8	19,5	0,6	4	1	20	2001
14,8	19,5	0,6	5	1	19	2009
14,8	19,5	0,6	5	1	19	2009
14,8	19,5	0,6	4	1	20	2011
15,5	22,4	0,6	3	1	22	2003
15,5	22,4	0,6	3	1	19	2015
13,5	14,8	0,6	2	1	22	2000

13,5	14,8	0,6	4	1	23	2006
13,5	14,8	0,6	2	1	22	2009
13,5	14,8	0,6	2	1	19	2013
13,5	14,8	0,6	2	1	18	2014
13,5	14,8	0,6	2	1	21	2014
13,5	14,8	0,6	2	1	23	2014
13,5	14,8	0,6	3	1	18	2015
14,9	19,9	0,6	5	1	18	2000
14,9	19,9	0,6	4	1	26	2003
14,9	19,9	0,6	4	1	20	2012
12,3	11,2	0,6	2	1	21	2009
14,3	17,6	0,6	3	1	18	1999
14,3	17,6	0,6	3	1	19	2001
14,3	17,6	0,6	3	1	18	2008
14,3	17,6	0,6	4	1	22	2011
14,3	17,6	0,6	3	1	19	2012
14,3	17,6	0,6	3	1	18	2015
15,9	24,2	0,6	5	1	26	2001
18,1	35,7	0,6	4	1	15	2015

15,7	23,3	0,6	4	1	22	2003
15,7	23,3	0,6	5	1	19	2010
15,7	23,3	0,6	5	1	19	2010
16,2	25,6	0,6	6	1	26	2003
20,8	54,2	0,6	8	1	18	2012
17,7	33,4	0,6	4	1	19	2013
15,4	22	0,6	4	1	17	2012
13,4	14,5	0,6	3	1	24	2001
13,4	14,5	0,6	3	1	21	2003
19,9	47,5	0,6	6	1	18	2012
13,7	15,5	0,6	3	1	20	2004
14,4	18	0,6	3	1	18	2012
14,1	16,9	0,6	3	1	26	1999
14,1	16,9	0,6	2	1	26	2001
14,1	16,9	0,6	4	1	21	2006
14,1	16,9	0,6	3	1	18	2014
14,1	16,9	0,6	3	1	22	2015
18,6	38,8	0,6	7	1	19	2000
16	24,7	0,6	4	1	21	1999

16	24,7	0,6	6	1	24	2001
16,4	26,6	0,6	5	1	23	2004
15,3	21,6	0,6	3	1	18	2001
15,3	21,6	0,6	4	1	20	2001
15,3	21,6	0,6	4	1	25	2003
12,4	11,5	0,6	1	1	26	2001
12,4	11,5	0,6	3	1	25	2004
12,4	11,5	0,6	2	1	18	2014
15,6	22,9	0,6	4	1	23	2003
15,6	22,9	0,6	5	1	23	2011
13,9	16,2	0,6	3	1	23	2007
13,9	16,2	0,6	3	1	17	2008
17,2	30,7	0,6	4	1	16	2015
16,7	28,1	0,6	5	1	20	2011
16,6	27,6	0,6	9	1	19	2014
14,5	18,4	0,6	3	1	18	2015
13,3	14,2	0,6	2	1	26	2001
13,3	14,2	0,6	3	1	20	2002
13,3	14,2	0,6	3	1	21	2011

13,3	14,2	0,6	3	1	22	2011
15,2	21,2	0,6	4	1	20	1999
15,2	21,2	0,6	3	1	21	2001
15,2	21,2	0,6	3	1	26	2001
15,2	21,2	0,6	3	1	23	2002
15,2	21,2	0,6	8	1	26	2005
12,1	10,7	0,6	2	1	20	2007
14,6	18,8	0,6	3	1	23	2006
17,7	33,5	0,6	6	1	20	2010
17,7	33,5	0,6	6	1	20	2010
15,1	20,8	0,6	4	1	20	1999
15,1	20,8	0,6	3	1	20	2003
15,1	20,8	0,6	4	1	23	2007
12,5	11,8	0,6	2	1	22	2005
14,2	17,3	0,6	3	1	18	2002
14,2	17,3	0,6	4	1	23	2009
14,2	17,3	0,6	3	1	22	2011
14,2	17,3	0,6	3	1	16	2015
15,5	22,5	0,6	4	1	21	2005

15,5	22,5	0,6	5	1	19	2010
15,5	22,5	0,6	5	1	19	2010
15,5	22,5	0,6	5	1	19	2012
13,6	15,2	0,6	3	1	22	1999
13,6	15,2	0,6	2	1	20	2000
13,6	15,2	0,6	8	1	26	2001
13,6	15,2	0,6	5	1	20	2006
13,2	13,9	0,6	2	1	20	2002
13,2	13,9	0,6	3	1	19	2008
13,2	13,9	0,6	3	1	19	2009
13,2	13,9	0,6	2	1	19	2013
14,7	19,2	0,6	5	1	21	2000
14,7	19,2	0,6	3	1	24	2002
14,7	19,2	0,6	3	1	25	2004
14,7	19,2	0,6	3	1	18	2014
14,7	19,2	0,6	3	1	19	2014
14,7	19,2	0,6	3	1	15	2015
15	20,4	0,6	5	1	18	2000
15	20,4	0,6	4	1	19	2006

15	20,4	0,6	3	1	20	2013
15	20,4	0,6	3	1	19	2014
15	20,4	0,6	5	1	19	2014
15	20,4	0,6	3	1	18	2015
15	20,4	0,6	3	1	19	2015
17,3	31,3	0,6	4	1	18	2012
15,9	24,3	0,6	6	1	17	2008
15,9	24,3	0,6	4	1	19	2012
14,8	19,6	0,6	5	1	18	2007
14,8	19,6	0,6	3	1	21	2008
15,7	23,4	0,6	4	1	23	2001
15,7	23,4	0,6	4	1	26	2005
15,7	23,4	0,6	7	1	22	2007
15,7	23,4	0,6	3	1	17	2008
15,7	23,4	0,6	5	1	19	2012
15,7	23,4	0,6	4	1	19	2012
15,7	23,4	0,6	4	1	18	2015
12,6	12,1	0,6	3	1	22	2005
14	16,6	0,6	2	1	19	2002

14	16,6	0,6	5	1	20	2007
14	16,6	0,6	3	1	21	2008
14	16,6	0,6	3	1	16	2015
13,1	13,6	0,6	3	1	21	2006
13,1	13,6	0,6	2	1	26	2009
13,1	13,6	0,6	2	1	17	2014
16,3	26,2	0,6	4	1	22	2001
16,3	26,2	0,6	8	1	20	2013
18,8	40,2	0,6	6	1	18	2015
13,8	15,9	0,6	3	1	26	2003
13,8	15,9	0,6	5	1	25	2007
13,8	15,9	0,6	4	1	20	2013
11,2	8,5	0,6	2	1	25	2004
15,4	22,1	0,6	3	1	18	2015
16,8	28,7	0,6	4	1	20	2000
16,8	28,7	0,6	3	1	18	2002
16,8	28,7	0,6	4	1	24	2006
14,3	17,7	0,6	2	1	19	2001
14,3	17,7	0,6	3	1	18	2002

14,3	17,7	0,6	7	1	19	2009
14,3	17,7	0,6	3	1	23	2010
14,3	17,7	0,6	5	1	23	2010
14,3	17,7	0,6	3	1	23	2010
14,3	17,7	0,6	5	1	23	2010
14,3	17,7	0,6	3	1	19	2011
17,6	33	0,6	5	1	20	2010
17,6	33	0,6	5	1	20	2010
12,7	12,4	0,6	3	1	21	2005
12,7	12,4	0,6	2	1	20	2009
12,7	12,4	0,6	2	1	23	2009
13	13,3	0,6	2	1	24	2001
13	13,3	0,6	2	1	23	2007
13	13,3	0,6	2	1	22	2009
13	13,3	0,6	2	1	26	2009
13	13,3	0,6	3	1	21	2011
13	13,3	0,6	2	1	17	2014
13	13,3	0,6	2	1	18	2014
13	13,3	0,6	3	1	19	2014

16,7	28,2	0,6	5	1	15	2015
17,4	31,9	0,6	5	1	18	2000
16,6	27,7	0,6	6	1	16	2015
12,9	13	0,6	2	1	17	2014
13,5	14,9	0,6	3	1	21	2000
13,5	14,9	0,6	2	1	20	2002
13,5	14,9	0,6	3	1	23	2004
13,5	14,9	0,6	2	1	21	2005
13,5	14,9	0,6	2	1	18	2008
11,7	9,7	0,6	2	1	25	2004
12,2	11	0,6	2	1	19	2006
15,6	23	0,6	4	1	15	2015
15,3	21,7	0,6	4	1	19	2001
15,3	21,7	0,6	3	1	20	2007
15,8	23,9	0,6	5	1	20	1999
15,8	23,9	0,6	6	1	17	2008
14,4	18,1	0,6	3	1	21	2003
14,4	18,1	0,6	2	1	24	2009
14,4	18,1	0,6	4	1	24	2009

16,1	25,3	0,6	3	1	22	2003
17,8	34,2	0,6	9	1	24	2006
14,1	17	0,6	2	1	18	2000
14,1	17	0,6	5	1	20	2000
14,1	17	0,6	3	1	21	2011
14,1	17	0,6	3	1	15	2015
14,1	17	0,6	3	1	23	2015
15,2	21,3	0,6	3	1	19	2000
15,2	21,3	0,6	3	1	25	2003
15,2	21,3	0,6	3	1	21	2010
15,2	21,3	0,6	3	1	21	2010
17	29,8	0,6	4	1	19	2015
13,7	15,6	0,6	2	1	22	1999
13,4	14,6	0,6	2	1	20	2000
14,5	18,5	0,6	3	1	22	2001
14,5	18,5	0,6	2	1	24	2001
14,5	18,5	0,6	4	1	19	2009
14,5	18,5	0,6	4	1	21	2009
14,5	18,5	0,6	3	1	18	2014

15,5	22,6	0,6	5	1	23	2010
15,5	22,6	0,6	5	1	23	2010
13,9	16,3	0,6	3	1	24	2001
13,9	16,3	0,6	3	1	20	2008
15,9	24,4	0,6	4	1	20	2001
15,1	20,9	0,6	5	1	21	1999
15,1	20,9	0,6	4	1	22	2007
15,1	20,9	0,6	6	1	25	2007
15,1	20,9	0,6	3	1	20	2012
15,7	23,5	0,6	5	1	24	2001
15,7	23,5	0,6	10	1	18	2015
16,3	26,3	0,6	6	1	23	2003
14,6	18,9	0,6	4	1	26	1999
14,6	18,9	0,6	3	1	17	2008
14,6	18,9	0,6	4	1	19	2012
16,8	28,8	0,6	6	1	19	2013
15	20,5	0,6	4	1	18	2000
15	20,5	0,6	4	1	26	2003
15	20,5	0,6	6	1	23	2005

15	20,5	0,6	5	1	19	2006
15	20,5	0,6	3	1	19	2008
15	20,5	0,6	4	1	21	2011
15	20,5	0,6	4	1	19	2011
17,4	32	0,6	6	1	21	2015
11,4	9	0,6	2	1	20	2004
11,4	9	0,6	2	1	25	2004
20	48,6	0,6	5	1	15	2015
14,7	19,3	0,6	3	1	19	2000
14,7	19,3	0,6	3	1	19	2001
14,7	19,3	0,6	3	1	21	2010
14,7	19,3	0,6	3	1	21	2010
14,7	19,3	0,6	4	1	15	2015
14,9	20,1	0,6	3	1	21	2000
14,9	20,1	0,6	5	1	19	2013
12	10,5	0,6	2	1	26	2006
12	10,5	0,6	2	1	20	2007
12	10,5	0,6	2	1	19	2009
14,8	19,7	0,6	3	1	26	2006

14,8	19,7	0,6	4	1	23	2008
14,8	19,7	0,6	2	1	22	2009
14,8	19,7	0,6	7	1	23	2009
14,2	17,4	0,6	4	1	21	2006
14,2	17,4	0,6	3	1	19	2014
17,7	33,7	0,6	4	1	19	2012
13,3	14,3	0,6	2	1	23	2003
13,3	14,3	0,6	2	1	25	2003
15,4	22,2	0,6	4	1	22	1999
16	24,9	0,6	2	1	19	2013
13,6	15,3	0,6	3	1	22	1999
13,6	15,3	0,6	3	1	26	2003
17,3	31,5	0,6	6	1	24	2001
12,4	11,6	0,6	3	1	21	2005
12,4	11,6	0,6	2	1	19	2006
15,6	23,1	0,6	4	1	17	2002
15,6	23,1	0,6	5	1	21	2003
15,8	24	0,6	3	1	20	2003
15,8	24	0,6	5	1	19	2012

17,9	34,9	0,6	4	1	19	2013
17	29,9	0,6	5	1	20	2013
14	16,7	0,6	4	1	23	1999
14	16,7	0,6	2	1	19	2000
14	16,7	0,6	3	1	25	2003
14	16,7	0,6	3	1	19	2005
14	16,7	0,6	5	1	18	2007
14	16,7	0,6	3	1	19	2008
14	16,7	0,6	3	1	21	2008
14	16,7	0,6	4	1	20	2011
14	16,7	0,6	2	1	19	2012
14	16,7	0,6	3	1	18	2015
11,6	9,5	0,6	2	1	20	2007
16,1	25,4	0,6	3	1	17	2014
15,3	21,8	0,6	4	1	21	1999
15,3	21,8	0,6	3	1	18	2000
15,3	21,8	0,6	3	1	19	2002
15,3	21,8	0,6	3	1	22	2003
15,3	21,8	0,6	4	1	20	2009

13,2	14	0,6	5	1	23	2007
13,2	14	0,6	2	1	20	2009
13,2	14	0,6	2	1	24	2009
13,2	14	0,6	2	1	24	2009
14,3	17,8	0,6	5	1	27	1999
14,3	17,8	0,6	2	1	18	2000
14,3	17,8	0,6	2	1	23	2001
14,3	17,8	0,6	3	1	22	2003
14,3	17,8	0,6	4	1	23	2009
13,8	16	0,6	3	1	18	2002
13,8	16	0,6	3	1	26	2003
16,2	25,9	0,6	5	1	19	2012
12,5	11,9	0,6	2	1	19	2014
17,4	32,1	0,6	10	1	22	2015
15,2	21,4	0,6	3	1	24	2002
15,2	21,4	0,6	3	1	21	2003
15,2	21,4	0,6	5	1	22	2005
13,1	13,7	0,6	2	1	21	2000
13,1	13,7	0,6	2	1	21	2006

13,1	13,7	0,6	2	1	24	2006
13,1	13,7	0,6	3	1	26	2006
13,1	13,7	0,6	3	1	17	2008
13,1	13,7	0,6	3	1	20	2008
13,1	13,7	0,6	2	1	19	2013
13,1	13,7	0,6	2	1	19	2013
13,1	13,7	0,6	2	1	20	2013
16,8	28,9	0,6	5	1	20	2000
16,8	28,9	0,6	6	1	17	2002
16,8	28,9	0,6	4	1	22	2015
15,9	24,5	0,6	6	1	19	2007
14,4	18,2	0,6	6	1	25	1999
14,4	18,2	0,6	3	1	18	2000
14,4	18,2	0,6	3	1	20	2000
14,4	18,2	0,6	2	1	21	2003
14,4	18,2	0,6	3	1	26	2003
17,7	33,8	0,6	4	1	15	2015
15,5	22,7	0,6	4	1	19	1999
15,5	22,7	0,6	5	1	20	2000

16,3	26,4	0,6	6	1	20	2013
12,1	10,8	0,6	2	1	23	2006
12,1	10,8	0,6	2	1	26	2006
18,5	38,6	0,6	6	1	20	2013
13,5	15	0,6	2	1	19	2000
13,5	15	0,6	3	1	26	2001
13,5	15	0,6	2	1	20	2002
13,5	15	0,6	3	1	25	2005
13,5	15	0,6	4	1	23	2009
15,7	23,6	0,6	4	1	18	2002
15,7	23,6	0,6	6	1	22	2003
15,7	23,6	0,6	5	1	22	2004
15,7	23,6	0,6	3	1	19	2012
11,3	8,8	0,6	2	1	23	2004
12,6	12,2	0,6	2	1	23	2010
12,6	12,2	0,6	2	1	23	2010
13	13,4	0,6	2	1	20	2002
13	13,4	0,6	3	1	21	2005
13	13,4	0,6	2	1	19	2006

13	13,4	0,6	2	1	24	2006
13	13,4	0,6	2	1	26	2006
13	13,4	0,6	3	1	20	2007
15,1	21	0,6	3	1	25	2002
15,1	21	0,6	5	1	19	2009
15,1	21	0,6	4	1	22	2015
19,1	42,5	0,6	7	1	17	2012
16,5	27,4	0,6	4	1	18	2002
16,5	27,4	0,6	5	1	19	2012
14,1	17,1	0,6	3	1	22	2005
14,1	17,1	0,6	4	1	24	2006
14,1	17,1	0,6	3	1	23	2010
14,1	17,1	0,6	3	1	23	2010
14,1	17,1	0,6	4	1	20	2011
14,1	17,1	0,6	4	1	21	2014
14,1	17,1	0,6	4	1	23	2014
14,5	18,6	0,6	3	1	19	2000
14,5	18,6	0,6	3	1	19	2002
14,5	18,6	0,6	4	1	21	2005

14,5	18,6	0,6	5	1	19	2007
14,5	18,6	0,6	3	1	15	2015
14,5	18,6	0,6	3	1	21	2015
12,7	12,5	0,6	2	1	20	2002
12,7	12,5	0,6	2	1	23	2010
12,7	12,5	0,6	2	1	23	2010
12,7	12,5	0,6	2	1	19	2013
12,9	13,1	0,6	2	1	21	2011
12,8	12,8	0,6	2	1	19	2000
12,8	12,8	0,6	3	1	20	2004
12,8	12,8	0,6	2	1	20	2009
12,8	12,8	0,6	2	1	19	2013
12,8	12,8	0,6	3	1	21	2014
16	25	0,6	5	1	21	1999
16	25	0,6	4	1	25	2004
16	25	0,6	5	1	21	2005
16	25	0,6	4	1	19	2012
19,2	43,2	0,6	7	1	18	2012
16	25	0,6	5	1	20	2013

15	20,6	0,6	4	1	23	1999
15	20,6	0,6	4	1	23	2007
15	20,6	0,6	4	1	25	2007
15	20,6	0,6	3	1	16	2008
15	20,6	0,6	4	1	19	2009
15	20,6	0,6	3	1	18	2014
14,6	19	0,6	8	1	22	2001
14,6	19	0,6	4	1	26	2009
14,6	19	0,6	4	1	19	2013
13,7	15,7	0,6	2	1	21	2000
13,7	15,7	0,6	2	1	21	2003
13,7	15,7	0,6	3	1	22	2007
15,4	22,3	0,6	3	1	17	2014
17	30	0,6	5	1	19	2013
13,9	16,4	0,6	2	1	19	1999
14,7	19,4	0,6	3	1	26	2001
14,7	19,4	0,6	3	1	22	2002
14,7	19,4	0,6	4	1	26	2003
14,8	19,8	0,6	2	1	21	2000

14,8	19,8	0,6	5	1	20	2010
14,8	19,8	0,6	5	1	20	2010
13,4	14,7	0,6	2	1	26	2003
13,4	14,7	0,6	2	1	20	2011
15,8	24,1	0,6	3	1	19	2007
16,1	25,5	0,6	3	1	21	2001
15,6	23,2	0,6	4	1	20	2013
14,2	17,5	0,6	2	1	17	2002
14,2	17,5	0,6	3	1	21	2005
14,2	17,5	0,6	3	1	22	2011
14,2	17,5	0,6	6	1	19	2014
17,2	31,1	0,6	5	1	18	2000
11,9	10,3	0,6	2	1	19	2007
12,2	11,1	0,6	2	1	19	2014
17,7	33,9	0,6	5	1	15	2015
15,3	21,9	0,6	5	1	18	2000
15,9	24,6	0,6	3	1	21	2001
13,3	14,4	0,6	2	1	20	2002
16,4	27	0,6	6	1	21	2010

16,4	27	0,6	6	1	21	2010
11,2	8,6	0,6	1	1	23	2005
14,3	17,9	0,6	2	1	24	2001
14,3	17,9	0,6	3	1	24	2001
18	35,7	0,6	6	1	19	2013
16,5	27,5	0,6	7	1	22	2001
16,5	27,5	0,6	3	1	21	2015
13,6	15,4	0,6	3	1	19	2013
15,2	21,5	0,6	5	1	19	2007
15,2	21,5	0,6	3	1	16	2008
15,2	21,5	0,6	4	1	24	2009
15,2	21,5	0,6	5	1	17	2014
17,3	31,7	0,6	5	1	19	2000
14	16,8	0,6	4	1	22	2004
14	16,8	0,6	4	1	20	2006
14	16,8	0,6	2	1	21	2006
14	16,8	0,6	3	1	20	2007
14	16,8	0,6	3	1	20	2008
14	16,8	0,6	4	1	20	2009

14	16,8	0,6	6	1	22	2011
14	16,8	0,6	4	1	17	2014
14	16,8	0,6	2	1	15	2015
15,5	22,8	0,6	3	1	24	2001
15,5	22,8	0,6	3	1	21	2003
15,5	22,8	0,6	4	1	23	2006
15,7	23,7	0,6	5	1	26	2000
15,7	23,7	0,6	3	1	18	2002
13,8	16,1	0,6	3	1	25	2003
13,8	16,1	0,6	7	1	21	2009
16	25,1	0,6	5	1	20	2001
16	25,1	0,6	10	1	23	2002
15,1	21,1	0,6	4	1	22	2005
15,1	21,1	0,6	5	1	24	2006
15,1	21,1	0,6	3	1	19	2013
15,1	21,1	0,6	3	1	18	2015
14,4	18,3	0,6	2	1	26	2003
14,4	18,3	0,6	4	1	24	2009
13,2	14,1	0,6	2	1	26	1999

13,2	14,1	0,6	3	1	19	2007
13,2	14,1	0,6	3	1	23	2009
13,2	14,1	0,6	2	1	26	2009
13,2	14,1	0,6	3	1	21	2011
13,2	14,1	0,6	2	1	20	2012
13,2	14,1	0,6	2	1	19	2013
17,4	32,3	0,6	7	1	24	2001
17,7	34	0,6	6	1	16	2015
17,2	31,2	0,6	6	1	17	2002
10,5	7,1	0,6	2	1	23	2004
15	20,7	0,6	6	1	23	2011
15	20,7	0,6	4	1	20	2013
15	20,7	0,6	4	1	18	2015
14,5	18,7	0,6	2	1	24	2001
14,5	18,7	0,6	3	1	20	2003
14,5	18,7	0,6	4	1	20	2004
14,5	18,7	0,6	5	1	26	2006
14,5	18,7	0,6	3	1	19	2010
14,5	18,7	0,6	3	1	19	2010

14,5	18,7	0,6	4	1	17	2012
14,5	18,7	0,6	3	1	16	2015
12	10,6	0,6	2	1	18	2007
16,1	25,6	0,6	4	1	18	1999
17,8	34,6	0,6	5	1	21	2000
14,1	17,2	0,6	3	1	26	2003
14,1	17,2	0,6	3	1	20	2007
14,1	17,2	0,6	3	1	19	2008
21,3	59,3	0,6	9	1	17	2014
12,4	11,7	0,6	2	1	22	2011
14,9	20,3	0,6	3	1	16	2008
14,9	20,3	0,6	5	1	19	2012
14,9	20,3	0,6	4	1	19	2013
14,6	19,1	0,6	3	1	18	2014
13,5	15,1	0,6	2	1	21	2000
13,5	15,1	0,6	2	1	23	2010
13,5	15,1	0,6	2	1	23	2010
13,5	15,1	0,6	2	1	16	2015
18,2	37	0,6	4	1	19	2012

13,1	13,8	0,6	2	1	26	1999
13,1	13,8	0,6	2	1	22	2001
13,1	13,8	0,6	5	1	18	2007
13,1	13,8	0,6	3	1	19	2007
13,1	13,8	0,6	2	1	22	2009
13,1	13,8	0,6	2	1	26	2009
13,1	13,8	0,6	2	1	19	2010
13,1	13,8	0,6	2	1	19	2010
14,8	19,9	0,6	3	1	21	2015
14,7	19,5	0,6	4	1	21	2009
14,7	19,5	0,6	5	1	21	2010
14,7	19,5	0,6	5	1	21	2010
16,2	26,1	0,6	4	1	19	2010
16,2	26,1	0,6	4	1	19	2010
17,1	30,7	0,6	4	1	17	2002
16,3	26,6	0,6	6	1	22	2004
16,3	26,6	0,6	8	1	20	2013
16,3	26,6	0,6	5	1	19	2014
15,3	22	0,6	4	1	21	2013

15,3	22	0,6	3	1	15	2015
16,6	28,1	0,6	10	1	25	2004
13,9	16,5	0,6	5	1	26	2006
13,9	16,5	0,6	3	1	16	2015
12,5	12	0,6	1	1	26	2001
12,5	12	0,6	3	1	22	2005
16,5	27,6	0,6	4	1	22	2001
16,5	27,6	0,6	6	1	19	2013
13,7	15,8	0,6	2	1	26	1999
13,7	15,8	0,6	2	1	26	2001
13,7	15,8	0,6	3	1	19	2015
13	13,5	0,6	2	1	25	1999
13	13,5	0,6	2	1	21	2001
13	13,5	0,6	2	1	20	2002
13	13,5	0,6	3	1	22	2004
13	13,5	0,6	3	1	23	2005
13	13,5	0,6	2	1	24	2006
13	13,5	0,6	3	1	18	2007
13	13,5	0,6	2	1	20	2009

13	13,5	0,6	3	1	21	2011
13	13,5	0,6	2	1	20	2013
13	13,5	0,6	2	1	20	2013
13	13,5	0,6	2	1	21	2014
21,7	62,8	0,6	10	1	17	2012
14,2	17,6	0,6	2	1	24	2001
14,2	17,6	0,6	4	1	22	2011
14,2	17,6	0,6	4	1	22	2011
14,2	17,6	0,6	3	1	19	2014
14,2	17,6	0,6	3	1	15	2015
17	30,2	0,6	7	1	17	2012
11,8	10,1	0,6	2	1	22	2007
12,6	12,3	0,6	2	1	20	2002
12,6	12,3	0,6	2	1	26	2009
12,6	12,3	0,6	2	1	17	2014
12,9	13,2	0,6	3	1	21	2005
15,5	22,9	0,6	4	1	22	2004
15,7	23,8	0,6	4	1	18	1999
15,2	21,6	0,6	4	1	19	2015

13,4	14,8	0,6	3	1	23	2001
13,4	14,8	0,6	2	1	19	2006
13,4	14,8	0,6	4	1	24	2006
17,2	31,3	0,6	5	1	23	2003
12,7	12,6	0,6	2	1	18	2002
12,7	12,6	0,6	2	1	21	2009
12,8	12,9	0,6	2	1	20	2013
12,8	12,9	0,6	2	1	21	2014
16	25,2	0,6	5	1	19	2004
12,1	10,9	0,6	2	1	19	2007
12,1	10,9	0,6	2	1	23	2010
12,1	10,9	0,6	2	1	23	2010
14,3	18	0,6	4	1	22	2011
14,3	18	0,6	4	1	18	2015
17,5	33	0,6	4	1	19	2013
15,1	21,2	0,6	6	1	17	2008
15,1	21,2	0,6	5	1	19	2012
16,1	25,7	0,6	5	1	16	2008
16,1	25,7	0,6	5	1	20	2013

14	16,9	0,6	4	1	19	1999
14	16,9	0,6	2	1	21	2000
14	16,9	0,6	3	1	21	2008
14	16,9	0,6	3	1	22	2015
15,4	22,5	0,6	3	1	20	2006
15,4	22,5	0,6	5	1	21	2013
15,4	22,5	0,6	3	1	18	2015
15,8	24,3	0,6	4	1	24	2001
15,8	24,3	0,6	4	1	18	2014
17,3	31,9	0,6	5	1	21	2010
17,3	31,9	0,6	5	1	21	2010
17,3	31,9	0,6	7	1	19	2014
13,6	15,5	0,6	3	1	24	2001
13,6	15,5	0,6	6	1	22	2005
13,6	15,5	0,6	3	1	25	2008
13,6	15,5	0,6	2	1	23	2009
13,6	15,5	0,6	3	1	18	2015
14,4	18,4	0,6	3	1	26	2001
14,4	18,4	0,6	3	1	26	2003

14,4	18,4	0,6	2	1	23	2008
14,4	18,4	0,6	2	1	22	2013
14,4	18,4	0,6	3	1	18	2015
14,4	18,4	0,6	3	1	18	2015
16,7	28,7	0,6	4	1	19	2001
16,7	28,7	0,6	7	1	22	2001
16,2	26,2	0,6	5	1	22	2005
16,2	26,2	0,6	6	1	22	2015
15	20,8	0,6	5	1	18	1999
15	20,8	0,6	4	1	19	1999
15	20,8	0,6	5	1	23	1999
15	20,8	0,6	3	1	21	2001
15	20,8	0,6	4	1	22	2009
15	20,8	0,6	3	1	19	2012
13,3	14,5	0,6	2	1	22	2000
13,3	14,5	0,6	2	1	26	2009
13,3	14,5	0,6	3	1	20	2011
13,3	14,5	0,6	9	1	21	2011
13,3	14,5	0,6	2	1	18	2012

13,3	14,5	0,6	4	1	21	2013
13,3	14,5	0,6	2	1	18	2014
15,6	23,4	0,6	4	1	21	1999
15,6	23,4	0,6	5	1	19	2014
13,8	16,2	0,6	5	1	19	1999
13,8	16,2	0,6	3	1	21	2000
13,8	16,2	0,6	3	1	25	2003
13,8	16,2	0,6	3	1	18	2008
13,8	16,2	0,6	3	1	19	2013
16,3	26,7	0,6	5	1	23	2011
14,5	18,8	0,6	4	1	20	1999
14,5	18,8	0,6	3	1	18	2015
14,9	20,4	0,6	5	1	19	2012
12,2	11,2	0,6	1	1	26	2003
12,2	11,2	0,6	2	1	18	2007
12,2	11,2	0,6	2	1	22	2009
12,2	11,2	0,6	2	1	19	2011
14,6	19,2	0,6	3	1	22	2001
14,6	19,2	0,6	3	1	17	2002

14,6	19,2	0,6	3	1	20	2003
14,6	19,2	0,6	7	1	21	2009
14,8	20	0,6	3	1	23	2006
14,8	20	0,6	3	1	20	2012
14,7	19,6	0,6	3	1	26	2003
15,3	22,1	0,6	4	1	23	1999
15,3	22,1	0,6	5	1	22	2007
15,3	22,1	0,6	3	1	20	2011
14,1	17,3	0,6	2	1	25	2003
14,1	17,3	0,6	4	1	21	2006
14,1	17,3	0,6	3	1	17	2008
14,1	17,3	0,6	2	1	19	2013
18	36	0,6	8	1	15	2015
13,2	14,2	0,6	3	1	19	2005
13,2	14,2	0,6	2	1	19	2008
13,2	14,2	0,6	3	1	23	2009
13,2	14,2	0,6	2	1	19	2013
13,2	14,2	0,6	2	1	19	2013
13,2	14,2	0,6	2	1	17	2014

13,2	14,2	0,6	2	1	18	2014
13,2	14,2	0,6	3	1	18	2014
20,2	50,9	0,6	5	1	18	2012
15,7	23,9	0,6	3	1	19	2001
19,3	44,4	0,6	8	1	21	2013
15,5	23	0,6	5	1	22	2004
16	25,3	0,6	5	1	20	1999
16	25,3	0,6	4	1	17	2002
16	25,3	0,6	4	1	22	2011
13,5	15,2	0,6	2	1	22	2000
13,5	15,2	0,6	2	1	18	2002
13,5	15,2	0,6	3	1	25	2007
13,5	15,2	0,6	3	1	23	2010
13,5	15,2	0,6	3	1	23	2010
13,5	15,2	0,6	2	1	20	2012
18,7	40,4	0,6	4	1	18	2012
15,2	21,7	0,6	4	1	18	1999
15,2	21,7	0,6	4	1	20	2000
15,2	21,7	0,6	6	1	24	2001

15,2	21,7	0,6	3	1	17	2002
15,2	21,7	0,6	4	1	23	2003
15,2	21,7	0,6	4	1	17	2009
15,2	21,7	0,6	9	1	22	2009
19,9	48,7	0,6	7	1	18	2012
12,3	11,5	0,6	2	1	26	2006
11,7	9,9	0,6	1	1	26	2001
17,6	33,7	0,6	6	1	24	2006
14,2	17,7	0,6	4	1	21	2006
14,2	17,7	0,6	3	1	18	2014
14,2	17,7	0,6	3	1	18	2014
14,2	17,7	0,6	3	1	22	2015
16,1	25,8	0,6	5	1	21	2000
16,1	25,8	0,6	5	1	19	2013
13,1	13,9	0,6	2	1	24	2006
13,1	13,9	0,6	5	1	23	2010
13,1	13,9	0,6	5	1	23	2010
13,7	15,9	0,6	2	1	22	2001
13,7	15,9	0,6	4	1	25	2005

13,7	15,9	0,6	3	1	19	2008
13,7	15,9	0,6	3	1	20	2011
16,7	28,8	0,6	4	1	23	2002
16,2	26,3	0,6	3	1	22	2003
16,2	26,3	0,6	5	1	18	2012
15,8	24,4	0,6	4	1	21	2015
15,1	21,3	0,6	4	1	18	2004
16,6	28,3	0,6	4	1	15	2015
17	30,4	0,6	5	1	21	2010
17	30,4	0,6	5	1	21	2010
15,4	22,6	0,6	4	1	20	2013
16,3	26,8	0,6	5	1	20	2003
16,3	26,8	0,6	4	1	19	2015
16,5	27,8	0,6	4	1	19	2002
14,3	18,1	0,6	3	1	21	2000
14,3	18,1	0,6	3	1	19	2010
14,3	18,1	0,6	3	1	19	2010
13	13,6	0,6	3	1	21	2005
13	13,6	0,6	2	1	20	2010

13	13,6	0,6	2	1	23	2010
13	13,6	0,6	2	1	20	2010
13	13,6	0,6	2	1	23	2010
13	13,6	0,6	2	1	19	2013
13	13,6	0,6	2	1	19	2014
17,2	31,5	0,6	4	1	23	2002
12	10,7	0,6	1	1	26	2001
12	10,7	0,6	2	1	19	2007
12	10,7	0,6	2	1	23	2007
12	10,7	0,6	2	1	23	2009
13,4	14,9	0,6	3	1	20	2000
13,4	14,9	0,6	2	1	19	2002
13,4	14,9	0,6	3	1	21	2005
13,4	14,9	0,6	3	1	19	2014
15	20,9	0,6	6	1	21	1999
15	20,9	0,6	7	1	19	2009
15,9	24,9	0,6	5	1	18	1999
12,5	12,1	0,6	2	1	26	2009
12,5	12,1	0,6	2	1	20	2013

14	17	0,6	2	1	22	2001
14	17	0,6	4	1	19	2007
14	17	0,6	5	1	25	2007
14	17	0,6	4	1	24	2009
14	17	0,6	4	1	20	2011
14	17	0,6	2	1	17	2012
14	17	0,6	4	1	21	2013
14	17	0,6	3	1	18	2014
12,9	13,3	0,6	2	1	25	1999
12,9	13,3	0,6	3	1	23	2007
14,4	18,5	0,6	6	1	23	1999
14,9	20,5	0,6	4	1	18	1999
14,9	20,5	0,6	3	1	23	2003
21	57,4	0,6	8	1	19	2013
15,3	22,2	0,6	3	1	21	2000
15,3	22,2	0,6	3	1	23	2002
15,3	22,2	0,6	4	1	21	2003
12,6	12,4	0,6	3	1	19	2008
12,6	12,4	0,6	2	1	23	2010

12,6	12,4	0,6	2	1	23	2010
12,8	13	0,6	2	1	22	2011
12,8	13	0,6	2	1	20	2013
14,5	18,9	0,6	6	1	20	1999
14,5	18,9	0,6	3	1	20	2000
12,7	12,7	0,6	2	1	19	2009
12,7	12,7	0,6	2	1	23	2009
12,7	12,7	0,6	2	1	19	2013
14,8	20,1	0,6	3	1	20	2003
20,8	55,8	0,6	10	1	18	2012
16	25,4	0,6	5	1	21	2014
14,6	19,3	0,6	3	1	18	2000
14,6	19,3	0,6	4	1	21	2009
14,6	19,3	0,6	5	1	26	2009
13,6	15,6	0,6	3	1	25	2003
15,7	24	0,6	5	1	20	1999
15,7	24	0,6	4	1	20	2004
14,7	19,7	0,6	3	1	22	2004
14,7	19,7	0,6	3	1	21	2015

13,8	16,3	0,6	4	1	26	1999
13,8	16,3	0,6	2	1	24	2006
13,8	16,3	0,6	4	1	24	2009
15,5	23,1	0,6	5	1	18	1999
15,5	23,1	0,6	3	1	21	2001
15,5	23,1	0,6	4	1	15	2015
16,7	28,9	0,6	6	1	20	2011
13,3	14,6	0,6	2	1	23	2002
13,3	14,6	0,6	3	1	18	2008
13,3	14,6	0,6	2	1	20	2008
13,3	14,6	0,6	3	1	21	2015
14,1	17,4	0,6	3	1	23	1999
15,2	21,8	0,6	4	1	20	2001
15,2	21,8	0,6	5	1	22	2005
15,2	21,8	0,6	5	1	26	2009
15,2	21,8	0,6	7	1	19	2012
15,2	21,8	0,6	4	1	21	2012
15,2	21,8	0,6	3	1	17	2014
11,8	10,2	0,6	1	1	25	1999

12,1	11	0,6	2	1	22	2007
16,2	26,4	0,6	5	1	22	2003
16,2	26,4	0,6	5	1	26	2005
16,2	26,4	0,6	7	1	21	2014
17,2	31,6	0,6	7	1	21	2012
16,5	27,9	0,6	3	1	18	2014
16,5	27,9	0,6	3	1	15	2015
16,3	26,9	0,6	5	1	18	1999
16,3	26,9	0,6	7	1	26	2003
16,3	26,9	0,6	4	1	17	2008
16,3	26,9	0,6	6	1	20	2011
16,3	26,9	0,6	4	1	18	2014
15,8	24,5	0,6	5	1	18	1999
15,8	24,5	0,6	4	1	22	1999
15,8	24,5	0,6	5	1	19	2012
16,4	27,4	0,6	3	1	20	2003
16,4	27,4	0,6	6	1	21	2003
16,4	27,4	0,6	6	1	25	2004
17,5	33,3	0,6	6	1	19	2011

11,1	8,5	0,6	1	1	22	2007
16,9	30	0,6	7	1	17	2014
15,1	21,4	0,6	7	1	20	2007
15,1	21,4	0,6	6	1	23	2008
15,6	23,6	0,6	4	1	20	1999
15,6	23,6	0,6	5	1	20	1999
15,6	23,6	0,6	5	1	22	1999
15,6	23,6	0,6	3	1	19	2012
14,2	17,8	0,6	2	1	24	2001
14,2	17,8	0,6	3	1	25	2003
14,2	17,8	0,6	4	1	21	2006
14,2	17,8	0,6	4	1	26	2006
14,2	17,8	0,6	3	1	21	2008
14,2	17,8	0,6	7	1	21	2009
14,2	17,8	0,6	2	1	17	2014
14,2	17,8	0,6	5	1	21	2014
13,2	14,3	0,6	2	1	22	1999
13,2	14,3	0,6	3	1	21	2000
13,2	14,3	0,6	3	1	20	2002

13,2	14,3	0,6	2	1	23	2002
13,2	14,3	0,6	2	1	18	2008
13,2	14,3	0,6	3	1	19	2008
13,2	14,3	0,6	4	1	23	2009
13,2	14,3	0,6	3	1	17	2014
13,2	14,3	0,6	3	1	21	2014
13,2	14,3	0,6	3	1	15	2015
13,9	16,7	0,6	3	1	24	2001
13,9	16,7	0,6	3	1	24	2006
13,5	15,3	0,6	3	1	19	2006
13,5	15,3	0,6	2	1	21	2013
17,3	32,2	0,6	3	1	19	2015
17,1	31,1	0,6	5	1	19	2013
15	21	0,6	5	1	22	1999
15	21	0,6	3	1	19	2000
15	21	0,6	4	1	24	2001
15	21	0,6	3	1	23	2003
15	21	0,6	4	1	22	2004
15	21	0,6	6	1	23	2006

15	21	0,6	3	1	20	2008
15	21	0,6	5	1	24	2009
15	21	0,6	4	1	20	2013
13,7	16	0,6	2	1	20	2000
13,7	16	0,6	5	1	19	2000
13,7	16	0,6	3	1	21	2003
13,7	16	0,6	3	1	22	2004
10,4	7	0,6	1	1	23	2009
12,2	11,3	0,6	2	1	25	2003
20	49,8	0,6	10	1	19	2013
16	25,5	0,6	5	1	20	2000
16	25,5	0,6	6	1	19	2007
16	25,5	0,6	4	1	16	2008
17,4	32,8	0,6	5	1	19	2013
15,3	22,3	0,6	5	1	23	2006
15,3	22,3	0,6	5	1	18	2007
14,9	20,6	0,6	3	1	19	2014
13,1	14	0,6	2	1	20	2002
13,1	14	0,6	3	1	23	2005

13,1	14	0,6	2	1	24	2009
13,1	14	0,6	2	1	17	2014
13,1	14	0,6	3	1	19	2014
13,1	14	0,6	2	1	19	2014
15,7	24,1	0,6	6	1	22	1999
15,7	24,1	0,6	4	1	24	2001
15,7	24,1	0,6	6	1	20	2007
15,7	24,1	0,6	6	1	19	2015
17	30,6	0,6	4	1	21	2015
15,5	23,2	0,6	7	1	21	2009
16,1	26	0,6	5	1	18	1999
16,1	26	0,6	5	1	20	2013
19,2	44,1	0,6	5	1	19	2010
19,2	44,1	0,6	5	1	19	2010
11,9	10,5	0,6	2	1	23	2007
14,8	20,2	0,6	4	1	18	2015
14	17,1	0,6	4	1	25	2002
14	17,1	0,6	4	1	25	2004
14,5	19	0,6	3	1	26	2000

14,5	19	0,6	2	1	20	2003
14,5	19	0,6	3	1	20	2004
14,5	19	0,6	4	1	20	2006
14,5	19	0,6	3	1	19	2014
16,5	28	0,6	6	1	24	2001
14,7	19,8	0,6	5	1	21	2000
14,7	19,8	0,6	4	1	26	2006
14,7	19,8	0,6	4	1	20	2011
14,7	19,8	0,6	4	1	19	2013
12,3	11,6	0,6	2	1	19	2006
12,3	11,6	0,6	2	1	19	2006
14,6	19,4	0,6	4	1	25	1999
13,4	15	0,6	2	1	20	2000
13,4	15	0,6	3	1	25	2004
13,4	15	0,6	3	1	18	2008
13,4	15	0,6	4	1	22	2009
13,4	15	0,6	2	1	24	2009
16,3	27	0,6	4	1	19	2013
13	13,7	0,6	3	1	22	2005

13	13,7	0,6	3	1	23	2007
13	13,7	0,6	3	1	25	2008
13	13,7	0,6	2	1	19	2013
11	8,3	0,6	2	1	23	2004
15,2	21,9	0,6	3	1	18	2002
15,2	21,9	0,6	4	1	24	2006
15,2	21,9	0,6	3	1	15	2015
11,3	9	0,6	2	1	23	2004
17,3	32,3	0,6	6	1	22	2003
13,8	16,4	0,6	3	1	23	2008
12,4	11,9	0,6	2	1	22	2004
12,4	11,9	0,6	3	1	25	2004
12,4	11,9	0,6	2	1	20	2006
12,4	11,9	0,6	2	1	21	2006
13,6	15,7	0,6	2	1	23	2015
12,9	13,4	0,6	3	1	23	2005
16,8	29,6	0,6	6	1	19	2001
15,4	22,8	0,6	5	1	19	2000
15,4	22,8	0,6	3	1	19	2001

15,4	22,8	0,6	6	1	16	2015
15,6	23,7	0,6	3	1	20	2000
15,6	23,7	0,6	3	1	20	2002
14,1	17,5	0,6	2	1	22	2001
14,1	17,5	0,6	3	1	26	2003
14,1	17,5	0,6	2	1	16	2008
14,1	17,5	0,6	7	1	19	2009
15,9	25,1	0,6	4	1	19	2013
15,9	25,1	0,6	4	1	22	2013
15,1	21,5	0,6	3	1	18	2000
15,1	21,5	0,6	3	1	22	2001
12,5	12,2	0,6	2	1	22	2005
12,5	12,2	0,6	2	1	23	2010
12,5	12,2	0,6	2	1	23	2010
12,8	13,1	0,6	3	1	23	2003
12,8	13,1	0,6	3	1	21	2005
12,8	13,1	0,6	2	1	24	2006
12,8	13,1	0,6	2	1	19	2014
13,3	14,7	0,6	2	1	19	2000

13,3	14,7	0,6	3	1	26	2003
13,3	14,7	0,6	2	1	22	2009
12,6	12,5	0,6	2	1	22	2002
12,6	12,5	0,6	2	1	24	2006
12,7	12,8	0,6	2	1	21	2003
12,7	12,8	0,6	4	1	20	2009
12,7	12,8	0,6	2	1	20	2009
16	25,6	0,6	5	1	19	2004
12	10,8	0,6	2	1	19	2006
12	10,8	0,6	2	1	21	2011
16	25,6	0,6	6	1	21	2013
14,2	17,9	0,6	3	1	18	2007
14,2	17,9	0,6	5	1	22	2007
14,2	17,9	0,6	3	1	20	2008
14,2	17,9	0,6	4	1	23	2008
14,2	17,9	0,6	4	1	20	2011
14,2	17,9	0,6	3	1	18	2014
14,2	17,9	0,6	3	1	19	2014
14,2	17,9	0,6	3	1	19	2015

15	21,1	0,6	7	1	19	2002
15	21,1	0,6	3	1	22	2003
15	21,1	0,6	9	1	21	2005
15	21,1	0,6	5	1	23	2007
15	21,1	0,6	3	1	19	2012
15	21,1	0,6	3	1	21	2015
16,6	28,6	0,6	3	1	19	2000
16,6	28,6	0,6	5	1	20	2010
16,6	28,6	0,6	5	1	20	2010
15,7	24,2	0,6	6	1	19	1999
15,7	24,2	0,6	3	1	24	2001
15,7	24,2	0,6	4	1	24	2002
15,3	22,4	0,6	5	1	22	2011
17,6	34,1	0,6	5	1	19	2013
13,9	16,8	0,6	3	1	21	2008
13,9	16,8	0,6	2	1	20	2013
18,1	37,1	0,6	7	1	19	2015
15,5	23,3	0,6	7	1	23	2001
15,5	23,3	0,6	6	1	25	2005

15,5	23,3	0,6	4	1	20	2013
16,4	27,6	0,6	4	1	18	2015
17,3	32,4	0,6	6	1	17	2002
14,9	20,7	0,6	4	1	20	1999
14,3	18,3	0,6	5	1	21	2000
14,3	18,3	0,6	3	1	18	2001
14,3	18,3	0,6	4	1	23	2002
14,3	18,3	0,6	3	1	21	2015
18	36,5	0,6	9	1	20	2010
18	36,5	0,6	9	1	20	2010
13,5	15,4	0,6	2	1	20	2000
13,5	15,4	0,6	3	1	20	2002
13,5	15,4	0,6	3	1	19	2014
17,1	31,3	0,6	6	1	15	2015
13,2	14,4	0,6	4	1	22	2004
13,2	14,4	0,6	2	1	24	2006
13,2	14,4	0,6	2	1	26	2009
13,2	14,4	0,6	2	1	21	2014
13,7	16,1	0,6	2	1	18	2002

13,7	16,1	0,6	3	1	19	2008
13,7	16,1	0,6	3	1	21	2011
13,7	16,1	0,6	4	1	22	2011
13,7	16,1	0,6	2	1	17	2014
14,8	20,3	0,6	5	1	23	1999
14,8	20,3	0,6	3	1	25	2007
15,8	24,7	0,6	5	1	23	2003
14,4	18,7	0,6	3	1	21	2003
20,3	52,4	0,6	7	1	17	2012
15,2	22	0,6	5	1	20	2000
15,2	22	0,6	4	1	23	2011
15,2	22	0,6	4	1	21	2012
14,7	19,9	0,6	3	1	23	2002
14,7	19,9	0,6	4	1	25	2004
14,7	19,9	0,6	6	1	25	2005
14,7	19,9	0,6	5	1	20	2013
14,5	19,1	0,6	3	1	26	2001
14,5	19,1	0,6	7	1	19	2009
14,5	19,1	0,6	5	1	20	2009

14,5	19,1	0,6	6	1	22	2009
12,1	11,1	0,6	2	1	18	2014
14,6	19,5	0,6	3	1	20	2004
14,6	19,5	0,6	6	1	20	2011
14	17,2	0,6	3	1	20	2001
14	17,2	0,6	3	1	25	2004
14	17,2	0,6	4	1	21	2005
14	17,2	0,6	3	1	18	2008
14	17,2	0,6	4	1	21	2011
14	17,2	0,6	3	1	19	2015
14	17,2	0,6	3	1	23	2015
11,8	10,3	0,6	1	1	23	2015
15,6	23,8	0,6	5	1	20	2013
15,6	23,8	0,6	6	1	18	2015
15,9	25,2	0,6	5	1	22	2013
15,4	22,9	0,6	4	1	18	2015
18,2	37,8	0,6	6	1	19	2013
13,1	14,1	0,6	2	1	24	2001
13,1	14,1	0,6	2	1	24	2006

13,1	14,1	0,6	2	1	22	2009
15,1	21,6	0,6	9	1	23	2007
16	25,7	0,6	5	1	21	1999
16	25,7	0,6	5	1	25	2004
17,3	32,5	0,6	3	1	18	2015
16,5	28,2	0,6	5	1	25	2004
16,5	28,2	0,6	4	1	18	2015
12,2	11,4	0,6	2	1	23	2003
12,2	11,4	0,6	2	1	24	2006
12,2	11,4	0,6	2	1	19	2007
13,8	16,5	0,6	2	1	22	2001
13,8	16,5	0,6	3	1	18	2015
11,6	9,8	0,6	2	1	22	2011
14,1	17,6	0,6	2	1	25	2002
14,1	17,6	0,6	4	1	25	2007
14,1	17,6	0,6	3	1	18	2014
15,7	24,3	0,6	3	1	20	2013
17,1	31,4	0,6	4	1	21	2015
13	13,8	0,6	5	1	23	1999

13	13,8	0,6	3	1	23	2005
13	13,8	0,6	5	1	25	2007
13	13,8	0,6	3	1	18	2008
13	13,8	0,6	2	1	21	2008
13	13,8	0,6	4	1	23	2009
13	13,8	0,6	2	1	26	2009
13	13,8	0,6	3	1	21	2015
15	21,2	0,6	7	1	21	1999
15	21,2	0,6	3	1	20	2000
15	21,2	0,6	4	1	20	2006
15	21,2	0,6	3	1	18	2007
15	21,2	0,6	5	1	21	2012
15	21,2	0,6	5	1	19	2012
15,3	22,5	0,6	3	1	20	2000
15,3	22,5	0,6	5	1	22	2000
15,5	23,4	0,6	3	1	18	2000
15,5	23,4	0,6	5	1	19	2002
15,5	23,4	0,6	4	1	23	2015
16,8	29,8	0,6	4	1	19	2012

14,2	18	0,6	3	1	24	2002
14,2	18	0,6	3	1	24	2002
14,2	18	0,6	6	1	23	2007
14,2	18	0,6	4	1	21	2011
14,2	18	0,6	3	1	19	2012
12,3	11,7	0,6	2	1	26	1999
12,3	11,7	0,6	3	1	22	2005
12,3	11,7	0,6	3	1	23	2005
12,3	11,7	0,6	2	1	26	2009
12,3	11,7	0,6	2	1	23	2010
12,3	11,7	0,6	2	1	23	2010
12,3	11,7	0,6	2	1	21	2014
15,8	24,8	0,6	3	1	18	1999
15,8	24,8	0,6	5	1	19	2012
14,9	20,8	0,6	3	1	22	2004
11,1	8,6	0,6	2	1	25	2004
12,9	13,5	0,6	4	1	20	2006
12,9	13,5	0,6	3	1	23	2007
12,9	13,5	0,6	2	1	20	2013

12,9	13,5	0,6	2	1	17	2014
11,9	10,6	0,6	2	1	22	2007
13,3	14,8	0,6	3	1	20	2008
13,3	14,8	0,6	3	1	21	2008
14,3	18,4	0,6	3	1	23	1999
14,3	18,4	0,6	3	1	22	2003
14,3	18,4	0,6	3	1	22	2003
14,3	18,4	0,6	3	1	18	2014
14,3	18,4	0,6	3	1	23	2015
9,2	4,9	0,6	1	1	20	2004
20,1	51,1	0,6	7	1	18	2012
13,9	16,9	0,6	4	1	22	2013
13,9	16,9	0,6	3	1	19	2014
14,8	20,4	0,6	3	1	26	2003
18	36,7	0,6	4	1	18	2012
15,2	22,1	0,6	5	1	19	1999
15,2	22,1	0,6	5	1	25	1999
15,2	22,1	0,6	5	1	19	2006
15,2	22,1	0,6	3	1	17	2008

12,4	12	0,6	3	1	26	1999
12,4	12	0,6	2	1	19	2002
12,4	12	0,6	2	1	20	2013
12,4	12	0,6	2	1	23	2015
12,8	13,2	0,6	2	1	18	2014
15,6	23,9	0,6	4	1	18	1999
16,6	28,8	0,6	5	1	20	2000
14,7	20	0,6	4	1	18	1999
14,7	20	0,6	4	1	25	2003
14,7	20	0,6	4	1	20	2009
14,7	20	0,6	3	1	15	2015
12,7	12,9	0,6	2	1	26	2000
12,7	12,9	0,6	2	1	18	2002
12,7	12,9	0,6	2	1	26	2009
12,7	12,9	0,6	2	1	19	2011
14,5	19,2	0,6	4	1	21	2011
14,6	19,6	0,6	3	1	23	2003
14,6	19,6	0,6	2	1	19	2014
16,9	30,4	0,6	5	1	22	2013

9,5	5,4	0,6	1	1	23	2005
12,6	12,6	0,6	3	1	23	2005
12,6	12,6	0,6	2	1	23	2006
12,6	12,6	0,6	2	1	18	2014
16	25,8	0,6	9	1	19	2013
17,1	31,5	0,6	9	1	22	2000
13,5	15,5	0,6	4	1	26	2000
13,5	15,5	0,6	2	1	19	2013
20	50,4	0,6	7	1	17	2014
13,7	16,2	0,6	3	1	18	2015
16,1	26,3	0,6	4	1	23	2002
16,1	26,3	0,6	4	1	19	2007
15,1	21,7	0,6	5	1	21	2001
15,1	21,7	0,6	6	1	21	2011
16,2	26,8	0,6	6	1	19	2001
16,3	27,3	0,6	4	1	17	2002
16,3	27,3	0,6	4	1	17	2014
13,2	14,5	0,6	2	1	26	2003
13,2	14,5	0,6	2	1	23	2011

13,2	14,5	0,6	2	1	17	2014
14	17,3	0,6	3	1	20	2002
14	17,3	0,6	5	1	19	2009
14	17,3	0,6	4	1	24	2009
14	17,3	0,6	4	1	24	2009
15,7	24,4	0,6	6	1	23	2011
15,7	24,4	0,6	7	1	19	2012
11,3	9,1	0,6	2	1	22	2007
12	10,9	0,6	2	1	26	2006
12	10,9	0,6	2	1	19	2011
12	10,9	0,6	2	1	19	2013
17,2	32,1	0,6	5	1	23	2002
18,4	39,3	0,6	6	1	19	2013
10,1	6,5	0,6	1	1	21	2009
17	31	0,6	6	1	18	2000
17	31	0,6	5	1	22	2013
15,3	22,6	0,6	5	1	25	2005
15,3	22,6	0,6	4	1	22	2011
15,5	23,5	0,6	4	1	27	1999

11	8,4	0,6	2	1	23	2004
15	21,3	0,6	7	1	21	1999
15	21,3	0,6	3	1	22	2003
15	21,3	0,6	3	1	20	2006
15	21,3	0,6	7	1	20	2009
15	21,3	0,6	6	1	21	2010
15	21,3	0,6	8	1	23	2010
15	21,3	0,6	6	1	21	2010
15	21,3	0,6	8	1	23	2010
11,5	9,6	0,6	2	1	23	2007
17,8	35,6	0,6	4	1	19	2012
16,7	29,4	0,6	4	1	20	2003
16,7	29,4	0,6	3	1	20	2003
16,7	29,4	0,6	10	1	23	2008
16,7	29,4	0,6	4	1	15	2015
20	50,5	0,6	6	1	19	2013
15,8	24,9	0,6	5	1	19	2012
14,1	17,7	0,6	5	1	23	2010
14,1	17,7	0,6	5	1	23	2010

14,1	17,7	0,6	3	1	18	2014
14,1	17,7	0,6	3	1	15	2015
14,1	17,7	0,6	3	1	15	2015
18,3	38,7	0,6	5	1	19	2013
17,3	32,7	0,6	7	1	22	2001
13,8	16,6	0,6	4	1	19	1999
13,8	16,6	0,6	2	1	21	2008
13,8	16,6	0,6	2	1	25	2008
13,1	14,2	0,6	4	1	23	2006
13,1	14,2	0,6	3	1	25	2007
13,1	14,2	0,6	2	1	18	2008
13,1	14,2	0,6	3	1	20	2008
13,1	14,2	0,6	2	1	21	2014
13,1	14,2	0,6	2	1	23	2014
20,1	51,3	0,6	8	1	19	2013
13,4	15,2	0,6	3	1	20	2002
16,9	30,5	0,6	10	1	21	2013
14,2	18,1	0,6	2	1	22	2000
14,2	18,1	0,6	4	1	23	2005

14,2	18,1	0,6	3	1	18	2008
14,2	18,1	0,6	3	1	20	2008
14,2	18,1	0,6	3	1	15	2015
15,2	22,2	0,6	3	1	22	2001
15,2	22,2	0,6	3	1	21	2008
15,2	22,2	0,6	3	1	16	2015
15,6	24	0,6	3	1	19	2000
12,1	11,2	0,6	3	1	23	2005
12,1	11,2	0,6	2	1	23	2007
12,1	11,2	0,6	2	1	23	2009
16,5	28,4	0,6	6	1	22	2001
16,5	28,4	0,6	6	1	20	2013
16	25,9	0,6	7	1	21	2011
16	25,9	0,6	4	1	22	2011
16	25,9	0,6	6	1	22	2013
14,8	20,5	0,6	4	1	20	2011
18,4	39,4	0,6	8	1	19	2015
15,4	23,1	0,6	5	1	22	1999
15,4	23,1	0,6	4	1	18	2000

15,4	23,1	0,6	4	1	20	2003
15,4	23,1	0,6	4	1	18	2007
15,4	23,1	0,6	4	1	16	2015
16,1	26,4	0,6	3	1	26	2001
16,1	26,4	0,6	5	1	19	2004
20,5	54,5	0,6	5	1	19	2010
20,5	54,5	0,6	5	1	19	2010
14,3	18,5	0,6	4	1	22	1999
14,3	18,5	0,6	2	1	21	2000
14,3	18,5	0,6	3	1	18	2002
14,3	18,5	0,6	3	1	19	2002
14,3	18,5	0,6	2	1	18	2012
13	13,9	0,6	2	1	20	2002
13	13,9	0,6	3	1	23	2007
13	13,9	0,6	3	1	25	2007
13	13,9	0,6	3	1	25	2007
13	13,9	0,6	3	1	20	2008
13	13,9	0,6	2	1	19	2013
13	13,9	0,6	2	1	19	2013

13	13,9	0,6	2	1	21	2014
16,3	27,4	0,6	7	1	20	1999
16,3	27,4	0,6	6	1	23	2002
16,8	30	0,6	3	1	15	2015
16,2	26,9	0,6	5	1	20	1999
16,2	26,9	0,6	4	1	18	2015
14,7	20,1	0,6	4	1	21	1999
14,7	20,1	0,6	4	1	21	2003
17,9	36,3	0,6	6	1	19	2012
14,4	18,9	0,6	4	1	19	1999
14,4	18,9	0,6	3	1	18	2014
11,8	10,4	0,6	2	1	21	2005
11,8	10,4	0,6	3	1	21	2005
14,6	19,7	0,6	2	1	19	2000
14,6	19,7	0,6	3	1	25	2003
14,6	19,7	0,6	4	1	19	2012
14,5	19,3	0,6	4	1	25	2004
13,3	14,9	0,6	2	1	20	2000
13,3	14,9	0,6	3	1	18	2014

16,7	29,5	0,6	6	1	22	2003
11,2	8,9	0,6	2	1	23	2008
12,9	13,6	0,6	3	1	21	2005
12,9	13,6	0,6	2	1	21	2006
12,9	13,6	0,6	3	1	19	2012
15,5	23,6	0,6	3	1	21	2001
15,3	22,7	0,6	4	1	22	2001
15,3	22,7	0,6	3	1	24	2001
15,3	22,7	0,6	3	1	19	2008
15,8	25	0,6	5	1	19	2001
13,7	16,3	0,6	2	1	24	2006
13,7	16,3	0,6	7	1	19	2012
13,7	16,3	0,6	3	1	21	2015
16,6	29	0,6	5	1	18	2000
18,6	40,8	0,6	5	1	21	2010
18,6	40,8	0,6	5	1	21	2010
13,5	15,6	0,6	4	1	21	2006
13,5	15,6	0,6	4	1	23	2006
13,5	15,6	0,6	3	1	26	2009

15	21,4	0,6	5	1	21	2000
15	21,4	0,6	5	1	21	2000
15	21,4	0,6	3	1	26	2001
15	21,4	0,6	4	1	19	2011
15	21,4	0,6	4	1	19	2012
15	21,4	0,6	4	1	20	2013
14	17,4	0,6	3	1	22	2000
14	17,4	0,6	2	1	26	2000
14	17,4	0,6	3	1	18	2008
14	17,4	0,6	3	1	23	2008
14	17,4	0,6	4	1	23	2009
14	17,4	0,6	3	1	15	2015
12,3	11,8	0,6	2	1	19	2013
12,8	13,3	0,6	3	1	25	2007
19,3	45,6	0,6	10+	1	17	2014
18	37	0,6	5	1	17	2014
16,5	28,5	0,6	6	1	23	2002
16,5	28,5	0,6	8	1	25	2008
11,4	9,4	0,6	2	1	23	2007

12,4	12,1	0,6	2	1	23	1999
12,4	12,1	0,6	2	1	21	2009
12,7	13	0,6	2	1	26	1999
12,7	13	0,6	2	1	19	2000
12,7	13	0,6	2	1	20	2002
12,7	13	0,6	2	1	18	2014
16	26	0,6	5	1	18	1999
13,2	14,6	0,6	3	1	23	2005
13,2	14,6	0,6	3	1	25	2007
13,2	14,6	0,6	2	1	24	2009
13,2	14,6	0,6	2	1	18	2014
15,6	24,1	0,6	6	1	20	2001
15,6	24,1	0,6	5	1	20	2001
15,6	24,1	0,6	3	1	21	2003
14,9	21	0,6	5	1	18	2007
14,9	21	0,6	3	1	19	2012
12,5	12,4	0,6	3	1	23	1999
12,5	12,4	0,6	2	1	23	2011
12,6	12,7	0,6	2	1	19	2013

11,9	10,7	0,6	1	1	26	2001
11,9	10,7	0,6	2	1	21	2006
14,1	17,8	0,6	3	1	20	2001
14,1	17,8	0,6	4	1	19	2006
14,1	17,8	0,6	4	1	19	2009
14,1	17,8	0,6	6	1	20	2011
14,1	17,8	0,6	3	1	21	2011
14,1	17,8	0,6	3	1	19	2012
14,1	17,8	0,6	3	1	15	2015
16,1	26,5	0,6	4	1	20	2013
15,2	22,3	0,6	5	1	23	1999
15,2	22,3	0,6	4	1	22	2015
18,8	42,2	0,6	4	1	19	2010
18,8	42,2	0,6	4	1	19	2010
15,4	23,2	0,6	4	1	26	1999
15,4	23,2	0,6	4	1	20	2006
15,4	23,2	0,6	3	1	19	2013
13,8	16,7	0,6	3	1	22	2005
13,8	16,7	0,6	3	1	18	2008

14,8	20,6	0,6	3	1	23	2001
14,2	18,2	0,6	4	1	18	1999
14,2	18,2	0,6	6	1	23	2008
19	43,6	0,6	4	1	17	2014
15,7	24,6	0,6	4	1	20	1999
15,7	24,6	0,6	4	1	19	2001
15,7	24,6	0,6	5	1	22	2001
15,7	24,6	0,6	4	1	23	2002
15,7	24,6	0,6	6	1	25	2004
15,7	24,6	0,6	4	1	19	2013
19,7	48,6	0,6	5	1	19	2010
19,7	48,6	0,6	5	1	19	2010
19,3	45,7	0,6	8	1	19	2013
18,1	37,7	0,6	8	1	22	2013
13,4	15,3	0,6	3	1	25	2005
13,4	15,3	0,6	2	1	19	2013
17,4	33,5	0,6	7	1	19	2014
14,7	20,2	0,6	4	1	18	1999
14,7	20,2	0,6	3	1	15	2015

13,6	16	0,6	3	1	19	2002
13,6	16	0,6	4	1	23	2005
13,6	16	0,6	2	1	21	2006
13,6	16	0,6	2	1	24	2009
13,6	16	0,6	2	1	19	2012
14,3	18,6	0,6	5	1	22	1999
14,3	18,6	0,6	4	1	26	1999
14,3	18,6	0,6	4	1	25	2003
14,3	18,6	0,6	4	1	19	2009
14,3	18,6	0,6	4	1	24	2009
14,3	18,6	0,6	6	1	23	2011
14,3	18,6	0,6	3	1	19	2014
15,1	21,9	0,6	4	1	19	2012
14,6	19,8	0,6	5	1	22	2005
14,6	19,8	0,6	7	1	23	2009
14,4	19	0,6	4	1	18	2002
14,4	19	0,6	3	1	21	2003
14,4	19	0,6	4	1	22	2003
14,4	19	0,6	3	1	22	2003

14,4	19	0,6	4	1	19	2014
14,5	19,4	0,6	3	1	24	2001
14,5	19,4	0,6	4	1	20	2006
14,5	19,4	0,6	6	1	24	2006
15,8	25,1	0,6	3	1	26	2003
15,5	23,7	0,6	4	1	22	1999
19,5	47,2	0,6	8	1	20	2013
12	11	0,6	2	1	19	2006
12	11	0,6	2	1	23	2009
15,3	22,8	0,6	5	1	22	2001
15,3	22,8	0,6	3	1	20	2003
13,9	17,1	0,6	3	1	18	2008
13,9	17,1	0,6	3	1	22	2015
11,7	10,2	0,6	2	1	23	2004
11,7	10,2	0,6	2	1	25	2004
15,9	25,6	0,6	10	1	26	2005
16,8	30,2	0,6	4	1	20	2003
15	21,5	0,6	3	1	26	2003
16	26,1	0,6	5	1	20	1999

16	26,1	0,6	3	1	19	2012
16	26,1	0,6	4	1	21	2012
16	26,1	0,6	7	1	21	2014
13	14	0,6	2	1	25	2005
13	14	0,6	2	1	26	2009
16,1	26,6	0,6	8	1	19	2002
16,1	26,6	0,6	5	1	17	2012
16,1	26,6	0,6	5	1	18	2012
16,2	27,1	0,6	4	1	25	2004
15,6	24,2	0,6	3	1	21	2001
15,6	24,2	0,6	6	1	22	2001
15,6	24,2	0,6	4	1	19	2013
18,1	37,8	0,6	7	1	18	2012
20	51	0,6	7	1	17	2012
13,3	15	0,6	2	1	19	2000
13,3	15	0,6	2	1	21	2000
13,3	15	0,6	2	1	24	2002
13,3	15	0,6	2	1	22	2013
11,3	9,2	0,6	2	1	20	2004

16,7	29,7	0,6	5	1	26	2000
16,7	29,7	0,6	7	1	19	2001
14	17,5	0,6	5	1	19	2000
14	17,5	0,6	2	1	17	2012
14	17,5	0,6	3	1	18	2014
13,7	16,4	0,6	4	1	25	1999
13,7	16,4	0,6	3	1	19	2000
13,7	16,4	0,6	3	1	25	2004
17,4	33,6	0,6	9	1	26	1999
17,4	33,6	0,6	5	1	19	2000
15,2	22,4	0,6	5	1	19	2000
15,2	22,4	0,6	3	1	25	2003
15,2	22,4	0,6	5	1	18	2007
15,2	22,4	0,6	5	1	19	2012
12,1	11,3	0,6	2	1	23	2007
14,9	21,1	0,6	3	1	24	2002
14,9	21,1	0,6	3	1	18	2007
15,4	23,3	0,6	4	1	24	2002
15,4	23,3	0,6	4	1	22	2004

15,4	23,3	0,6	5	1	21	2006
15,4	23,3	0,6	3	1	23	2007
17,1	31,9	0,6	6	1	21	2013
17,1	31,9	0,6	4	1	18	2015
18,5	40,4	0,6	4	1	15	2015
13,5	15,7	0,6	3	1	23	2001
13,5	15,7	0,6	3	1	18	2007
13,5	15,7	0,6	3	1	20	2009
13,5	15,7	0,6	2	1	21	2012
17,5	34,2	0,6	8	1	20	2013
17,5	34,2	0,6	7	1	18	2014
12,9	13,7	0,6	2	1	22	2009
12,9	13,7	0,6	2	1	22	2013
12,9	13,7	0,6	3	1	18	2014
15,7	24,7	0,6	5	1	18	2000
15,7	24,7	0,6	4	1	18	2014
14,8	20,7	0,6	5	1	21	2012
14,8	20,7	0,6	3	1	19	2012
14,1	17,9	0,6	3	1	18	2008

14,1	17,9	0,6	5	1	20	2012
14,1	17,9	0,6	4	1	23	2015
11	8,5	0,6	2	1	23	2004
18,6	41,1	0,6	4	1	19	2012
12,2	11,6	0,6	2	1	23	2007
15,8	25,2	0,6	4	1	20	2013
12,8	13,4	0,6	2	1	24	2006
12,8	13,4	0,6	2	1	26	2009
12,8	13,4	0,6	3	1	23	2010
12,8	13,4	0,6	3	1	23	2010
12,8	13,4	0,6	3	1	17	2014
15,1	22	0,6	3	1	26	2003
15,1	22	0,6	5	1	20	2009
15,1	22	0,6	5	1	19	2012
15,1	22	0,6	5	1	21	2012
15,1	22	0,6	4	1	19	2015
14,7	20,3	0,6	4	1	18	1999
14,7	20,3	0,6	3	1	19	2001
14,7	20,3	0,6	3	1	23	2003

14,2	18,3	0,6	4	1	20	2009
14,2	18,3	0,6	3	1	21	2014
14,2	18,3	0,6	3	1	21	2015
13,2	14,7	0,6	2	1	21	2013
13,2	14,7	0,6	3	1	17	2014
13,8	16,8	0,6	2	1	27	1999
13,8	16,8	0,6	3	1	17	2008
13,8	16,8	0,6	3	1	22	2015
16,4	28,2	0,6	4	1	19	2012
15,9	25,7	0,6	5	1	21	1999
15,3	22,9	0,6	3	1	19	2012
15,3	22,9	0,6	4	1	19	2012
14,3	18,7	0,6	3	1	20	2009
14,3	18,7	0,6	7	1	21	2012
14,3	18,7	0,6	3	1	19	2013
14,3	18,7	0,6	4	1	15	2015
14,3	18,7	0,6	3	1	18	2015
12,7	13,1	0,6	2	1	21	2014
14,5	19,5	0,6	5	1	26	2000

14,5	19,5	0,6	6	1	19	2008
14,5	19,5	0,6	5	1	21	2012
18,5	40,5	0,6	6	1	18	2015
16	26,2	0,6	5	1	24	2009
16	26,2	0,6	5	1	21	2013
14,4	19,1	0,6	4	1	25	1999
14,4	19,1	0,6	3	1	19	2002
14,4	19,1	0,6	3	1	19	2015
16,2	27,2	0,6	6	1	21	1999
16,2	27,2	0,6	7	1	17	2009
12,4	12,2	0,6	2	1	19	2002
12,6	12,8	0,6	2	1	19	2013
15	21,6	0,6	3	1	21	1999
12,5	12,5	0,6	2	1	19	2000
15	21,6	0,6	4	1	22	2001
15	21,6	0,6	7	1	22	2005
12,5	12,5	0,6	2	1	19	2006
15	21,6	0,6	6	1	23	2007
12,5	12,5	0,6	2	1	22	2009

12,5	12,5	0,6	2	1	23	2010
12,5	12,5	0,6	2	1	23	2010
15	21,6	0,6	3	1	19	2012
15	21,6	0,6	3	1	19	2012
15	21,6	0,6	5	1	19	2012
12,5	12,5	0,6	2	1	18	2014
15	21,6	0,6	4	1	16	2015
13,4	15,4	0,6	3	1	24	2009
13,4	15,4	0,6	4	1	18	2015
15,6	24,3	0,6	3	1	19	2000
15,6	24,3	0,6	6	1	18	2012
17,8	36,1	0,6	6	1	20	2013
13,9	17,2	0,6	3	1	25	2002
13,9	17,2	0,6	3	1	20	2010
13,9	17,2	0,6	3	1	20	2010
13,9	17,2	0,6	4	1	21	2012
16,6	29,3	0,6	6	1	20	2001
13,1	14,4	0,6	2	1	23	2001
13,1	14,4	0,6	3	1	25	2007

13,1	14,4	0,6	2	1	17	2009
13,1	14,4	0,6	3	1	22	2009
13,1	14,4	0,6	4	1	21	2011
13,1	14,4	0,6	2	1	17	2014
13,1	14,4	0,6	2	1	21	2014
13,1	14,4	0,6	2	1	21	2014
13,1	14,4	0,6	2	1	23	2014
11,2	9	0,6	1	1	24	2009
11,6	10	0,6	2	1	23	2005
15,2	22,5	0,6	4	1	26	2000
15,2	22,5	0,6	3	1	26	2001
15,2	22,5	0,6	3	1	20	2006
15,2	22,5	0,6	4	1	20	2011
15,2	22,5	0,6	3	1	19	2012
15,4	23,4	0,6	3	1	18	2001
15,4	23,4	0,6	7	1	23	2006
15,4	23,4	0,6	3	1	16	2008
15,4	23,4	0,6	4	1	21	2011
20,5	55,2	0,6	5	1	17	2014

15,7	24,8	0,6	3	1	18	2015
14,9	21,2	0,6	4	1	21	2012
14	17,6	0,6	4	1	25	1999
14	17,6	0,6	2	1	21	2000
14	17,6	0,6	4	1	25	2004
14	17,6	0,6	7	1	20	2009
14	17,6	0,6	3	1	26	2009
15,8	25,3	0,6	3	1	18	2001
19,2	45,4	0,6	8	1	22	2013
16,4	28,3	0,6	5	1	18	1999
16,4	28,3	0,6	8	1	22	2003
17,4	33,8	0,6	5	1	18	2000
17,4	33,8	0,6	5	1	20	2000
14,8	20,8	0,6	3	1	21	2000
22,2	70,2	0,6	7	1	20	2013
13,7	16,5	0,6	3	1	21	2009
13	14,1	0,6	2	1	18	2000
13	14,1	0,6	2	1	21	2008
13	14,1	0,6	2	1	23	2010

13	14,1	0,6	2	1	23	2010
13	14,1	0,6	2	1	22	2011
15,5	23,9	0,6	3	1	22	2001
15,5	23,9	0,6	5	1	19	2012
13,3	15,1	0,6	3	1	22	2002
13,3	15,1	0,6	3	1	21	2011
13,3	15,1	0,6	3	1	17	2014
17,5	34,4	0,6	4	1	19	2013
15,1	22,1	0,6	4	1	22	2007
16,3	27,8	0,6	6	1	19	2013
16,7	29,9	0,6	6	1	24	2001
16	26,3	0,6	3	1	18	2002
16	26,3	0,6	5	1	24	2002
16	26,3	0,6	6	1	19	2013
14,1	18	0,6	3	1	23	2003
14,1	18	0,6	3	1	20	2008
14,1	18	0,6	2	1	21	2014
14,1	18	0,6	2	1	23	2014
16,2	27,3	0,6	7	1	26	2003

15,3	23	0,6	3	1	18	2000
15,3	23	0,6	3	1	25	2004
15,3	23	0,6	6	1	21	2008
15,3	23	0,6	4	1	19	2013
13,5	15,8	0,6	3	1	22	2000
13,5	15,8	0,6	2	1	25	2002
13,5	15,8	0,6	2	1	19	2013
14,7	20,4	0,6	5	1	22	2009
16,9	31	0,6	4	1	19	2012
12	11,1	0,6	2	1	20	2004
12	11,1	0,6	3	1	22	2005
12	11,1	0,6	2	1	22	2007
12	11,1	0,6	2	1	18	2014
14,2	18,4	0,6	3	1	25	2003
17,2	32,7	0,6	9	1	26	2003
17,2	32,7	0,6	6	1	19	2011
15,6	24,4	0,6	7	1	24	2006
18,3	39,4	0,6	7	1	19	2013
14,3	18,8	0,6	3	1	21	2000

14,3	18,8	0,6	4	1	25	2004
14,3	18,8	0,6	3	1	18	2008
14,3	18,8	0,6	5	1	19	2012
14,5	19,6	0,6	5	1	19	1999
15	21,7	0,6	5	1	21	2006
15	21,7	0,6	4	1	22	2011
10,8	8,1	0,6	2	1	23	2004
10,8	8,1	0,6	2	1	25	2005
14,4	19,2	0,6	3	1	17	2012
14,4	19,2	0,6	3	1	21	2015
13,8	16,9	0,6	4	1	26	1999
13,8	16,9	0,6	3	1	18	2007
13,8	16,9	0,6	3	1	23	2010
13,8	16,9	0,6	3	1	23	2010
13,8	16,9	0,6	3	1	19	2011
13,8	16,9	0,6	3	1	21	2015
17	31,6	0,6	6	1	19	2013
17	31,6	0,6	4	1	16	2015
15,4	23,5	0,6	3	1	21	2003

13,2	14,8	0,6	2	1	21	2006
13,2	14,8	0,6	4	1	26	2009
13,2	14,8	0,6	4	1	22	2011
13,2	14,8	0,6	2	1	23	2011
12,1	11,4	0,6	2	1	19	2014
15,2	22,6	0,6	4	1	20	2001
15,2	22,6	0,6	4	1	24	2002
15,2	22,6	0,6	5	1	23	2002
15,2	22,6	0,6	3	1	17	2008
15,2	22,6	0,6	6	1	22	2011
12,8	13,5	0,6	3	1	25	2005
12,8	13,5	0,6	3	1	21	2008
12,8	13,5	0,6	2	1	20	2009
16,4	28,4	0,6	6	1	23	2007
14,9	21,3	0,6	5	1	18	1999
14,9	21,3	0,6	4	1	18	2002
14,9	21,3	0,6	4	1	20	2012
15,8	25,4	0,6	4	1	20	2003
15,8	25,4	0,6	6	1	23	2003

16,7	30	0,6	3	1	18	2014
13,9	17,3	0,6	3	1	21	2008
13,4	15,5	0,6	2	1	19	2000
16,3	27,9	0,6	6	1	18	2000
16,3	27,9	0,6	4	1	19	2000
16,3	27,9	0,6	6	1	20	2011
12,2	11,7	0,6	2	1	20	2008
12,2	11,7	0,6	2	1	19	2014
15,9	25,9	0,6	4	1	23	2005
12,7	13,2	0,6	3	1	22	2005
12,7	13,2	0,6	2	1	23	2009
16,2	27,4	0,6	4	1	16	2015
15,5	24	0,6	4	1	23	2011
16	26,4	0,6	5	1	19	2000
16	26,4	0,6	4	1	20	2001
16	26,4	0,6	3	1	19	2012
11,3	9,3	0,6	1	1	26	2001
16,1	26,9	0,6	5	1	18	2000
16,1	26,9	0,6	5	1	19	2012

14,8	20,9	0,6	3	1	17	2002
14,8	20,9	0,6	6	1	23	2007
14,8	20,9	0,6	4	1	19	2012
15,1	22,2	0,6	5	1	24	2001
12,3	12	0,6	2	1	22	2005
12,3	12	0,6	2	1	19	2011
12,3	12	0,6	2	1	19	2013
12,6	12,9	0,6	2	1	21	2008
16,6	29,5	0,6	4	1	20	2013
15,3	23,1	0,6	5	1	20	2003
13,1	14,5	0,6	3	1	26	2003
20	51,6	0,6	6	1	18	2015
14	17,7	0,6	5	1	22	1999
14	17,7	0,6	2	1	22	2000
14	17,7	0,6	2	1	26	2000
14	17,7	0,6	2	1	26	2003
14	17,7	0,6	4	1	25	2008
14	17,7	0,6	4	1	20	2011
12,4	12,3	0,6	2	1	22	2011

12,4	12,3	0,6	2	1	20	2013
12,4	12,3	0,6	2	1	17	2014
12,5	12,6	0,6	2	1	22	2001
12,5	12,6	0,6	2	1	20	2002
11,8	10,6	0,6	2	1	23	2005
14,7	20,5	0,6	5	1	20	1999
14,7	20,5	0,6	4	1	24	2009
14,7	20,5	0,6	7	1	19	2012
20,2	53,2	0,6	6	1	17	2014
16,5	29	0,6	6	1	18	2002
13,7	16,6	0,6	3	1	25	1999
13,7	16,6	0,6	2	1	24	2002
13,7	16,6	0,6	4	1	24	2009
13,7	16,6	0,6	4	1	22	2011
17,6	35,2	0,6	4	1	15	2015
14,1	18,1	0,6	3	1	20	2006
14,1	18,1	0,6	5	1	25	2007
14,1	18,1	0,6	6	1	17	2008
14,1	18,1	0,6	3	1	20	2008

14,1	18,1	0,6	4	1	22	2009
14,6	20,1	0,6	5	1	18	2000
14,6	20,1	0,6	7	1	22	2007
15	21,8	0,6	3	1	24	2001
15	21,8	0,6	4	1	22	2004
15	21,8	0,6	3	1	18	2015
13,3	15,2	0,6	3	1	23	2003
13,3	15,2	0,6	4	1	25	2004
14,2	18,5	0,6	5	1	23	1999
14,2	18,5	0,6	5	1	26	1999
14,2	18,5	0,6	5	1	20	2007
16,4	28,5	0,6	6	1	22	2003
14,5	19,7	0,6	3	1	22	2001
14,5	19,7	0,6	4	1	20	2006
14,5	19,7	0,6	6	1	20	2013
13,5	15,9	0,6	2	1	20	2002
20,9	59	0,6	8	1	21	2013
16,7	30,1	0,6	4	1	18	2000
16,7	30,1	0,6	6	1	19	2000

16,7	30,1	0,6	5	1	21	2001
14,3	18,9	0,6	5	1	22	1999
14,3	18,9	0,6	5	1	19	2000
13	14,2	0,6	5	1	26	1999
13	14,2	0,6	2	1	26	2001
13	14,2	0,6	2	1	26	2003
13	14,2	0,6	3	1	23	2005
13	14,2	0,6	2	1	20	2009
13	14,2	0,6	2	1	18	2014
13	14,2	0,6	2	1	19	2014
13	14,2	0,6	2	1	23	2015
14,4	19,3	0,6	3	1	22	2002
14,4	19,3	0,6	2	1	25	2003
14,4	19,3	0,6	3	1	26	2003
14,4	19,3	0,6	3	1	15	2015
15,2	22,7	0,6	4	1	21	1999
15,2	22,7	0,6	5	1	21	2006
15,2	22,7	0,6	6	1	23	2008
18	37,7	0,6	6	1	20	2013

19,4	47,2	0,6	6	1	20	2013
15,8	25,5	0,6	3	1	26	2003
15,8	25,5	0,6	6	1	22	2007
15,8	25,5	0,6	5	1	19	2012
15,8	25,5	0,6	3	1	18	2015
17,2	32,9	0,6	10	1	17	2008
15,9	26	0,6	4	1	17	2012
13,8	17	0,6	3	1	21	2000
13,8	17	0,6	2	1	21	2006
13,8	17	0,6	3	1	21	2009
13,8	17	0,6	4	1	19	2012
14,9	21,4	0,6	10	1	25	2007
16	26,5	0,6	6	1	26	2001
16	26,5	0,6	5	1	18	2015
11,6	10,1	0,6	2	1	22	2007
16,6	29,6	0,6	5	1	18	2001
16,6	29,6	0,6	5	1	19	2012
15,5	24,1	0,6	6	1	21	1999
15,5	24,1	0,6	5	1	22	1999

15,5	24,1	0,6	5	1	26	2000
15,5	24,1	0,6	3	1	18	2001
15,5	24,1	0,6	3	1	26	2001
15,5	24,1	0,6	5	1	19	2006
17,8	36,5	0,6	4	1	19	2013
17,7	35,9	0,6	4	1	18	2012
17,7	35,9	0,6	6	1	22	2013
16,8	30,7	0,6	4	1	22	2013
16,8	30,7	0,6	4	1	20	2013
12,9	13,9	0,6	2	1	19	2014
18,1	38,4	0,6	9	1	23	2001
15,1	22,3	0,6	3	1	19	2012
15,1	22,3	0,6	3	1	19	2013
15,3	23,2	0,6	4	1	22	2001
15,3	23,2	0,6	5	1	26	2001
16,5	29,1	0,6	7	1	21	2012
16,5	29,1	0,6	5	1	21	2013
13,2	14,9	0,6	3	1	18	2007
13,2	14,9	0,6	3	1	23	2008

10,5	7,5	0,6	2	1	20	2004
13,9	17,4	0,6	3	1	25	2007
13,9	17,4	0,6	2	1	21	2013
11,4	9,6	0,6	2	1	23	2004
15,6	24,6	0,6	5	1	19	2000
13,6	16,3	0,6	2	1	23	1999
13,6	16,3	0,6	3	1	20	2006
13,6	16,3	0,6	3	1	19	2012
12	11,2	0,6	2	1	22	1999
12	11,2	0,6	2	1	24	2009
13,4	15,6	0,6	4	1	26	1999
13,4	15,6	0,6	2	1	24	2009
16,7	30,2	0,6	4	1	20	2013
12,8	13,6	0,6	2	1	20	2013
14,7	20,6	0,6	5	1	21	2000
14,7	20,6	0,6	3	1	24	2001
14,7	20,6	0,6	3	1	24	2001
14,7	20,6	0,6	6	1	20	2013
14,7	20,6	0,6	3	1	18	2014

14	17,8	0,6	5	1	26	1999
14	17,8	0,6	4	1	23	2005
14	17,8	0,6	3	1	25	2007
14	17,8	0,6	3	1	22	2011
14	17,8	0,6	2	1	19	2014
16,3	28,1	0,6	5	1	19	2010
16,3	28,1	0,6	5	1	19	2010
15	21,9	0,6	6	1	25	2004
15	21,9	0,6	4	1	21	2005
15,4	23,7	0,6	3	1	20	2003
15,4	23,7	0,6	4	1	22	2011
15,4	23,7	0,6	4	1	19	2012
17,3	33,6	0,6	6	1	23	2002
20,1	52,7	0,6	4	1	19	2013
15,8	25,6	0,6	3	1	18	2001
15,8	25,6	0,6	5	1	23	2005
15,8	25,6	0,6	6	1	22	2011
14,6	20,2	0,6	4	1	21	2001
14,6	20,2	0,6	4	1	19	2012

16,2	27,6	0,6	5	1	19	1999
16,2	27,6	0,6	4	1	26	1999
15,2	22,8	0,6	5	1	19	1999
15,2	22,8	0,6	3	1	21	2001
15,2	22,8	0,6	3	1	22	2003
14,1	18,2	0,6	4	1	25	2004
14,1	18,2	0,6	5	1	25	2007
14,1	18,2	0,6	3	1	16	2008
14,1	18,2	0,6	3	1	19	2012
14,1	18,2	0,6	3	1	21	2015
17	31,9	0,6	10	1	19	2012
17	31,9	0,6	4	1	19	2013
11,7	10,4	0,6	2	1	23	2007
16	26,6	0,6	4	1	20	1999
16	26,6	0,6	6	1	22	1999
16	26,6	0,6	6	1	26	2005
16	26,6	0,6	6	1	21	2013
13,1	14,6	0,6	2	1	25	1999
13,1	14,6	0,6	2	1	25	2003

13,1	14,6	0,6	2	1	21	2008
13,1	14,6	0,6	2	1	22	2011
19,6	48,9	0,6	8	1	22	2013
13,7	16,7	0,6	3	1	26	1999
13,7	16,7	0,6	2	1	21	2011
13,7	16,7	0,6	3	1	18	2015
14,5	19,8	0,6	3	1	22	2001
14,5	19,8	0,6	4	1	21	2009
14,5	19,8	0,6	2	1	19	2013
16,8	30,8	0,6	4	1	23	2003
14,2	18,6	0,6	3	1	21	2000
14,2	18,6	0,6	4	1	25	2004
14,2	18,6	0,6	3	1	21	2005
14,2	18,6	0,6	3	1	17	2008
14,4	19,4	0,6	5	1	27	1999
14,4	19,4	0,6	3	1	26	2000
14,4	19,4	0,6	3	1	21	2005
14,4	19,4	0,6	5	1	23	2009
14,4	19,4	0,6	3	1	21	2012

14,3	19	0,6	4	1	21	2009
14,3	19	0,6	3	1	18	2015
12,2	11,8	0,6	2	1	18	2000
12,2	11,8	0,6	3	1	23	2005
12,2	11,8	0,6	2	1	20	2007
12,2	11,8	0,6	2	1	25	2007
12,2	11,8	0,6	2	1	20	2012
12,2	11,8	0,6	2	1	20	2013
15,5	24,2	0,6	5	1	21	1999
15,5	24,2	0,6	5	1	25	2002
12,6	13	0,6	2	1	23	2007
16,5	29,2	0,7	4	1	24	2001
20,8	58,5	0,7	5	1	16	2015
12,3	12,1	0,7	2	1	20	2002
12,3	12,1	0,7	2	1	19	2013
12,3	12,1	0,7	2	1	18	2014
12,5	12,7	0,7	2	1	21	2006
12,5	12,7	0,7	3	1	23	2010
12,5	12,7	0,7	3	1	23	2010

12,5	12,7	0,7	2	1	20	2013
13,5	16	0,7	2	1	22	2009
13,3	15,3	0,7	2	1	19	2000
13,3	15,3	0,7	3	1	16	2015
12,4	12,4	0,7	2	1	22	2002
12,4	12,4	0,7	2	1	19	2013
15,3	23,3	0,7	3	1	16	2015
15,3	23,3	0,7	3	1	21	2015
16,7	30,3	0,7	6	1	20	2013
15,1	22,4	0,7	4	1	26	2001
15,1	22,4	0,7	3	1	19	2010
15,1	22,4	0,7	3	1	19	2010
15,6	24,7	0,7		1	20	2011
13,8	17,1	0,7	3	1	25	2007
13	14,3	0,7	3	1	23	2005
13	14,3	0,7	4	1	21	2006
13	14,3	0,7	2	1	26	2009
13	14,3	0,7	2	1	23	2010
13	14,3	0,7	2	1	23	2010

13	14,3	0,7	2	1	19	2013
13	14,3	0,7	2	1	18	2014
11,5	9,9	0,7	2	1	20	2004
11,5	9,9	0,7	2	1	26	2006
17,6	35,5	0,7	4	1	19	2012
15,7	25,2	0,7	5	1	19	1999
15,7	25,2	0,7	5	1	19	2000
17,5	34,9	0,7	6	1	22	2013
17	32	0,7	5	1	19	2012
18,7	42,6	0,7	6	1	20	2013
11,3	9,4	0,7	2	1	22	2004
11,3	9,4	0,7	2	1	25	2004
16,2	27,7	0,7	5	1	25	2004
15,8	25,7	0,7	3	1	17	2012
20,9	59,5	0,7	6	1	17	2012
16,1	27,2	0,7	5	1	19	2000
15	22	0,7	5	1	20	2004
15	22	0,7	3	1	17	2008
16	26,7	0,7	6	1	22	2000

13,6	16,4	0,7	2	1	26	2001
13,6	16,4	0,7	4	1	25	2005
13,6	16,4	0,7	2	1	19	2010
13,6	16,4	0,7	2	1	19	2010
13,6	16,4	0,7	3	1	22	2015
15,2	22,9	0,7	5	1	20	2012
13,2	15	0,7	3	1	25	2007
13,2	15	0,7	3	1	19	2008
13,2	15	0,7	3	1	18	2014
16,5	29,3	0,7	6	1	20	2013
14,6	20,3	0,7	5	1	18	1999
14,6	20,3	0,7	5	1	22	1999
14,6	20,3	0,7	6	1	22	2002
14,6	20,3	0,7	4	1	26	2006
14	17,9	0,7	3	1	26	1999
14	17,9	0,7	5	1	20	2000
14	17,9	0,7	3	1	21	2003
14	17,9	0,7	4	1	25	2004
14	17,9	0,7	2	1	17	2014

13,4	15,7	0,7	4	1	22	1999
13,4	15,7	0,7	6	1	21	2008
13,4	15,7	0,7	4	1	25	2008
13,4	15,7	0,7	5	1	19	2012
13,4	15,7	0,7	2	1	19	2013
15,5	24,3	0,7	5	1	23	1999
15,5	24,3	0,7	4	1	22	2004
15,5	24,3	0,7	4	1	22	2011
16,9	31,5	0,7	5	1	19	2012
16,7	30,4	0,7	6	1	20	2013
14,5	19,9	0,7	5	1	22	1999
11,9	11	0,7	2	1	19	2006
17,7	36,2	0,7	5	1	18	2014
14,1	18,3	0,7	4	1	25	1999
14,1	18,3	0,7	3	1	21	2008
14,1	18,3	0,7	3	1	19	2014
14,1	18,3	0,7	3	1	15	2015
16,4	28,8	0,7	4	1	18	2015
14,9	21,6	0,7	4	1	25	2003

14,9	21,6	0,7	3	1	19	2008
14,4	19,5	0,7	2	1	24	2002
14,4	19,5	0,7	3	1	24	2002
14,2	18,7	0,7	3	1	21	1999
14,2	18,7	0,7	4	1	21	2006
14,2	18,7	0,7	4	1	20	2007
14,2	18,7	0,7	4	1	23	2008
14,2	18,7	0,7	3	1	25	2008
14,3	19,1	0,7	5	1	27	1999
14,3	19,1	0,7	2	1	21	2000
14,3	19,1	0,7	3	1	19	2002
14,3	19,1	0,7	4	1	20	2011
15,6	24,8	0,7	4	1	24	2001
15,6	24,8	0,7	4	1	22	2003
12,8	13,7	0,7	3	1	25	2007
12,8	13,7	0,7	2	1	22	2011
15,3	23,4	0,7	4	1	19	2001
15,3	23,4	0,7	5	1	20	2013
17	32,1	0,7	5	1	21	2013

17	32,1	0,7	6	1	22	2013
16,3	28,3	0,7	5	1	19	2000
16,3	28,3	0,7	7	1	25	2004
15,1	22,5	0,7	5	1	20	1999
15,1	22,5	0,7	3	1	21	2001
15,1	22,5	0,7	3	1	23	2008
11	8,7	0,7	2	1	20	2004
11	8,7	0,7	2	1	23	2004
16,6	29,9	0,7	4	1	20	2004
16,8	31	0,7	4	1	18	2002
16,2	27,8	0,7	4	1	19	2001
16,2	27,8	0,7	6	1	18	2007
13,1	14,7	0,7	2	1	19	2002
13,1	14,7	0,7	2	1	23	2008
13,1	14,7	0,7	9	1	20	2011
12	11,3	0,7	2	1	21	2006
12	11,3	0,7	2	1	23	2007
12	11,3	0,7	2	1	26	2009
17,1	32,7	0,7	4	1	18	2012

16,1	27,3	0,7	4	1	19	2012
12,7	13,4	0,7	2	1	22	2009
15,9	26,3	0,7	6	1	21	2013
16	26,8	0,7	6	1	24	2006
13,5	16,1	0,7	3	1	22	2011
13,5	16,1	0,7	3	1	23	2015
15,4	23,9	0,7	4	1	16	2015
13,8	17,2	0,7	3	1	25	2008
13,3	15,4	0,7	2	1	22	1999
13,3	15,4	0,7	4	1	23	2004
13,3	15,4	0,7	2	1	21	2012
12,1	11,6	0,7	3	1	22	2005
12,1	11,6	0,7	2	1	22	2007
12,1	11,6	0,7	2	1	25	2007
12,1	11,6	0,7	2	1	18	2014
14,7	20,8	0,7	5	1	25	1999
15	22,1	0,7	6	1	21	2006
15	22,1	0,7	4	1	23	2011
15	22,1	0,7	3	1	19	2013

11,2	9,2	0,7	2	1	23	2004
10,6	7,8	0,7	1	1	25	2008
18	38,2	0,7	5	1	19	2013
16,4	28,9	0,7	5	1	21	1999
16,4	28,9	0,7	4	1	18	2002
16,4	28,9	0,7	4	1	19	2013
15,5	24,4	0,7	4	1	18	1999
15,5	24,4	0,7	5	1	19	2012
12,2	11,9	0,7	2	1	24	2006
12,2	11,9	0,7	2	1	25	2007
12,2	11,9	0,7	2	1	21	2011
12,2	11,9	0,7	2	1	22	2011
13,9	17,6	0,7	3	1	23	2008
17	32,2	0,7	8	1	21	2013
17	32,2	0,7	5	1	20	2013
17	32,2	0,7	6	1	15	2015
13	14,4	0,7	2	1	21	2000
13	14,4	0,7	2	1	22	2002
13	14,4	0,7	2	1	23	2002

14,6	20,4	0,7	3	1	16	2015
17,9	37,6	0,7	4	1	18	2015
11,7	10,5	0,7	2	1	20	2004
12,3	12,2	0,7	2	1	23	2008
12,3	12,2	0,7	2	1	17	2014
15,6	24,9	0,7	6	1	20	2011
13,6	16,5	0,7	4	1	21	2006
14	18	0,7	4	1	20	2009
14	18	0,7	3	1	20	2011
14	18	0,7	3	1	16	2015
14,9	21,7	0,7	8	1	19	2011
18,1	38,9	0,7	6	1	19	2013
14,5	20	0,7	3	1	24	2001
14,5	20	0,7	3	1	23	2003
14,5	20	0,7	4	1	16	2015
15,3	23,5	0,7	6	1	27	1999
15,7	25,4	0,7	3	1	22	2001
15,7	25,4	0,7	5	1	19	2012
14,1	18,4	0,7	4	1	18	2014

14,1	18,4	0,7	3	1	21	2014
14,4	19,6	0,7	2	1	26	2001
14,4	19,6	0,7	4	1	19	2004
14,4	19,6	0,7	5	1	19	2007
14,4	19,6	0,7	3	1	22	2011
14,4	19,6	0,7	4	1	20	2013
15,1	22,6	0,7	3	1	21	2003
15,1	22,6	0,7	3	1	21	2003
13,2	15,1	0,7	3	1	20	2008
19,3	47,2	0,7	5	1	21	2010
19,3	47,2	0,7	5	1	21	2010
16,1	27,4	0,7	5	1	18	1999
16,1	27,4	0,7	3	1	20	2013
14,2	18,8	0,7	5	1	20	2000
14,2	18,8	0,7	3	1	26	2003
14,2	18,8	0,7	6	1	17	2008
14,2	18,8	0,7	4	1	19	2008
14,2	18,8	0,7	3	1	21	2008
14,2	18,8	0,7	3	1	21	2015

14,3	19,2	0,7	7	1	25	1999
14,3	19,2	0,7	5	1	26	1999
14,3	19,2	0,7	2	1	19	2000
14,3	19,2	0,7	3	1	23	2002
14,3	19,2	0,7	4	1	23	2010
14,3	19,2	0,7	4	1	23	2010
13,4	15,8	0,7	2	1	26	2001
13,4	15,8	0,7	2	1	20	2013
16,5	29,5	0,7	5	1	22	1999
16,5	29,5	0,7	5	1	20	2013
15,9	26,4	0,7	5	1	25	2007
14,8	21,3	0,7	3	1	22	2011
21,5	65,3	0,7	5	1	17	2014
13,7	16,9	0,7	2	1	27	1999
15	22,2	0,7	3	1	22	2003
15	22,2	0,7	3	1	25	2007
15	22,2	0,7	5	1	20	2009
15,2	23,1	0,7	5	1	22	1999
15,2	23,1	0,7	5	1	19	2013

15,5	24,5	0,7	5	1	21	1999
15,5	24,5	0,7	3	1	26	2001
15,5	24,5	0,7	4	1	20	2003
17,1	32,9	0,7	7	1	19	2014
16,6	30,1	0,7	4	1	18	2002
11,1	9	0,7	2	1	23	2007
21,4	64,5	0,7	6	1	17	2014
13,8	17,3	0,7	2	1	22	2001
13,1	14,8	0,7	3	1	23	2001
13,1	14,8	0,7	2	1	20	2002
13,1	14,8	0,7	3	1	23	2005
13,1	14,8	0,7	3	1	25	2007
11,3	9,5	0,7	1	1	26	2001
11,3	9,5	0,7	2	1	20	2004
11,3	9,5	0,7	2	1	18	2007
11,3	9,5	0,7	2	1	19	2007
13,5	16,2	0,7	4	1	27	1999
13,5	16,2	0,7	2	1	15	2015
15,6	25	0,7	4	1	19	2002

18,5	41,7	0,7	7	1	18	2012
14,6	20,5	0,7	8	1	20	2000
14,6	20,5	0,7	4	1	20	2002
14,6	20,5	0,7	3	1	23	2003
13,3	15,5	0,7	3	1	23	2008
13,3	15,5	0,7	2	1	20	2013
13,3	15,5	0,7	3	1	22	2015
10,8	8,3	0,7	2	1	23	2004
15,3	23,6	0,7	4	1	19	2013
15,7	25,5	0,7	4	1	21	1999
14,9	21,8	0,7	3	1	19	2012
12,7	13,5	0,7	2	1	25	1999
12,7	13,5	0,7	2	1	26	1999
12,7	13,5	0,7	2	1	22	2005
12,7	13,5	0,7	2	1	20	2013
13,9	17,7	0,7	3	1	19	2012
13,9	17,7	0,7	2	1	21	2014
13,9	17,7	0,7	2	1	23	2014
16,7	30,7	0,7	6	1	19	2013

15,8	26	0,7	3	1	22	2003
16	27	0,7	5	1	20	1999
14,5	20,1	0,7	3	1	23	2007
14,5	20,1	0,7	6	1	20	2013
15,1	22,7	0,7	4	1	23	2003
19,8	51,2	0,7	4	1	19	2013
14	18,1	0,7	2	1	25	1999
14	18,1	0,7	2	1	19	2001
14	18,1	0,7	2	1	24	2002
14	18,1	0,7	3	1	19	2008
14	18,1	0,7	3	1	19	2014
14	18,1	0,7	3	1	18	2015
12	11,4	0,7	3	1	21	2005
12	11,4	0,7	2	1	23	2007
12	11,4	0,7	2	1	23	2007
14,4	19,7	0,7	5	1	22	1999
15,4	24,1	0,7	4	1	19	2011
12,6	13,2	0,7	2	1	23	2010
12,6	13,2	0,7	2	1	23	2010

11,6	10,3	0,7	1	1	26	2001
11,6	10,3	0,7	2	1	21	2006
13,6	16,6	0,7	2	1	24	2001
14,1	18,5	0,7	6	1	23	2008
13	14,5	0,7	2	1	21	2006
13	14,5	0,7	3	1	25	2007
13	14,5	0,7	2	1	25	2007
13	14,5	0,7	2	1	21	2008
13	14,5	0,7	2	1	17	2014
14,3	19,3	0,7	4	1	23	1999
14,3	19,3	0,7	3	1	19	2000
14,3	19,3	0,7	4	1	17	2012
14,3	19,3	0,7	4	1	15	2015
14,3	19,3	0,7	3	1	18	2015
14,2	18,9	0,7	3	1	21	2005
14,2	18,9	0,7	3	1	20	2008
14,2	18,9	0,7	4	1	20	2008
14,2	18,9	0,7	3	1	21	2008
14,2	18,9	0,7	4	1	19	2012

14,8	21,4	0,7	4	1	21	1999
18,5	41,8	0,7	7	1	17	2014
16,6	30,2	0,7	4	1	18	2012
17,6	36	0,7	5	1	19	2012
12,1	11,7	0,7	2	1	19	2014
19	45,3	0,7	9	1	19	2010
19	45,3	0,7	9	1	19	2010
19	45,3	0,7	6	1	20	2013
17,5	35,4	0,7	6	1	19	2000
15,5	24,6	0,7	4	1	20	2013
18,9	44,6	0,7	8	1	19	2013
15,2	23,2	0,7	3	1	16	2008
15,2	23,2	0,7	3	1	19	2014
15	22,3	0,7	3	1	17	2002
15	22,3	0,7	4	1	25	2002
15	22,3	0,7	5	1	20	2009
13,4	15,9	0,7	3	1	25	1999
13,4	15,9	0,7	5	1	23	2007
13,4	15,9	0,7	2	1	20	2008

12,2	12	0,7	2	1	20	2002
12,2	12	0,7	2	1	25	2007
12,4	12,6	0,7	2	1	22	2002
12,4	12,6	0,7	2	1	26	2009
13,2	15,2	0,7	4	1	26	1999
13,2	15,2	0,7	3	1	23	2008
13,2	15,2	0,7	3	1	20	2011
16,2	28,1	0,7	6	1	19	2007
16,2	28,1	0,7	10	1	20	2011
12,3	12,3	0,7	2	1	20	2002
12,3	12,3	0,7	2	1	23	2005
12,3	12,3	0,7	2	1	25	2007
14,7	21	0,7	3	1	22	2000
13,7	17	0,7	4	1	22	2009
15,6	25,1	0,7	5	1	18	1999
11	8,8	0,7	2	1	22	2004
11	8,8	0,7	2	1	25	2004
11	8,8	0,7	2	1	26	2009
16,1	27,6	0,7	4	1	20	2001

11,4	9,8	0,7	2	1	23	2004
17	32,5	0,7	5	1	19	2014
15,7	25,6	0,7	7	1	25	1999
15,7	25,6	0,7	6	1	22	2003
15,8	26,1	0,7	4	1	23	2002
15,3	23,7	0,7	4	1	23	2002
15,3	23,7	0,7	4	1	21	2009
17,7	36,7	0,7	5	1	18	2000
21	61,3	0,7	7	1	18	2012
14,6	20,6	0,7	3	1	27	1999
14,6	20,6	0,7	6	1	25	2007
14,6	20,6	0,7	4	1	21	2011
11,2	9,3	0,7	1	1	24	2009
16,4	29,2	0,7	5	1	18	2001
16,4	29,2	0,7	3	1	23	2015
18,6	42,6	0,7	8	1	22	2013
14,9	21,9	0,7	4	1	20	1999
14,9	21,9	0,7	3	1	23	2007
13,8	17,4	0,7	4	1	23	1999

13,8	17,4	0,7	3	1	20	2002
15,1	22,8	0,7	3	1	26	2003
15,1	22,8	0,7	4	1	16	2015
17,2	33,7	0,7	9	1	18	2000
16,6	30,3	0,7	3	1	21	2003
13,5	16,3	0,7	2	1	19	2000
13,5	16,3	0,7	2	1	26	2000
13,5	16,3	0,7	2	1	23	2003
13,5	16,3	0,7	4	1	26	2006
13,5	16,3	0,7	3	1	18	2008
13,5	16,3	0,7	3	1	20	2011
13,5	16,3	0,7	3	1	18	2015
14,5	20,2	0,7	4	1	21	1999
14,5	20,2	0,7	5	1	22	2004
15,4	24,2	0,7	5	1	18	1999
15,4	24,2	0,7	4	1	24	2001
15,4	24,2	0,7	4	1	22	2004
13,1	14,9	0,7	3	1	15	2015
13,9	17,8	0,7	3	1	25	2007

12,8	13,9	0,7	5	1	23	2010
12,8	13,9	0,7	5	1	23	2010
13,3	15,6	0,7	3	1	22	2005
13,3	15,6	0,7	2	1	22	2011
13,3	15,6	0,7	2	1	19	2013
13,3	15,6	0,7	2	1	19	2013
13,3	15,6	0,7	3	1	19	2014
14,4	19,8	0,7	4	1	26	2003
17,8	37,4	0,7	5	1	21	2013
14,8	21,5	0,7	4	1	23	2006
14	18,2	0,7	3	1	25	2003
14	18,2	0,7	5	1	23	2007
14	18,2	0,7	4	1	15	2015
16,5	29,8	0,7	9	1	20	2000
11,8	10,9	0,7	2	1	22	1999
14,3	19,4	0,7	5	1	18	2000
14,3	19,4	0,7	2	1	21	2000
16,7	30,9	0,7	3	1	19	2000
15,2	23,3	0,7	5	1	19	2000

15,2	23,3	0,7	4	1	24	2009
14,1	18,6	0,7	5	1	22	2007
14,2	19	0,7	4	1	26	1999
14,2	19	0,7	4	1	18	2008
15	22,4	0,7	4	1	25	2003
15	22,4	0,7	5	1	17	2014
15,6	25,2	0,7	4	1	19	2012
13,6	16,7	0,7	2	1	22	2000
13,6	16,7	0,7	4	1	23	2009
12,7	13,6	0,7	2	1	20	2002
12,7	13,6	0,7	2	1	26	2009
17,1	33,2	0,7	5	1	20	2013
17,6	36,2	0,7	7	1	19	2012
16	27,2	0,7	8	1	16	2008
11,5	10,1	0,7	2	1	23	2004
15,7	25,7	0,7	5	1	19	2014
14,7	21,1	0,7	4	1	18	1999
14,7	21,1	0,7	6	1	21	1999
16,4	29,3	0,7	5	1	20	1999

17,3	34,4	0,7	5	1	19	2012
15,3	23,8	0,7	5	1	25	2004
13	14,6	0,7	2	1	19	2000
13	14,6	0,7	2	1	24	2002
13	14,6	0,7	3	1	23	2008
13	14,6	0,7	3	1	25	2008
16,6	30,4	0,7	5	1	22	2004
12,6	13,3	0,7	3	1	25	2007
13,4	16	0,7	3	1	21	2000
16,3	28,8	0,7	7	1	19	2012
13,7	17,1	0,7	3	1	26	2000
13,7	17,1	0,7	2	1	20	2002
13,7	17,1	0,7	2	1	24	2009
13,7	17,1	0,7	4	1	18	2015
16,9	32,1	0,7	5	1	21	2012
18,9	44,9	0,7	9	1	17	2012
18,9	44,9	0,7	6	1	20	2013
15,1	22,9	0,7	5	1	21	2000
15,1	22,9	0,7	4	1	23	2006

15,1	22,9	0,7	6	1	20	2013
14,6	20,7	0,7	4	1	21	2012
13,2	15,3	0,7	2	1	19	2000
11,3	9,6	0,7	2	1	23	2004
15,4	24,3	0,7	5	1	21	2000
15,4	24,3	0,7	6	1	18	2001
15,4	24,3	0,7	4	1	25	2002
15,4	24,3	0,7	4	1	21	2012
12	11,5	0,7	3	1	23	2005
12	11,5	0,7	2	1	23	2007
12	11,5	0,7	2	1	22	2011
12,5	13	0,7	2	1	23	2006
12,5	13	0,7	2	1	21	2011
16,5	29,9	0,7	5	1	18	1999
16,5	29,9	0,7	5	1	23	2010
16,5	29,9	0,7	5	1	23	2010
14,5	20,3	0,7	4	1	15	2015
13,8	17,5	0,7	3	1	22	2000
15,5	24,8	0,7	4	1	19	2002

12,1	11,8	0,7	2	1	25	2007
12,1	11,8	0,7	2	1	21	2014
12,1	11,8	0,7	2	1	23	2014
12,4	12,7	0,7	3	1	23	2005
12,4	12,7	0,7	2	1	20	2013
19,3	47,9	0,7	5	1	19	2013
17,3	34,5	0,7	5	1	19	2000
12,2	12,1	0,7	2	1	25	2003
12,2	12,1	0,7	2	1	22	2007
12,3	12,4	0,7	2	1	23	1999
12,3	12,4	0,7	2	1	25	2007
12,3	12,4	0,7	2	1	21	2008
14,4	19,9	0,7	4	1	23	2002
16	27,3	0,7	4	1	22	2000
16	27,3	0,7	6	1	23	2001
16	27,3	0,7	4	1	19	2012
16,4	29,4	0,7	5	1	20	2013
13,5	16,4	0,7	2	1	23	2002
15	22,5	0,7	5	1	22	1999

15	22,5	0,7	4	1	16	2015
15,7	25,8	0,7	5	1	19	2000
15,9	26,8	0,7	5	1	22	2001
15,9	26,8	0,7	4	1	22	2013
16,6	30,5	0,7	4	1	20	2013
15,8	26,3	0,7	6	1	22	2007
15,8	26,3	0,7	6	1	21	2013
14,3	19,5	0,7	5	1	18	1999
14,3	19,5	0,7	5	1	18	2000
14,3	19,5	0,7	2	1	25	2002
14,3	19,5	0,7	6	1	20	2011
14,3	19,5	0,7	3	1	21	2012
14,3	19,5	0,7	4	1	19	2012
14	18,3	0,7	3	1	26	2003
14	18,3	0,7	3	1	20	2007
14	18,3	0,7	5	1	25	2007
14	18,3	0,7	3	1	20	2011
14,2	19,1	0,7	4	1	22	1999
14,1	18,7	0,7	5	1	19	1999

14,1	18,7	0,7	3	1	23	2007
14,1	18,7	0,7	3	1	21	2014
14,1	18,7	0,7	3	1	23	2014
13,1	15	0,7	2	1	24	2001
13,1	15	0,7	3	1	25	2008
15,3	23,9	0,7	5	1	18	2007
15,3	23,9	0,7	4	1	22	2011
15,3	23,9	0,7	5	1	22	2013
16,3	28,9	0,7	5	1	22	2002
13,3	15,7	0,7	3	1	22	2000
13,3	15,7	0,7	2	1	22	2003
13,3	15,7	0,7	3	1	25	2005
13,3	15,7	0,7	3	1	23	2006
13,3	15,7	0,7	5	1	20	2013
14,7	21,2	0,7	4	1	18	1999
20	53,4	0,7	6	1	18	2012
12,8	14	0,7	3	1	22	2011
17	32,8	0,7	5	1	21	2014
24,3	95,8	0,7	9	1	15	2015

19	45,8	0,7	8	1	20	2013
17,9	38,3	0,7	4	1	18	2012
18,4	41,6	0,7	5	1	18	2012
16,5	30	0,7	9	1	22	2004
13,6	16,8	0,7	2	1	27	1999
13,6	16,8	0,7	4	1	21	2006
15,1	23	0,7	4	1	21	2006
11,7	10,7	0,7	2	1	23	1999
15,4	24,4	0,7	3	1	20	2003
15,4	24,4	0,7	4	1	20	2011
15,4	24,4	0,7	10	1	19	2012
14,9	22,1	0,7	5	1	25	1999
11,4	9,9	0,7	2	1	22	2011
17,3	34,6	0,7	5	1	21	2001
14,6	20,8	0,7	3	1	25	2004
14,6	20,8	0,7	7	1	22	2009
14,6	20,8	0,7	5	1	22	2011
18,2	40,3	0,7	8	1	19	2013
16,1	27,9	0,7	5	1	21	2013

16,1	27,9	0,7	7	1	19	2014
15,5	24,9	0,7	4	1	23	2011
15,5	24,9	0,7	3	1	22	2011
11	8,9	0,7	2	1	22	2004
11	8,9	0,7	2	1	22	2004
11	8,9	0,7	2	1	25	2004
11	8,9	0,7	1	1	26	2009
18	39	0,7	6	1	22	2013
20	53,5	0,7	7	1	17	2014
12,7	13,7	0,7	3	1	25	2005
12,7	13,7	0,7	2	1	17	2014
13,7	17,2	0,7	4	1	27	1999
13,7	17,2	0,7	4	1	25	2005
16	27,4	0,7	4	1	23	2002
16	27,4	0,7	6	1	20	2013
16,6	30,6	0,7	7	1	22	2002
16,6	30,6	0,7	5	1	17	2012
10,2	7,1	0,7	2	1	25	2004
13	14,7	0,7	3	1	23	2007

13	14,7	0,7	3	1	18	2014
13,4	16,1	0,7	2	1	21	2000
13,4	16,1	0,7	3	1	21	2008
13,4	16,1	0,7	3	1	25	2008
14,5	20,4	0,7	3	1	24	2001
14,5	20,4	0,7	3	1	22	2002
14,5	20,4	0,7	4	1	25	2005
14,5	20,4	0,7	5	1	22	2007
14,5	20,4	0,7	4	1	22	2011
15,2	23,5	0,7	3	1	26	2000
15,2	23,5	0,7	6	1	19	2001
15,7	25,9	0,7	3	1	20	2003
15,8	26,4	0,7	9	1	26	2001
15,8	26,4	0,7	4	1	21	2015
17,6	36,5	0,7	5	1	19	2010
17,6	36,5	0,7	5	1	19	2010
13,2	15,4	0,7	3	1	22	2000
13,2	15,4	0,7	5	1	26	2000
13,2	15,4	0,7	3	1	23	2005

13,2	15,4	0,7	3	1	25	2007
13,2	15,4	0,7	3	1	19	2008
13,2	15,4	0,7	3	1	23	2008
13,8	17,6	0,7	2	1	26	2001
13,8	17,6	0,7	3	1	21	2008
14,4	20	0,7	3	1	21	2000
14,4	20	0,7	5	1	26	2000
14,4	20	0,7	3	1	25	2003
12,6	13,4	0,7	2	1	26	2000
12,6	13,4	0,7	2	1	20	2002
12,6	13,4	0,7	2	1	23	2010
12,6	13,4	0,7	2	1	23	2010
17,1	33,5	0,7	6	1	21	2013
15,3	24	0,7	5	1	20	1999
15,3	24	0,7	5	1	21	1999
15,3	24	0,7	6	1	19	2008
14,3	19,6	0,7	6	1	25	1999
19,5	49,7	0,7	7	1	20	2013
16,2	28,5	0,7	6	1	19	2000

16,2	28,5	0,7	4	1	24	2002
14	18,4	0,7	2	1	26	2000
14	18,4	0,7	3	1	18	2002
14	18,4	0,7	6	1	21	2013
14	18,4	0,7	4	1	23	2015
14,2	19,2	0,7	4	1	26	1999
14,2	19,2	0,7	3	1	22	2003
13,5	16,5	0,7	2	1	25	2005
13,5	16,5	0,7	4	1	22	2011
11,5	10,2	0,7	2	1	25	2004
12,5	13,1	0,7	2	1	26	1999
12,5	13,1	0,7	2	1	20	2013
12,9	14,4	0,7	3	1	21	2008
15,4	24,5	0,7	4	1	20	2011
16,1	28	0,7	6	1	19	2011
16,1	28	0,7	4	1	22	2011
15,1	23,1	0,7	5	1	18	2007
15,1	23,1	0,7	4	1	22	2011
16,4	29,6	0,7	6	1	23	2001

14,9	22,2	0,7	5	1	18	2007
16,6	30,7	0,7	6	1	21	1999
16,6	30,7	0,7	4	1	15	2015
17,9	38,5	0,7	7	1	19	2012
12	11,6	0,7	2	1	26	2009
12,4	12,8	0,7	3	1	23	2004
12,4	12,8	0,7	2	1	24	2009
14,6	20,9	0,7	4	1	22	2002
13,3	15,8	0,7	3	1	19	2011
13,1	15,1	0,7	2	1	20	2000
13,1	15,1	0,7	3	1	19	2006
15,6	25,5	0,7	5	1	18	2000
15,6	25,5	0,7	5	1	22	2000
17	33	0,7	6	1	20	2013
15,9	27	0,7	5	1	22	2013
12,1	11,9	0,7	2	1	23	2005
12,3	12,5	0,7	3	1	26	1999
12,3	12,5	0,7	2	1	22	2011
15,7	26	0,7	5	1	19	1999

15,7	26	0,7	6	1	20	2000
16,3	29,1	0,7	5	1	19	2013
15,2	23,6	0,7	5	1	20	2000
15,2	23,6	0,7	5	1	23	2002
17,3	34,8	0,7	4	1	21	2012
11,3	9,7	0,7	2	1	22	2007
16,5	30,2	0,7	5	1	18	2000
16,5	30,2	0,7	7	1	19	2015
14,5	20,5	0,7	3	1	21	2008
14,5	20,5	0,7	3	1	23	2008
14,8	21,8	0,7	4	1	21	1999
14,8	21,8	0,7	4	1	27	1999
14,8	21,8	0,7	3	1	25	2007
14,8	21,8	0,7	5	1	25	2007
14,8	21,8	0,7	4	1	21	2012
15	22,7	0,7	3	1	19	2000
16,8	31,9	0,7	4	1	16	2015
16,8	31,9	0,7	4	1	16	2015
13,7	17,3	0,7	4	1	26	1999

13,7	17,3	0,7	7	1	22	2002
13,7	17,3	0,7	3	1	19	2015
15,3	24,1	0,7	4	1	22	2003
15,3	24,1	0,7	3	1	26	2003
15,3	24,1	0,7	4	1	22	2015
14,4	20,1	0,7	3	1	26	2005
14,4	20,1	0,7	4	1	19	2012
13,4	16,2	0,7	4	1	22	2002
16,9	32,5	0,7	6	1	19	2013
16,9	32,5	0,7	9	1	17	2014
16,1	28,1	0,7	6	1	20	2007
13,8	17,7	0,7	2	1	27	1999
13,8	17,7	0,7	2	1	27	1999
15,4	24,6	0,7	4	1	21	2000
13	14,8	0,7	3	1	20	2002
13	14,8	0,7	3	1	23	2005
14,3	19,7	0,7	2	1	27	1999
14,3	19,7	0,7	3	1	18	2012
14,3	19,7	0,7	2	1	21	2013

14,7	21,4	0,7	3	1	18	1999
12,7	13,8	0,7	2	1	19	2011
17	33,1	0,7	7	1	22	2013
16	27,6	0,7	5	1	21	1999
16	27,6	0,7	4	1	18	2001
15,1	23,2	0,7	3	1	20	2003
13,2	15,5	0,7	2	1	22	2002
13,2	15,5	0,7	3	1	23	2007
13,2	15,5	0,7	4	1	16	2008
13,2	15,5	0,7	2	1	18	2014
17,1	33,7	0,7	6	1	19	2012
15,5	25,1	0,7	5	1	19	2005
17,3	34,9	0,7	7	1	21	2014
14,2	19,3	0,7	2	1	24	2002
14,2	19,3	0,7	4	1	21	2006
14,2	19,3	0,7	3	1	23	2008
14,2	19,3	0,7	4	1	22	2011
14	18,5	0,7	5	1	23	1999
14	18,5	0,7	5	1	20	2010

14	18,5	0,7	5	1	20	2010
14,1	18,9	0,7	2	1	26	2003
14,1	18,9	0,7	4	1	18	2008
14,1	18,9	0,7	4	1	19	2008
11,7	10,8	0,7	2	1	22	2007
15,8	26,6	0,7	4	1	21	2013
15,7	26,1	0,7	5	1	21	1999
13,5	16,6	0,7	5	1	26	1999
13,5	16,6	0,7	2	1	22	2000
17,9	38,7	0,7	4	1	21	2012
14,6	21	0,7	4	1	21	2006
15,2	23,7	0,7	5	1	23	2006
15,2	23,7	0,7	4	1	15	2015
12,6	13,5	0,7	2	1	25	2007
16,2	28,7	0,7	5	1	24	2002
16,2	28,7	0,7	5	1	24	2002
16,2	28,7	0,7	5	1	19	2012
16,2	28,7	0,7	4	1	22	2013
16,9	32,6	0,7	4	1	18	2012

12,9	14,5	0,7	3	1	25	2007
14,8	21,9	0,7	5	1	21	2000
14,8	21,9	0,7	6	1	23	2008
15	22,8	0,7	3	1	23	2002
15	22,8	0,7	5	1	26	2006
15	22,8	0,7	5	1	19	2012
11,8	11,1	0,7	2	1	20	2002
16,4	29,8	0,7	6	1	23	2003
19,5	50,1	0,7	7	1	17	2012
15,3	24,2	0,7	5	1	21	2012
14,5	20,6	0,7	4	1	20	2011
13,6	17	0,7	3	1	19	2000
13,3	15,9	0,7	2	1	19	2013
13,3	15,9	0,7	2	1	18	2014
12,5	13,2	0,7	2	1	23	2002
12,5	13,2	0,7	3	1	21	2011
12,5	13,2	0,7	2	1	20	2013
17,1	33,8	0,7	6	1	20	2013
13,1	15,2	0,7	3	1	25	2004

13,1	15,2	0,7	2	1	20	2008
13,1	15,2	0,7	3	1	18	2014
11,2	9,5	0,7	2	1	23	2006
15,4	24,7	0,7	4	1	25	2002
19	46,4	0,7	5	1	21	2012
14,4	20,2	0,7	3	1	19	2002
14,4	20,2	0,7	3	1	22	2003
14,4	20,2	0,7	4	1	24	2009
14,4	20,2	0,7	4	1	22	2015
11,9	11,4	0,7	2	1	25	2007
12,4	12,9	0,7	2	1	22	2009
12,4	12,9	0,7	3	1	23	2009
15,9	27,2	0,7	4	1	20	1999
15,9	27,2	0,7	4	1	24	2002
15,9	27,2	0,7	4	1	20	2013
13,7	17,4	0,7	2	1	22	1999
15,5	25,2	0,7	3	1	23	2002
16,5	30,4	0,7	5	1	18	2014
15,1	23,3	0,7	4	1	20	2006

14,7	21,5	0,7	3	1	20	2003
15,6	25,7	0,7	4	1	18	2014
16,8	32,1	0,7	7	1	18	2001
15,7	26,2	0,7	5	1	26	2006
15,7	26,2	0,7	5	1	21	2013
12	11,7	0,7	2	1	23	2007
12	11,7	0,7	2	1	25	2007
12,3	12,6	0,7	2	1	19	2014
14,3	19,8	0,7	3	1	23	2003
12,8	14,2	0,7	2	1	20	2002
12,8	14,2	0,7	2	1	25	2007
12,8	14,2	0,7	2	1	20	2013
11,5	10,3	0,7	2	1	26	2001
11,5	10,3	0,7	1	1	26	2001
18	39,5	0,7	6	1	19	2008
13,8	17,8	0,7	3	1	23	1999
12,1	12	0,7	2	1	23	2005
12,1	12	0,7	2	1	25	2007
12,2	12,3	0,7	3	1	23	2005

12,2	12,3	0,7	2	1	23	2009
16,2	28,8	0,7	7	1	19	2012
13,4	16,3	0,7	4	1	22	1999
13,4	16,3	0,7	2	1	22	2000
13,4	16,3	0,7	2	1	21	2000
13,4	16,3	0,7	2	1	24	2002
13,4	16,3	0,7	3	1	25	2007
16,9	32,7	0,7	5	1	20	2013
14,2	19,4	0,7	6	1	26	1999
14,2	19,4	0,7	3	1	23	2008
14,2	19,4	0,7	3	1	18	2012
15,2	23,8	0,7	7	1	26	2006
14,1	19	0,7	4	1	21	1999
14,1	19	0,7	3	1	26	2003
14,1	19	0,7	2	1	21	2012
17	33,3	0,7	5	1	18	2000
14	18,6	0,7	5	1	19	2012
14,6	21,1	0,7	6	1	25	1999
17,2	34,5	0,7	6	1	23	1999

13	14,9	0,7	3	1	23	2003
13	14,9	0,7	3	1	18	2015
15,3	24,3	0,7	5	1	18	1999
15,3	24,3	0,7	4	1	22	2011
12,7	13,9	0,7	2	1	19	2000
12,7	13,9	0,7	3	1	25	2005
14,8	22	0,7	4	1	18	1999
21,6	68,4	0,7	7	1	18	2012
13,5	16,7	0,7	2	1	21	2000
13,5	16,7	0,7	3	1	25	2008
13,5	16,7	0,7	6	1	17	2014
18,3	41,6	0,7	6	1	19	2013
16,3	29,4	0,7	4	1	18	2000
14,5	20,7	0,7	5	1	27	1999
14,5	20,7	0,7	3	1	22	2000
14,5	20,7	0,7	8	1	23	2010
14,5	20,7	0,7	8	1	23	2010
18	39,6	0,7	5	1	18	2012
15,4	24,8	0,7	5	1	25	2003

15,4	24,8	0,7	4	1	22	2011
17,5	36,4	0,7	9	1	20	2011
17,5	36,4	0,7	7	1	17	2012
15,5	25,3	0,7	5	1	26	1999
15,5	25,3	0,7	9	1	23	2006
15,8	26,8	0,7	6	1	23	2006
16,9	32,8	0,7	3	1	17	2012
15,6	25,8	0,7	5	1	22	2000
15,7	26,3	0,7	4	1	25	2004
15,1	23,4	0,7	5	1	20	2000
15,1	23,4	0,7	3	1	18	2012
13,6	17,1	0,7	2	1	21	2000
13,6	17,1	0,7	5	1	26	2005
17	33,4	0,7	6	1	19	2011
17,3	35,2	0,7	4	1	21	2013
14,4	20,3	0,7	5	1	26	1999
14,4	20,3	0,7	5	1	27	1999
14,4	20,3	0,7	3	1	27	1999
14,4	20,3	0,7	5	1	22	2000

14,4	20,3	0,7	3	1	23	2003
12,6	13,6	0,7	2	1	25	1999
12,6	13,6	0,7	2	1	25	2007
17,1	34	0,7	4	1	18	2012
17,1	34	0,7	3	1	19	2012
17,2	34,6	0,7	7	1	18	2012
17,9	39	0,7	4	1	18	2012
20	54,4	0,7	10	1	18	2012
13,3	16	0,7	2	1	22	2000
13,3	16	0,7	3	1	23	2008
14,3	19,9	0,7	5	1	25	1999
14,3	19,9	0,7	5	1	26	2000
14,3	19,9	0,7	3	1	20	2003
14,3	19,9	0,7	5	1	17	2012
15,2	23,9	0,7	5	1	19	2012
15,2	23,9	0,7	5	1	20	2013
13,7	17,5	0,7	2	1	20	2000
13,7	17,5	0,7	3	1	21	2003
13,7	17,5	0,7	3	1	20	2008

13,1	15,3	0,7	3	1	26	2005
18	39,7	0,7	6	1	20	2013
12,5	13,3	0,7	2	1	22	2002
14,2	19,5	0,7	3	1	22	2000
14,2	19,5	0,7	3	1	22	2001
14,2	19,5	0,7	4	1	23	2006
14,2	19,5	0,7	3	1	18	2008
14,2	19,5	0,7	3	1	18	2014
13,8	17,9	0,7	2	1	24	2002
13,8	17,9	0,7	4	1	20	2011
13,8	17,9	0,7	4	1	21	2012
16	27,9	0,7	5	1	20	1999
16	27,9	0,7	7	1	22	1999
16	27,9	0,7	5	1	22	2013
16,3	29,5	0,7	6	1	19	2013
14,1	19,1	0,7	2	1	21	2000
13,9	18,3	0,7	5	1	26	1999
15	23	0,7	4	1	27	1999
15	23	0,7	3	1	26	2000

15	23	0,7	5	1	19	2012
15	23	0,7	3	1	20	2012
14	18,7	0,7	4	1	23	2006
14	18,7	0,7	3	1	25	2007
14	18,7	0,7	3	1	18	2015
13,4	16,4	0,7	2	1	24	2006
11,8	11,2	0,7	2	1	25	2004
11,8	11,2	0,7	2	1	19	2006
15,4	24,9	0,7	7	1	24	2001
15,4	24,9	0,7	3	1	22	2003
15,4	24,9	0,7	4	1	22	2004
17	33,5	0,7	6	1	23	2006
17	33,5	0,7	5	1	22	2013
12,8	14,3	0,7	2	1	22	2000
12,8	14,3	0,7	2	1	22	2000
12,8	14,3	0,7	2	1	26	2000
12,8	14,3	0,7	4	1	20	2004
18,3	41,8	0,7	6	1	19	2013
15,5	25,4	0,7	4	1	20	1999

15,5	25,4	0,7	7	1	27	1999
15,5	25,4	0,7	6	1	22	2011
15,5	25,4	0,7	4	1	16	2015
15,7	26,4	0,7	4	1	20	1999
15,7	26,4	0,7	3	1	19	2000
15,7	26,4	0,7	7	1	20	2001
15,7	26,4	0,7	4	1	19	2011
14,5	20,8	0,7	4	1	21	2008
18	39,8	0,7	5	1	20	2013
12,3	12,7	0,7	2	1	24	2002
12,3	12,7	0,7	2	1	18	2014
20	54,6	0,7	6	1	22	2013
15,1	23,5	0,7	4	1	21	2001
13	15	0,7	2	1	27	1999
13	15	0,7	5	1	25	2007
13	15	0,7	2	1	19	2013
23	83,1	0,7	10	1	18	2012
12,1	12,1	0,7	4	1	23	2005
12,1	12,1	0,7	2	1	17	2014

14,7	21,7	0,7	3	1	19	2011
14,4	20,4	0,7	2	1	27	1999
14,4	20,4	0,7	6	1	23	2008
17,4	36	0,7	5	1	18	2014
15,2	24	0,7	3	1	19	2012
16,5	30,7	0,7	5	1	21	2000
12,7	14	0,7	2	1	22	2002
12,7	14	0,7	3	1	25	2004
16,3	29,6	0,7	6	1	19	2014
16	28	0,7	7	1	19	2009
11	9,1	0,7	1	1	25	2007
13,6	17,2	0,7	3	1	17	2014
17,2	34,8	0,7	6	1	22	2013
14,3	20	0,7	3	1	23	2003
14,3	20	0,7	6	1	17	2008
14,3	20	0,7	3	1	21	2015
15,3	24,5	0,7	4	1	26	1999
15,3	24,5	0,7	5	1	26	2000
13,3	16,1	0,7	3	1	19	2009

13,3	16,1	0,7	2	1	21	2013
13,3	16,1	0,7	2	1	22	2013
14,6	21,3	0,7	8	1	17	2009
14,6	21,3	0,7	4	1	19	2012
14,6	21,3	0,7	3	1	16	2015
15	23,1	0,7	3	1	19	2012
16,2	29,1	0,7	7	1	21	1999
16,2	29,1	0,7	6	1	18	2000
16,2	29,1	0,7	4	1	20	2007
15,4	25	0,7	5	1	22	2013
10,4	7,7	0,7	1	1	26	2009
14,2	19,6	0,7	4	1	21	1999
14,2	19,6	0,7	5	1	25	1999
14,2	19,6	0,7	3	1	21	2008
14,2	19,6	0,7	3	1	20	2011
14,2	19,6	0,7	2	1	20	2013
14,2	19,6	0,7	3	1	18	2014
15,8	27	0,7	6	1	21	1999
15,8	27	0,7	4	1	20	2011

16,4	30,2	0,7	5	1	18	1999
16,4	30,2	0,7	7	1	20	1999
16,4	30,2	0,7	8	1	19	2015
15,7	26,5	0,7	5	1	22	1999
15,7	26,5	0,7	4	1	22	2011
12,9	14,7	0,7	3	1	21	2011
14,8	22,2	0,7	3	1	26	2003
12,6	13,7	0,7	2	1	25	2008
13,8	18	0,7	3	1	22	2005
16,7	31,9	0,7	5	1	18	2000
14,1	19,2	0,7	3	1	23	2006
14,1	19,2	0,7	5	1	25	2007
13,1	15,4	0,7	3	1	25	2005
13,1	15,4	0,7	3	1	25	2007
19,5	50,8	0,7	5	1	19	2012
13,9	18,4	0,7	4	1	21	1999
13,9	18,4	0,7	3	1	21	2012
14	18,8	0,7	3	1	20	2012
19,2	48,5	0,7	10	1	20	2013

11,6	10,7	0,7	2	1	22	2007
14,5	20,9	0,7	3	1	27	1999
16,3	29,7	0,7	5	1	20	2003
17,1	34,3	0,7	7	1	21	2012
12,5	13,4	0,7	2	1	23	2002
12,5	13,4	0,7	2	1	25	2005
12,5	13,4	0,7	2	1	20	2013
19,4	50,1	0,7	8	1	18	2012
15,2	24,1	0,7	5	1	23	2006
14,7	21,8	0,7	3	1	27	1999
14,7	21,8	0,7	3	1	23	2002
14,7	21,8	0,7	3	1	15	2015
16,6	31,4	0,7	7	1	19	2012
15,9	27,6	0,7	3	1	22	2001
12,8	14,4	0,7	5	1	25	1999
12,8	14,4	0,7	4	1	23	2006
12,8	14,4	0,7	3	1	21	2008
19	47,1	0,7	5	1	18	2012
16,2	29,2	0,7	6	1	23	2005

16,2	29,2	0,7	6	1	19	2012
16,2	29,2	0,7	4	1	19	2013
15,3	24,6	0,7	6	1	20	2013
13,5	16,9	0,7	3	1	23	2008
13,2	15,8	0,7	2	1	21	2008
13,2	15,8	0,7	2	1	21	2014
12,4	13,1	0,7	3	1	21	2005
15,7	26,6	0,7	5	1	22	1999
15,7	26,6	0,7	3	1	18	2001
15,7	26,6	0,7	6	1	20	2013
14,3	20,1	0,7	3	1	23	1999
14,3	20,1	0,7	3	1	20	2008
14,3	20,1	0,7	3	1	21	2015
15	23,2	0,7	5	1	21	2000
15,5	25,6	0,7	5	1	20	2009
18	40,1	0,7	4	1	21	2012
14,6	21,4	0,7	3	1	26	2000
14,6	21,4	0,7	4	1	23	2008
13,6	17,3	0,7	2	1	21	2000

11,8	11,3	0,7	1	1	26	2001
17,2	35	0,7	6	1	22	2013
20,8	61,9	0,7	5	1	18	2012
16,5	30,9	0,7	7	1	23	2001
16,5	30,9	0,7	5	1	19	2012
14,8	22,3	0,7	10	1	26	2006
14,2	19,7	0,7	3	1	27	1999
16,3	29,8	0,7	4	1	18	1999
19	47,2	0,7	6	1	20	2013
13,7	17,7	0,7	3	1	26	2000
15,1	23,7	0,7	5	1	19	2013
12,2	12,5	0,7	2	1	25	2007
16	28,2	0,7	6	1	19	2011
14,1	19,3	0,7	5	1	23	2007
13,3	16,2	0,7	2	1	24	2002
16,6	31,5	0,7	8	1	23	2008
16,6	31,5	0,7	5	1	20	2013
12,1	12,2	0,7	2	1	22	2007
12,1	12,2	0,7	2	1	25	2007

13,8	18,1	0,7	4	1	23	2008
14	18,9	0,7	2	1	19	2001
14	18,9	0,7	3	1	26	2003
14	18,9	0,7	5	1	26	2005
14	18,9	0,7	4	1	21	2011
14,5	21	0,7	3	1	27	1999
13,9	18,5	0,7	2	1	24	2002
13,9	18,5	0,7	3	1	21	2011
17,4	36,3	0,7	10	1	21	2012
15,2	24,2	0,7	4	1	24	2002
16,7	32,1	0,7	5	1	22	2003
16,7	32,1	0,7	6	1	20	2011
13,1	15,5	0,7	3	1	19	2000
13,1	15,5	0,7	3	1	21	2000
13,1	15,5	0,7	4	1	23	2005
13,1	15,5	0,7	3	1	25	2007
13,1	15,5	0,7	4	1	20	2011
15,3	24,7	0,7	4	1	27	1999
15,3	24,7	0,7	3	1	20	2003

12,6	13,8	0,7	2	1	22	2002
14,4	20,6	0,7	2	1	26	2000
13,4	16,6	0,7	4	1	22	2001
15,7	26,7	0,7	5	1	19	2000
17,1	34,5	0,7	6	1	22	2013
20	55,2	0,7	6	1	19	2010
20	55,2	0,7	6	1	19	2010
17	33,9	0,7	5	1	22	2013
16,1	28,8	0,7	6	1	20	2013
15,6	26,2	0,7	3	1	20	2003
15	23,3	0,7	5	1	25	1999
15	23,3	0,7	5	1	27	1999
15	23,3	0,7	4	1	21	2012
16,3	29,9	0,7	5	1	21	2000
17,9	39,6	0,7	9	1	20	2011
14,3	20,2	0,7	4	1	21	1999
14,3	20,2	0,7	4	1	25	1999
14,3	20,2	0,7	3	1	21	2000
14,3	20,2	0,7	5	1	26	2000

14,3	20,2	0,7	4	1	24	2006
14,3	20,2	0,7	6	1	18	2008
14,3	20,2	0,7	4	1	19	2008
14,3	20,2	0,7	4	1	20	2009
14,6	21,5	0,7	4	1	22	2011
16	28,3	0,7	6	1	19	2000
16	28,3	0,7	6	1	20	2013
13,5	17	0,7	2	1	22	2000
13,5	17	0,7	4	1	18	2012
19	47,4	0,7	6	1	18	2012
11	9,2	0,7	2	1	25	2007
15,1	23,8	0,7	7	1	18	1999
15,1	23,8	0,7	2	1	26	2000
13,2	15,9	0,7	3	1	23	2008
14,2	19,8	0,7	5	1	19	2012
17,6	37,7	0,7	5	1	17	2012
13,6	17,4	0,7	2	1	27	1999
13,6	17,4	0,7	3	1	23	2008
13	15,2	0,7	2	1	23	1999

13	15,2	0,7	2	1	25	2002
13	15,2	0,7	3	1	21	2011
14,1	19,4	0,7	4	1	26	1999
14,1	19,4	0,7	4	1	24	2006
14,5	21,1	0,7	5	1	21	2000
14,5	21,1	0,7	4	1	17	2012
14,5	21,1	0,7	3	1	22	2015
15,8	27,3	0,7	4	1	19	2012
13,7	17,8	0,7	3	1	22	1999
13,7	17,8	0,7	2	1	26	2000
12,4	13,2	0,7	2	1	25	2008
14	19	0,7	2	1	27	1999
15,3	24,8	0,7	4	1	18	1999
15,3	24,8	0,7	3	1	19	2012
13,8	18,2	0,7	3	1	25	2008
15,5	25,8	0,7	6	1	18	2001
15,5	25,8	0,7	6	1	18	2008
15,5	25,8	0,7	4	1	20	2013
11,3	10	0,7	2	1	23	2004

11,3	10	0,7	2	1	23	2004
11,3	10	0,7	2	1	23	2007
12,3	12,9	0,7	2	1	23	2009
12,7	14,2	0,7	2	1	22	2000
12,7	14,2	0,7	2	1	23	2002
12,7	14,2	0,7	2	1	19	2006
14,4	20,7	0,7	3	1	20	2000
14,4	20,7	0,7	3	1	22	2003
15	23,4	0,7	3	1	17	2008
10,4	7,8	0,7	2	1	23	2004
18,4	43,2	0,7	8	1	22	2013
17,2	35,3	0,7	6	1	19	2013
11,8	11,4	0,7	1	1	26	2001
11,8	11,4	0,7	2	1	19	2014
16,2	29,5	0,7	8	1	19	2012
12,2	12,6	0,7	2	1	17	2014
13,1	15,6	0,7	4	1	24	2006
13,1	15,6	0,7	5	1	25	2007
14,8	22,5	0,7	4	1	27	1999

14,8	22,5	0,7	8	1	20	2010
14,8	22,5	0,7	8	1	20	2010
13,4	16,7	0,7	2	1	21	2000
13,4	16,7	0,7	3	1	22	2004
17,5	37,2	0,7	6	1	19	2013
15,1	23,9	0,7	4	1	19	2009
14,3	20,3	0,7	3	1	24	2002
12,1	12,3	0,7	3	1	23	2005
12,1	12,3	0,7	2	1	19	2006
12	12	0,7	3	1	25	2005
16,5	31,2	0,7	5	1	18	2001
15,8	27,4	0,7	4	1	24	2002
12,6	13,9	0,7	4	1	25	2005
12,6	13,9	0,7	3	1	26	2005
12,6	13,9	0,7	2	1	24	2009
16,1	29	0,7	6	1	20	2011
16,1	29	0,7	6	1	19	2012
13,5	17,1	0,7	2	1	26	1999
13,5	17,1	0,7	3	1	25	2003

15,7	26,9	0,7	3	1	19	2013
16,6	31,8	0,7	8	1	22	2007
14,5	21,2	0,7	4	1	23	2003
14,5	21,2	0,7	3	1	19	2013
14,5	21,2	0,7	3	1	20	2013
19	47,7	0,7	6	1	19	2013
15,4	25,4	0,7	5	1	19	2000
15,5	25,9	0,7	5	1	20	1999
14,1	19,5	0,7	4	1	19	2006
16,7	32,4	0,7	5	1	19	2012
13,6	17,5	0,7	2	1	22	2000
16	28,5	0,7	5	1	22	2003
16	28,5	0,7	10	1	20	2007
16	28,5	0,7	5	1	21	2013
16,4	30,7	0,7	5	1	18	1999
14	19,1	0,7	4	1	23	2008
13,7	17,9	0,7	5	1	26	1999
16,2	29,6	0,7	4	1	20	2001
15	23,5	0,7	10	1	21	2003

15	23,5	0,7	5	1	19	2013
13,9	18,7	0,7	3	1	20	1999
12,5	13,6	0,7	2	1	25	2007
12,5	13,6	0,7	3	1	20	2011
12,5	13,6	0,7	2	1	19	2013
13,8	18,3	0,7	3	1	25	2008
13,8	18,3	0,7	4	1	22	2011
13	15,3	0,7	2	1	25	2002
13	15,3	0,7	2	1	23	2002
10,6	8,3	0,7	2	1	25	2004
10,6	8,3	0,7	2	1	25	2004
15,1	24	0,7	3	1	15	2015
17,3	36,1	0,7	6	1	20	2013
14,6	21,7	0,7	4	1	21	2012
16,1	29,1	0,7	8	1	20	2007
16,6	31,9	0,7	5	1	19	2012
12,4	13,3	0,7	2	1	21	2006
12,4	13,3	0,7	2	1	25	2007
14,3	20,4	0,7	5	1	23	2007

15,2	24,5	0,7	6	1	18	2000
17,7	38,7	0,7	4	1	21	2015
17,1	34,9	0,7	9	1	22	1999
15,3	25	0,7	5	1	18	2000
12,7	14,3	0,7	2	1	20	2002
15,4	25,5	0,7	5	1	22	1999
15,5	26	0,7	7	1	21	1999
15,5	26	0,7	5	1	22	1999
15,5	26	0,7	4	1	20	2013
13,4	16,8	0,7	2	1	26	2000
13,4	16,8	0,7	3	1	22	2002
13,4	16,8	0,7	2	1	23	2002
13,4	16,8	0,7	2	1	20	2013
16,4	30,8	0,7	8	1	25	2005
13,1	15,7	0,7	2	1	21	2000
13,1	15,7	0,7	3	1	19	2014
14,2	20	0,7	3	1	18	2012
16,2	29,7	0,7	4	1	15	2015
12,3	13	0,7	2	1	25	2007

14,5	21,3	0,7	5	1	27	1999
14,5	21,3	0,7	4	1	21	2012
14,5	21,3	0,7	4	1	23	2015
11	9,3	0,7	2	1	25	2004
17,6	38,1	0,7	6	1	21	2013
18,7	45,7	0,7	8	1	19	2013
14,7	22,2	0,7	5	1	22	1999
13,5	17,2	0,7	4	1	22	1999
17,9	40,1	0,7	6	1	21	2013
11,7	11,2	0,7	2	1	23	2004
12,2	12,7	0,7	2	1	23	2008
16,3	30,3	0,7	3	1	18	2012
16,3	30,3	0,7	4	1	18	2012
17,5	37,5	0,7	6	1	20	2003
10,5	8,1	0,7	1	1	22	2007
14	19,2	0,7	3	1	18	2008
12,6	14	0,7	2	1	25	1999
12,6	14	0,7	2	1	23	2002
12,1	12,4	0,7	2	1	23	2007

15,1	24,1	0,7	5	1	23	1999
15,1	24,1	0,7	4	1	21	2000
15,1	24,1	0,7	3	1	19	2012
13,2	16,1	0,7	3	1	20	2002
13,2	16,1	0,7	2	1	24	2002
13,2	16,1	0,7	3	1	25	2007
13,7	18	0,7	2	1	27	1999
11,9	11,8	0,7	3	1	23	2005
12	12,1	0,7	2	1	25	2007
16,9	33,8	0,7	7	1	22	1999
17,4	36,9	0,7	6	1	20	2013
14,6	21,8	0,7	3	1	15	2015
16,4	30,9	0,7	6	1	23	2006
16	28,7	0,7	5	1	19	2012
15,3	25,1	0,7	4	1	20	2006
15,4	25,6	0,7	4	1	23	2003
13	15,4	0,7	2	1	24	2002
13	15,4	0,7	2	1	22	2013
17,3	36,3	0,7	5	1	19	2013

16,5	31,5	0,7	6	1	21	1999
16,5	31,5	0,7	6	1	19	2011
13,3	16,5	0,7	3	1	21	2000
18,3	43	0,7	6	1	19	2013
11,1	9,6	0,7	2	1	21	2014
14,5	21,4	0,7	5	1	20	2007
14,2	20,1	0,7	4	1	23	2006
14,2	20,1	0,7	3	1	19	2013
14,7	22,3	0,7	5	1	27	1999
14,7	22,3	0,7	3	1	22	2003
16,7	32,7	0,7	4	1	20	2003
17	34,5	0,7	7	1	23	2002
20	56,2	0,7	10	1	21	2013
21,3	67,9	0,7	6	1	20	2013
12,4	13,4	0,7	3	1	20	2000
12,4	13,4	0,7	4	1	19	2006
13,1	15,8	0,7	5	1	25	2007
12,7	14,4	0,7	2	1	19	2013
13,5	17,3	0,7	2	1	21	2000

13,5	17,3	0,7	4	1	22	2005
13,5	17,3	0,7	4	1	24	2009
16,2	29,9	0,7	5	1	20	2003
16,2	29,9	0,7	7	1	19	2012
15,6	26,7	0,7	6	1	24	2006
15,6	26,7	0,7	3	1	16	2008
17,7	39	0,7	5	1	19	2000
14,8	22,8	0,7	4	1	22	2011
15,2	24,7	0,7	5	1	19	2012
17,5	37,7	0,7	6	1	18	2000
15,3	25,2	0,7	5	1	27	1999
13,6	17,7	0,7	2	1	27	1999
15,4	25,7	0,7	5	1	24	2001
15,4	25,7	0,7	5	1	24	2002
13,9	18,9	0,7	5	1	23	1999
12,3	13,1	0,7	2	1	25	2007
18,6	45,3	0,7	5	1	18	2012
15,9	28,3	0,7	5	1	19	2000
16,7	32,8	0,7	5	1	22	2001

17	34,6	0,7	10	1	19	2007
17	34,6	0,7	8	1	22	2011
13,2	16,2	0,7	2	1	24	2002
13,2	16,2	0,7	3	1	15	2015
14,3	20,6	0,7	3	1	23	2003
14,3	20,6	0,7	4	1	25	2005
14,3	20,6	0,7	3	1	25	2008
10,3	7,7	0,7	1	1	22	2007
12,2	12,8	0,7	2	1	17	2014
18,7	46,1	0,7	4	1	19	2012
19	48,36	0,7	8	1	20	2013
14,7	22,4	0,7	4	1	20	1999
15	23,8	0,7	3	1	25	2002
14,5	21,5	0,7	4	1	22	2011
14,5	21,5	0,7	4	1	22	2013
13	15,5	0,7	2	1	26	1999
13	15,5	0,7	3	1	25	2007
11,7	11,3	0,7	2	1	20	2009
16	28,9	0,7	4	1	20	2011

13,3	16,6	0,7	2	1	22	2002
12,1	12,5	0,7	3	1	23	2008
12,1	12,5	0,7	2	1	23	2009
16,2	30	0,7	7	1	21	1999
16,5	31,7	0,7	5	1	21	2000
15,1	24,3	0,7	4	1	23	2008
11,8	11,6	0,7	2	1	25	2004
12	12,2	0,7	2	1	23	2009
16,7	32,9	0,7	10	1	20	2003
14,8	22,9	0,7	5	1	22	2002
15,4	25,8	0,7	4	1	22	2011
15,4	25,8	0,7	4	1	21	2013
16,9	34,1	0,7	8	1	25	1999
13,4	17	0,7	2	1	26	2000
13,4	17	0,7	3	1	26	2005
13,4	17	0,7	4	1	24	2009
12,5	13,8	0,7	5	1	26	2006
10,2	7,5	0,7	1	1	18	2007
20,7	62,7	0,7	10	1	22	2013

14	19,4	0,7	4	1	27	1999
14	19,4	0,7	2	1	24	2002
14	19,4	0,7	3	1	24	2006
19	48,5	0,7	7	1	20	2013
17,5	37,9	0,7	4	1	18	2012
13,1	15,9	0,7	2	1	26	2005
13,7	18,2	0,7	3	1	25	2005
13,7	18,2	0,7	3	1	25	2008
13,7	18,2	0,7	3	1	15	2015
12,7	14,5	0,7	2	1	22	2000
16	29	0,7	5	1	22	2000
15,7	27,4	0,7	4	1	20	2003
15	23,9	0,7	5	1	22	1999
15	23,9	0,7	5	1	20	2011
15	23,9	0,7	5	1	19	2012
16,6	32,4	0,7	6	1	19	2000
15,6	26,9	0,7	7	1	22	2001
15,1	24,4	0,7	4	1	27	1999
14,2	20,3	0,7	3	1	26	2000

15,9	28,5	0,7	5	1	18	2008
15,2	24,9	0,7	7	1	25	2008
15,2	24,9	0,7	8	1	19	2012
15,4	25,9	0,7	4	1	27	1999
17,8	40	0,7	5	1	19	2000
11,1	9,7	0,7	2	1	25	2004
12,3	13,2	0,7	2	1	26	2000
17,2	36,1	0,7	8	1	19	2013
21,2	67,6	0,7	6	1	18	2012
16,4	31,3	0,7	6	1	19	2011
13,3	16,7	0,7	4	1	23	2006
13,3	16,7	0,7	2	1	19	2012
12,6	14,2	0,7	3	1	21	2011
14,1	19,9	0,7	3	1	20	2011
17,1	35,5	0,7	7	1	18	2012
17,1	35,5	0,7	6	1	20	2013
14,4	21,2	0,7	4	1	21	1999
14,4	21,2	0,7	3	1	23	2003
13	15,6	0,7	4	1	23	2005

16,2	30,2	0,7	7	1	21	1999
16,2	30,2	0,7	7	1	18	2012
12,2	12,9	0,7	2	1	24	2002
16	29,1	0,7	5	1	18	1999
16	29,1	0,7	5	1	20	1999
16	29,1	0,7	5	1	18	2000
19,2	50,3	0,7	5	1	18	2012
17,8	40,1	0,7	6	1	19	2000
17,8	40,1	0,7	5	1	19	2013
16,3	30,8	0,7	6	1	22	2003
12,1	12,6	0,7	2	1	17	2014
13,5	17,5	0,7	2	1	21	2000
14,7	22,6	0,7	7	1	22	1999
14,7	22,6	0,7	3	1	21	2000
15,9	28,6	0,7	5	1	19	2012
15,9	28,6	0,7	6	1	20	2013
13,8	18,7	0,7	4	1	23	2008
15,5	26,5	0,7	5	1	18	2000
15,5	26,5	0,7	5	1	26	2000

16,1	29,7	0,7	6	1	20	2011
12,5	13,9	0,7	2	1	23	2003
12,5	13,9	0,7	4	1	25	2008
12,5	13,9	0,7	3	1	20	2013
13,7	18,3	0,7	2	1	26	2000
13,1	16	0,7	3	1	25	2007
13,1	16	0,7	3	1	25	2008
16,4	31,4	0,7	10	1	23	2008
11,8	11,7	0,7	3	1	25	2004
18,5	45,1	0,7	6	1	19	2013
15,8	28,1	0,7	5	1	19	2012
14,2	20,4	0,7	5	1	25	1999
16,2	30,3	0,7	10	1	19	2011
12,7	14,6	0,7	2	1	26	2001
12,7	14,6	0,7	2	1	22	2002
12,7	14,6	0,7	3	1	25	2005
16,7	33,2	0,7	7	1	22	2002
16	29,2	0,7	6	1	19	2013
13,2	16,4	0,7	6	1	23	2008

19,9	56,2	0,7	5	1	17	2012
12,4	13,6	0,7	2	1	22	2002
14,4	21,3	0,7	4	1	20	2009
19,5	52,9	0,7	5	1	19	2012
14,1	20	0,7	6	1	23	2008
15,6	27,1	0,7	6	1	19	1999
15,6	27,1	0,7	5	1	21	1999
15,6	27,1	0,7	6	1	20	2000
17,1	35,7	0,7	10	1	21	2013
15,9	28,7	0,7	5	1	21	2012
16,1	29,8	0,7	5	1	19	2012
13,3	16,8	0,7	2	1	26	2000
18,4	44,5	0,7	9	1	18	2012
15,1	24,6	0,7	4	1	20	2011
17,3	37	0,7	6	1	16	2008
15,4	26,1	0,7	6	1	21	1999
15,4	26,1	0,7	3	1	19	2013
12,3	13,3	0,7	2	1	18	2014
14,3	20,9	0,7	4	1	27	1999

14,3	20,9	0,7	2	1	26	2000
14,3	20,9	0,7	2	1	26	2000
14,3	20,9	0,7	4	1	18	2012
15,2	25,1	0,7	6	1	25	1999
15,3	25,6	0,7	5	1	18	2012
15,3	25,6	0,7	6	1	19	2013
19,2	50,6	0,7	7	1	18	2012
13,9	19,2	0,7	2	1	27	1999
13,9	19,2	0,7	2	1	21	2000
15,8	28,2	0,7	4	1	19	2012
18	41,7	0,7	7	1	17	2012
18	41,7	0,7	5	1	22	2013
14,5	21,8	0,7	5	1	19	2012
13,5	17,6	0,7	3	1	26	2005
13,5	17,6	0,7	3	1	19	2011
13,7	18,4	0,7	2	1	24	2002
13,7	18,4	0,7	2	1	25	2005
15,7	27,7	0,7	7	1	20	2006
12,2	13	0,7	2	1	25	2002

12,2	13	0,7	2	1	25	2007
12,2	13	0,7	2	1	20	2009
14,2	20,5	0,7	3	1	22	2000
13,1	16,1	0,7	3	1	25	2007
14,9	23,7	0,7	4	1	20	2011
14,9	23,7	0,7	7	1	19	2012
17,5	38,4	0,7	6	1	19	2013
14,4	21,4	0,7	5	1	27	1999
16,5	32,2	0,7	6	1	19	2000
12,1	12,7	0,7	2	1	26	2005
15	24,2	0,7	5	1	26	2000
12,9	15,4	0,7	2	1	24	2002
13,2	16,5	0,7	3	1	21	2008
15,1	24,7	0,7	5	1	20	1999
15,3	25,7	0,7	9	1	26	1999
14	19,7	0,7	3	1	21	2008
16,3	31,1	0,7	6	1	21	2013
18,2	43,3	0,7	8	1	22	2013
13,3	16,9	0,7	2	1	19	2000

13,3	16,9	0,7	3	1	17	2014
12,4	13,7	0,7	3	1	25	2005
13,4	17,3	0,7	4	1	23	2002
13,4	17,3	0,7	3	1	20	2011
16,8	34,1	0,7	5	1	18	2001
13,5	17,7	0,7	3	1	25	2007
13,5	17,7	0,7	3	1	15	2015
17,4	37,9	0,7	5	1	19	2000
13,6	18,1	0,7	4	1	21	2012
16,2	30,6	0,7	5	1	19	2012
14,6	22,4	0,7	6	1	27	1999
12,8	15,1	0,7	2	1	22	2000
15,8	28,4	0,7	5	1	19	2012
14,4	21,5	0,7	5	1	27	1999
14,4	21,5	0,7	6	1	19	2000
12,3	13,4	0,7	2	1	22	2000
12,3	13,4	0,7	2	1	19	2008
12,3	13,4	0,7	2	1	23	2008
15,2	25,3	0,7	4	1	27	1999

15,2	25,3	0,7	4	1	23	2008
17	35,4	0,7	6	1	22	2002
14,1	20,2	0,7	3	1	25	2002
14,7	22,9	0,7	3	1	23	2003
14,7	22,9	0,7	4	1	21	2006
15,7	27,9	0,7	7	1	23	1999
15,7	27,9	0,7	4	1	22	2011
15,7	27,9	0,7	6	1	19	2013
16,1	30,1	0,7	6	1	22	2013
12,2	13,1	0,7	3	1	25	2007
14,3	21,1	0,7	3	1	21	2000
14	19,8	0,7	3	1	25	2002
14,5	22	0,7	8	1	26	2001
14,5	22	0,7	5	1	18	2014
13,2	16,6	0,7	4	1	21	2012
14,8	23,4	0,7	3	1	26	2001
14,8	23,4	0,7	3	1	22	2015
19,1	50,3	0,7	10	1	20	2013
16,2	30,7	0,7	8	1	18	2008

17,3	37,4	0,7	6	1	22	2003
15,5	26,9	0,7	3	1	19	2011
15,8	28,5	0,7	5	1	18	2012
13,3	17	0,7	3	1	22	2002
21,9	75,9	0,7	9	1	18	2012
13,8	19	0,7	2	1	26	2000
15,3	25,9	0,7	5	1	19	2000
13,4	17,4	0,7	6	1	22	2011
18,5	45,8	0,7	9	1	23	2002
13,7	18,6	0,7	4	1	26	2000
14,4	21,6	0,7	3	1	25	2008
12	12,5	0,7	2	1	22	2002
12	12,5	0,7	2	1	19	2013
13,5	17,8	0,7	3	1	22	2002
13,5	17,8	0,7	3	1	25	2002
13,6	18,2	0,7	2	1	22	2002
15,7	28	0,7	5	1	25	2005
16,7	33,7	0,7	6	1	20	2013
18	42,2	0,7	6	1	20	2013

13	15,9	0,7	2	1	25	2002
12,4	13,8	0,7	2	1	22	2002
12,4	13,8	0,7	2	1	23	2002
11,6	11,3	0,7	2	1	25	2007
11,9	12,2	0,7	2	1	25	2007
14,7	23	0,7	3	1	23	2003
14,1	20,3	0,7	4	1	23	1999
17,3	37,5	0,7	4	1	18	2012
11,8	11,9	0,7	2	1	20	2009
17,6	39,5	0,7	6	1	22	2013
17	35,6	0,7	6	1	23	2006
14,3	21,2	0,7	6	1	23	2008
15,8	28,6	0,7	6	1	21	2003
14	19,9	0,7	7	1	25	1999
14	19,9	0,7	2	1	21	2000
18	42,3	0,7	7	1	20	2013
12,3	13,5	0,7	2	1	22	2002
12,3	13,5	0,7	2	1	20	2009
15,4	26,5	0,7	8	1	26	2006

16,6	33,2	0,7	7	1	24	2006
15	24,5	0,7	3	1	16	2008
15,3	26	0,7	6	1	27	1999
15,3	26	0,7	4	1	18	2012
17,1	36,3	0,7	8	1	22	2013
13,2	16,7	0,7	4	1	26	2005
13,2	16,7	0,7	3	1	23	2008
15,1	25	0,7	3	1	25	2002
15,2	25,5	0,7	4	1	22	2011
14,6	22,6	0,7	3	1	19	2012
13,7	18,7	0,7	3	1	27	1999
13,7	18,7	0,7	2	1	21	2000
13,7	18,7	0,7	2	1	21	2000
13,7	18,7	0,7	4	1	22	2002
13,7	18,7	0,7	2	1	24	2002
16,3	31,5	0,7	6	1	21	2003
12,7	14,9	0,7	2	1	26	2000
12,7	14,9	0,7	3	1	25	2005
12,7	14,9	0,7	2	1	25	2008

13,5	17,9	0,7	2	1	26	2000
13,5	17,9	0,7	3	1	22	2002
16,8	34,5	0,7	5	1	18	2000
14,1	20,4	0,7	5	1	25	1999
20,5	62,7	0,7	10	1	18	2012
16,5	32,7	0,7	5	1	19	2012
10,1	7,5	0,7	1	1	26	2009
17,3	37,7	0,7	9	1	19	2011
12,1	12,9	0,7	2	1	25	1999
14,5	22,2	0,7	3	1	26	2000
13	16	0,7	2	1	21	2000
15,4	26,6	0,7	3	1	22	2002
15,4	26,6	0,7	5	1	20	2012
14,3	21,3	0,7	5	1	22	2000
14	20	0,7	4	1	22	2011
15	24,6	0,7	5	1	27	1999
12,4	13,9	0,7	2	1	24	2009
12	12,6	0,7	2	1	21	2013
18,2	44	0,7	8	1	18	2012

12,6	14,6	0,7	2	1	25	2002
14,7	23,2	0,7	3	1	15	2015
13,2	16,8	0,7	3	1	25	2003
18	42,6	0,7	10	1	18	2012
13,8	19,2	0,7	4	1	22	2002
17	35,9	0,7	7	1	19	2012
13,3	17,2	0,7	3	1	23	2008
13,7	18,8	0,7	2	1	24	2002
13,7	18,8	0,7	2	1	18	2012
16,9	35,3	0,7	8	1	19	2013
12,9	15,7	0,7	3	1	26	2005
13,4	17,6	0,7	2	1	22	2002
13,4	17,6	0,7	2	1	24	2002
15,3	26,2	0,7	5	1	21	2001
14,9	24,2	0,7	4	1	22	2002
14,9	24,2	0,7	3	1	25	2002
13,5	18	0,7	2	1	24	2002
15,2	25,7	0,7	5	1	26	2000
15	24,7	0,7	4	1	26	1999

15	24,7	0,7	7	1	18	2012
12,5	14,3	0,7	2	1	21	2000
12,7	15	0,7	2	1	26	1999
16,6	33,5	0,7	6	1	19	2013
15,5	27,3	0,7	4	1	20	2013
18,2	44,2	0,7	6	1	18	2012
14,2	21	0,7	5	1	21	2012
14,4	21,9	0,7	4	1	26	2000
14,4	21,9	0,7	3	1	25	2008
14,4	21,9	0,7	3	1	25	2008
19,5	54,4	0,7	8	1	22	2013
15,9	29,5	0,7	6	1	19	2013
13,1	16,5	0,7	3	1	25	2008
15,3	26,3	0,7	4	1	20	2013
12,8	15,4	0,7	2	1	26	1999
12,8	15,4	0,7	2	1	25	2008
13,8	19,3	0,7	3	1	22	2000
15,2	25,8	0,7	5	1	19	2012
17	36,1	0,7	6	1	20	2013

13,2	16,9	0,7	4	1	25	2005
12,6	14,7	0,7	3	1	25	1999
16	30,1	0,7	10	1	23	2008
14,1	20,6	0,7	5	1	22	2002
14,1	20,6	0,7	4	1	25	2007
17,2	37,4	0,7	6	1	21	2001
13,7	18,9	0,7	3	1	23	2003
14,3	21,5	0,7	3	1	17	2008
13,6	18,5	0,7	5	1	22	2000
13,6	18,5	0,7	4	1	26	2005
18	42,9	0,7	8	1	21	2013
18	42,9	0,7	6	1	19	2013
13,4	17,7	0,7	4	1	25	2008
17,7	40,8	0,7	5	1	20	2013
15,5	27,4	0,7	7	1	21	2012
14,6	22,9	0,7	4	1	22	2000
14,6	22,9	0,7	5	1	21	2003
15,9	29,6	0,7	5	1	21	2012
23,2	92	0,7	8	1	18	2012

14,2	21,1	0,7	4	1	21	2000
14,2	21,1	0,7	2	1	25	2002
14,2	21,1	0,7	5	1	19	2012
15,3	26,4	0,7	5	1	21	2012
18	43	0,7	4	1	21	2012
15,2	25,9	0,7	5	1	21	2000
15,6	28	0,7	5	1	23	1999
17,7	40,9	0,7	5	1	19	2000
19	50,6	0,7	6	1	15	2015
10,4	8,3	0,7	2	1	23	2004
13,1	16,6	0,7	5	1	25	2007
14,3	21,6	0,7	5	1	23	1999
14,3	21,6	0,7	3	1	19	2012
16,5	33,2	0,7	6	1	22	2013
12,8	15,5	0,7	2	1	25	2002
13,2	17	0,7	2	1	21	2000
17,8	41,7	0,7	8	1	20	2013
12,4	14,1	0,7	4	1	25	1999
12,4	14,1	0,7	3	1	23	2005

13,3	17,4	0,7	3	1	23	1999
13,4	17,8	0,7	3	1	25	2008
11,2	10,4	0,7	2	1	25	2004
17,6	40,4	0,7	6	1	21	2013
14,5	22,6	0,7	3	1	22	2003
12,3	13,8	0,7	3	1	26	2005
15,7	28,7	0,7	5	1	26	2000
13	16,3	0,7	2	1	25	2002
16	30,4	0,7	5	1	19	2012
14,7	23,6	0,7	4	1	27	1999
15,2	26,1	0,7	3	1	26	2000
13,6	18,7	0,7	4	1	26	1999
19,1	51,8	0,7	5	1	19	2013
14	20,4	0,7	3	1	26	1999
14	20,4	0,7	5	1	21	2000
13,2	17,1	0,7	2	1	25	2008
13,2	17,1	0,7	3	1	20	2011
17,3	38,5	0,7	10	1	16	2008
15	25,1	0,7	7	1	20	1999

15	25,1	0,7	5	1	19	2012
13,3	17,5	0,7	4	1	26	1999
14,2	21,3	0,7	3	1	26	2000
18	43,4	0,7	8	1	22	2013
16	30,5	0,7	4	1	20	2013
13,9	20	0,7	4	1	26	2005
17,6	40,6	0,7	6	1	20	2013
17,2	37,9	0,7	6	1	22	2013
12,1	13,2	0,7	2	1	25	2002
16,5	33,5	0,7	6	1	20	2013
13,8	19,6	0,7	2	1	27	1999
16,4	32,9	0,7	6	1	19	2011
14,7	23,7	0,7	3	1	24	2001
15,1	25,7	0,7	9	1	21	1999
17,8	42,1	0,7	7	1	18	2012
14,8	24,2	0,7	5	1	23	2007
15	25,2	0,7	6	1	23	2008
17,2	38	0,7	6	1	22	2013
12,7	15,3	0,7	2	1	22	2002

12,7	15,3	0,7	3	1	25	2008
12,7	15,3	0,7	3	1	25	2008
14	20,5	0,7	3	1	25	2007
14	20,5	0,7	3	1	25	2008
16,7	34,8	0,7	8	1	21	2001
13,1	16,8	0,7	3	1	25	2007
11,2	10,5	0,7	2	1	22	2004
12,5	14,6	0,7	2	1	23	2002
18	43,6	0,7	8	1	20	2013
13,2	17,2	0,7	3	1	22	2002
13,5	18,4	0,7	4	1	26	1999
13,5	18,4	0,7	2	1	26	2000
18,8	49,7	0,7	9	1	17	2012
16,4	33	0,7	8	1	19	2006
13,9	20,1	0,7	2	1	25	2002
12,2	13,6	0,7	3	1	23	2005
14,1	21	0,7	4	1	24	2006
14,1	21	0,7	6	1	20	2008
11,7	12	0,7	2	1	22	2002

15,1	25,8	0,7	9	1	20	2000
18,2	45,2	0,7	8	1	22	2013
16,5	33,7	0,8	7	1	18	2012
12,1	13,3	0,8	2	1	25	2007
10,7	9,2	0,8	2	1	25	2004
13	16,5	0,8	2	1	25	2002
17	36,9	0,8	6	1	21	2013
13,5	18,5	0,8	7	1	27	1999
13,5	18,5	0,8	2	1	25	2005
16	30,8	0,8	5	1	19	2012
12,3	14	0,8	3	1	23	2005
14,3	22	0,8	5	1	23	1999
16,1	31,4	0,8	5	1	18	2012
14,1	21,1	0,8	2	1	21	2000
14,1	21,1	0,8	3	1	21	2012
16,3	32,6	0,8	6	1	20	2011
18,2	45,4	0,8	6	1	22	2013
17	37	0,8	8	1	18	2012
17	37	0,8	5	1	20	2013

15,6	28,6	0,8	4	1	17	2008
18,3	46,2	0,8	6	1	19	2013
16,6	34,5	0,8	6	1	19	2013
14	20,7	0,8	4	1	27	1999
15,7	29,2	0,8	4	1	22	2011
16,2	32,1	0,8	4	1	18	2012
15	25,5	0,8	5	1	21	2012
13	16,6	0,8	3	1	15	2015
13,1	17	0,8	6	1	25	2008
13,3	17,8	0,8	2	1	25	2008
15,3	27,1	0,8	4	1	25	2002
14,4	22,6	0,8	5	1	27	1999
16,5	34	0,8	5	1	18	2000
13,8	19,9	0,8	5	1	27	1999
13,8	19,9	0,8	6	1	25	2008
12,5	14,8	0,8	2	1	22	2002
15,8	29,9	0,8	5	1	21	2012
14,6	23,6	0,8	8	1	26	1999
15	25,6	0,8	3	1	25	2002

15,6	28,8	0,8	3	1	19	2000
11	10,1	0,8	2	1	23	2005
16,5	34,1	0,8	4	1	18	2012
16	31,1	0,8	4	1	19	2013
16,2	32,3	0,8	5	1	19	2012
12,6	15,2	0,8	3	1	22	2002
13	16,7	0,8	3	1	25	2008
13,1	17,1	0,8	4	1	25	2008
13,3	17,9	0,8	2	1	25	2002
17,5	40,8	0,8	6	1	20	2013
14,2	21,8	0,8	3	1	25	2002
12,7	15,6	0,8	2	1	22	2002
14	20,9	0,8	4	1	25	2002
11,7	12,2	0,8	2	1	26	1999
14,7	24,2	0,8	3	1	23	2007
16,1	31,8	0,8	9	1	20	2013
20,1	61,9	0,8	5	1	17	2012
22,3	84,6	0,8	10	1	18	2012
12,5	14,9	0,8	2	1	21	2009

12,8	16	0,8	3	1	21	2014
15,8	30,1	0,8	10	1	23	2002
10,2	8,1	0,8	2	1	19	2007
13,6	19,2	0,8	4	1	21	2008
20,1	62	0,8	10	1	19	2012
14,4	22,8	0,8	5	1	22	2002
15,4	27,9	0,8	5	1	24	2006
19	52,4	0,8	7	1	18	2012
16,4	33,7	0,8	4	1	19	2013
14,5	23,3	0,8	5	1	21	2012
13	16,8	0,8	2	1	22	2002
14,2	21,9	0,8	4	1	25	2008
12,6	15,3	0,8	2	1	25	2002
12,6	15,3	0,8	3	1	23	2008
14,8	24,8	0,8	7	1	20	1999
14	21	0,8	4	1	21	2012
15,5	28,5	0,8	5	1	21	2000
15,5	28,5	0,8	5	1	25	2002
15,5	28,5	0,8	6	1	21	2003

15,8	30,2	0,8	5	1	19	2012
14,3	22,4	0,8	5	1	26	1999
13,7	19,7	0,8	5	1	22	2002
18,1	45,5	0,8	10	1	22	2013
14,5	23,4	0,8	4	1	22	2002
18	44,8	0,8	6	1	20	2013
13,8	20,2	0,8	3	1	22	2002
13	16,9	0,8	3	1	26	2005
15,1	26,5	0,8	4	1	19	2012
13,7	19,8	0,8	3	1	25	2008
20,4	65,4	0,8	8	1	18	2012
15,3	27,6	0,8	4	1	25	2002
16,9	37,2	0,8	7	1	21	2001
16,3	33,4	0,8	5	1	18	2012
13,6	19,4	0,8	2	1	25	2008
15,6	29,3	0,8	5	1	21	2000
12,8	16,2	0,8	2	1	25	2002
19,2	54,7	0,8	8	1	21	2013
13,4	18,6	0,8	2	1	21	2000

13,4	18,6	0,8	2	1	22	2002
15	26,1	0,8	6	1	19	2008
12,3	14,4	0,8	5	1	21	2006
13,7	19,9	0,8	2	1	26	2000
13,1	17,4	0,8	2	1	25	2002
15,6	29,4	0,8	9	1	20	2000
11,6	12,1	0,8	3	1	26	2005
14,2	22,2	0,8	3	1	23	2001
15,5	28,9	0,8	8	1	20	2013
13,4	18,7	0,8	2	1	21	2000
15,2	27,3	0,8	6	1	21	1999
14,5	23,7	0,8	3	1	25	2002
14,7	24,7	0,8	6	1	20	1999
19,1	54,2	0,8	6	1	20	2013
13	17,1	0,8	3	1	25	2008
15,1	26,8	0,8	5	1	18	1999
13,1	17,5	0,8	3	1	26	2005
19,2	55,1	0,8	6	1	21	2012
14,2	22,3	0,8	3	1	25	2002

12,1	13,8	0,8	3	1	26	2005
14,4	23,3	0,8	6	1	21	1999
14,7	24,8	0,8	6	1	21	2000
12,7	16	0,8	3	1	21	2015
13,4	18,8	0,8	2	1	25	2002
13,4	18,8	0,8	2	1	25	2002
13,4	18,8	0,8	3	1	25	2008
15,5	29,1	0,8	3	1	20	2003
13,2	18	0,8	3	1	25	2008
16,3	33,9	0,8	5	1	23	2006
18,2	47,2	0,8	8	1	19	2012
11,4	11,6	0,8	2	1	23	2007
19,6	59	0,8	9	1	22	2013
10,7	9,6	0,8	2	1	25	2004
17,4	41,3	0,8	10	1	19	2012
18,8	52,1	0,8	6	1	20	2013
15,1	27	0,8	4	1	22	2013
13,5	19,3	0,8	4	1	22	1999
12,6	15,7	0,8	2	1	22	2002

13,7	20,2	0,8	3	1	20	2013
12,7	16,1	0,8	2	1	25	2008
13,6	19,8	0,8	3	1	26	2005
13,6	19,8	0,8	3	1	25	2008
13,1	17,7	0,8	3	1	26	2005
13,5	19,4	0,8	3	1	25	2008
14,6	24,6	0,8	5	1	27	1999
13,2	18,2	0,8	2	1	25	2008
15,5	29,5	0,8	7	1	19	2012
13	17,6	0,8	2	1	22	2002
14,7	25,5	0,8	5	1	22	2002
14,7	25,5	0,8	8	1	20	2011
17,7	44,6	0,8	10	1	19	2012
13,5	19,8	0,8	4	1	25	2008
13,5	19,8	0,8	3	1	25	2008
12,6	16,1	0,8	3	1	20	2013
19,2	57	0,8	6	1	21	2013
13	17,7	0,8	3	1	25	2005
14,3	23,6	0,8	4	1	20	2013

10,4	9,1	0,8	1	1	26	2009
12,9	17,4	0,8	2	1	22	2002
13,7	20,9	0,8	4	1	26	2005
13,2	18,7	0,8	4	1	23	2005
17,3	42,1	0,8	10	1	19	2013
13,1	18,3	0,8	2	1	25	2002
13,4	19,6	0,8	2	1	22	2002
12,6	16,3	0,8	2	1	25	2008
10,4	9,2	0,8	1	1	23	2015
14,7	26	0,8	5	1	19	2000
11	10,9	0,8	1	1	25	2008
12,9	17,6	0,8	4	1	24	2006
17,3	42,5	0,8	10	1	21	2012
13,5	20,2	0,8	4	1	21	2012
12,1	14,6	0,8	2	1	25	2002
14,3	24,1	0,8	4	1	22	2011
13,8	21,7	0,8	4	1	26	2005
12	14,3	0,8	2	1	22	2002
13,7	21,3	0,8	3	1	25	2008

12,8	17,4	0,8	2	1	25	2002
14,7	26,4	0,8	4	1	25	2008
14,2	23,8	0,8	5	1	21	2012
15	28,1	0,8	6	1	20	2013
14	22,9	0,8	4	1	22	2011
15,2	29,4	0,8	5	1	19	2012
14	23	0,8	3	1	25	2002
12,8	17,6	0,8	4	1	23	2006
12,7	17,2	0,8	2	1	25	2008
11,2	11,8	0,8	2	1	26	2005
13,3	19,8	0,8	6	1	25	2008
16,1	35,3	0,8	6	1	22	2013
14,1	23,8	0,8	5	1	18	2012
12,3	15,8	0,8	2	1	25	2002
18,1	50,6	0,9	7	1	18	2012
15,7	33,1	0,9	7	1	18	2012
14,5	26,1	0,9	5	1	19	2012
12,7	17,6	0,9	3	1	25	2008
16,3	37,3	0,9	7	1	19	2012

13,1	19,4	0,9	4	1	26	2005
10,1	8,9	0,9	1	1	26	2001
14,3	25,4	0,9	6	1	25	2008
16,2	37,3	0,9	6	1	21	2013
10,4	9,9	0,9	2	1	23	2006
13,9	23,7	0,9	5	1	19	2007
14	24,3	0,9	4	1	25	2005
18,3	54,5	0,9	7	1	18	2012
16,6	41,3	0,9	7	1	19	2012
14,1	25,4	0,9	5	1	26	1999
13,9	24,5	0,9	3	1	18	2004
17	45	0,9	10	1	22	2013
15,4	33,6	0,9	5	1	18	2012
15,3	34,1	1,0	5	1	20	2007
15,1	33,1	1,0	3	1	19	2000
12,7	20,8	1,0	3	1	20	2003
9,6	5	0,6	1	0	21	2006
7,3	2,3	0,6	1	0	23	2010
7,3	2,3	0,6	1	0	23	2010

11,6	10,2	0,7	1	0	26	2001
11,9	7,2	0,4	1		18	2007
9	3,5	0,5	1		22	2011
11,8	8,2	0,5	3		21	2005
12	8,8	0,5	2		18	2007
10	5,2	0,5	1		22	2013
9,7	4,8	0,5	1		20	2003
10,8	6,7	0,5	1		22	2007
9	3,9	0,5	1		21	2009
9,4	4,5	0,5	1		21	2005
9,6	4,8	0,5	1		25	2003
14,9	18,1	0,5	3		25	2003
11,6	8,8	0,6	2		19	2007
14,5	17,8	0,6			20	2011
8	3	0,6	1		25	2003
12,1	10,4	0,6	2		20	2008
7,9	3	0,6	1		25	2005
9,3	5	0,6	1		19	2006
13,3	14,7	0,6	2		23	2007

9,7	5,8	0,6	1	26	2009
11,7	10,2	0,6	1	26	2001
9,8	6	0,6	2	23	2004
10,6	7,6	0,6	1	26	2001
10,3	7	0,6	1	26	2009
9,3	5,2	0,6	1	21	2008
10,1	6,7	0,7	1	26	2001
12,2	11,9	0,7	2	23	2007
7,7	3	0,7	1	25	2005
8,7	4,4	0,7	1	26	2003
7,3	2,6	0,7	1	25	2005
14	18,5	0,7	3	24	2001
8,6	4,3	0,7	1	26	2003
8,4	4,2	0,7	1	23	2003
9,6	6,4	0,7	1	26	2003
8,2	4	0,7	1	25	2003
13,8	20,1	0,8	4	26	1999
