# Supplementary material for: Thermal conditions and age structure determine the spawning regularities and condition of Baltic herring (Clupea harengus membras) in the NE of the Baltic Sea
Source: PeerJ. 2019 Jul 22;7:e7345. doi: 10.7717/peerj.7345 (PMC6657675; doi:10.7717/peerj.7345)
Supplement: Appendix 2 — The start of herring spawning, end of spawning, winter air temperature and larval occurrence in 1947-2015. [file peerj-07-7345-s002.pdf]

The start of herring spawning, end of spawning, winter air temperature and larval occurrence in 1947-2015.

Year	Start of spawning	End of spawning	Winter air temperature	Larval occurence
1947	19	26	-26,4	21
1948	18	27	-13,9	22
1949	17	27	-4,5	19
1953	19	28	-15,3	21
1954	18	27	-18,4	21
1955	20	27	-16,0	19
1964	18	27	-14,6	20
1965	18	28	-13,6	20
1966	18	28	-21,9	21
1974	17	26	-2,8	20
1975	17	26	-1,4	18
1976	18	28	-18,0	20
1984	18	28	-10,0	20
1985	18	28	-26,8	20
1986	18	27	-15,2	20
1999	18	29	-7,7	20
2000	18	28	-2,4	19
2001	18	27	-6,9	19
2002	17	28	-0,7	19
2003	20	29	-12,3	20
2004	18	27	-9,6	20
2005	19	24	-12,6	20
2006	19	31	-16,9	21
2007	18	25	-6,8	19
2008	16	26	1,7	18
2009	17	25	-6,9	19
2010	19	26	-21,9	22
2011	19	26	-15,0	21
2012	17	25	-13,1	20
2013	19	27	-14,9	21
2014	17	27	-3,10	19
2015	15	23	3,3	19
