# Supplementary material for: Thermal conditions and age structure determine the spawning regularities and condition of Baltic herring (Clupea harengus membras) in the NE of the Baltic Sea
Source: PeerJ. 2019 Jul 22;7:e7345. doi: 10.7717/peerj.7345 (PMC6657675; doi:10.7717/peerj.7345)
Supplement: Appendix 3 — The number of young (2-3 yr old) and old (4-8+ yr old) herring in spawning stock (ICES, 2018). [file peerj-07-7345-s003.pdf]

The number of young (2-3 yr old) and old (4-8+ yr old) herring in spawning stock (ICES 2018).

YEAR	1999	2000	2001	2002	2003	2004	2005	2006	2007	2008	2009	2010	2011	2012	2013	2014	2015
**1**	288547	263537	607175	226183	697117	101490	314097	686923	199132	539304	276442	278640	108843	487116	526068	92167	214909
**2**	204812	203497	192328	422963	157662	516756	68004	222478	491116	135431	387306	201512	185677	80552	357292	391327	68496
**3**	65079	127285	116635	115700	239279	93234	301653	37802	127848	298400	80104	247839	128077	120671	54733	238541	270285
**NO 2-3 YR**	**269891**	**330782**	**308963**	**538663**	**396941**	**609990**	**369657**	**260280**	**618964**	**433831**	**467410**	**449351**	**313754**	**201223**	**412025**	**629868**	**338781**
**4**	114303	39072	71689	61275	62104	112403	48627	167927	20670	64745	180296	47439	156270	75371	73822	36162	155177
**5**	50097	64947	20794	35074	31879	29928	46264	24301	95197	9332	37984	102849	28887	91854	43965	47443	23406
**6**	21103	26792	29823	9680	17953	16379	12816	21043	10766	50439	5662	21385	59257	15867	52996	28137	28369
**7**	18113	9709	14336	13470	4640	8319	7584	5883	10857	3413	31115	2782	12475	33312	9432	31762	17421
**8+**	23973	14605	17891	12471	13444	18191	7756	7092	7718	7655	8939	26943	15596	12389	25423	23002	32054
**NO 4+**	**227589**	**155125**	**154533**	**131970**	**130020**	**185220**	**123047**	**226246**	**145208**	**135584**	**263996**	**201398**	**272485**	**228793**	**205638**	**166506**	**256427**
